# Health and economic impact of seasonal influenza mass vaccination strategies in European settings: A mathematical modelling and cost-effectiveness analysis

**DOI:** 10.1016/j.vaccine.2022.01.015

**Published:** 2022-02-23

**Authors:** Frank G. Sandmann, Edwin van Leeuwen, Sibylle Bernard-Stoecklin, Itziar Casado, Jesús Castilla, Lisa Domegan, Alin Gherasim, Mariëtte Hooiveld, Irina Kislaya, Amparo Larrauri, Daniel Levy-Bruhl, Ausenda Machado, Diogo F.P. Marques, Iván Martínez-Baz, Clara Mazagatos, Jim McMenamin, Adam Meijer, Josephine L.K. Murray, Baltazar Nunes, Joan O'Donnell, Arlene Reynolds, Dominic Thorrington, Richard Pebody, Marc Baguelin

**Affiliations:** a UK Health Security Agency, London NW9 5EQ, United Kingdom.; b Department of Infectious Disease Epidemiology, London School of Hygiene and Tropical Medicine (LSHTM), London, UK; c School of Public Health, Imperial College London, London, UK; d Santé Publique France, Saint Maurice, France; e Instituto de Salud Pública de Navarra – IdiSNA, CIBERESP, Pamplona, Spain; f Health Service Executive-Health Protection Surveillance Centre, Dublin, Ireland; g National Centre of Epidemiology/CIBER de Epidemiologia y Salud Pública (CIBERESP). Institute of Health Carlos III, Madrid, Spain; h Nivel (Netherlands Institute for Health Services Research), Utrecht, the Netherlands; i Instituto Nacional de Saúde Dr. Ricardo Jorge (INSA), Lisbon, Portugal; j Health Protection Scotland (HPS), Glasgow, UK; k Centre for Infectious Disease Research, Diagnostics and Laboratory Surveillance, National Institute for Public Health and the Environment (RIVM), Bilthoven, the Netherlands; l Haute Autorité de Santé (HAS), Saint-Denis, France

**Keywords:** Influenza, Vaccination, Mathematical model, Economic evaluation, Public health, Policy

## Abstract

**Introduction:**

Despite seasonal influenza vaccination programmes in most countries targeting individuals aged ≥ 65 (or ≥ 55) years and high risk-groups, significant disease burden remains. We explored the impact and cost-effectiveness of 27 vaccination programmes targeting the elderly and/or children in eight European settings (n = 205.8 million).

**Methods:**

We used an age-structured dynamic-transmission model to infer age- and (sub-)type-specific seasonal influenza virus infections calibrated to England, France, Ireland, Navarra, The Netherlands, Portugal, Scotland, and Spain between 2010/11 and 2017/18. The base-case vaccination scenario consisted of non-adjuvanted, non-high dose trivalent vaccines (TV) and no universal paediatric vaccination. We explored i) moving the elderly to “improved” (i.e., adjuvanted or high-dose) trivalent vaccines (iTV) or non-adjuvanted non-high-dose quadrivalent vaccines (QV); ii) adopting mass paediatric vaccination with TV or QV; and iii) combining the elderly and paediatric strategies. We estimated setting-specific costs and quality-adjusted life years (QALYs) gained from the healthcare perspective, and discounted QALYs at 3.0%.

**Results:**

In the elderly, the estimated numbers of infection per 100,000 population are reduced by a median of 261.5 (range across settings: 154.4, 475.7) when moving the elderly to iTV and by 150.8 (77.6, 262.3) when moving them to QV. Through indirect protection, adopting mass paediatric programmes with 25% uptake achieves similar reductions in the elderly of 233.6 using TV (range: 58.9, 425.6) or 266.5 using QV (65.7, 477.9), with substantial health gains from averted infections across ages. At €35,000/QALY gained, moving the elderly to iTV plus adopting mass paediatric QV programmes provides the highest mean net benefits and probabilities of being cost-effective in all settings and paediatric coverage levels.

**Conclusion:**

Given the direct and indirect protection, and depending on the vaccine prices, model results support a combination of having moved the elderly to an improved vaccine and adopting universal paediatric vaccination programmes across the European settings.

## Highlights

• Seasonal influenza vaccine programmes usually target at-risk and older individuals.
• We used an age-structured dynamic-transmission model for eight European settings.
• Older people benefit from adjuvanted or high-dose trivalent or quadrivalent vaccines.
• Adopting mass paediatric influenza vaccination is also likely to be cost-effective.
• Results rest on vaccine costs, willingness to vaccinate and unknown long-term effects.

## 1 Introduction

Seasonal influenza is a recurring public health concern, with an estimated 38–74 million episodes of infection,[1] 3.7–23 million hospitalisations,[1] and 0.29–0.65 million excess deaths across all ages each year globally[2]. The highest burden is seen in older adults through person-to-person transmission,[1], [2] and most countries have adopted annual mass vaccination programmes targeting adults over 65 (or 55) years and high risk populations.[3], [4] Despite many countries achieving moderate to high uptake in the elderly,[4] a significant burden of disease remains with the current generation of vaccines as outlined above, e.g. due to variable vaccine effectiveness across seasons. Moreover, given the infectious nature of influenza, vaccination programmes not only aiming directly at the elderly and adult clinical at-risk groups need to be considered but also programmes that confer indirect protection to these groups due to herd immunity effects.[5]

Children are considered an important potential vaccination target, because of their large number of contacts and high susceptibility, which could make them an important transmission route of influenza.[4], [6], [7], [8], [12] Despite some countries having adopted mass paediatric vaccination programmes for seasonal influenza (most notably the USA, UK, Ireland, and Finland),[4], [6] the majority of countries globally are relying on national vaccination programmes for high risk groups and the elderly.[4] In Europe, however, paediatric programmes are currently considered in several countries given the significant residual burden of disease in many seasons,[7] as well as the advances in vaccine development of quadrivalent and live-attenuated influenza vaccines and improved vaccines for the elderly in an adjuvanted, high-dose or cell-based form.

This study thus aimed to evaluate the health and economic impact of seasonal influenza mass vaccination programmes in several European countries to support decision making on optimal influenza vaccination strategies internationally. In order to reduce the burden of seasonal influenza virus infections, with a special focus on the elderly and children, we explored the impact of i) different vaccination programmes aimed at the elderly, ii) introducing paediatric mass vaccination, and iii) a combination of these vaccination programmes jointly focusing on the elderly and children.

## 2 Methods

### 2.1 Settings

**Fig. 1:**
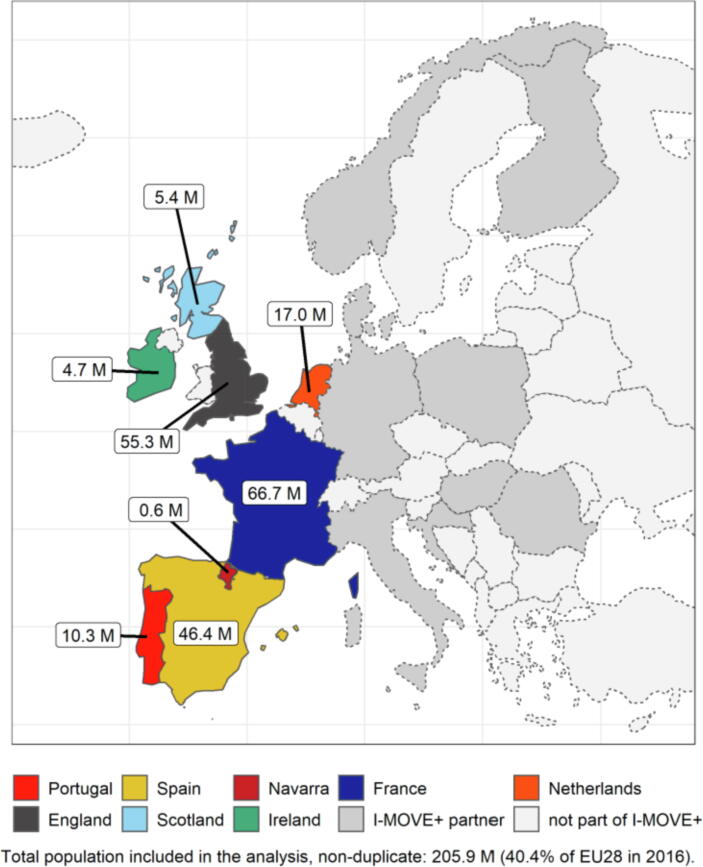
Settings included in the cost-effectiveness analysis, and all partner countries participating in the Integrated Monitoring of Vaccines in Europe project, I-MOVE+ .Note: Partner countries participating in the EU-funded I-MOVE+ project but not in this cost-effectiveness analysis are coloured in dark grey; countries not part of the I-MOVE+ project are coloured in light grey. Shapefiles with country borders were taken from Eurostat GISCO (https://ec.europa.eu/eurostat/web/gisco). EU: European Union, I-MOVE+: Integrated Monitoring of Vaccines in Europe project, M: million.

This analysis of seasonal influenza vaccination programmes was performed as part of the Integrated Monitoring of Vaccines in Europe project (I-MOVE+ ), a collaboration of 26 public health partners in 15 European countries. This study included eight self-selected partners in six countries of the I-MOVE+ collaboration that opted to participate in the cost-effectiveness analysis, and who were able to provide the data required for the modelling (Fig. 1). The combined population of the settings included 206 million individuals, representing 40% of the European Union (EU28).

### 2.2 Epidemiological model and statistical analysis

We used an age-structured dynamic-transmission compartmental model to simulate seasonal influenza virus epidemics that were calibrated to the age-specific number of influenza-like-illness (ILI) primary care consultations and virological confirmation of influenza virus infection for each setting and for each post-pandemic season from 2010/11 to 2017/18 (for France from 2014/15 due to changes in data collection). The model was based on a previously developed mathematical model of Susceptible-Exposed-Infected-Recovered compartments.[8], [9] We extended the model further to be specific to each setting, using an adaptive Markov chain Monte Carlo (MCMC) approach to infer the expected number of infections by age group, risk group, and influenza virus (sub-)type dependent on the profile of susceptibility/immunity and the ascertainment rate of cases (see Appendix).[10] The model synthesised data on primary care influenza surveillance,[11] virological tests for influenza, vaccination uptake and coverage, clinical risk groups for influenza, social contact mixing,[12], [13] and vaccine effectiveness (VE) estimates per age group, season, and influenza virus (sub-)type.[14]

We examined the resulting MCMC output by means of visual inspection of the model fit, trace plots of the inferred parameters, auto- and cross-correlation of parameters, and the effective sample size (ESS). Seasons with an ESS below 100 were excluded from the analysis to minimise autocorrelation (This concerned the results from ten of 180 seasons in total; see Appendix. Excluding the seasons changed results only slightly).

Based on the median estimated incidence by season we also calculated the Spearman rank correlation coefficients and their significance between settings for influenza A(H1N1)pdm09, A(H3N2) and influenza B using the logit-transformed fraction of infections.

### 2.3 Patients, interventions, and comparators

We explored a grand total of 27 mass vaccination strategies for each setting, including the base case consisting of an inactivated influenza vaccination programme with non-adjuvanted, non-high dose trivalent inactivated vaccine (TV) and no universal paediatric vaccination (Table 1). (For England and Scotland, where a mass paediatric vaccination programme was introduced in 2013/14, we still fitted the data using the actual vaccination uptake rates, but then used the inferred parameters to model the number of infections using the vaccination uptake rates pre-paediatric vaccination (2012/13) as the base case.) The base case was then compared to i) changing the vaccine for the elderly population (≥65 years), ii) adopting mass paediatric vaccination (4–16 years), and iii) combining each strategy for the elderly with each paediatric strategy.

**Table 1:** Mass vaccination strategies explored in this analysis for each setting.

Scenario	Vaccination strategy	Description
1	base case	• maintain influenza vaccination programme with non-adjuvanted, non-high dose trivalent vaccine (TV) • no universal paediatric vaccination^a^
2	elderly (iTV)	• change the vaccine for the elderly population from TV to an “improved” (adjuvanted or high-dose) trivalent vaccine (iTV) • no universal paediatric vaccination^a^
3	elderly (QV)	• change the vaccine for the elderly population from TV to a non-adjuvanted, non-high dose quadrivalent vaccine (QV) • no universal paediatric vaccination^a^
4	paed. (TV), 10%	• maintain influenza vaccination programme with TV • adopt mass paediatric vaccination with TV (at 10% coverage)
5	paed. (TV), 25%	• maintain influenza vaccination programme with TV • adopt mass paediatric vaccination with TV (at 25% coverage)
6	paed. (TV), 50%	• maintain influenza vaccination programme with TV • adopt mass paediatric vaccination with TV (at 50% coverage)
7	paed. (TV), 75%	• maintain influenza vaccination programme with TV • adopt mass paediatric vaccination with TV (at 75% coverage)
8	paed. (QV), 10%	• maintain influenza vaccination programme with TV • adopt mass paediatric vaccination with QV (at 10% coverage)
9	paed. (QV), 25%	• maintain influenza vaccination programme with TV • adopt mass paediatric vaccination with QV (at 25% coverage)
10	paed. (QV), 50%	• maintain influenza vaccination programme with TV • adopt mass paediatric vaccination with QV (at 50% coverage)
11	paed. (QV), 75%	• maintain influenza vaccination programme with TV • adopt mass paediatric vaccination with QV (at 75% coverage)
12	eld. (iTV) + paed. (TV), 10%	• change the vaccine for the elderly population from TV to iTV • adopt mass paediatric vaccination with TV (at 10% coverage)
13	eld. (iTV) + paed. (TV), 25%	• change the vaccine for the elderly population from TV to iTV • adopt mass paediatric vaccination with TV (at 25% coverage)
14	eld. (iTV) + paed. (TV), 50%	• change the vaccine for the elderly population from TV to iTV • adopt mass paediatric vaccination with TV (at 50% coverage)
15	eld. (iTV) + paed. (TV), 75%	• change the vaccine for the elderly population from TV to iTV • adopt mass paediatric vaccination with TV (at 75% coverage)
16	eld. (QV) + paed. (TV), 10%	• change the vaccine for the elderly population from TV to QV • adopt mass paediatric vaccination with TV (at 10% coverage)
17	eld. (QV) + paed. (TV), 25%	• change the vaccine for the elderly population from TV to QV • adopt mass paediatric vaccination with TV (at 25% coverage)
18	eld. (QV) + paed. (TV), 50%	• change the vaccine for the elderly population from TV to QV • adopt mass paediatric vaccination with TV (at 50% coverage)
19	eld. (QV) + paed. (TV), 75%	• change the vaccine for the elderly population from TV to QV • adopt mass paediatric vaccination with TV (at 75% coverage)
20	eld. (iTV) + paed. (QV), 10%	• change the vaccine for the elderly population from TV to iTV • adopt mass paediatric vaccination with QV (at 10% coverage)
21	eld. (iTV) + paed. (QV), 25%	• change the vaccine for the elderly population from TV to iTV • adopt mass paediatric vaccination with QV (at 25% coverage)
22	eld. (iTV) + paed. (QV), 50%	• change the vaccine for the elderly population from TV to iTV • adopt mass paediatric vaccination with QV (at 50% coverage)
23	eld. (iTV) + paed. (QV), 75%	• change the vaccine for the elderly population (from TV to iTV) • adopt mass paediatric vaccination with QV (at 75% coverage)
24	eld. (QV) + paed. (QV), 10%	• change the vaccine for the elderly population (from TV to QV) • adopt mass paediatric vaccination with QV (at 10% coverage)
25	eld. (QV) + paed. (QV), 25%	• change the vaccine for the elderly population from TV to QV • adopt mass paediatric vaccination with QV (at 25% coverage)
26	eld. (QV) + paed. (QV), 50%	• change the vaccine for the elderly population from TV to QV • adopt mass paediatric vaccination with QV (at 50% coverage)
27	eld. (QV) + paed. (QV), 75%	• change the vaccine for the elderly population from TV to QV • adopt mass paediatric vaccination with QV (at 75% coverage)

a: For England and Scotland, where a paediatric vaccination programme was introduced in 2013/14, we still fitted the data using the actual vaccination uptake rates, but then used the inferred parameters to model the number of infections using the vaccination uptake rates pre-paediatric vaccination (2012/13) as the base case.

eld.: elderly vaccination change (moving from TV to iTV or QV), iTV: “improved” trivalent vaccines (i.e., adjuvanted or high-dose), paed.: paediatric mass vaccination (scenario with specified vaccine and uptake rate), QV: quadrivalent vaccines (non-adjuvanted, non-high dose), TV: trivalent vaccines (non-adjuvanted, non-high dose).

For the base case, we used season-specific VE estimates published by the European Centre for Disease Prevention and Control (ECDC) in 2010/11 to 2016/17; in the absence of estimates for 2017/18 we used the pooled VE estimates of Belongia et al. (see Appendix).[14] For moving the elderly population to a different vaccine, we modelled either an “improved” (i.e., adjuvanted or high-dose) trivalent vaccine (iTV), or a non-adjuvanted non-high dose quadrivalent vaccine (QV). In the absence of robust head-to-head evidence on differences in VE between the adjuvanted trivalent vaccine and the high dose trivalent vaccine, we used the same values for both vaccines, which we termed the “improved” trivalent vaccine (iTV). For this vaccine, we used the relative efficacy of 24.2% reported for high dose trivalent vaccine over standard-dose trivalent vaccine.[15] For the QV that protects against an additional influenza type B strain than the TV, in the absence of reliable data, we upscaled the VE estimates for influenza virus type B using the relative ratio of the 95% upper confidence interval of the TV to the pooled central VE estimate of TV in Belongia et al. (2016),[14] with unchanged estimates from ECDC for the influenza virus (sub-)type A (see Appendix).

The universal paediatric vaccination programmes with either TV or QV were based on vaccinating children and adolescents aged 4–16 years as informed by previous research,[8], [9] and chosen to reflect the age of children in formal education across Europe.[16] We used uptake rates of 10%, 25%, 50%, and 75% to explore levels considered as plausible in the settings investigated.

### 2.4 Cost-effectiveness analysis

We conservatively adopted the perspective of the national healthcare provider, and we averaged all results to represent one season.[17] The model estimated the number of symptomatic ILI cases due to influenza virus infection,[18] the number of influenza-related outpatient consultation visits to a general practitioner (GP), the number of influenza-related hospitalisations, and the excess number of influenza-related deaths.[2]

The economic evaluation used the quality-adjusted life year (QALY) as the primary outcome measure, which was informed by the number of ILI cases, hospitalisations, and premature deaths due to influenza. QALY losses per non-fatal episode of illness were sourced from the literature,[19], [20], [21], [22] while we estimated the number of QALYs lost from premature mortality due to influenza using the age- and sex-specific life expectancies and utility norms of the general population in each setting (see Appendix).

For the costs, we included only the direct medical costs of the vaccine price and administration, outpatient (GP) consultations, and hospitalisations. We sourced published vaccine prices that differed by vaccine product and country (see Appendix). For the costs of the “improved” trivalent vaccine, we considered the highest price of the influenza vaccines available per setting plus an assumed additional 50% premium in the base case (informed by the ratios of quadrivalent to trivalent vaccine prices), but we also explored a comprehensive range of vaccine prices of €0-€40 per dose in scenario analysis (see Appendix). All costs represent 2017 euros (€), which we inflated and converted where necessary.

We performed an incremental cost-utility analysis (CUA) among the seasonal influenza strategies, using the base case as the starting point in all settings. We estimated the impact of the different vaccination scenarios based on the QALY gain from vaccination (i.e., the averted QALY loss due to influenza). We discounted QALY losses from premature mortality at 3% in line with WHO-CHOICE recommendations;[17] costs were not discounted as none of them were incurred beyond one year.[17]

### 2.5 Uncertainty analysis

For the different vaccination scenarios, we obtained the results of 5,000 iterations of the epidemiological model using as input the inferred set of parameters. In addition, we explored the parameter uncertainty of the other input parameters of the cost-effectiveness analysis using a probabilistic sensitivity analysis with Monte Carlo sampling and 5,000 random draws matching the epidemiological model.[23] We used the mean and variance of the provided data to inform the uncertainty of the distributions. In the absence of information on the measure of spread of parameters, the mean value of the data was used, which propagates a considerable amount of uncertainty through the model. We used log-normal distributions for costs, beta distributions for the influenza-(sub-)type specific proportions of infected cases with ILI symptoms and for utilities, and a log-normal distribution for the quality-adjusted life-years lost due to premature mortality.

Results were visualized using the cost-effectiveness acceptability curve (CEAC) and the cost-effectiveness acceptability frontier (CEAF). The CEAC shows the probability of interventions being cost-effective for a range of cost-per-QALY values, while the CEAF indicates the optimal intervention with the highest mean net benefit.[24] For the threshold analysis of the iTV price between €0-€40 per dose, we recalculated the vaccination costs with each price and present results based on the CEAF, indicating the 50% (median) and 90% probability of being both cost-effective and the optimal strategy.[25]

### 2.6 Software

All analyses were conducted in R using the R-packages Hmisc, tidyverse and fluEvidenceSynthesis.[10]

## 3 Results

The transmission-dynamic model matched the observed number of ILI-consultations per 100,000 population in most seasons, settings, and influenza virus (sub-)types, except for a few seasons with low numbers to fit the model that were excluded from further analysis (either due to poor model fit or low ESS, with minimal impact on results; see Appendix). The incidences showed similar peaks in the same seasons and (sub-)types across most settings, particularly for those without mass paediatric vaccination. The Spearman rank correlation coefficients revealed that the size of the influenza outbreaks were mostly positively correlated and were often significant in the eight studied settings, but without a clear trend between settings with and without mass paediatric vaccination programmes (see Appendix).

**Fig. 2:**
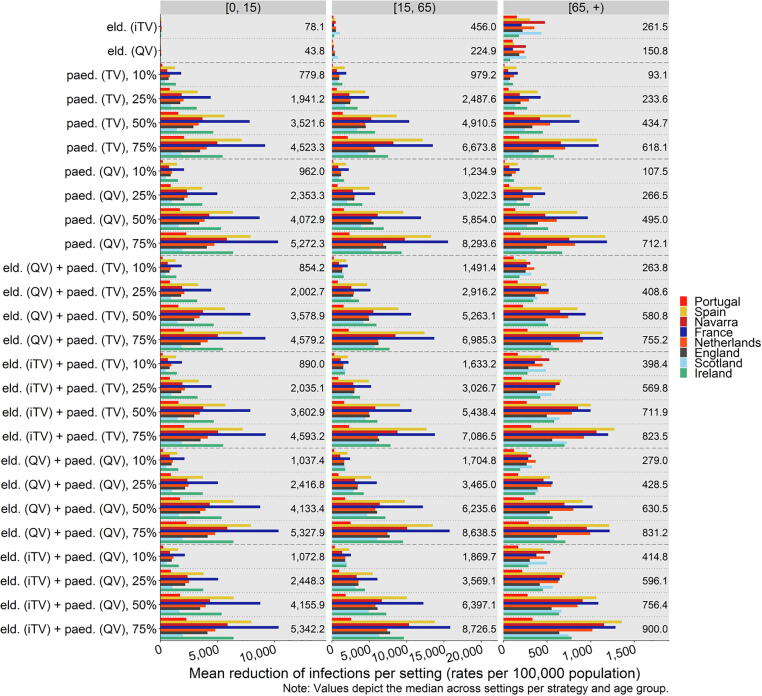
Mean reduction of influenza virus infections per 100,000 population across vaccination strategies and settings by age groups of children and adolescents (0–14 years), adults (15–64 years), and the elderly (65 + years). Note that the epidemiological model explored paediatric mass vaccination for individuals up to age 16.eld.: elderly vaccination change (moving from TV to iTV or QV), iTV: “improved” trivalent vaccine (i.e., adjuvanted or high-dose), paed.: paediatric mass vaccination (scenario with specified vaccine and uptake rate), QV: quadrivalent vaccine (non-adjuvanted, non-high dose), TV: trivalent vaccine (non-adjuvanted, non-high dose).

Based on the model fit for the base case, our model results suggest the mean number of infections in the elderly per 100,000 population reduces across settings by a median of 261.5 (range: 154.4, 475.7) when switching the elderly to an iTV, and by 150.8 (77.6, 262.3) when switching the elderly to QV (Fig. 2, and Appendix). Similar reductions in the elderly can be achieved through indirect protection from a mass paediatric vaccination programme (at an uptake level of 25%) of 233.6 (range: 58.9, 425.6) using TV, and of 266.5 (65.7, 477.9) using QV (Fig. 2, panel 3 “[65, +)”, and Appendix). For the paediatric age groups, however, the indirect protection from moving the elderly population to a different vaccine, without introducing mass paediatric vaccination, is marginal with 78.1 and 43.8 averted infections per 100,000 population (Fig. 2, panel 1 “[0, 15)”). Proportionally, all 27 vaccination strategies avert the highest numbers of infections in individuals aged 15–64 years (Fig. 2, panel 2 “[15, 65)”).

Generally across all ages, moving the elderly to an iTV leads to fewer infections than moving the elderly to QV, while introducing only a paediatric QV programme leads to fewer infections than a paediatric TV programme (Fig. 2); the highest reductions can be achieved with a combination of moving the elderly to iTV and the children to QV (Fig. 2).

**Fig. 3:**
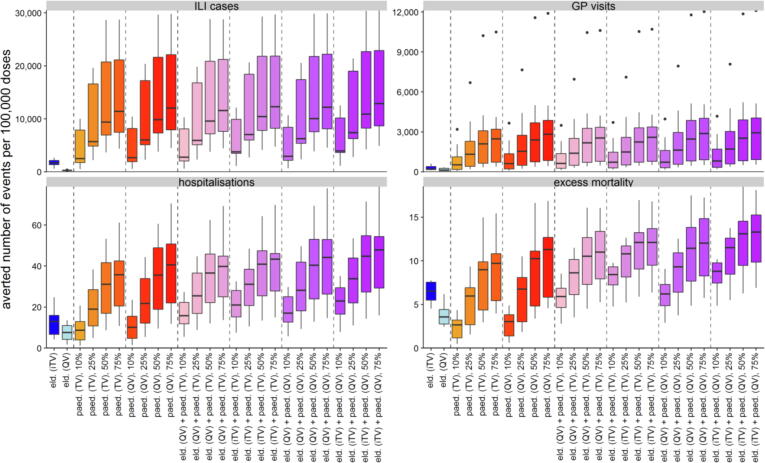
Mean number of events averted per 100,000 doses, across all ages and settings. Note that the wide range of uncertainty is reflecting the impact observed in different settings. Elderly solo programmes in blue, paediatric solo programmes in orange-red, combination programmes in pink-purple.eld.: elderly vaccination change (moving from TV to iTV or QV), GP: general practitioner, ILI: influenza-like illness, iTV: “improved” trivalent vaccine (i.e., adjuvanted or high-dose), paed.: paediatric mass vaccination (scenario with specified vaccine and uptake rate), QV: quadrivalent vaccine (non-adjuvanted, non-high dose), TV: trivalent vaccine (non-adjuvanted, non-high dose).

Across settings, a mass paediatric influenza vaccination programme at a paediatric vaccine uptake level of 25% is likely averting more influenza-related ILI cases and GP visits across all ages per dose than moving only the elderly population to a different vaccine (iTV or QV; Fig. 3, top row). For the more severe outcomes of hospitalisations and deaths, a mass paediatric programme is likely to provide similar reductions to the elderly programmes at an uptake of 25%, 50%, and 75% (hospitalisations) and 50% and 75% (excess deaths); see Fig. 3, bottom row. The highest mean numbers of events averted across all outcomes can be achieved with the combination programmes (Fig. 3). For results per setting see Appendix.

**Fig. 4:**
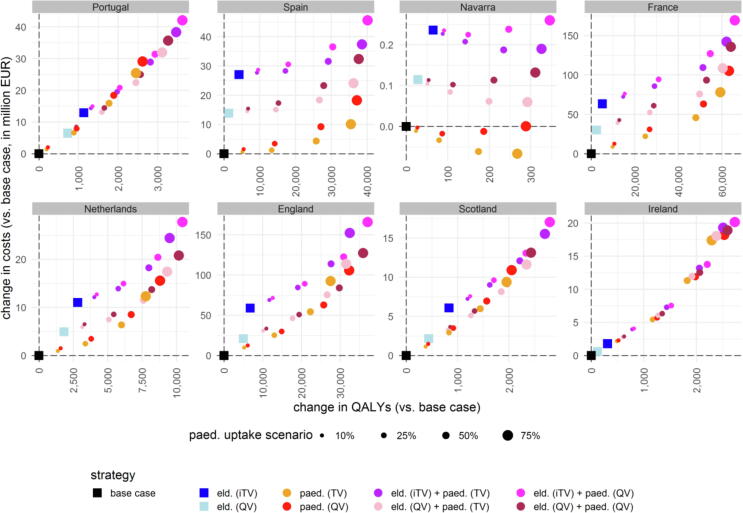
Change in total costs and QALYs per strategy in each setting.eld.: elderly vaccination change (moving from TV to iTV or QV), EUR: euros, iTV: “improved” trivalent vaccine (i.e., adjuvanted or high-dose), paed.: paediatric mass vaccination (scenario with specified vaccine and uptake rate), QALY: quality-adjusted life year, QV: quadrivalent vaccine (non-adjuvanted, non-high dose), TV: trivalent vaccine (non-adjuvanted, non-high dose).

Moving the elderly to an iTV resulted in more QALYs gained than a mass paediatric programme at 10% uptake in five settings (except in Spain, France and Ireland; Fig. 4), but at higher costs (except in Ireland; Fig. 4). Hospitalisations drove healthcare costs in Navarra, the Netherlands, Scotland and Ireland, while GP visits drove healthcare costs in Portugal, Spain, France, and England (see Appendix). However, the QALY gain from averted hospitalisations was negligible in all settings, with averted mortality being the driver of the QALY gain in Portugal, France, the Netherlands, England and Scotland (and non-fatal, non-hospitalised ILI cases being the driver in Spain, Navarra, and Ireland; see Appendix).

**Fig. 5:**
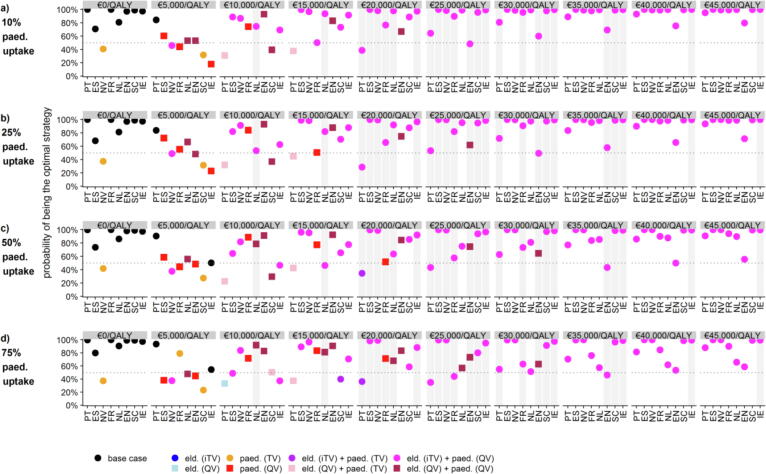
Optimal vaccination strategy for willingness-to-pay ranges of €0-€45,000/QALY per paediatric uptake scenario and per setting (based on cost-effectiveness acceptability frontier, CEAF). Note: Grey vertical bars indicate local cost-effectiveness thresholds used officially or unofficially in each settingCEAF: cost-effectiveness acceptability frontier, eld.: elderly vaccination change (moving from TV to iTV or QV), EN: England, ES: Spain, FR: France, IE: Ireland, iTV: “improved” trivalent vaccine (i.e., adjuvanted or high-dose), NL: Netherlands, NV: Navarra, paed.: paediatric mass vaccination (scenario with specified vaccine and uptake rate), PT: Portugal, QALY: quality-adjusted life year, QV: quadrivalent vaccine (non-adjuvanted, non-high dose), SC: Scotland, TV: trivalent vaccine (non-adjuvanted, non-high dose).

In terms of the probability of being cost-effective while realising the highest mean net benefit, the base case achieves the highest probability at €0/QALY gained (i.e., if healthcare providers are unwilling to pay anything extra for additional QALY gains) in seven settings (except Navarra), while adopting a paediatric TV solo programme is cost-saving in Navarra across paediatric uptake levels; see Fig. 5. At €15,000/QALY gained adopting a mass paediatric vaccination programme achieves the highest probability, with or without moving the elderly to an improved vaccine (Fig. 5). Moving the elderly to iTV plus adopting mass paediatric QV programmes provides the highest mean net benefits in all settings at €25,000/QALY gained (with 10% mass paediatric uptake), €30,000/QALY gained (25% mass paediatric uptake), and €35,000/QALY gained (50% or 75% mass paediatric uptake). Due to diminishing rates of returns of the herd effects the probability that the optimal vaccination strategies are cost-effective decreases as the paediatric mass vaccination coverage goes up.

Across the entire willingness-to-pay (WTP) ranges used officially or unofficially in each setting (Fig. 5, the grey shaded areas), the combination of moving the elderly to an iTV and adopting paediatric QV vaccination is the optimal option in all settings at an expected uptake of mass paediatric vaccination of 10%, and for Spain, Navarra, Ireland, and Scotland at all 4 uptake levels investigated (Fig. 5). Combining an elderly QV and mass paediatric QV programme may also be beneficial in England (at 25%, 50%, or 75% uptake) and the Netherlands (at 50% or 75% uptake), while combining an elderly iTV and mass paediatric TV programme may be beneficial in Portugal (at 50% or 75% uptake); France may benefit from adopting a mass paediatric QV programme alone (at 50% or 75% uptake; Fig. 5).

These findings are subject to the assumptions made for the iTV prices; at a willingness-to-pay of €25,000/QALY gained the median threshold price (excluding administration costs) may range between €10 and €24 per dose for the iTV used as part of the optimal combination programme in most settings and paediatric uptake rates (see Appendix; no median price is defined for Portugal at paediatric uptake of 75%). If requiring conservatively for 90% of simulations to be below the willingness-to-pay threshold, the price ranges between €7 and €18 per dose (where defined; see Appendix).

## 4 Discussion

This study explored the health and economic impact of 27 mass vaccination programmes targeting seasonal influenza virus infections in eight European settings. The results of the transmission-dynamic model suggest that although moving the elderly to an iTV is preferable over moving them to QV or keeping the base case of TV both in terms of the health and economic impact, a mass paediatric vaccination programme appears as good if not better than only moving the elderly to a different vaccine given the indirect herd protection (assuming vaccine uptake of 25%, 50%, or 75%). At a willingness-to-pay of €35,000/QALY gained, the highest net benefits and probabilities of being cost-effective across settings are predicted when moving the elderly to an iTV plus adopting paediatric QV programmes. This finding is subject to the assumptions made for the iTV effectiveness and price, which may be up to €20 per dose (depending on the setting) for the combination programme to be the optimal option and 90% of its simulations to be below a threshold of €25,000/QALY gained, a conservative rule applied similarly in England.[25] If the iTVs are going to be priced too far above our conservative base case assumptions, adopting QVs for the elderly plus introducing a paediatric vaccination programme with TV or QV becomes more cost-effective and the optimal strategy across settings and most paediatric uptake scenarios.

### 4.1 Strengths and limitations

Our population-level analysis is one of the first studies using a transmission-dynamic model to explore the impact and cost-effectiveness of adopting a universal paediatric vaccination programme with inactivated QV or TV and/or moving the elderly to an improved vaccine in Europe. Our study addresses an important issue regarding the direct and indirect benefits of paediatric vaccination, which has previously been demonstrated to be highly impactful in the USA.[26] Despite the increased complexity of using such models, it allowed us to consider the potential direct and indirect protection from vaccination. The results of this analysis may support decision-making on the optimal seasonal influenza vaccination programme and provide orientation in a fast-advancing field with various treatment options and vaccination strategies.

This study included a total population of 206 million individuals in eight European settings. In order to enhance comparability, we harmonised the analysis for all partner settings using the same epidemiological model, identical methodological choices (e.g. the discount rate and perspective), identical input categories for the costs and outcomes, and the same data sources for key input parameters (demographics, ILI rate, and excess mortality). Nonetheless, some data were unavailable and necessitated simplifying assumptions (e.g. extrapolating contact matrices from neighbouring countries and estimating utility values for France and Portugal). In the absence of knowing the exact coverage level realistically achievable we explored 4 uptake levels that match what has been observed in the eight settings. Furthermore, we did not explore increased coverage levels for the elderly given the observed difficulty in most countries of increasing and even maintaining observed coverage levels prior to the coronavirus disease 2019 (COVID-19) pandemic.[27] It is also unclear whether the substantial increase in influenza vaccine uptake in the elderly for the 2020/21 winter will be sustained in future.

We also had to make assumptions about the influenza virus type B VE estimates with the QV, in the absence of reliable data. Due to lack of data, we were also unable to split up the influenza virus type B lineages Yamagata- (B/Yam) and Victoria-like (B/Vic). Similarly, we used the relative effectiveness of high-dose TV for all iTV given the absence of robust head-to-head evidence, which is not the same as evidence of absence. Furthermore, without published VE estimates from ECDC for 2017/18 at the time of analysis we used the pooled estimates of Belongia et al., which may be biased as they included the 2009 pandemic season.[14] The impact on results is expected to be minimal, however, not least because changes in the vaccine coverage have previously been shown to be more impactful on the health burden than changes in VE.[28]

By including sub-country settings we were able to explore differences on different levels of aggregation that highlight the variability between settings in terms of influenza epidemiology, healthcare seeking behaviour, level of detail of surveillance, severity of hospitalised influenza cases, resource organisation of healthcare systems, and a possible link with climate and antibiotic consumption.[29], [30] Despite the quantitative differences, however, the main policy conclusions are near-identical across settings. Future work will look at including further countries and possibly other vaccination strategies such as the use of cell-based and live-attenuated vaccines, increased uptake in the elderly, in high risk groups and in health care workers and/or different age groups for the paediatric programmes (including adults aged 18–64 years and investigating any cocooning effect), and in the longer-term impact and effect of mass paediatric vaccination on other age groups by using a multi-season model and through direct observation of herd effects (which is especially relevant for newer vaccines that may confer immunity of longer than one year).

The analysis did not consider adverse events following immunization given the short duration of mild events and the rare occurrence of serious adverse events for seasonal influenza vaccines, nor did we include costs related to the communication and organisation efforts of vaccine delivery, productivity costs or costs borne by patients given the healthcare payer perspective. Furthermore, given the wide uncertainty intervals considered in probabilistic sensitivity analyses it seems unlikely to us for the main conclusions of this study to change substantially. Lastly, the analysis was conducted prior to the COVID-19 pandemic and did not include any short-term implications on the seasonal influenza epidemiology due to the impact of non-pharmaceutical interventions adopted in response to the COVID-19 pandemic together with the accumulation of susceptible individuals due to the absence of influenza circulation in winter 2020/21. The analysis also did not consider whether the COVID-19 pandemic will contribute to increased future influenza vaccine coverage and increased appreciation of the importance of seasonal influenza vaccines which may be sustained in the years to come. Given the prospect of COVID-19 becoming endemic, signals of immunity “blunting” from receiving repeated influenza vaccines, and the prospect of a universal influenza vaccine becoming available soon, future research should investigate the impact of combining vaccination programmes at regular intervals (e.g., biannually).

### 4.2 Policy implications

The findings of this study indicate that at current coverage levels, although it may be conceivable for these to increase following the COVID-19 pandemic, the elderly population can best be protected from human influenza virus infection by introducing a universal paediatric vaccination programme (alone, provided sufficient coverage can be achieved and maintained, or in combination with moving the elderly to an improved TV, i.e. an adjuvanted or high-dose trivalent vaccine). Moving the elderly to an improved TV also seems sensible given that there may be some seasons in which indirect protection of vaccinating children is insufficient to protect the elderly population, and with a previous study estimating the benefit of vaccinating the elderly instead of children improving with lower VE.[28] Next to the direct protection of young children and adolescents, introduction of a mass paediatric programme can result in comparable reductions of infections in the elderly as moving them to a different vaccine, at current coverage levels. Future studies should explore additional age groups,[26] and consider additional efforts aimed at improving the coverage levels in the existing programmes.

Currently, across all ages, the adoption of a mass paediatric vaccination programme, in combination with the added benefits of having switched the elderly to either iTV or QV, is crucial for getting the most value-for-money from a seasonal influenza vaccination programme. This analysis may provide a reference for informing policy changes and the additional costs and benefits to expect when moving to a different vaccination programme.

## 5 Conclusions

For the eight European settings included in this study, our model results indicate that indirect protection through introducing a mass paediatric influenza vaccination programme can result in comparable reductions of infections in the elderly as moving them to a different vaccine, though at considerable variation between settings. However, the mass paediatric programme has the added benefit of large overall reductions across all age groups. The highest mean net benefit can be achieved with a combination of introducing mass paediatric vaccination with QVs and moving the elderly to iTVs, although depending on the prices for the vaccines.

This study underlines the potential benefits of moving the elderly population to improved vaccines and adopting a universal paediatric influenza programme in a range of European settings, taking into account the available country-specific demography, epidemiology, and healthcare resource use across eight post-pandemic seasonal influenza seasons (four seasons for France). The conclusions are, however, subject to assumptions for the prices of the iTVs, the willingness to pay of the national healthcare providers, the willingness of the population to get vaccinated, and the unknown long-term impact of the vaccines.

## Declaration of Competing Interest

The authors declare that they have no known competing financial interests or personal relationships that could have appeared to influence the work reported in this paper.
