# Supplementary material for: Health and economic impact of seasonal influenza mass vaccination strategies in European settings: A mathematical modelling and cost-effectiveness analysis
Source: Vaccine. 2022 Feb 23;40(9):1306–15. doi: 10.1016/j.vaccine.2022.01.015 (PMC8861572; doi:10.1016/j.vaccine.2022.01.015)
Supplement: Supplementary data 1 [file mmc1.docx]

# Supplementary Appendix

[Input parameters of the transmission-dynamic model by setting 2](#_Toc76675141)

[Input parameters of the cost-effectiveness analysis by setting 5](#_Toc76675142)

[Model fit (aggregated) to the supplied data per setting 9](#_Toc76675143)

[Model fit (disaggregated) to the supplied data per setting 12](#_Toc76675144)

[Estimated sample sizes for the inference mixing by setting, season, and influenza virus subtype 36](#_Toc76675145)

[Spearman rank correlation coefficients between settings based on the incidence by season 37](#_Toc76675146)

[Intermediate base case results per setting 38](#_Toc76675147)

[Relative reductions in infections for the elderly per setting 41](#_Toc76675148)

[Relative reductions in infections across ages per setting 42](#_Toc76675149)

[Number of events averted per 100,000 doses per setting 50](#_Toc76675150)

[Direct medical costs (in million €) by cost category per setting 55](#_Toc76675151)

[QALY gains by category per setting 60](#_Toc76675152)

[Cost-effectiveness acceptability curve (CEAC) and frontier (CEAF) per setting 65](#_Toc76675153)

[Threshold analysis of the iTV price per dose (based on cost-effectiveness acceptability frontier, CEAF) 67](#_Toc76675154)

[CHEERS checklist - Items to include when reporting economic evaluations of health interventions 68](#_Toc76675155)

Input parameters of the transmission-dynamic model by setting

The transmission-dynamic model used an adaptive Markov chain Monte Carlo (MCMC) approach to infer the expected number of infections by age group, risk group, and influenza subtype. The model has been used previously to inform the decision of paediatric vaccination uptake in the UK.^1,2^ For the epidemiological model, we used the following set of parameters in all settings:

| Supplementary Table 1: Input parameters used in each setting | | | | |
| --- | --- | --- | --- | --- |
| Parameter | | | Value | Source |
| Latent period | | | 0.8 days | Ferguson et al. (2005)^3^ |
| Infectious period | | | 1.8 days | Ferguson et al. (2005)^3^ |
| Vaccine efficacy values: | | |  |  |
| Season | Age group | Subtype | Value  (in %) | Source |
| 2010/11 | [0, 15) | B | 62.9 | Kissling et al. (2011)^4^ |
| 2010/11 | [15, 60) | B | 63.7 | Kissling et al. (2011)^4^ |
| 2010/11 | [60, +) | B | 55.5 | Kissling et al. (2011)^4^ |
| 2010/11 | [0, 15) | H1N1 | 77.2 | Kissling et al. (2011)^4^ |
| 2010/11 | [15, 60) | H1N1 | 27.2 | Kissling et al. (2011)^4^ |
| 2010/11 | [60, +) | H1N1 | 72.3 | Kissling et al. (2011)^4^ |
| 2011/12 | [0, 15) | H3N2 | 19.4 | Kissling et al. (2013)^5^ |
| 2011/12 | [15, 60) | H3N2 | 63.3 | Kissling et al. (2013)^5^ |
| 2011/12 | [60, +) | H3N2 | 15.1 | Kissling et al. (2013)^5^ |
| 2012/13 | [0, 15) | B | 22.3 | Kissling et al. (2014)^6^ |
| 2012/13 | [15, 60) | B | 63.6 | Kissling et al. (2014)^6^ |
| 2012/13 | [60, +) | B | 44.0 | Kissling et al. (2014)^6^ |
| 2012/13 | [0, 15) | H1N1 | 36.5 | Kissling et al. (2014)^6^ |
| 2012/13 | [15, 60) | H1N1 | 55.6 | Kissling et al. (2014)^6^ |
| 2012/13 | [60, +) | H1N1 | 59.1 | Kissling et al. (2014)^6^ |
| 2012/13 | [0, 15) | H3N2 | 36.1 | Kissling et al. (2014)^6^ |
| 2012/13 | [15, 60) | H3N2 | 43.6 | Kissling et al. (2014)^6^ |
| 2012/13 | [60, +) | H3N2 | 37.3 | Kissling et al. (2014)^6^ |
| 2013/14 | [0, 15) | H1N1 | 64.4 | Valenciano et al. (2015)^7^ |
| 2013/14 | [15, 60) | H1N1 | 38.8 | Valenciano et al. (2015)^7^ |
| 2013/14 | [60, +) | H1N1 | 49.1 | Valenciano et al. (2015)^7^ |
| 2014/15 | [0, 15) | H1N1 | 62.1 | Valenciano et al. (2016)^8^ |
| 2014/15 | [15, 60) | H1N1 | 41.4 | Valenciano et al. (2016)^8^ |
| 2014/15 | [60, +) | H1N1 | 50.4 | Valenciano et al. (2016)^8^ |
| 2014/15 | [0, 15) | H3N2 | 73.1 | Valenciano et al. (2016)^8^ |
| 2014/15 | [15, 60) | H3N2 | 59.7 | Valenciano et al. (2016)^8^ |
| 2014/15 | [60, +) | H3N2 | 22.4 | Valenciano et al. (2016)^8^ |
| 2015/16 | [0, 15) | B | -47.6 | Kissling et al. (2018)^9^ |
| 2015/16 | [15, 65) | B | 27.3 | Kissling et al. (2018)^9^ |
| 2015/16 | [65, +) | B | 9.3 | Kissling et al. (2018)^9^ |
| 2015/16 | [0, 15) | H1N1 | 31.9 | Kissling et al. (2018)^9^ |
| 2015/16 | [15, 65) | H1N1 | 41.4 | Kissling et al. (2018)^9^ |
| 2015/16 | [65, +) | H1N1 | 13.2 | Kissling et al. (2018)^9^ |
| 2016/17 | [0, 15) | H3N2 | 44.1 | Kissling, Rondy, and Team (2017)^10^ |
| 2016/17 | [15, 65) | H3N2 | 46.9 | Kissling, Rondy, and Team (2017)^10^ |
| 2016/17 | [65, +) | H3N2 | 23.4 | Kissling, Rondy, and Team (2017)^10^ |
| 2017/18 | [0, 20) | B | 56 | Belongia et al. (2016)^11^ |
| 2017/18 | [20, 65) | B | 54 | Belongia et al. (2016)^11^ |
| 2017/18 | [65, +) | B | 63 | Belongia et al. (2016)^11^ |
| 2017/18 | [0, 20) | H1N1 | 69 | Belongia et al. (2016)^11^ |
| 2017/18 | [20, 65) | H1N1 | 73 | Belongia et al. (2016)^11^ |
| 2017/18 | [65, +) | H1N1 | 62 | Belongia et al. (2016)^11^ |
| 2017/18 | [0, 20) | H3N2 | 43 | Belongia et al. (2016)^11^ |
| 2017/18 | [20, 65) | H3N2 | 35 | Belongia et al. (2016)^11^ |
| 2017/18 | [65, +) | H3N2 | 24 | Belongia et al. (2016)^11^ |
| EN: England, ES: Spain, FR: France, IE: Ireland, iTV: “improved” trivalent vaccines (i.e., adjuvanted or high-dose), NL: Netherlands, NV: Navarra, PT: Portugal, QV: quadrivalent vaccines (non-adjuvanted, non-high dose), SC: Scotland, TV: trivalent vaccines (non-adjuvanted, non-high dose). | | | | |

We also used setting-specific parameters for the primary care influenza surveillance, virological data for influenza, vaccination uptake and coverage, clinical risk groups for influenza, and social contact mixing. Key population statistics across settings are shown in Supplementary Table 2.

| Supplementary Table 2: Key statistics across settings, 2010/11 to 2017/18. | | | | | | | | |
| --- | --- | --- | --- | --- | --- | --- | --- | --- |
|  | **PT** | **ES** | **NV** | **FR** | **NL** | **EN** | **SC** | **IE** |
| Timeframe of the data | 2010/11-2017/18 | 2010/11-2017/18 | 2010/11-2017/18 | 2014/15-2017/18 | 2010/11-2017/18 | 2010/11-2017/18 | 2010/11-2017/18 | 2010/11-2017/18 |
|  |  |  |  |  |  |  |  |  |
| Population, total (in M.)^a^ | 10.5 | 46.6 | 0.6 | 65.8 | 16.8 | 53.9 | 5.3 | 4.6 |
| Population, 4-16 y.^b^ | 13.2% | 13.0% | 14.2% | 16.2% | 15.1% | 15.1% | 13.9% | 17.9% |
| Population, 65+ y.^c^ | 19.6% | 17.9% | 18.7% | 17.8% | 17.0% | 17.2% | 17.7% | 12.4% |
|  |  |  |  |  |  |  |  |  |
| mean coverage, total^a^ | 17.7% | 10.1% | 15.7% | 10.7% | 19.7% | 17.6% | 22.7% | 6.7% |
| mean coverage, 0-14 y.^d^ | 5.2% | 0.0% | 3.4% | 0.9% | 2.3% | 4.7% | 5.5% | 0.0% |
| mean coverage, 15-64 y.^d^ | 10.6% | 0.0% | 6.6% | 2.2% | 11.5% | 6.3% | 12.3% | 0.0% |
| mean coverage, 65+ y.^d^ | 50.9% | 56.4% | 57.8% | 49.4% | 69.4% | 74.0% | 77.3% | 53.7% |
| EN: England, ES: Spain, FR: France, IE: Ireland, iTV: “improved” trivalent vaccines (i.e., adjuvanted or high-dose), NL: Netherlands, NV: Navarra, PT: Portugal, QV: quadrivalent vaccines (non-adjuvanted, non-high dose), SC: Scotland, TV: trivalent vaccines (non-adjuvanted, non-high dose).  a: The values for the population and the coverage represent mean values between 2010/11 and 2017/18. The analysis used season-specific input values.  b: Target group of the mass paediatric vaccination scenarios with TV or QV.  c: Target group of changing the vaccine for the elderly population from TV to iTV or QV.  d: Age groups chosen to match Figure 2 in the main text. | | | | | | | | |

The original study used parameter specific priors for the transmission rate and the ascertainment rate.^1^ To generalise to multiple settings we converted their prior on the transmission rate into a prior on the basic reproductive number, resulting in a normal distribution with mean 1.9 and standard deviation of 0.25. We also implemented a prior on the ascertainment rate. The main contributing factors to the ascertainment rate are the symptomatic rate, the GP visitation rate when symptomatic and the swab positivity rate. For the symptomatic rate we used the (sub-)type specific results from Carrat et al.^12^ For the GP visitation rate age group specific rates were available only for the Netherlands.^13^ As such, we based the relative visitation rate in each age group on the data from the Netherlands, but the mean visitation rate was assumed to be specific for each setting.^14^ Where no data was available we used values from culturally similar settings, i.e. for Navarra the results of Spain were used and for Ireland and Scotland the results of England. Finally, the swab positivity rate was based on the previous study by Lieberman et al. (2009).^15^

For the primary care influenza surveillance, the age-stratified weekly data of ILI GP consultations and the total population monitored by the GPs were obtained from I-MOVE+ project partners or reports sent to WHO/ECDC.^16^ The age-stratified weekly virological data on test-positives and the total number of tests were supplied by I-MOVE+ project partners. Weekly vaccination uptake and coverage were used where available; for Portugal, Spain and France only the cumulative end-of-season coverage was available, which we scaled up into weekly estimates by using the uptake profile of England. For the clinical risk groups of influenza, the proportions of the total population for each age group that have comorbidities increasing the risk of influenza-related complications was available for each setting. For the social contact mixing, in the absence of alternatives we relied on the POLYMOD data for all settings but France.^17,18^ POLYMOD was conducted in the following eight countries: Belgium, Germany, Finland, the United Kingdom, Italy, Luxembourg, the Netherlands, and Poland. We assumed that the data of Italy serves as a reasonable proxy for Portugal, Spain, and Navarra, while we assumed that the data of the UK approximate that of Ireland.

Input parameters of the cost-effectiveness analysis by setting

The cost-effectiveness analysis adopted the time horizon of one year, which captures the vast majority of benefits and costs of seasonal influenza and is in line with the recommendations of WHO.^19,20^ In order to adjust for changes in severity in-between seasons, we averaged the impact of vaccination for all eight post-pandemic seasons supplied by each partner (four seasons were used for France to account for changes in data collection in 2014). The minimum number of seasons included for each setting is thus in line with recommendations by WHO.^19,20^

### Influenza-related natural outcomes (excluding QALYs)

For the economic evaluation, we estimated four natural outcome measures attributable to influenza-like illness (ILI): the number of symptomatic cases, influenza-related outpatient visits (i.e., consultations of general practitioners, GPs), influenza-related hospitalisations, and influenza-related excess deaths. Where available, we used absolute (count) data to calibrate the base case against to obtain robust results for the scenarios.

The number of symptomatic cases with influenza was derived from the inferred number of infections using the epidemiological model.^21^ We used the influenza (sub-)type specific proportions of infected cases with ILI symptoms reported in Carrat et al. (2008).^12^

For the GP visits due to influenza, we used the number of surveillance count data stratified by age (PT, ES, IE, FR, SC) and non-age-stratified (NV); and the number of GP visits based on the previously estimated probability given infection (EN, NL).^22,23^ For Navarra, we stratified the annual surveillance count data using the age-stratified data from Spain.^24^ For Ireland, in the absence of suitable and published sources on GP consultations we used the age-stratified Influenzanet data published for the Netherlands.^13^ Another study of the Influenzanet data comparing the non-age-stratified proportions of GP visits between eight countries showed that – while Ireland was not included – the confidence intervals of the non-age-stratified GP visits in 2012-2013 in the Netherlands and the UK were around 30% and overlapped.^25^ If one accepts that the UK is a more adequate proxy for Ireland than e.g. Portugal and Spain, with self-reported GP visits of nearly 50% according to Paolotti et al.,^25^ this suggests that the Netherlands may be the more sensible setting to use for GP visits in Ireland. For Scotland, we back-calculated the aggregated weekly rate of GP visits per 100,000 between 2010-2011 and 2016-2017 to absolute numbers using population estimates. In the absence of age-stratified data, we used the age-stratified proportion of calls to the medical advice helpline in Ireland. This decision was based on the fact that the data from the Netherlands reported the proportion per age group (i.e., not adding up to 100% when looking across age groups), and Ireland being presumed to be more closely related to Scotland than Portugal or Spain. However, we do acknowledge that this stratification potentially introduces bias in the analysis, which is somewhat captured with the wide range of values in the probabilistic sensitivity analyses.

For the hospitalisations due to influenza, we used the number of surveillance count data stratified by age (PT, ES, NV, IE); age-stratified administrative count data (EN, FR, SC); and the number of hospitalisations based on the previously estimated probability of hospitalisation given infection (EN, NL).^22,23^ For Spain, the numbers represented severe hospitalized confirmed influenza cases.^24^ Where necessary, we reformatted age groups according to the age groups of the epidemiological model. We then used the mean proportion across seasons, conditioned on the number of infections in the base case scenario.

In terms of the differences seen between Spain and Navarra, a region within Spain, the main differences appear to arise from: 1) a pattern of higher incidence in Northern Spain; 2) the surveillance in Navarra covers all hospitalizations while national surveillance covers only severe cases in a sample of hospitals from which the total number of hospitalization is estimated; 3) in Navarra consultations are reported electronically from clinical records, while in other regions of Spain they require GP participation.

For premature deaths related to influenza, the available data differed widely by looking into in-hospital mortality due to influenza or excess mortality due to influenza. Therefore, we have opted to harmonise the methodology and data sources by using the detailed estimates of excess mortality in Appendix Table 6 in Iuliano et al. (2018) for all settings.^26^ The authors provided the estimates as median and 95%-credible interval, from which we recovered the mean values and the variance.^27^ For Navarra, we used the estimates for Spain, while for Scotland we used the estimates for the “UK (England and Wales)”. The estimates of Portugal, Spain, the Netherlands, and the UK represented the mean annual influenza-associated respiratory excess mortality rates, while the estimates for France and Ireland were derived from a Bayesian Hierarchical model that extrapolated the influenza-related excess mortality from other countries. Although excess mortality for France and Ireland have been estimated to be higher than reported by Iuliano et al., our analysis used the published estimates of Iuliano et al. for reasons of consistency and standardising data sources. However, we used the estimates supplied by France and Ireland to attribute deaths in patients below the age of 65, which are reported by Iuliano et al. only as a composite age group (see next paragraph). Using higher mortality estimates would lead to more favourable cost-effectiveness ratios by preventing more deaths.

The excess mortality data were published by Iuliano et al. for the three age groups of [0,65), [65,75), and [75,+);^26^ in order to separate the deaths attributable to the different age groups below the age of 65 years that we used in our analysis, we used any additional information available per setting (i.e., using age-stratified in-hospital deaths for Spain,^24^ Navarra,^24^ and the Netherlands,^23^; and using the age-stratified excess deaths for Portugal,^28^ France [I-MOVE+ partner], Ireland [I-MOVE+ partner], England,^29^ and Scotland [I-MOVE+ partner]). The impact of this age-stratification (below the age of 65 years) on the results is marginal given that the vast majority of deaths occur in the elderly aged 65+ years.

### Influenza-related QALYs

The primary outcome measure used for the economic evaluation was the quality-adjusted life year (QALY) given that the preferred type of economic evaluation for vaccines aiming at seasonal influenza virus infections is a cost-utility analysis (CUA) capturing the impact on the quality of life of patients as well as the potential reduction in life years lived due to premature mortality. We estimated the impact of the different vaccination scenarios based on the QALY gain from vaccination (i.e., the averted QALY loss due to influenza), and vaccinating the target populations reduced the incidence of infections, which is reflected in lower numbers of symptomatic ILI cases, healthcare resource use, premature mortality, and QALYs lost.

We considered three categories for QALYs related to influenza in this analysis: non-fatal and non-hospitalised cases, non-fatal and hospitalised cases, and fatal cases.

For the QALYs lost per hospitalised and non-hospitalised case in Spain and Navarra, we used the study of Hollmann et al. (2013) on influenza A(H1N1)pdm09 patients in Spain.^30^ Baguelin et al. (2013) report on QALY losses for non-hospitalised and hospitalised influenza cases in the UK,^1^ which we assumed to be transferable to England, Scotland, and Ireland. For the Netherlands, we used the number of QALYs lost per person per non-fatal non-hospitalised episode from adults with lower respiratory tract infection,^31^ and the number of QALYs lost per person per non-fatal hospitalised episode from the elderly with hospitalized community acquired pneumonia.^32^

We estimated the QALYs lost per hospitalised and non-hospitalised case in Portugal and France based on EQ-5D scores for Spain,^30^ and the EQ-5D value tariffs for Portugal and France.^33,34^ Different populations internationally are generally valuing the health-related quality of life of diseases differently due to cultural and other (partly unknown) reasons,^35^ and the population in Portugal generally appears to be valuing health state utilities lower than the population in Spain.^36^ Moreover, EQ-5D value tariffs were readily available for Portugal and France (but e.g. not for Ireland). We thus derived estimates of the QALYs lost by mapping the health state utility values of the national tariffs of Portugal^33^ and France^34^ to the EQ-5D information of the survey from Spain.^30^ In particular, the study of Hollmann et al. (2013) included 432 inpatients and 563 outpatients whose proportions of answers on all three levels of the five descriptive dimensions of the EQ-5D we used to sample the underlying health states.^30^ We then mapped the utility values of the tariffs from Portugal and France to the health states. We accounted for the fact that we sampled all five dimensions of the EQ-5D independently from each other but only depending on their reported relative frequency,^30^ which is likely to introduce bias, by also mapping the utility values of the Spanish tariff ^37^ and adjusting the values by the difference of the sampled health states with the empirically reported health states. We then used these values specific to Portugal and France in the cost-effectiveness analysis to estimate QALY losses for non-fatal cases using an assumed duration of 7 days of illness for non-hospitalised patients and a mean hospital length of stay of 10 days. All QALY losses from illness episodes were not discounted as they occurred within one year.

For the QALYs lost from premature mortality due to influenza, we used the age- and sex-specific life expectancies for each country as published by the World Health Organization (WHO) as 3-yearly average for 2014-2016. For Scotland and Navarra, we used local data published for the same period by the respective national institutes of statistics (ONS and INE, respectively). We then multiplied the age- and sex-specific life expectancies with the age- and sex-specific utility norms of the general population for the remaining life time had the patients not died prematurely of influenza.^36,38^ Given that the QALY losses from premature mortality occur over more than one year, we discounted QALYs after the first year at 3.0% according to WHO guidance.^19^

| Supplementary Table 3: Influenza-related QALY losses by setting | | | | | | | | |
| --- | --- | --- | --- | --- | --- | --- | --- | --- |
| category | **PT** | **ES** | **NV** | **FR** | **NL** | **EN** | **SC** | **IE** |
| QALY inpatient | 0.0140^a^ | 0.0310 | 0.0310 | 0.0112^a^ | 0.0118 | 0.0170 | 0.0170 | 0.0170 |
| QALY outpatient | 0.0089^a^ | 0.0090 | 0.0090 | 0.0068^a^ | 0.0045 | 0.0078 | 0.0078 | 0.0078 |
|  |  |  |  |  |  |  |  |  |
| life expectancy (LE), in years |  |  |  |  |  |  |  |  |
| 0-4 | 80.9 | 82.5 | 83.3 | 82.3 | 81.2 | 79.6 | 77.3 | 81.0 |
| 5-14 | 74.0 | 75.7 | 76.5 | 75.5 | 74.4 | 72.2 | 70.0 | 74.2 |
| 15-64 | 44.9 | 46.3 | 47.0 | 46.4 | 45.0 | 43.1 | 41.2 | 45.0 |
| 65+ | 12.7 | 13.5 | 13.8 | 14.1 | 12.5 | 8.9 | 8.3 | 12.4 |
| QALE, undiscounted, in years |  |  |  |  |  |  |  |  |
| 0-4 | 70.9 | 72.0 | 72.6 | 71.9 | 71.1 | 69.9 | 68.3 | 71.0 |
| 5-14 | 65.8 | 67.1 | 67.7 | 66.9 | 66.1 | 64.4 | 62.7 | 66.0 |
| 15-64 | 41.8 | 43.1 | 43.7 | 43.1 | 42.0 | 40.3 | 38.6 | 42.0 |
| 65+ | 12.1 | 12.9 | 13.2 | 13.5 | 12.0 | 8.5 | 7.9 | 11.8 |
| QALE, discounted (3.0%), in years |  |  |  |  |  |  |  |  |
| 0-4 | 27.1 | 27.2 | 27.2 | 27.2 | 27.1 | 27.0 | 26.9 | 27.1 |
| 5-14 | 26.7 | 26.8 | 26.8 | 26.8 | 26.7 | 26.5 | 26.4 | 26.7 |
| 15-64 | 22.8 | 23.1 | 23.2 | 23.1 | 22.8 | 22.4 | 21.6 | 22.8 |
| 65+ | 10.1 | 10.6 | 10.8 | 11.0 | 10.0 | 7.5 | 7.1 | 9.9 |
| EN: England, ES: Spain, FR: France, IE: Ireland, LE: life expectancy, NL: Netherlands, NV: Navarra, PT: Portugal, QALE: quality-adjusted life expectancy, QALY: quality-adjusted life year, SC: Scotland.  a: Simulated values by mapping the health state utility values of the national tariffs of Portugal^33^ and France^34^ to the EQ-5D information of the survey from Spain.^30^ The QALY loss is expected to be lower in Portugal than Spain given that the population in Portugal generally appears to be valuing health state utilities lower than the population in Spain.^36,37^ | | | | | | | | |

### Influenza-related healthcare costs

We considered four cost categories related to influenza in this analysis: vaccination (i.e., vaccine plus administration), outpatient/GP visits, and hospitalisations. The base year of costs was 2017, and where necessary we inflated and converted costs to represent 2017 euros (€1 = £0.88).^39,40^

For the costs of vaccination, we considered the vaccine price and the costs of vaccine administration. For the vaccines we used list prices (for Portugal: National Authority of Medicines and Health Products [Autoridade Nacional do Medicamento e Produtos de Saúde], infarmed; France: AMELI; The Netherlands: Foundation for National Influenza Prevention Programme [Stichting Nationaal Programma Grieppreventie], SNPG; and England and Scotland: British National Formulary, BNF) and public tender prices (for Spain and Navarra). Non-public prices for Ireland were supplied by I-MOVE+ partners and are shown only indicatively here. The costs for vaccine administration were also taken from published sources, where available (Spain, Navarra, the Netherlands, England, Scotland), or assuming a GP visit every second year and otherwise opportunistic administration given that our study focussed on vaccinating the elderly and children/adolescents (Portugal, France, Ireland). The same assumption was used in a previous study on vaccine administration.^41^ Administration costs play a role particularly in determining the added value of introducing universal paediatric vaccination to a newly targeted group of individuals.

For the costs per outpatient visit, we obtained unit costs from national data sources for France, The Netherlands, England, and Scotland.^42,43^ For Portugal, Spain, Navarra, and Ireland we were supplied with the costs per GP visit by the I-MOVE+ partners. For Ireland, only the GP consultations paid for by the healthcare provider were considered in the costing, i.e. patients aged <6 and >69 years.^44^ Hence, GP consultations play only a marginal role in our cost-effectiveness analysis for Ireland due to how the healthcare system is organized.

For the costs per hospitalisation, we used the mean costs per hospital admission for respiratory infections or pneumonia across ages (PT, FR, EN, SC), stratified by age (NV, NL, IE), and we used the costs for the elderly for all ages given data availability (ES). For Portugal, we used the mean costs per hospital admission for “respiratory infections and pneumonia”. For Spain, published estimates for hospital stays due to influenza were only available for the elderly.^45^ For Navarra, we received age-stratified mean data on hospital stays due to influenza between 2015/16 and 2017/18, which were multiplied with the mean cost of a hospital bed-day for influenza. For France, we used the mean cost of a hospital admission for influenza. For the Netherlands, the costs per hospital admission were calculated based on the mean length of stay (LOS) of patients admitted with community-acquired pneumonia and the standard costs per bed-day of paediatric, general and ICU wards.^46,47^ We adjusted the costs for different age groups by the time spent in non-ICU wards and ICU wards as supplied by the I-MOVE+ partner. For England, we used the activity-weighted mean costs per hospital admission for the same healthcare resource group (HRG)-code used in Pitman et al., 2013, for "Lobar, Atypical or Viral Pneumonia".^48,49^ For Scotland, we received the average cost of a hospital stay in a respiratory bed.^50^ For Ireland, we received data on the length of hospital stay due to influenza by age, which we multiplied with the mean cost of a hospital bed-day.

| Supplementary Table 4: Influenza-related healthcare costs by setting | | | | | | | | |
| --- | --- | --- | --- | --- | --- | --- | --- | --- |
| category | **PT** | **ES** | **NV** | **FR** | **NL** | **EN** | **SC** | **IE** |
| vaccine admin. | (€28)^a^ | €6 | €6 | (€9.3)^a^ | €11.4 | £12.2 | £12.2 | (€25)^a^ |
| trivalent vaccine | €6.05 | €2.9 | €2.9 | €6.1 | €3.6 | £6.86 | £6.86 | <€10^b^ |
| improved” trivalent vaccine (iTV)^c^ | (€18.8) | (€9.0) | (€9.0) | (€16.8) | (€10.8) | (£14.9) | (£14.9) | (<€15)^b^ |
| quadrivalent vaccine | €12.5 | €6.0 | €6.0 | €11.2 | (€7.2)^d^ | £9.94 | £9.94 | <€10^b^ |
| GP consultations | <€100^b^ | <€150^b^ | <€100^b^ | 18.6 | €84.2 | £37 | £37 | €50^b^ |
| hospitalisations | <€2,500 | €3,263 | <€5,000^e^ | <€2,500 | €3,405^e^ | £1,916 | <£3,000 | <€5,000^e^ |
| willingness-to-pay threshold | (€30,000)^f^ | (€20,000-€25,000)^f^ | (€20,000-€25,000)^f^ | (€30,000)^f^ | €20,000 (€10k-€80k) | £20,000-£30,000 | £20,000-£30,000 | €20,000-€45,000 |
| EN: England, ES: Spain, FR: France, GP: general practitioner, IE: Ireland, iTV: “improved” trivalent vaccine (i.e., adjuvanted or high-dose), NL: Netherlands, NV: Navarra, PT: Portugal, QALY: quality-adjusted life year, QV: quadrivalent vaccine (non-adjuvanted, non-high dose), SC: Scotland, TV: trivalent vaccine (non-adjuvanted, non-high dose).  a: No values for the costs of vaccine administration publicly available; we have assumed the costs of a GP visit every second year and otherwise opportunistic administration given the age groups of elderly and children/adolescents, similar to Ethgen et al. (2016).^41^  b: Prices not public and shown only indicatively here.  c: For the improved trivalent vaccine, we assumed for the base case a vaccine price that was 50% higher than the currently highest price for TV or QV(i.e., max(TV, QV)*1.5). This assumption was explored further in a threshold analysis.  d: No vaccine price available for the quadrivalent vaccine in the Netherlands; we have assumed a price twice as high as the TV based on the price difference observed in the majority of settings.  e: Calculated as age-stratified estimates but shown non-age-stratified here for reasons of sensitivity.  f: Unofficial but frequently cited cost-effectiveness threshold values. | | | | | | | | |

Model fit (aggregated) to the supplied data per setting

Figures of the aggregated model fit for the three influenza virus subtypes.


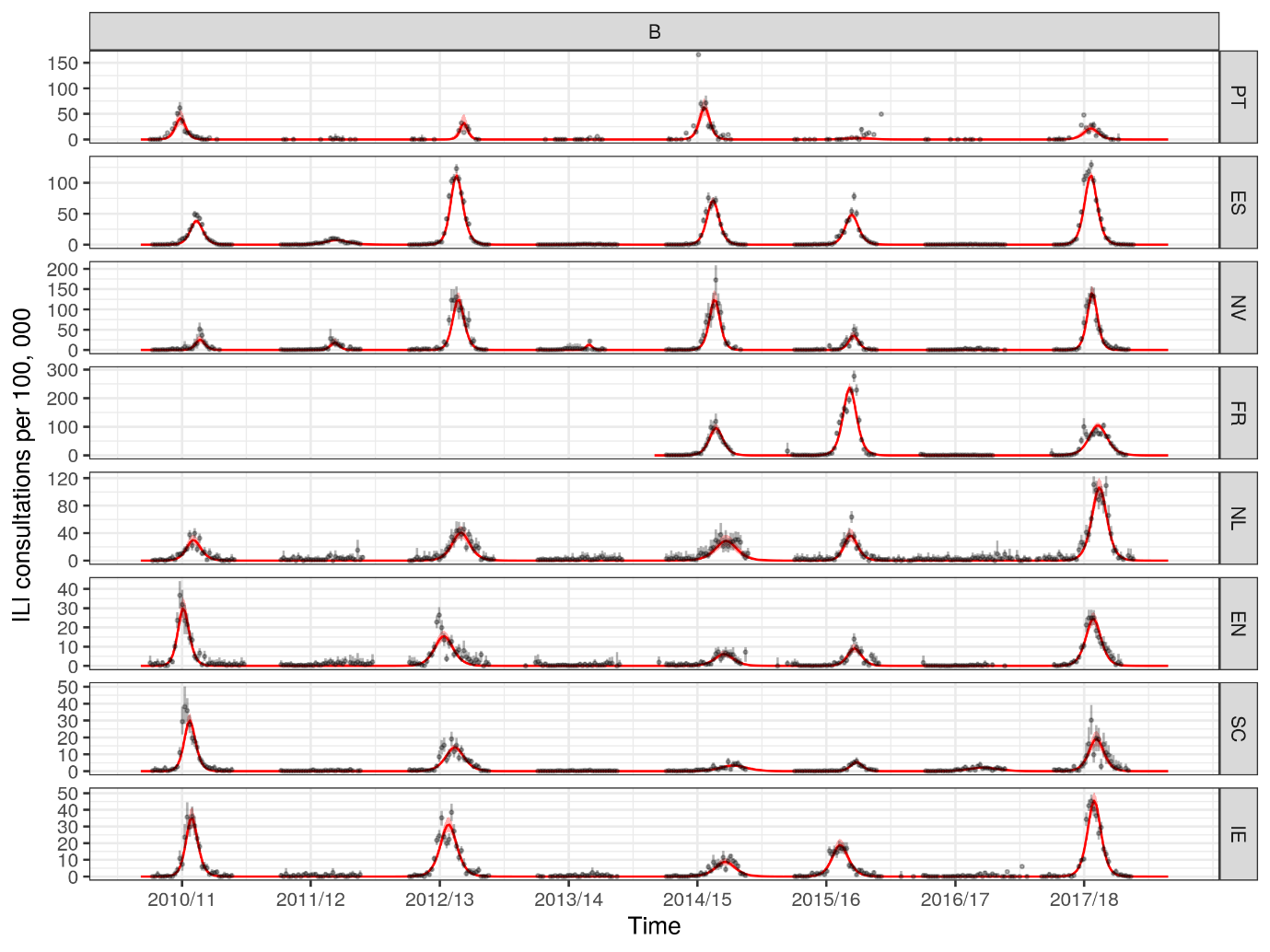
Supplementary Figure 1. Model fit (aggregated) to the supplied data for the settings included in the analysis, influenza B.

EN: England, ES: Spain, FR: France, IE: Ireland, ILI: influenza-like illness, NL: Netherlands, NV: Navarra, PT: Portugal, SC: Scotland.


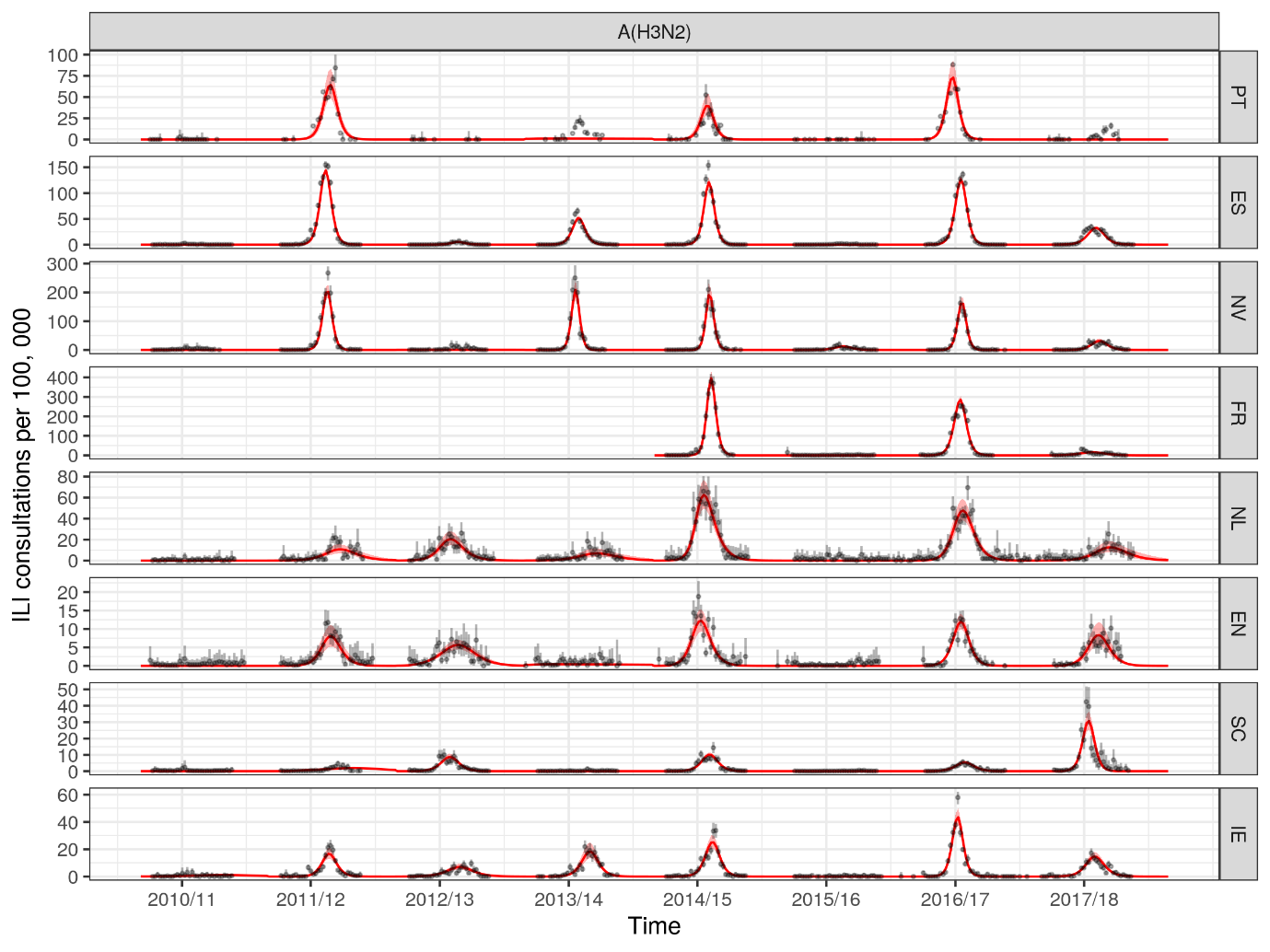
Supplementary Figure 2. Model fit (aggregated) to the supplied data for the settings included in the analysis, influenza A(H3N2).

EN: England, ES: Spain, FR: France, IE: Ireland, ILI: influenza-like illness, NL: Netherlands, NV: Navarra, PT: Portugal, SC: Scotland.


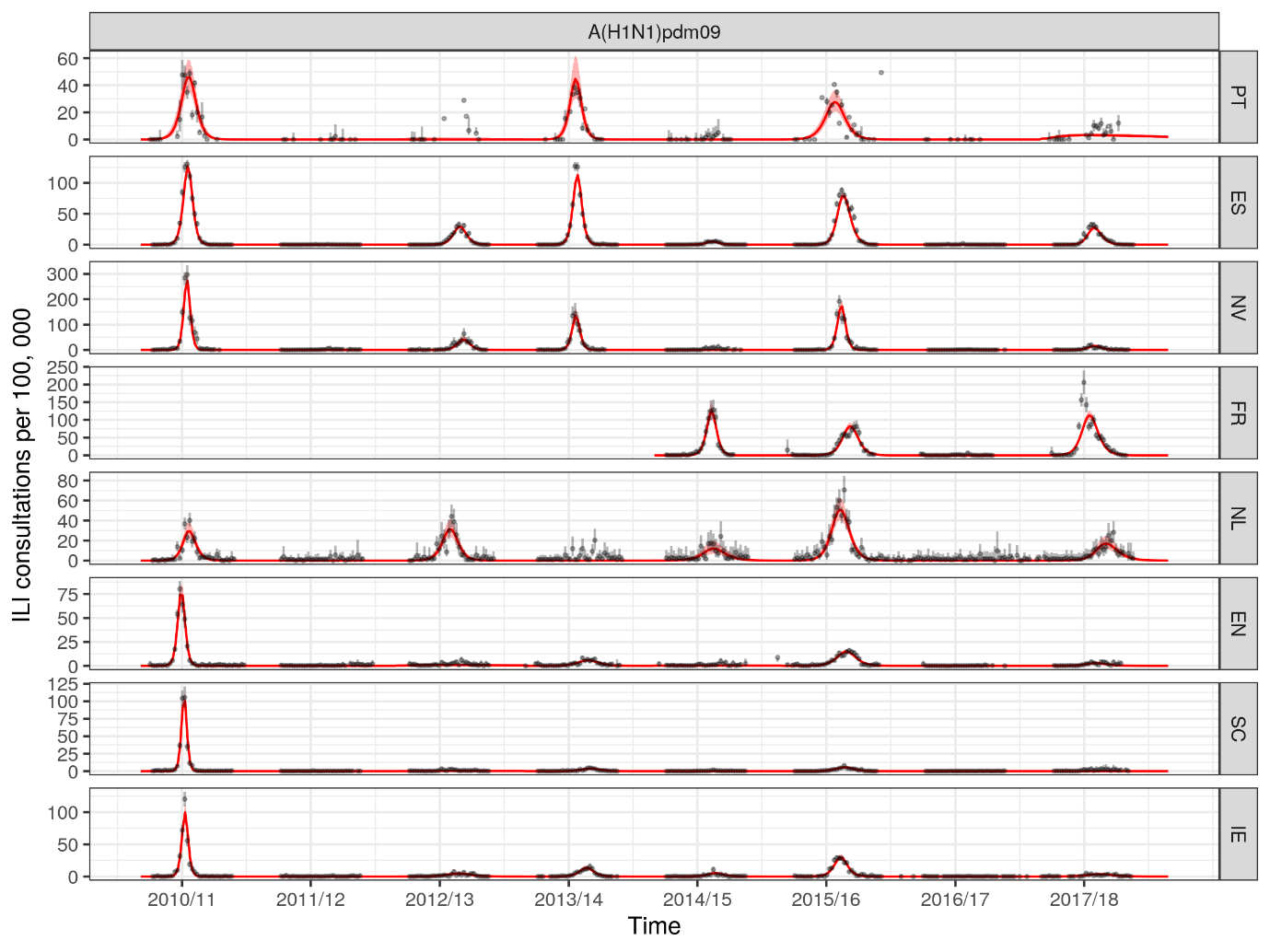
Supplementary Figure 3. Model fit (aggregated) to the supplied data for the settings included in the analysis, influenza A(H1N1)pdm09.

EN: England, ES: Spain, FR: France, IE: Ireland, ILI: influenza-like illness, NL: Netherlands, NV: Navarra, PT: Portugal, SC: Scotland.

We excluded from the further analysis the inference results of ten seasons per strain based on model fit and effective sample sizes (Supplementary Table 5): Influenza A(H1N1) in Portugal (2017/2018) and Ireland (2012/2013); influenza A(H3N2) in France (2015/2016), Ireland (2010/2011 and 2013/2014), Scotland (2011/2012) and England (2017/2018); and influenza type B in Portugal (2015/2016), Spain (2012/2013), and Navarra (2016/2017); results changed only slightly for the settings involved.

Model fit (disaggregated) to the supplied data per setting

Figures of the disaggregated model fit for the three influenza virus subtypes.

Supplementary Figure 4: Posterior parameter values for EN and influenza virus subtype B. Box plots highlight the median, the 50% CI and the 95% CI. Susceptibility and ascertainment rates are assumed to be different for children, adults and elderly.


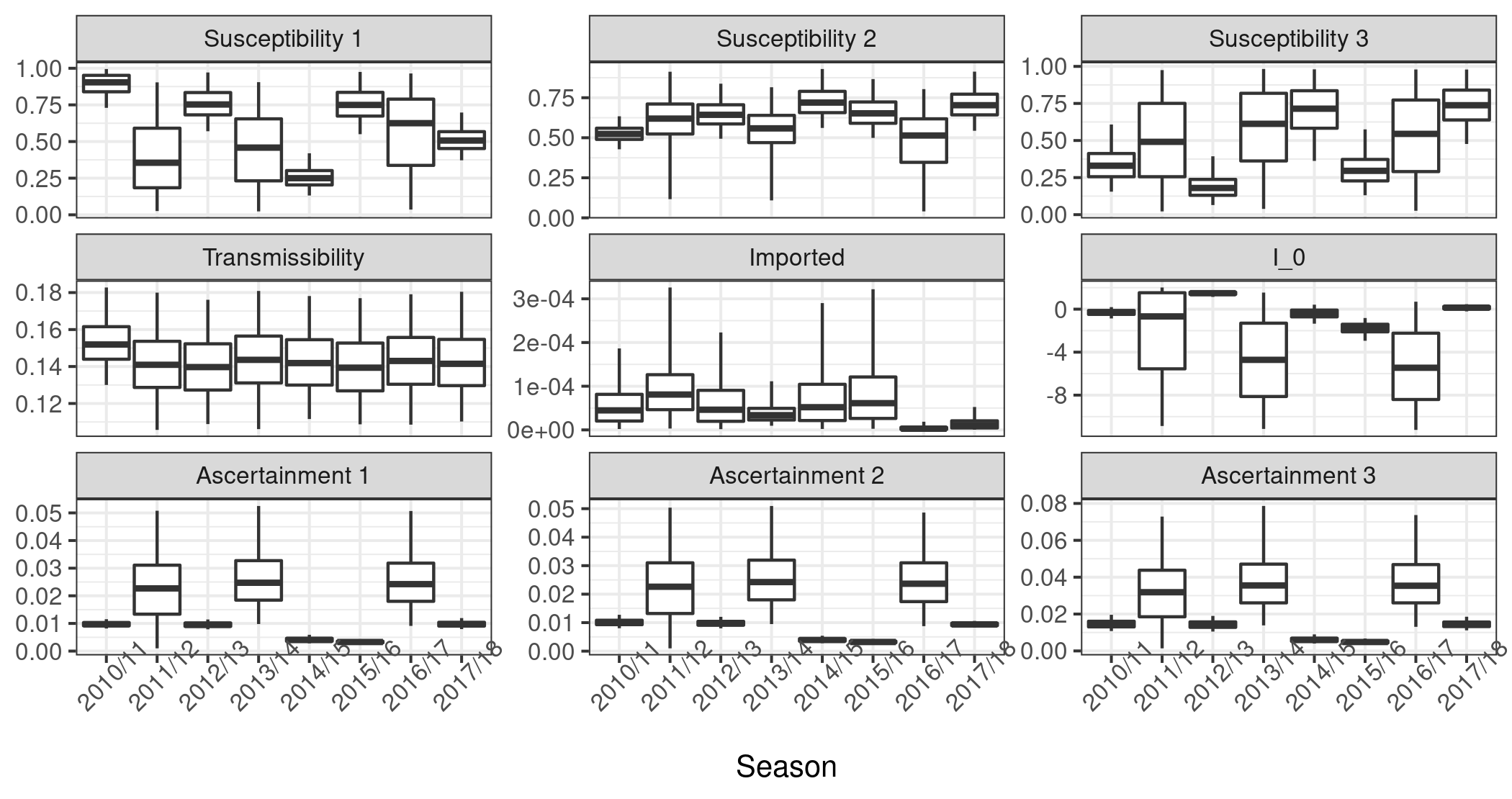


Supplementary Figure 5: Fit of the model to the data for EN and influenza virus subtype B. Red indicates the predicted values, while black shows the data and associated uncertainty (95% CI).


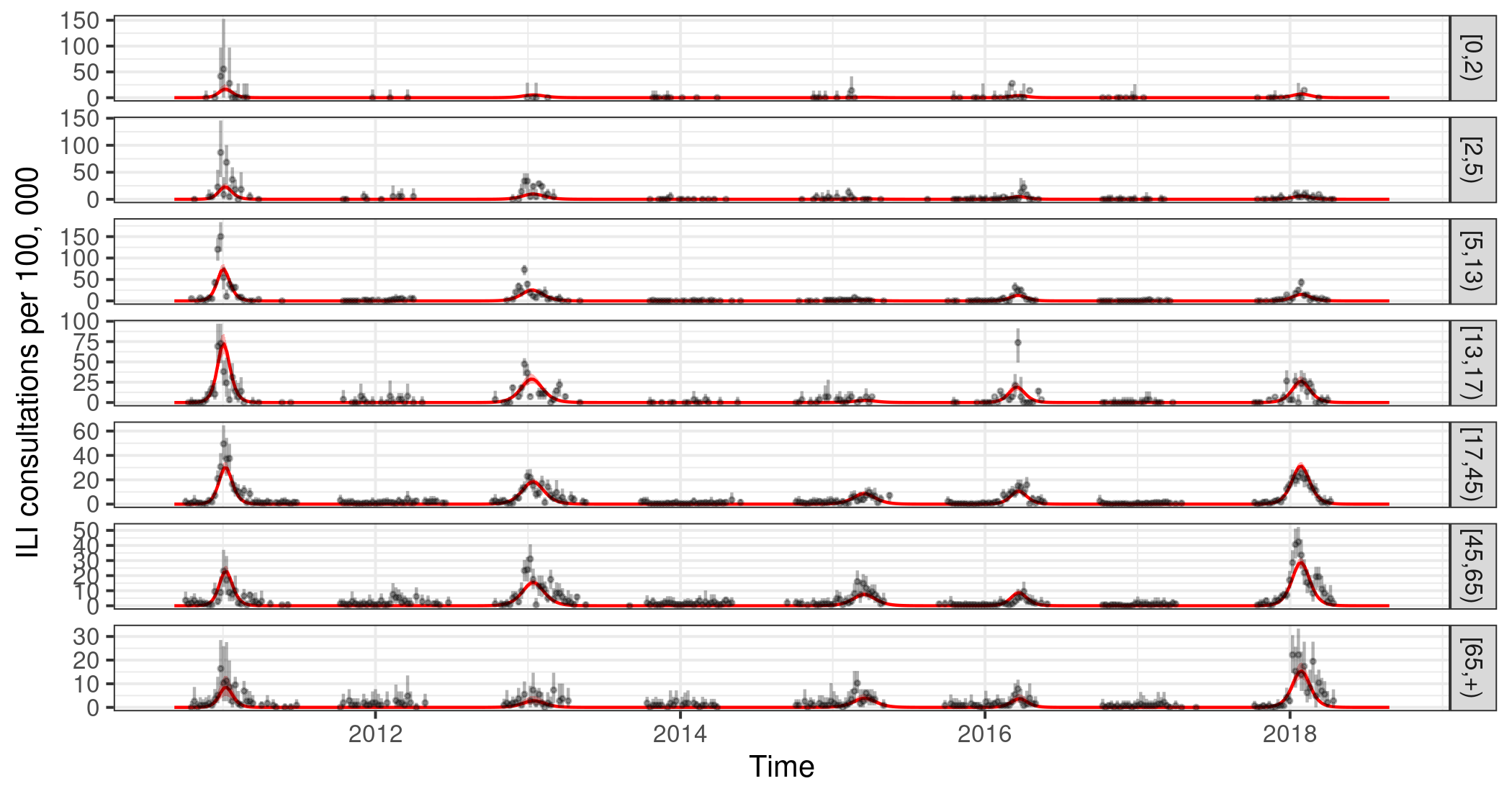


Supplementary Figure 6: Posterior parameter values for EN and influenza virus subtype H1N1. Box plots highlight the median, the 50% CI and the 95% CI. Susceptibility and ascertainment rates are assumed to be different for children, adults and elderly.


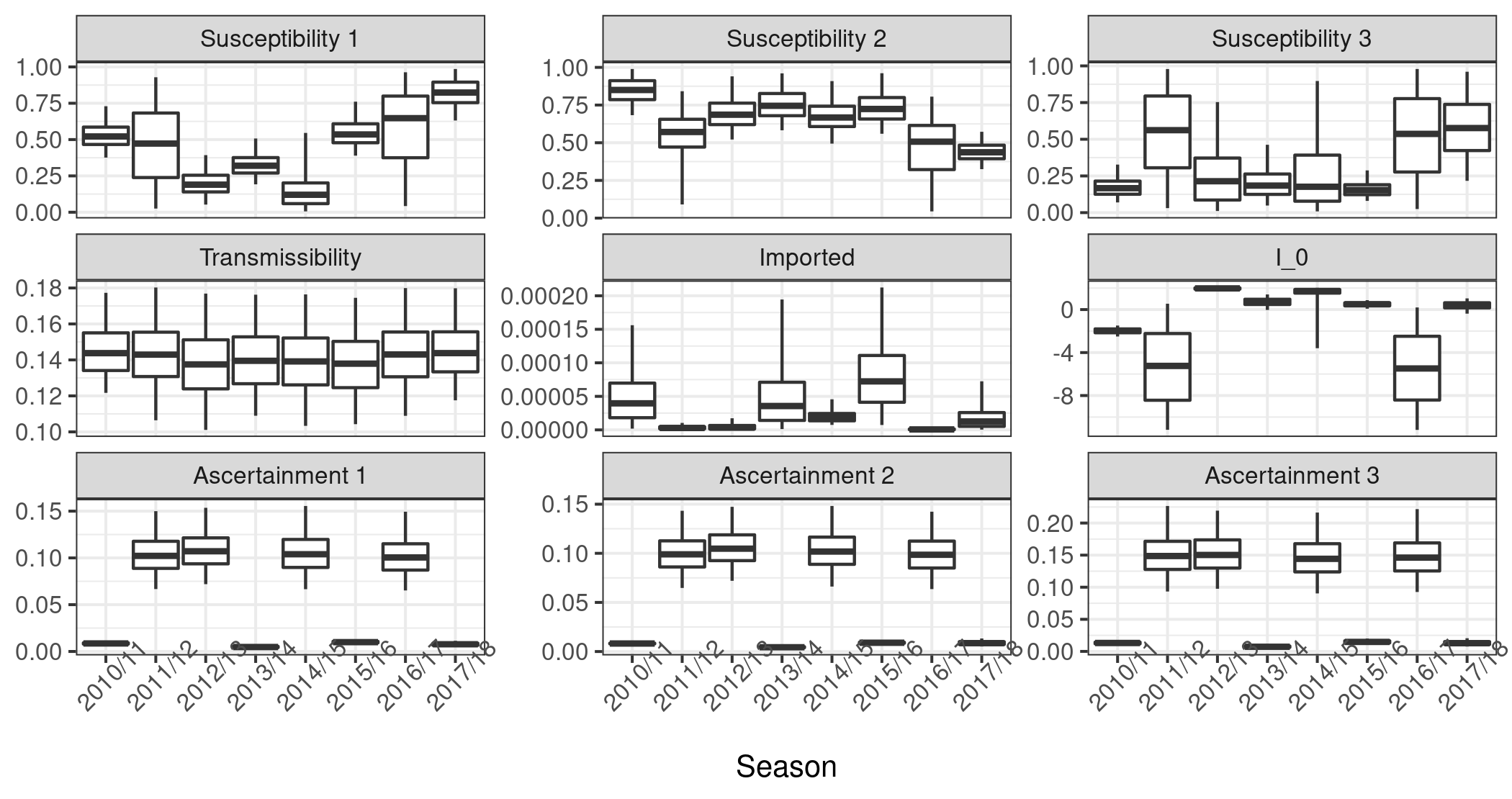


Supplementary Figure 7: Fit of the model to the data for EN and influenza virus subtype H1N1. Red indicates the predicted values, while black shows the data and associated uncertainty (95% CI).


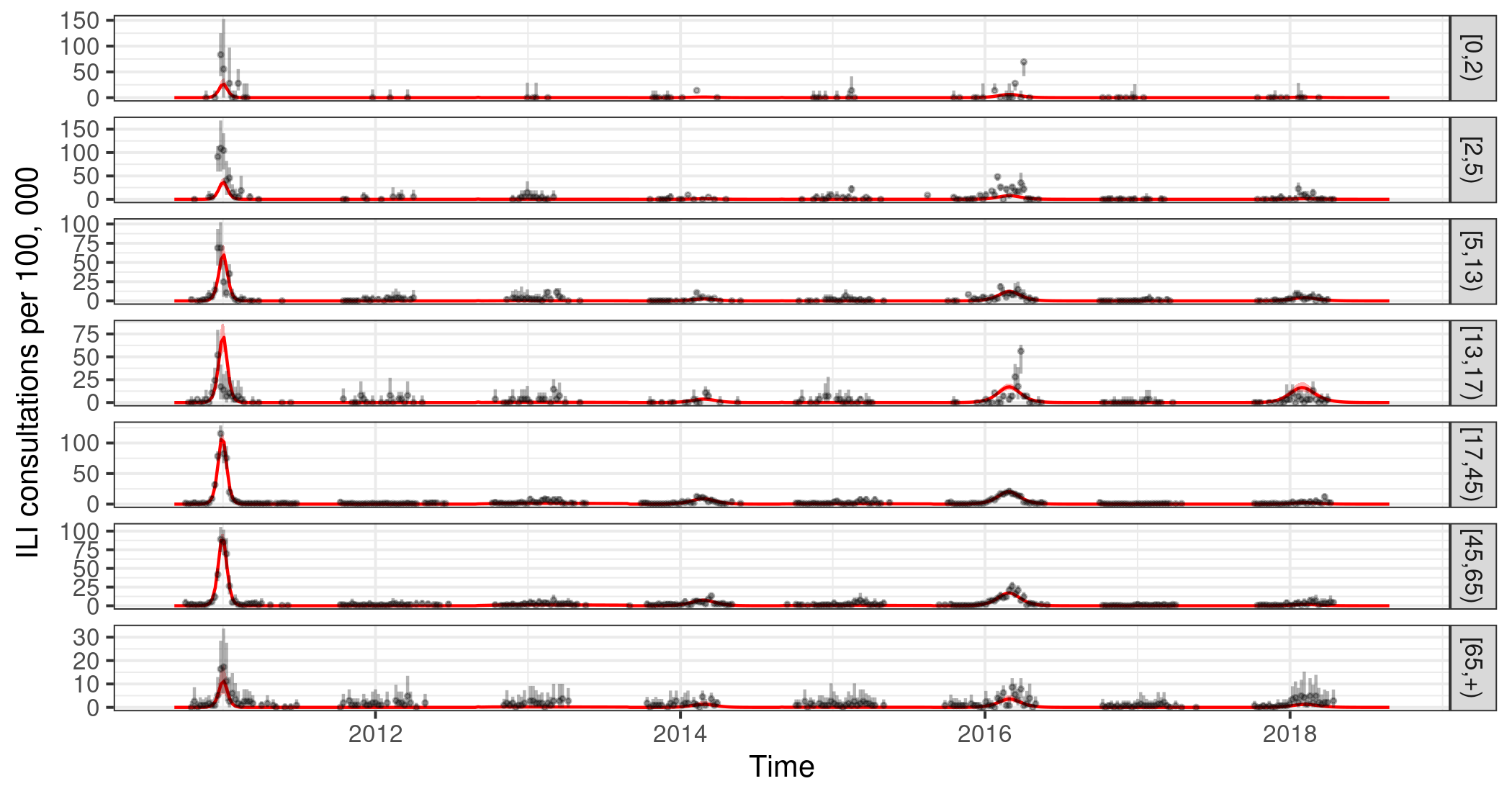


Supplementary Figure 8: Posterior parameter values for EN and influenza virus subtype H3N2. Box plots highlight the median, the 50% CI and the 95% CI. Susceptibility and ascertainment rates are assumed to be different for children, adults and elderly.


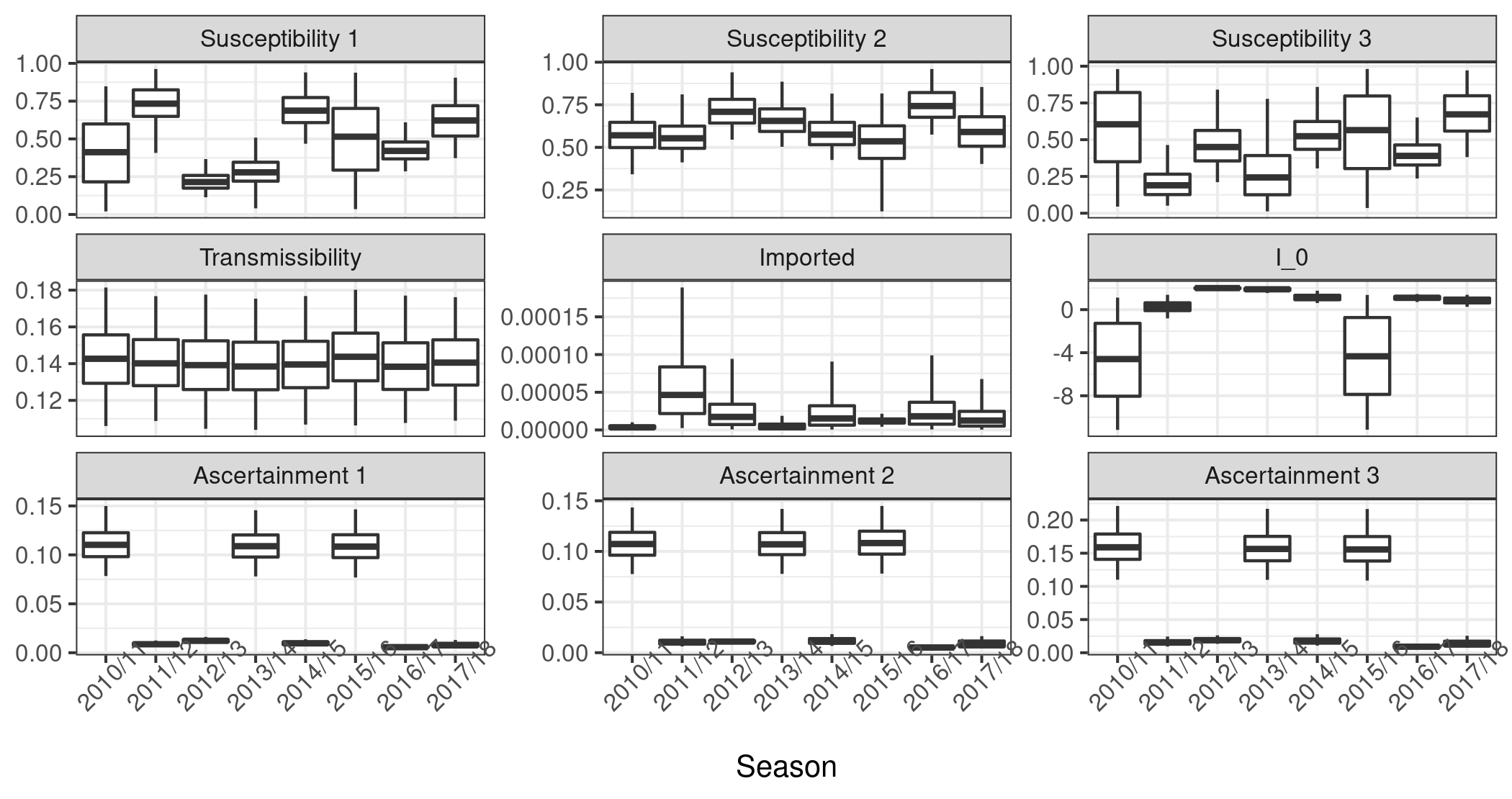


Supplementary Figure 9: Fit of the model to the data for EN and influenza virus subtype H3N2. Red indicates the predicted values, while black shows the data and associated uncertainty (95% CI).


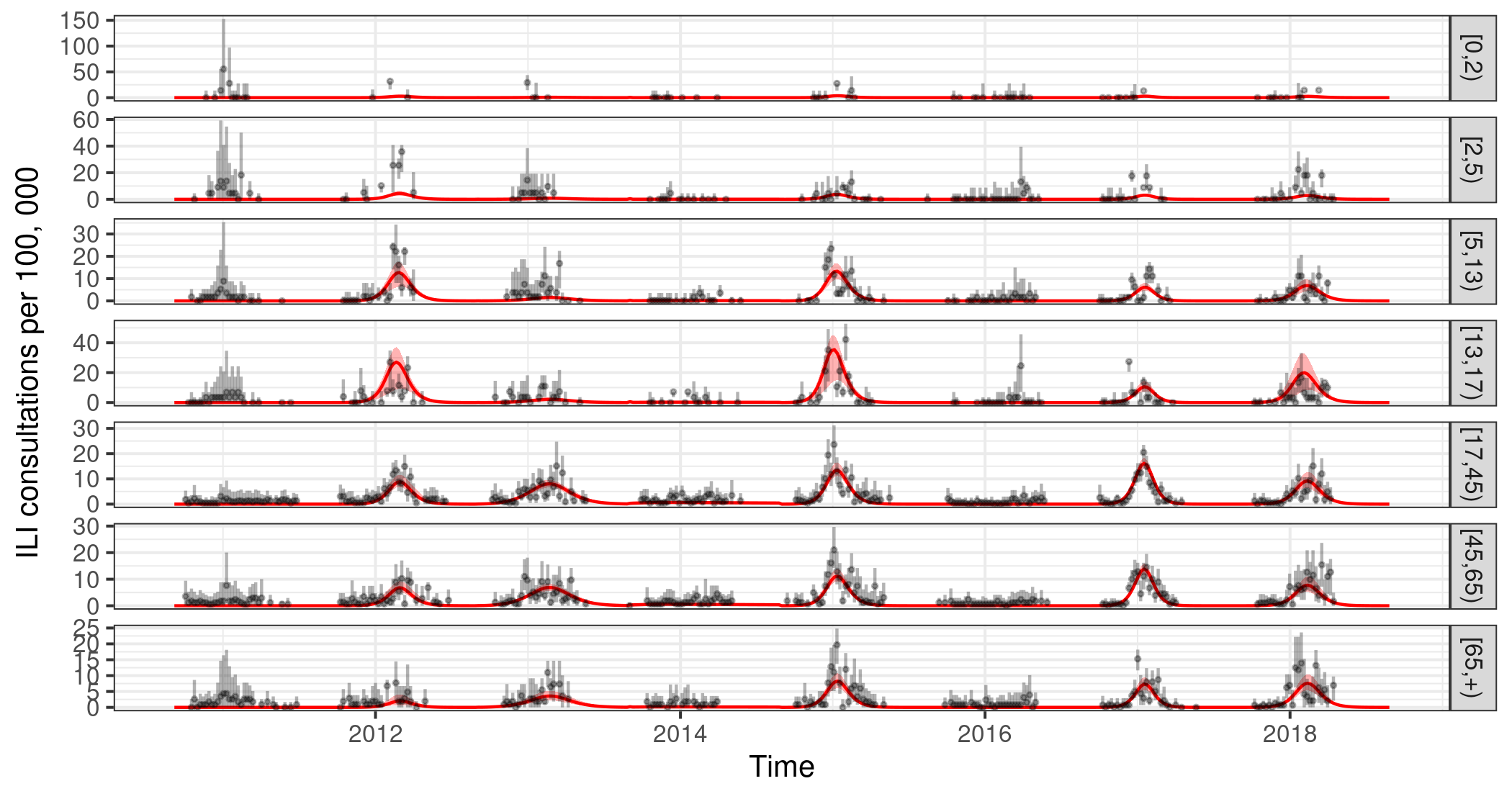


Supplementary Figure 10: Posterior parameter values for ES and influenza virus subtype B. Box plots highlight the median, the 50% CI and the 95% CI. Susceptibility and ascertainment rates are assumed to be different for children, adults and elderly.


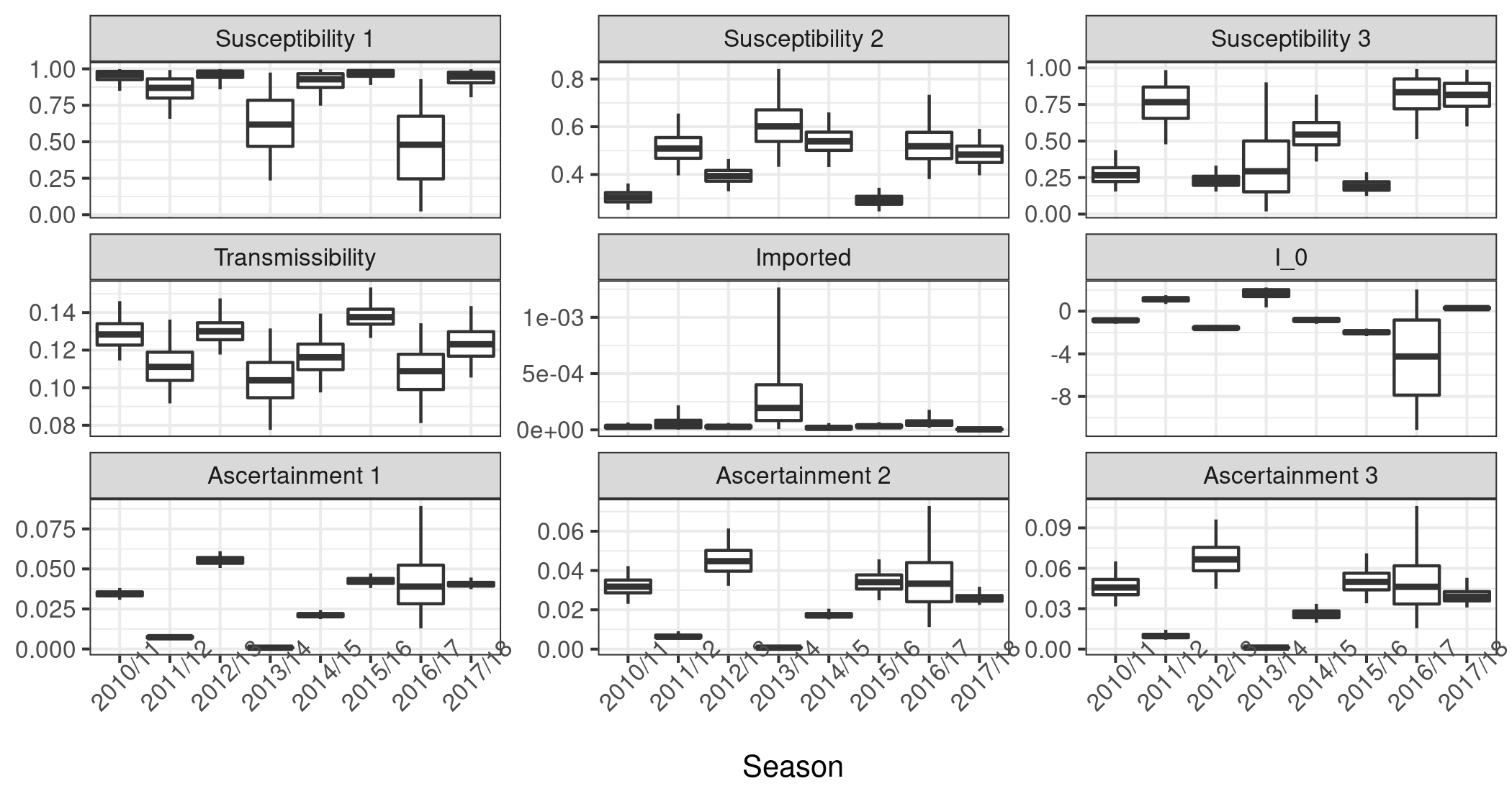


Supplementary Figure 11: Fit of the model to the data for ES and influenza virus subtype B. Red indicates the predicted values, while black shows the data and associated uncertainty (95% CI).


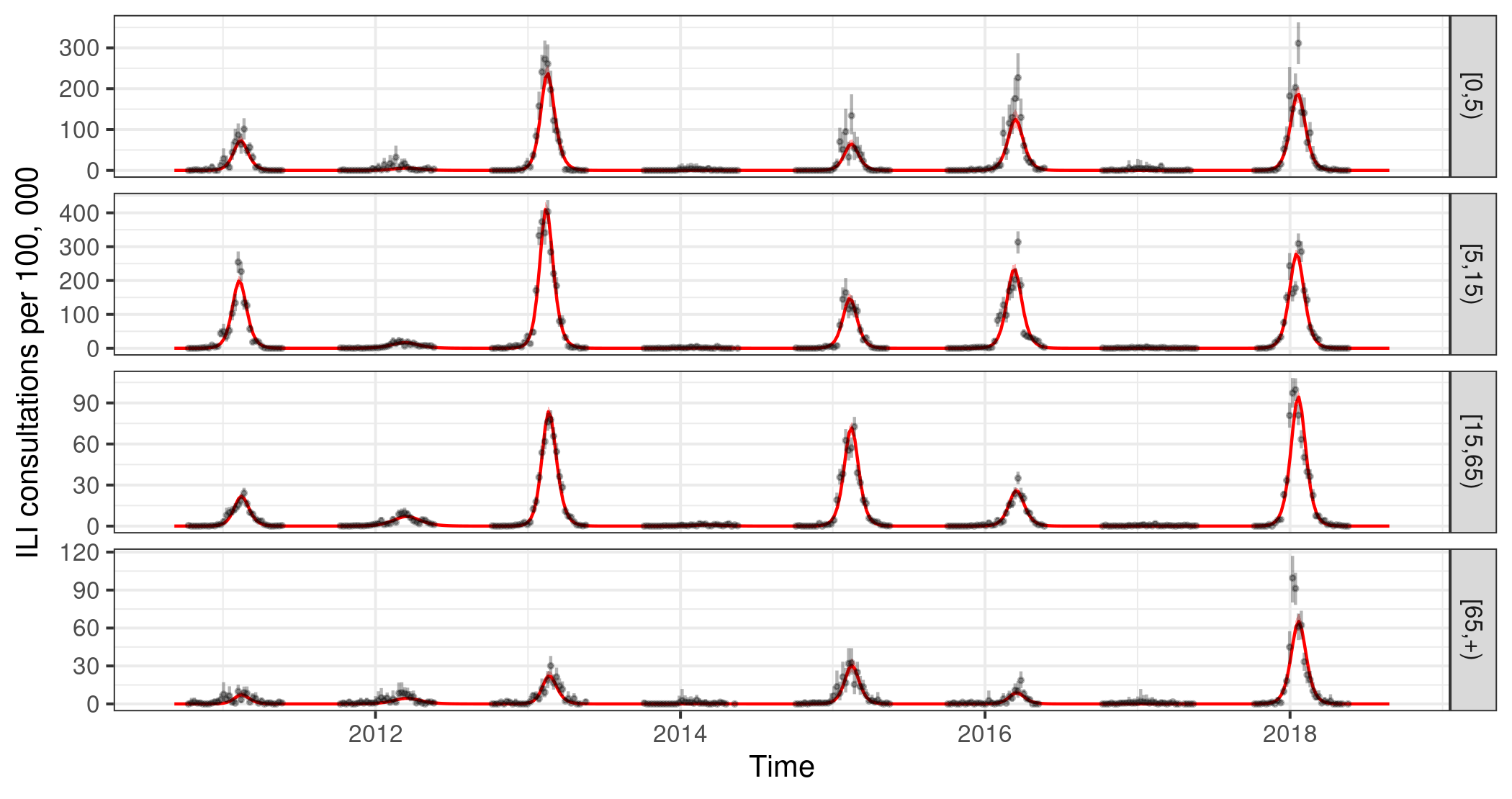


Supplementary Figure 12: Posterior parameter values for ES and influenza virus subtype H1N1. Box plots highlight the median, the 50% CI and the 95% CI. Susceptibility and ascertainment rates are assumed to be different for children, adults and elderly.


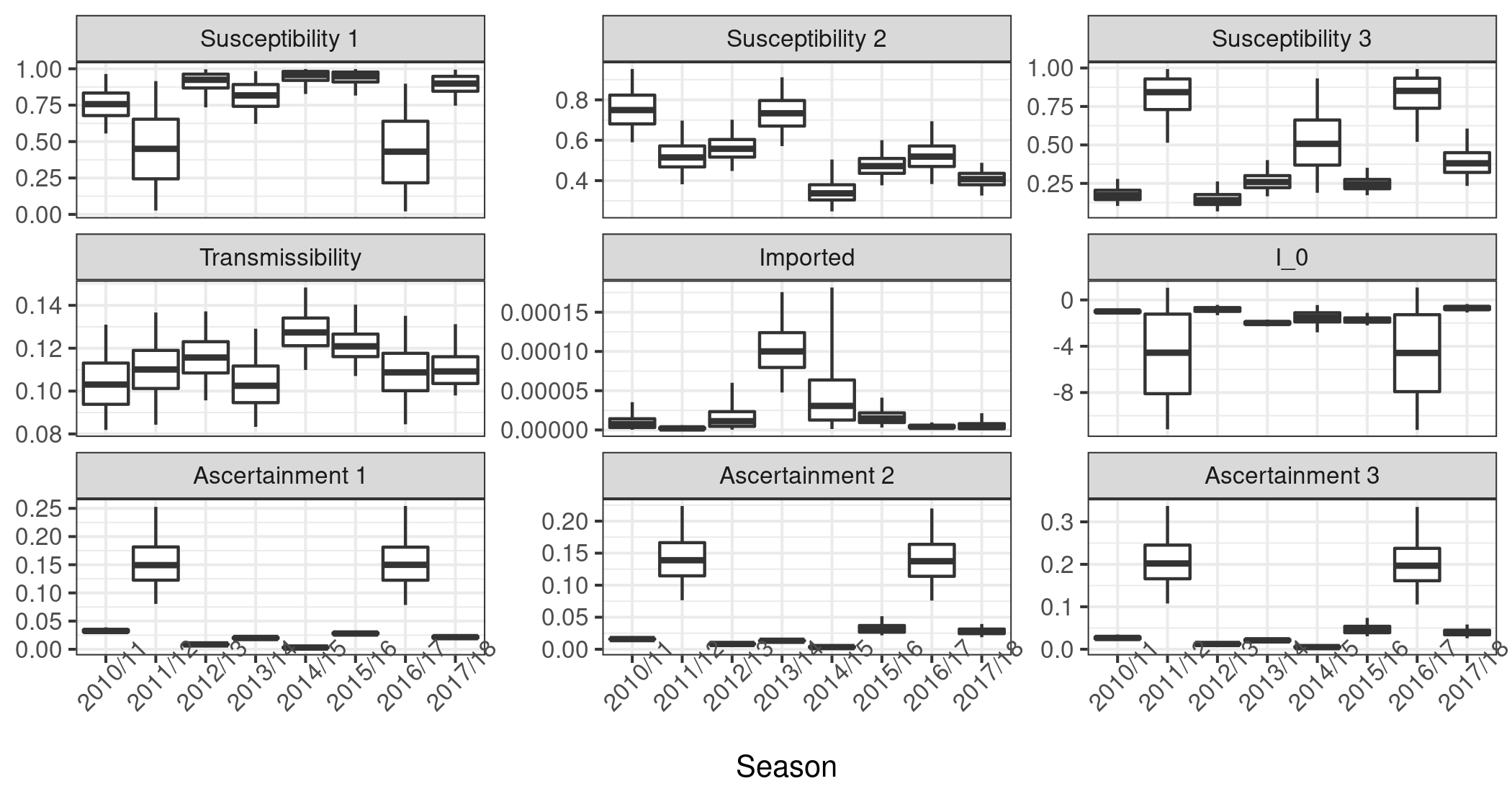


Supplementary Figure 13: Fit of the model to the data for ES and influenza virus subtype H1N1. Red indicates the predicted values, while black shows the data and associated uncertainty (95% CI).


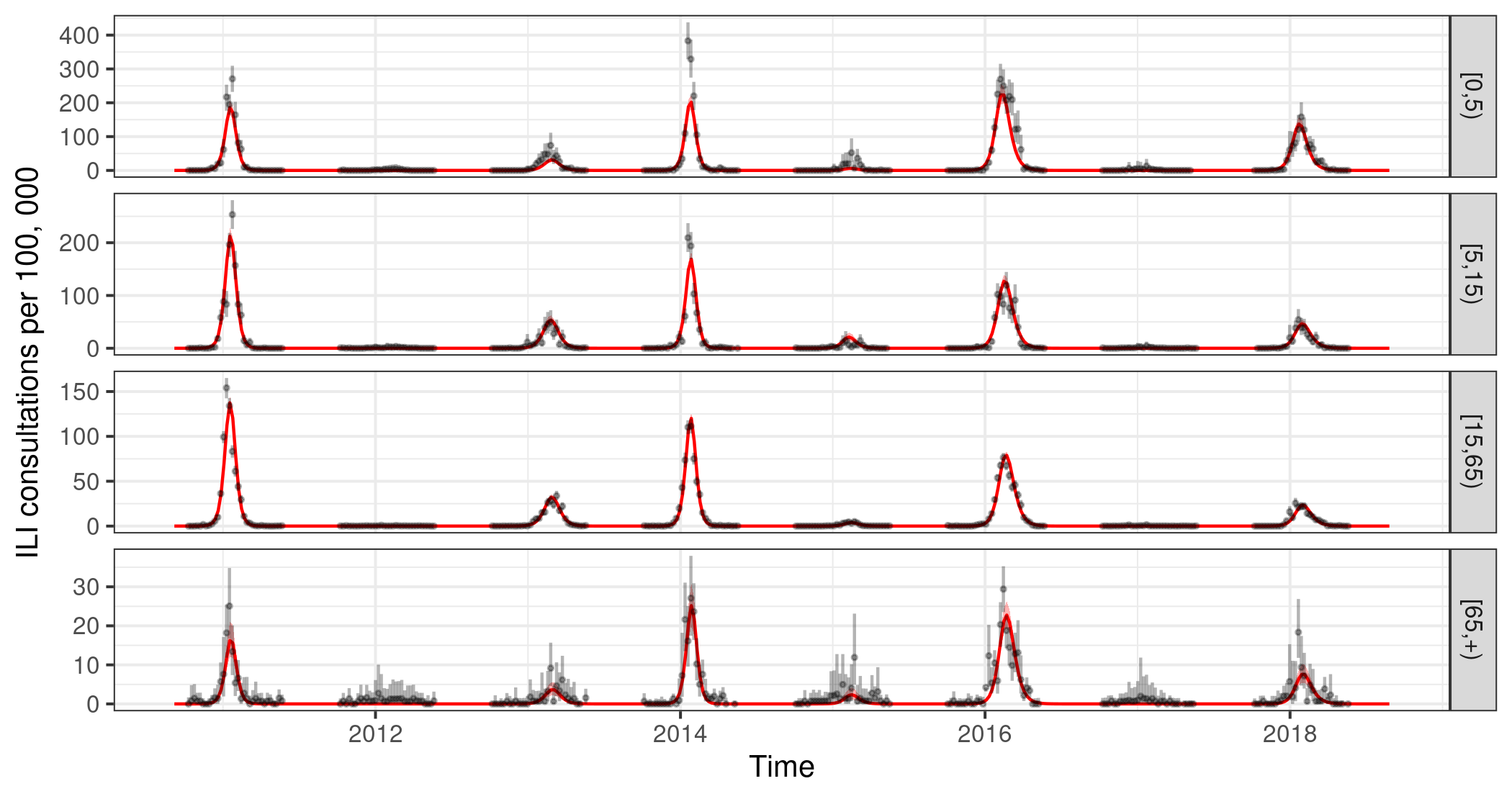


Supplementary Figure 14: Posterior parameter values for ES and influenza virus subtype H3N2. Box plots highlight the median, the 50% CI and the 95% CI. Susceptibility and ascertainment rates are assumed to be different for children, adults and elderly.


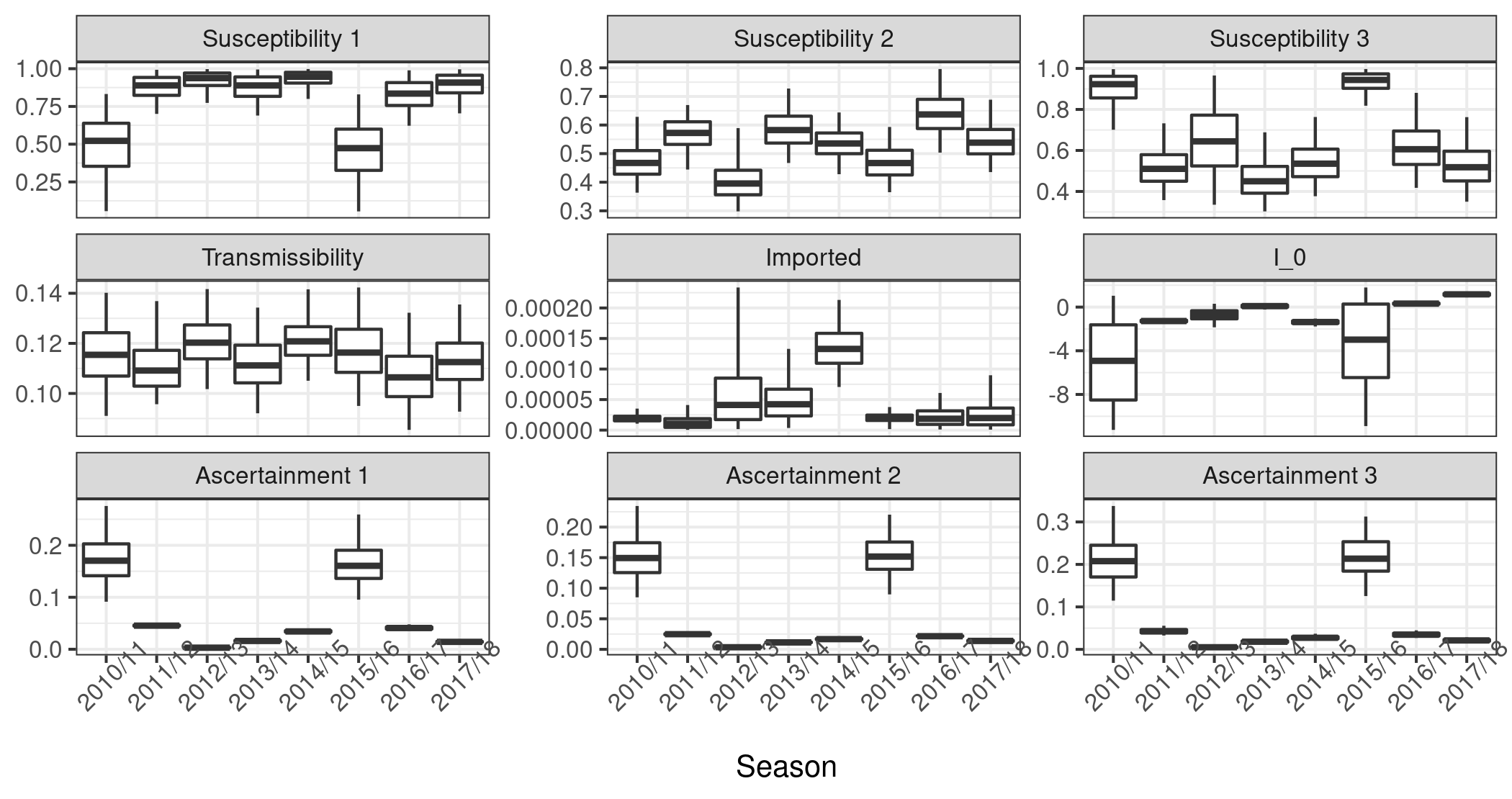


Supplementary Figure 15: Fit of the model to the data for ES and influenza virus subtype H3N2. Red indicates the predicted values, while black shows the data and associated uncertainty (95% CI).


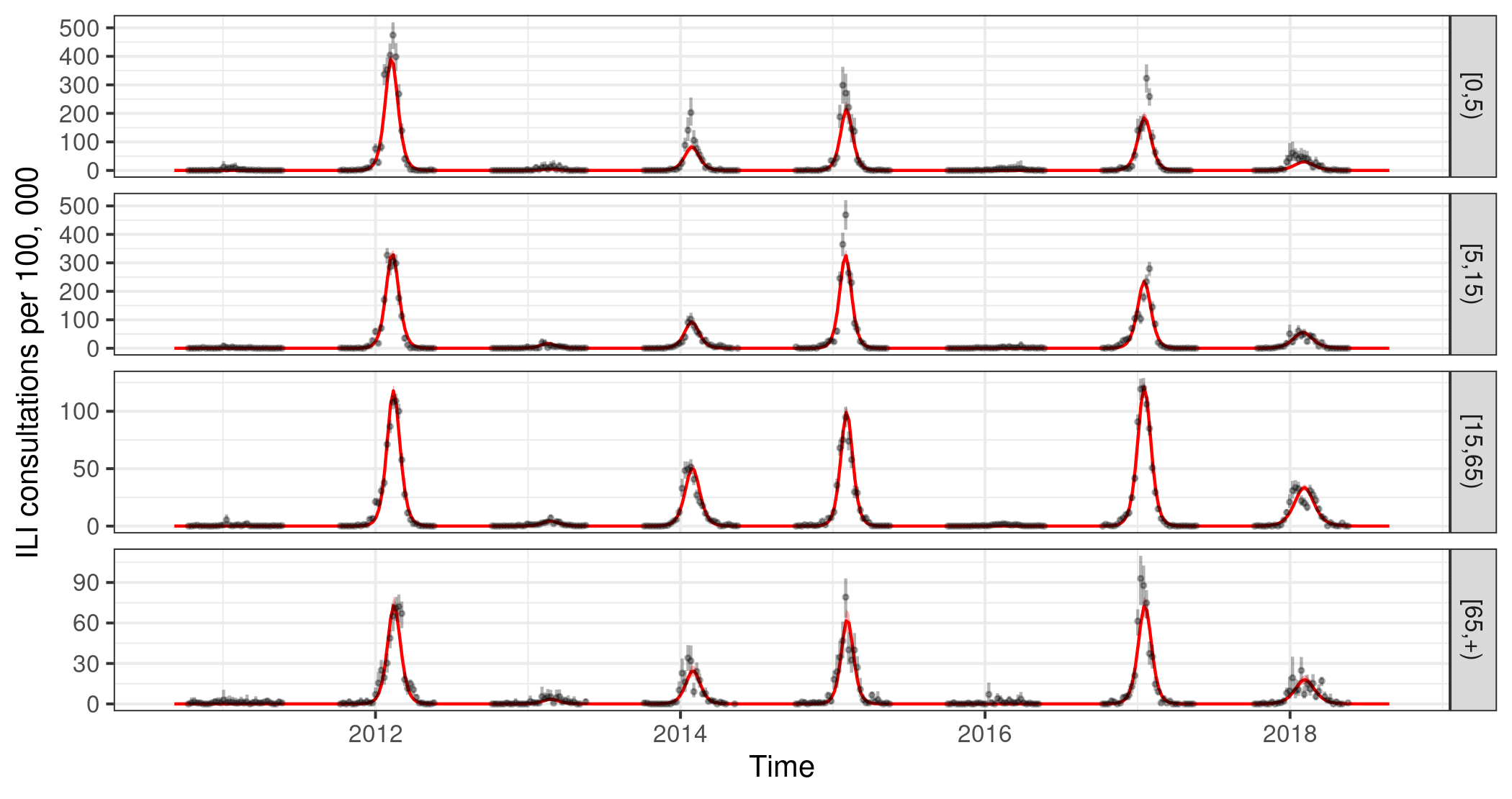


Supplementary Figure 16: Posterior parameter values for FR and influenza virus subtype B. Box plots highlight the median, the 50% CI and the 95% CI. Susceptibility and ascertainment rates are assumed to be different for children, adults and elderly.


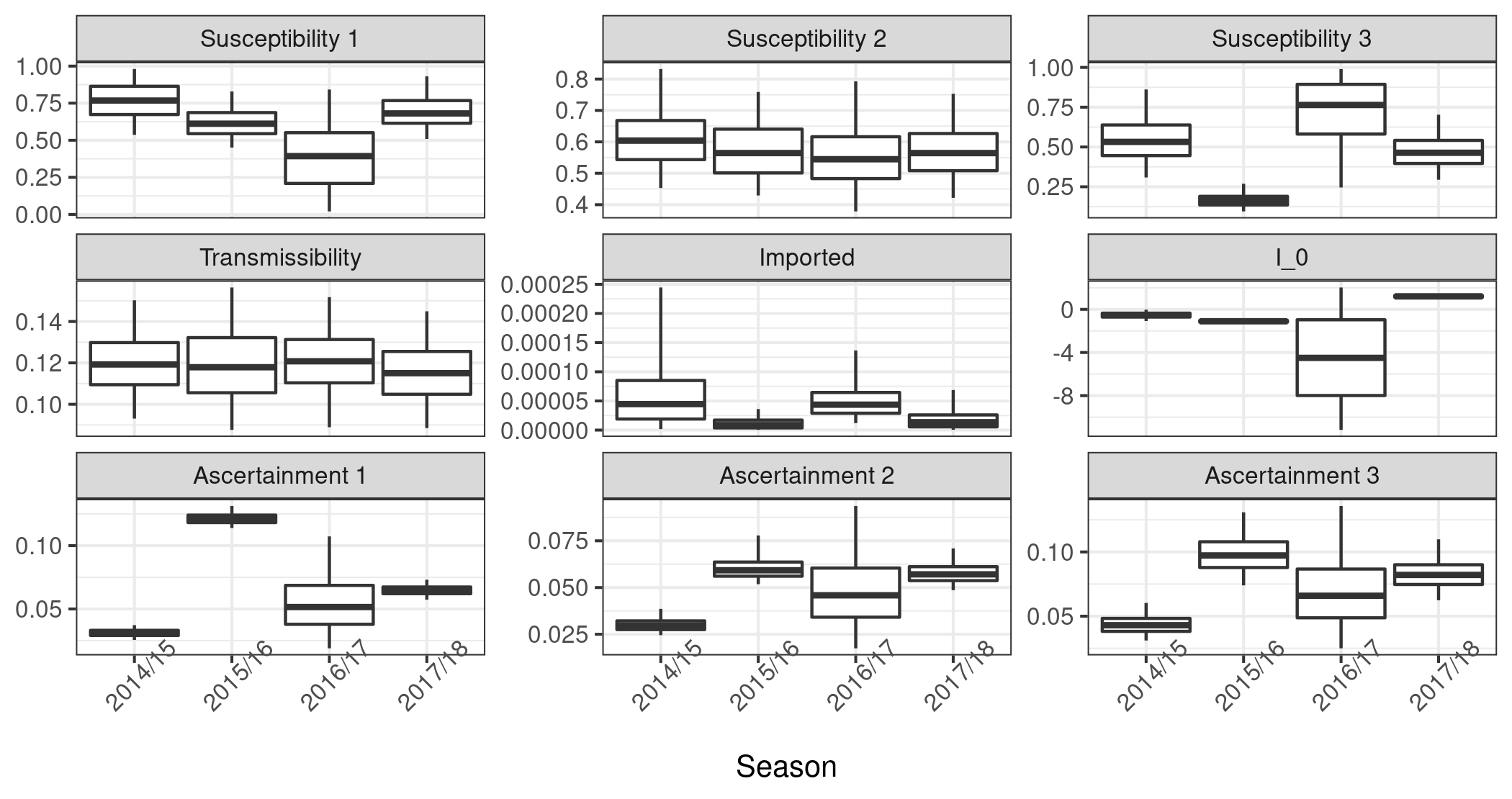


Supplementary Figure 17: Fit of the model to the data for FR and influenza virus subtype B. Red indicates the predicted values, while black shows the data and associated uncertainty (95% CI).


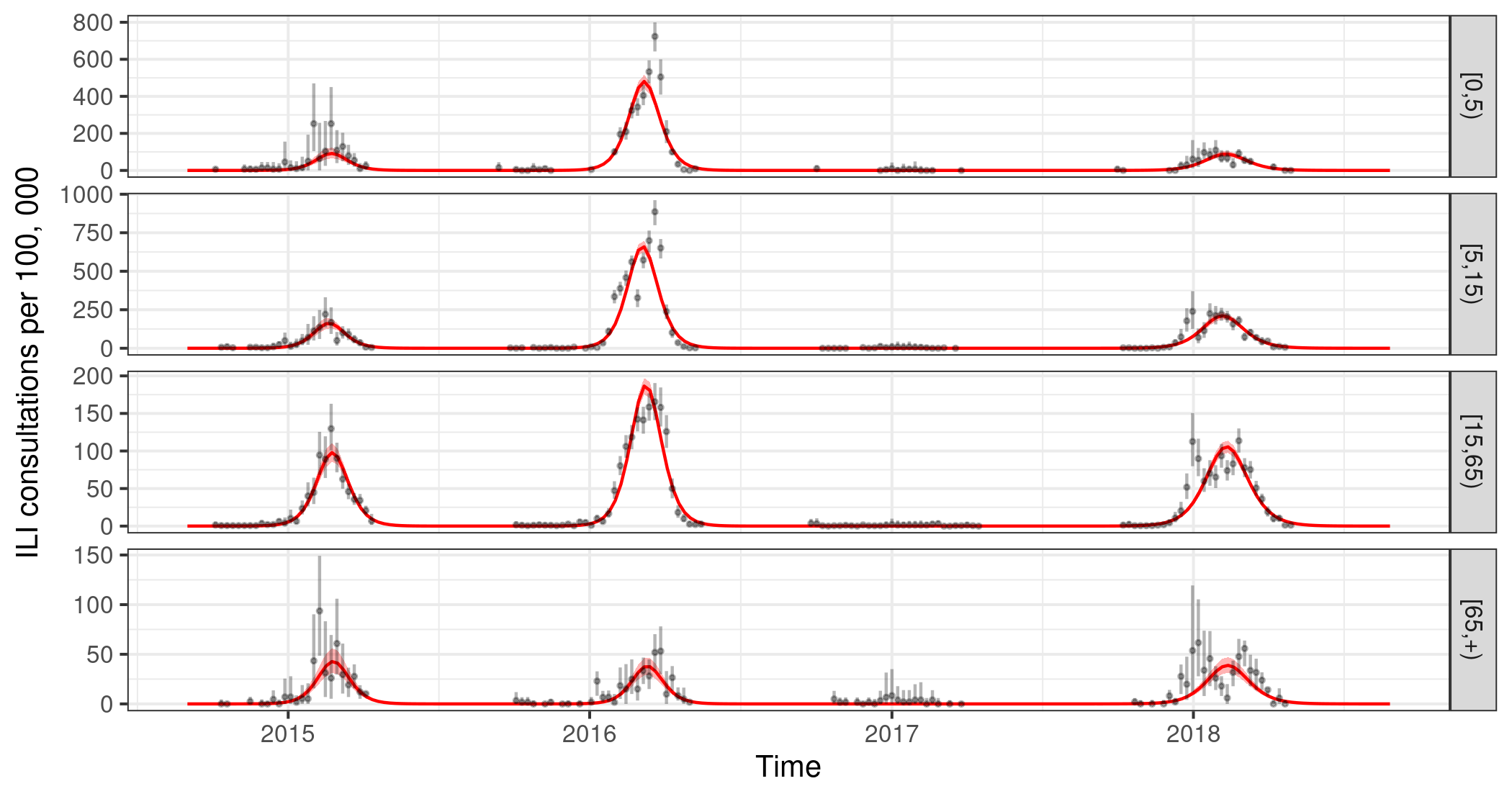


Supplementary Figure 18: Posterior parameter values for FR and influenza virus subtype H1N1. Box plots highlight the median, the 50% CI and the 95% CI. Susceptibility and ascertainment rates are assumed to be different for children, adults and elderly.


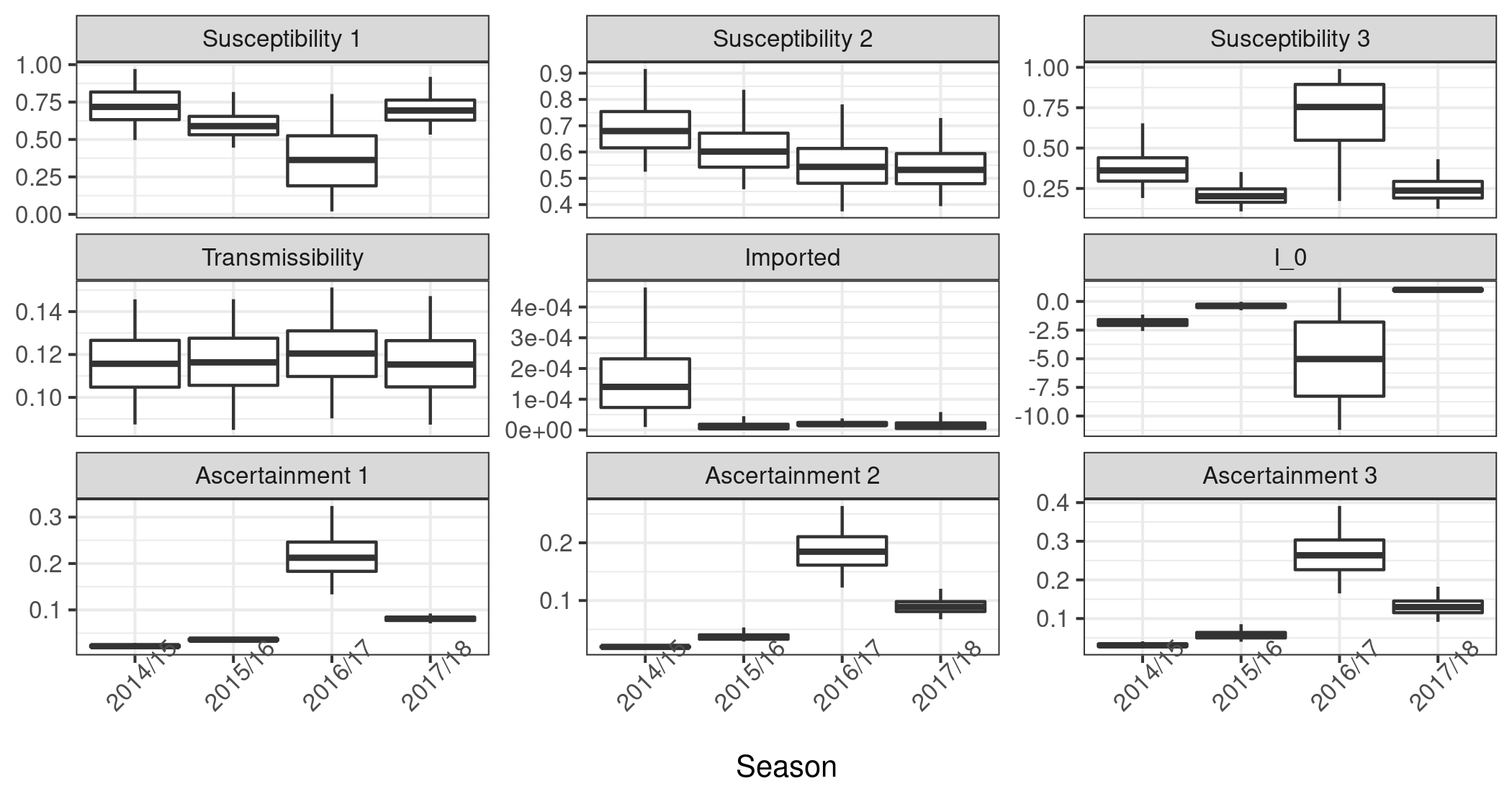


Supplementary Figure 19: Fit of the model to the data for FR and influenza virus subtype H1N1. Red indicates the predicted values, while black shows the data and associated uncertainty (95% CI).


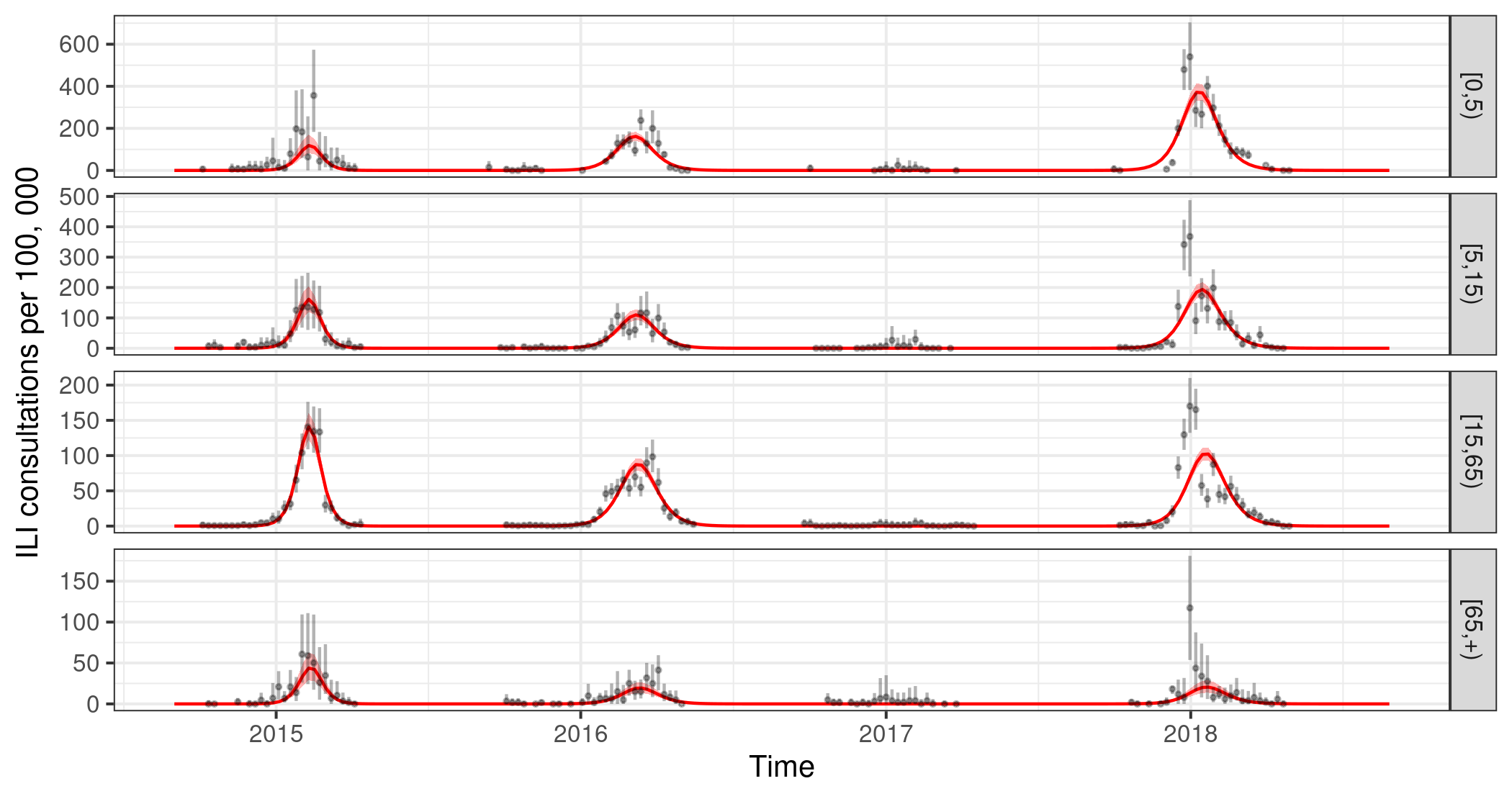


Supplementary Figure 20: Posterior parameter values for FR and influenza virus subtype H3N2. Box plots highlight the median, the 50% CI and the 95% CI. Susceptibility and ascertainment rates are assumed to be different for children, adults and elderly.


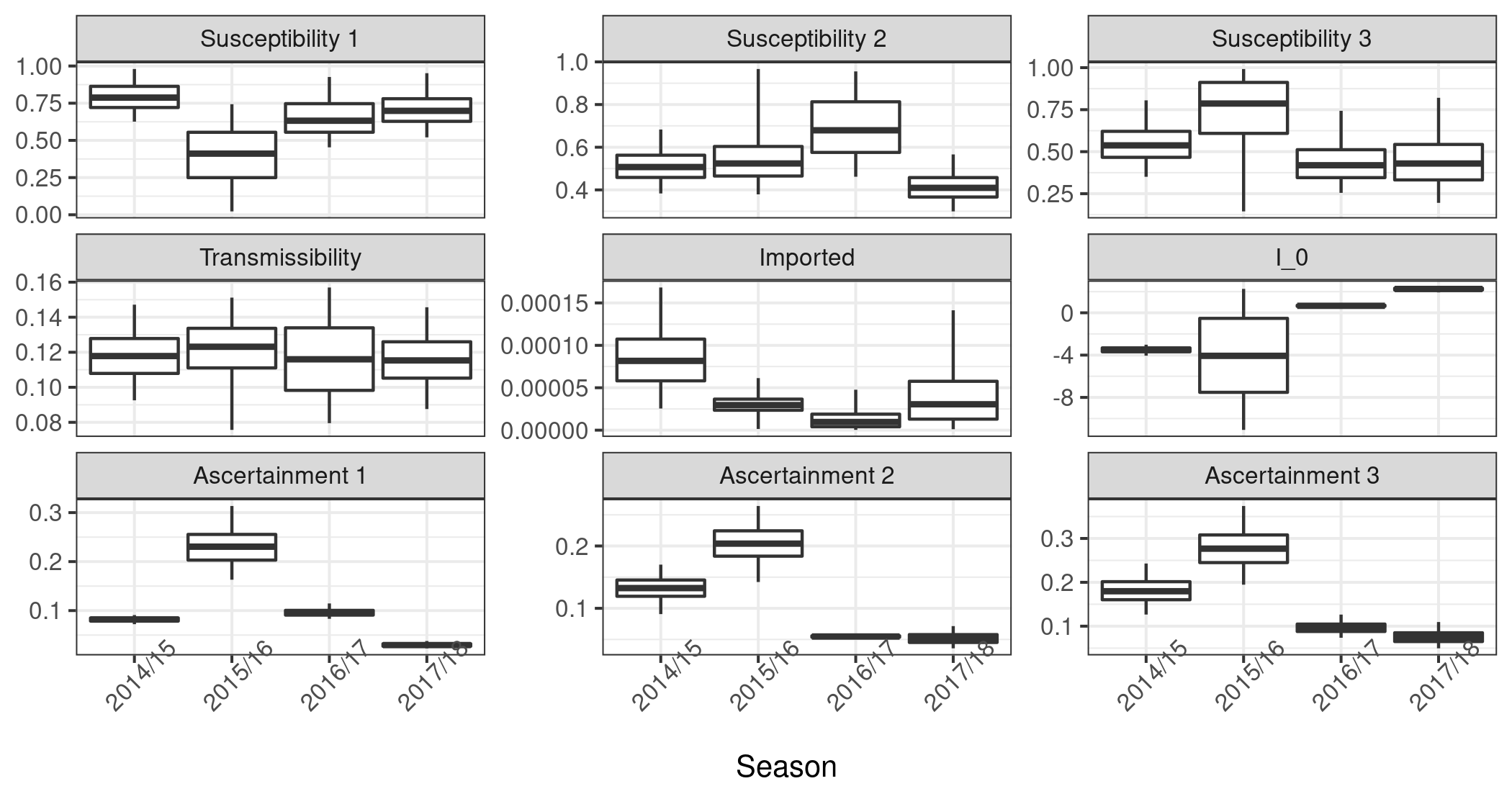


Supplementary Figure 21: Fit of the model to the data for FR and influenza virus subtype H3N2. Red indicates the predicted values, while black shows the data and associated uncertainty (95% CI).


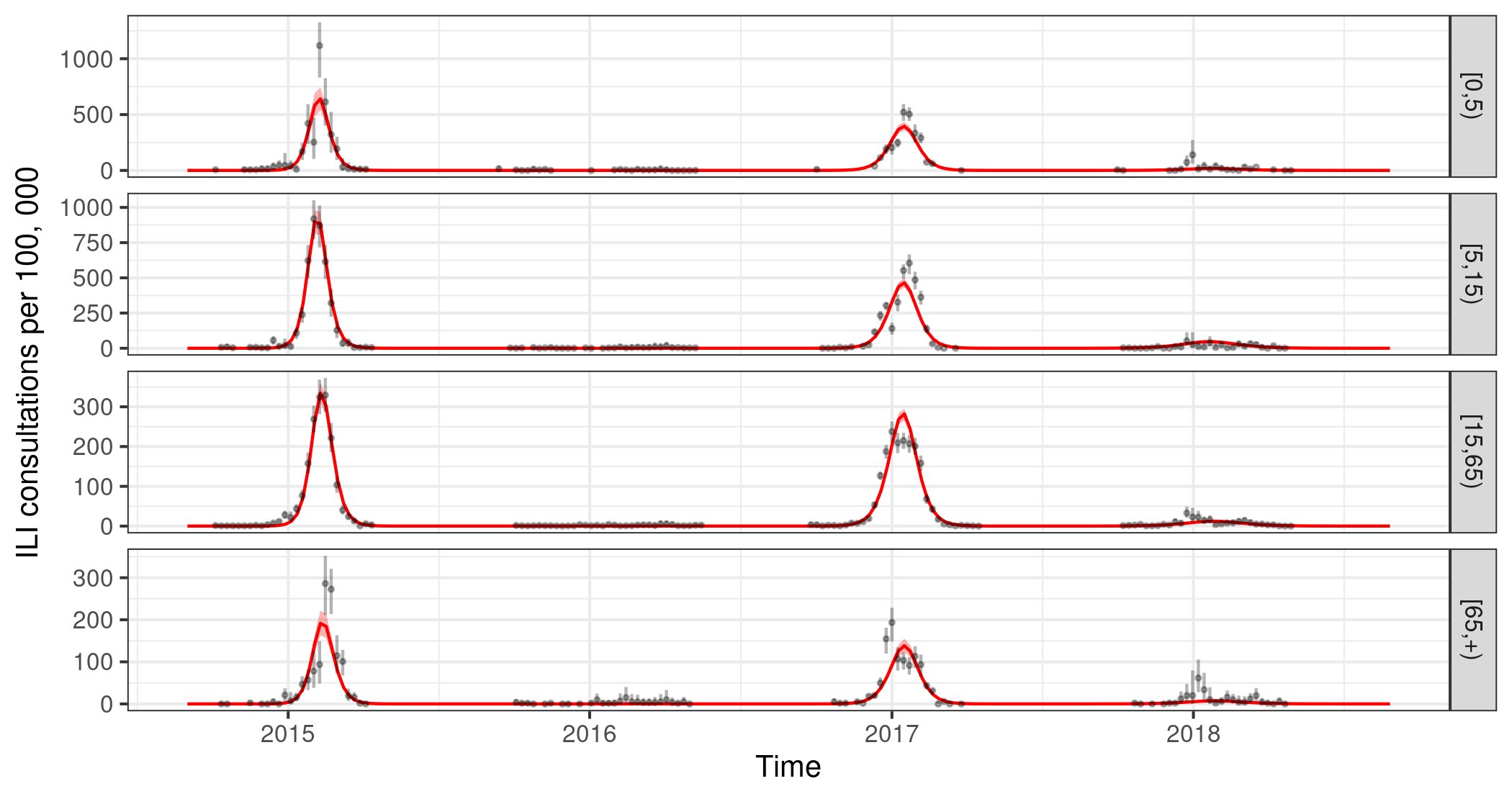


Supplementary Figure 22: Posterior parameter values for IE and influenza virus subtype B. Box plots highlight the median, the 50% CI and the 95% CI. Susceptibility and ascertainment rates are assumed to be different for children, adults and elderly.


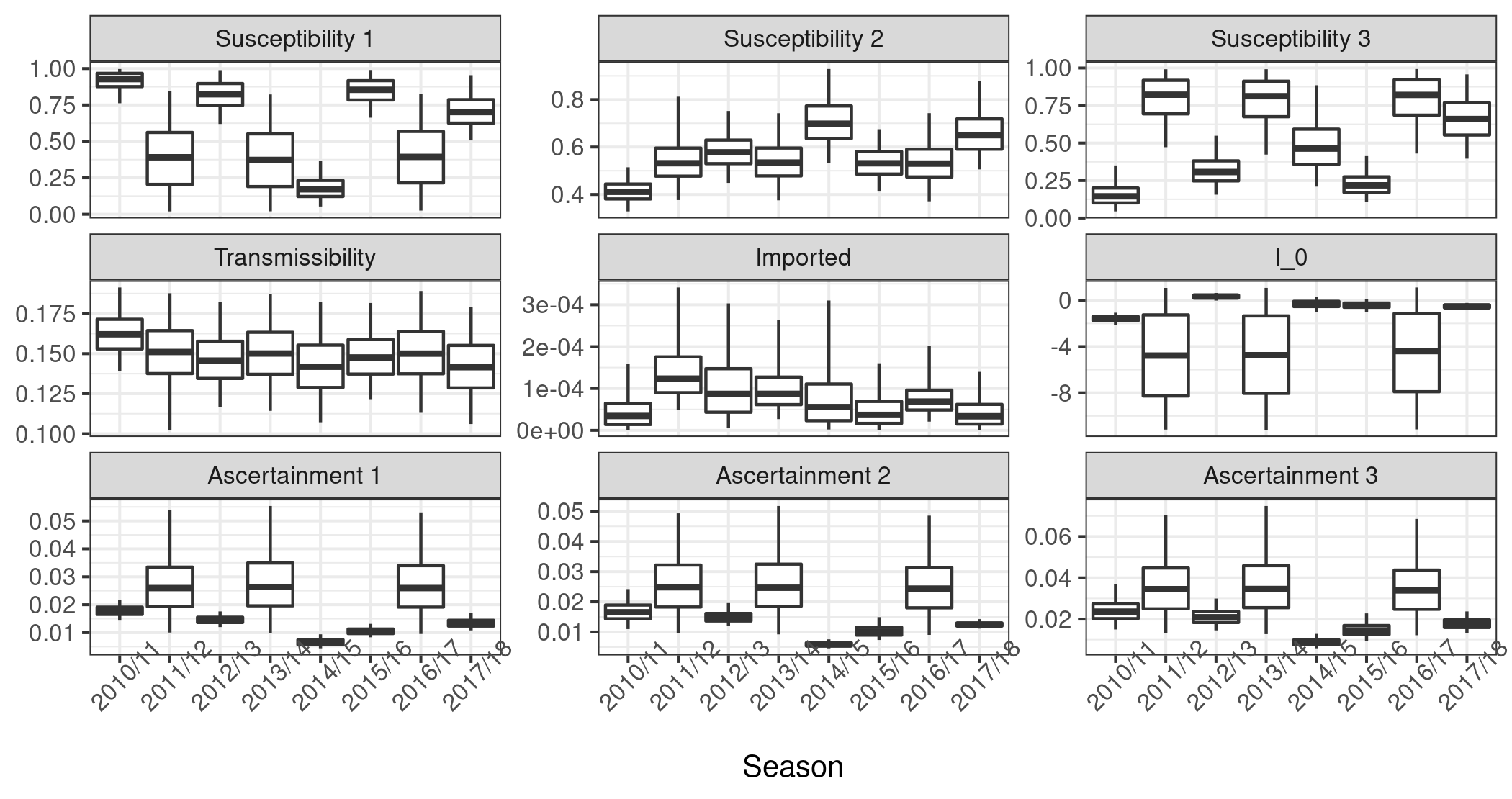


Supplementary Figure 23: Fit of the model to the data for IE and influenza virus subtype B. Red indicates the predicted values, while black shows the data and associated uncertainty (95% CI).


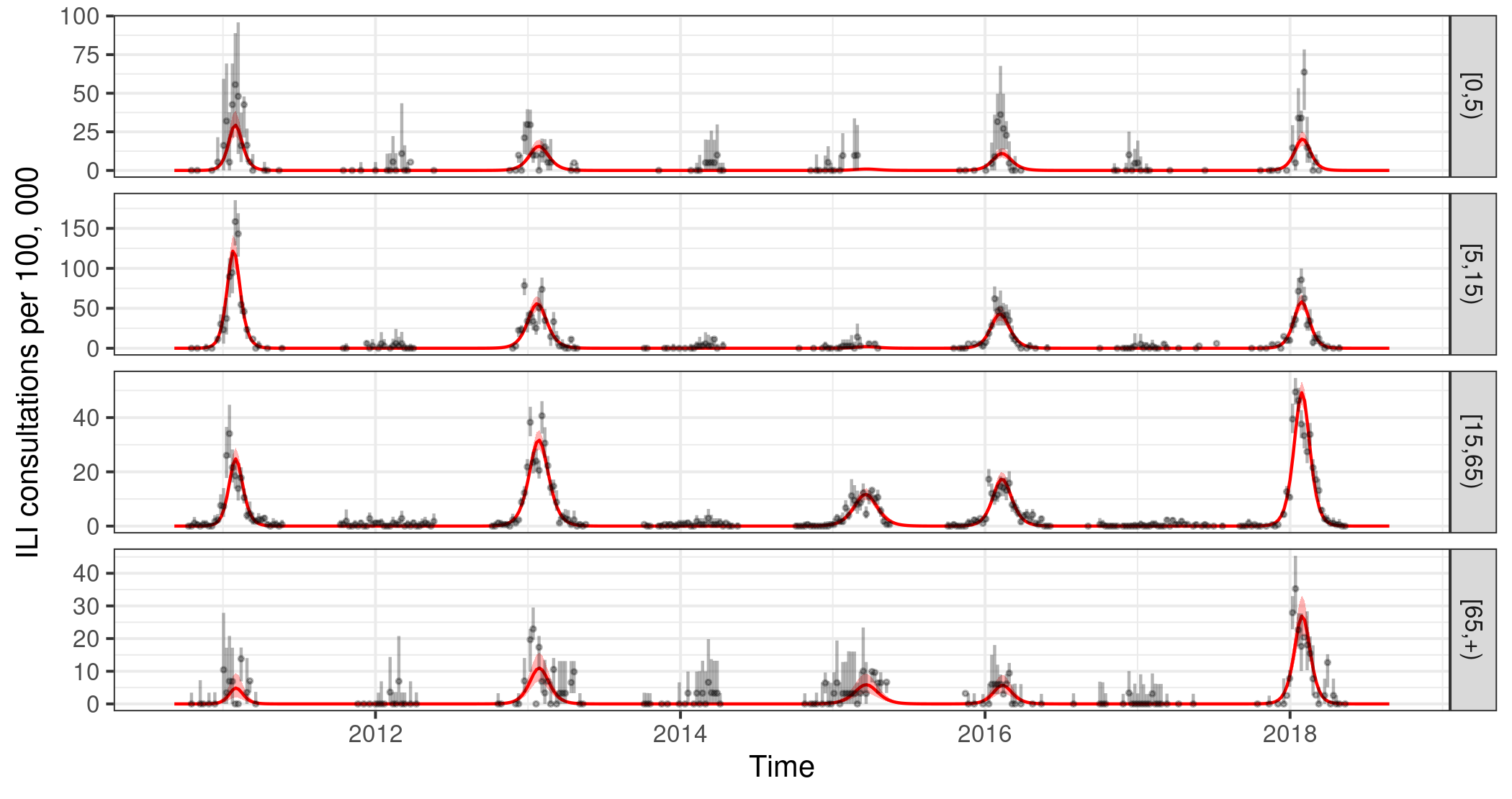


Supplementary Figure 24: Posterior parameter values for IE and influenza virus subtype H1N1. Box plots highlight the median, the 50% CI and the 95% CI. Susceptibility and ascertainment rates are assumed to be different for children, adults and elderly.


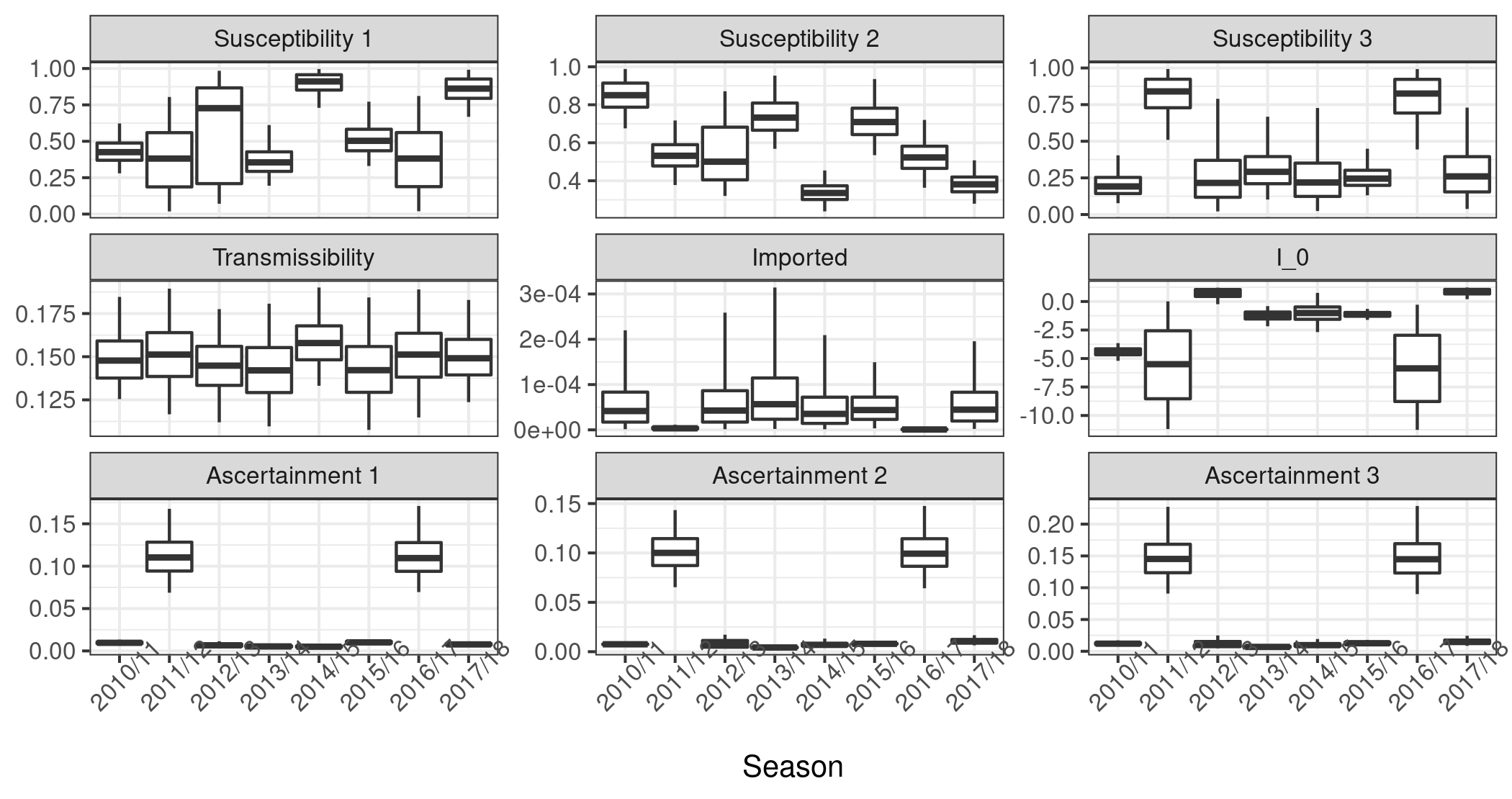


Supplementary Figure 25: Fit of the model to the data for IE and influenza virus subtype H1N1. Red indicates the predicted values, while black shows the data and associated uncertainty (95% CI).


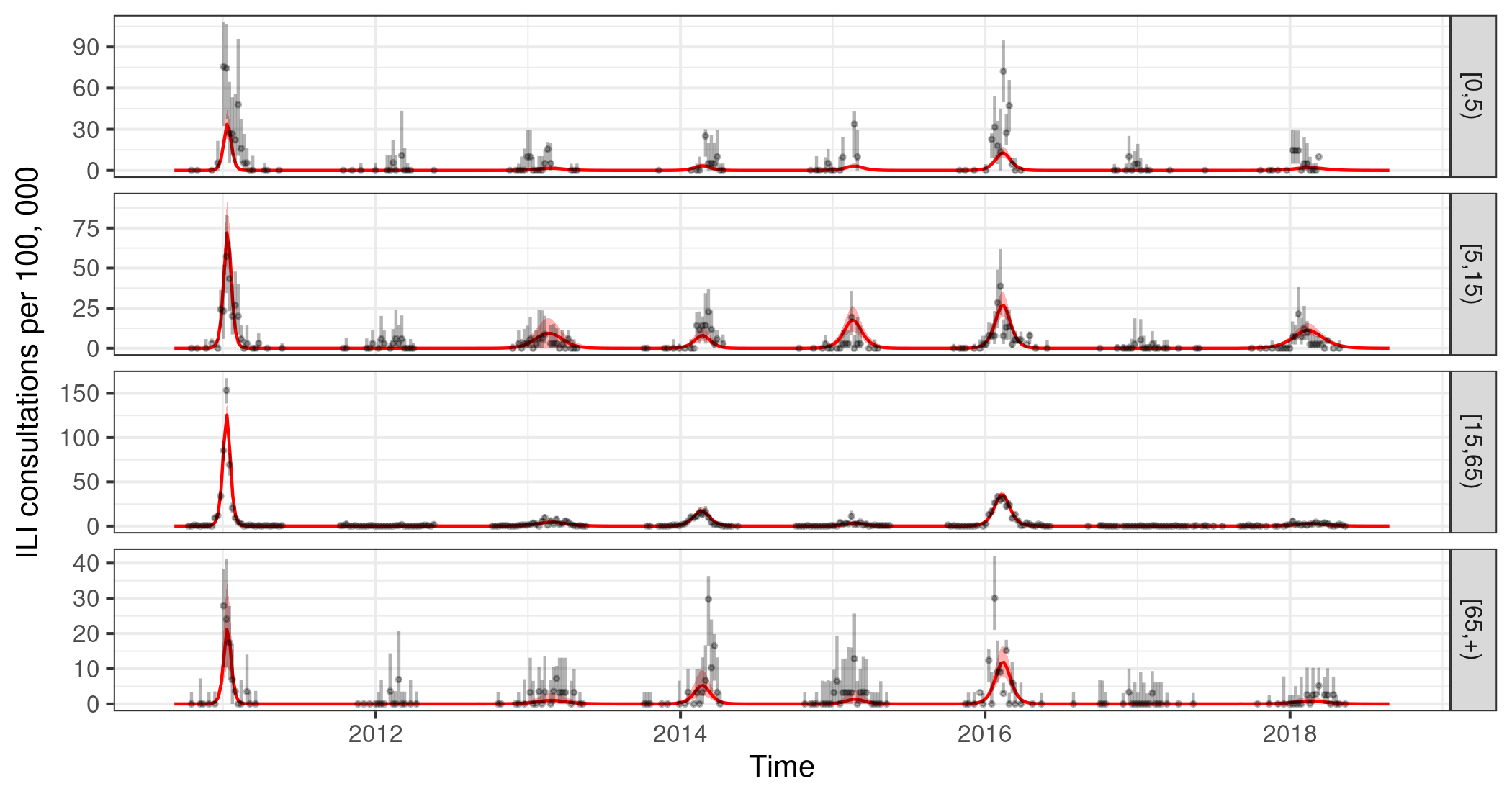


Supplementary Figure 26: Posterior parameter values for IE and influenza virus subtype H3N2. Box plots highlight the median, the 50% CI and the 95% CI. Susceptibility and ascertainment rates are assumed to be different for children, adults and elderly.


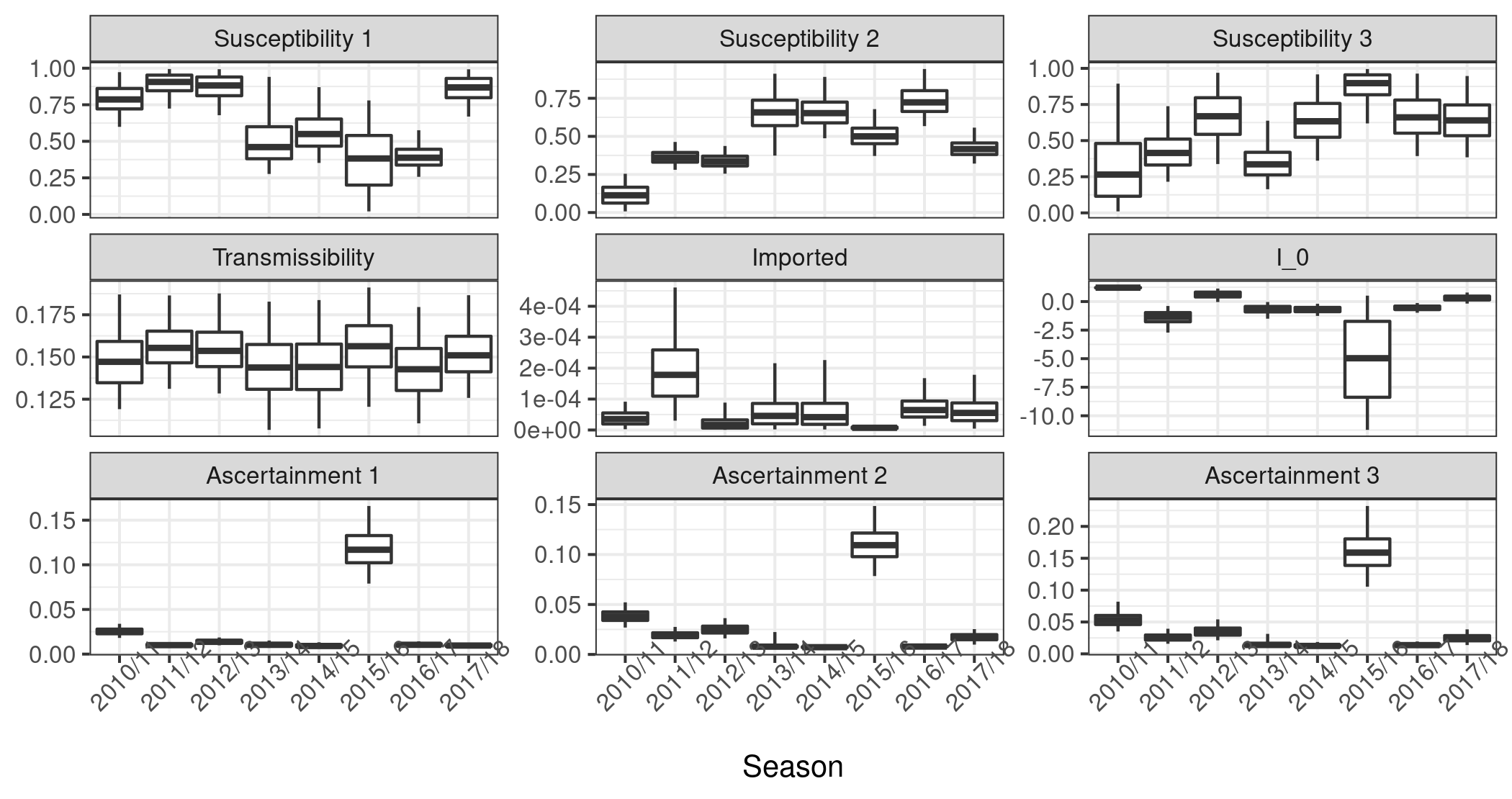


Supplementary Figure 27: Fit of the model to the data for IE and influenza virus subtype H3N2. Red indicates the predicted values, while black shows the data and associated uncertainty (95% CI).


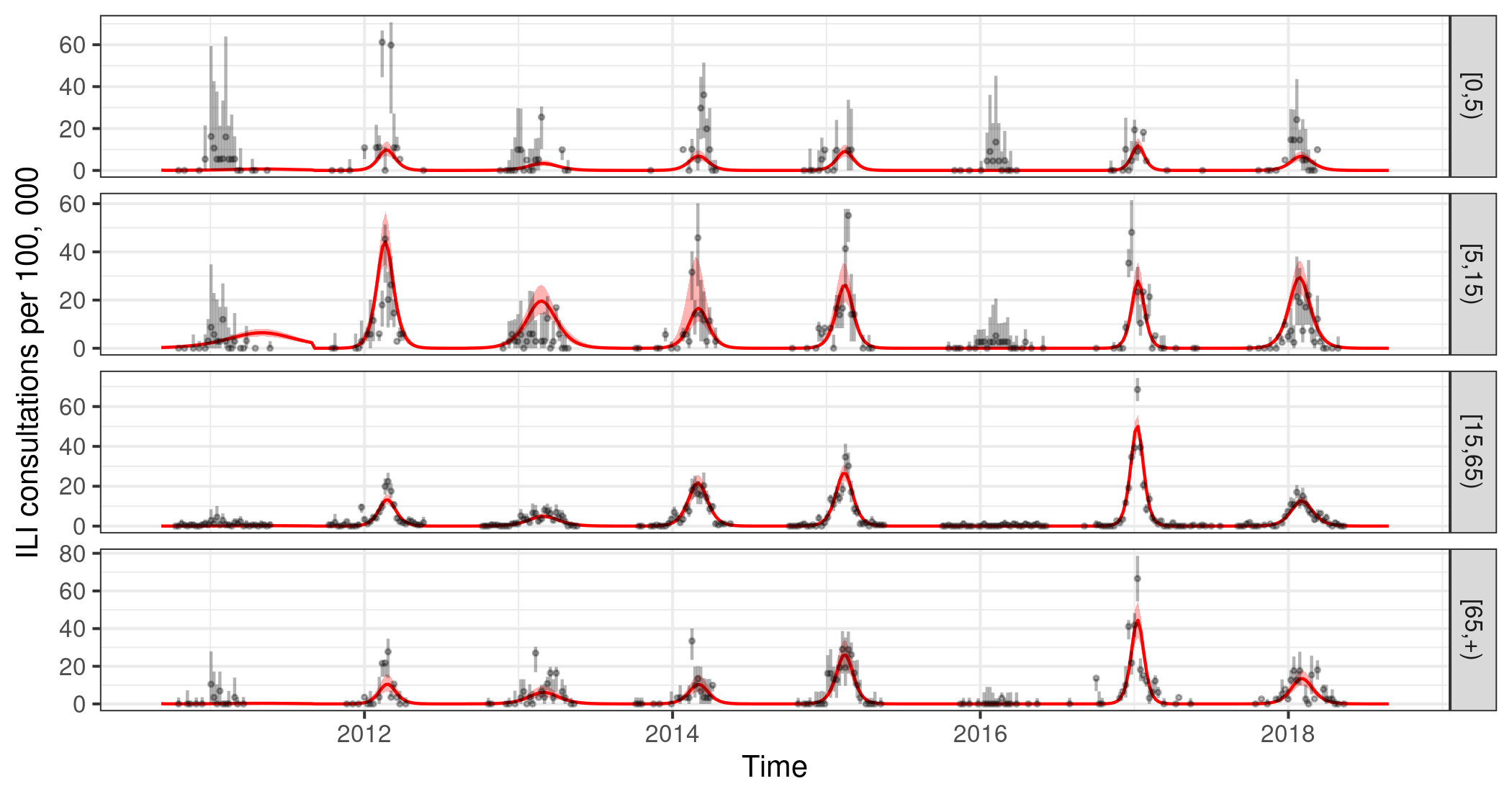


Supplementary Figure 28: Posterior parameter values for NL and influenza virus subtype B. Box plots highlight the median, the 50% CI and the 95% CI. Susceptibility and ascertainment rates are assumed to be different for children, adults and elderly.


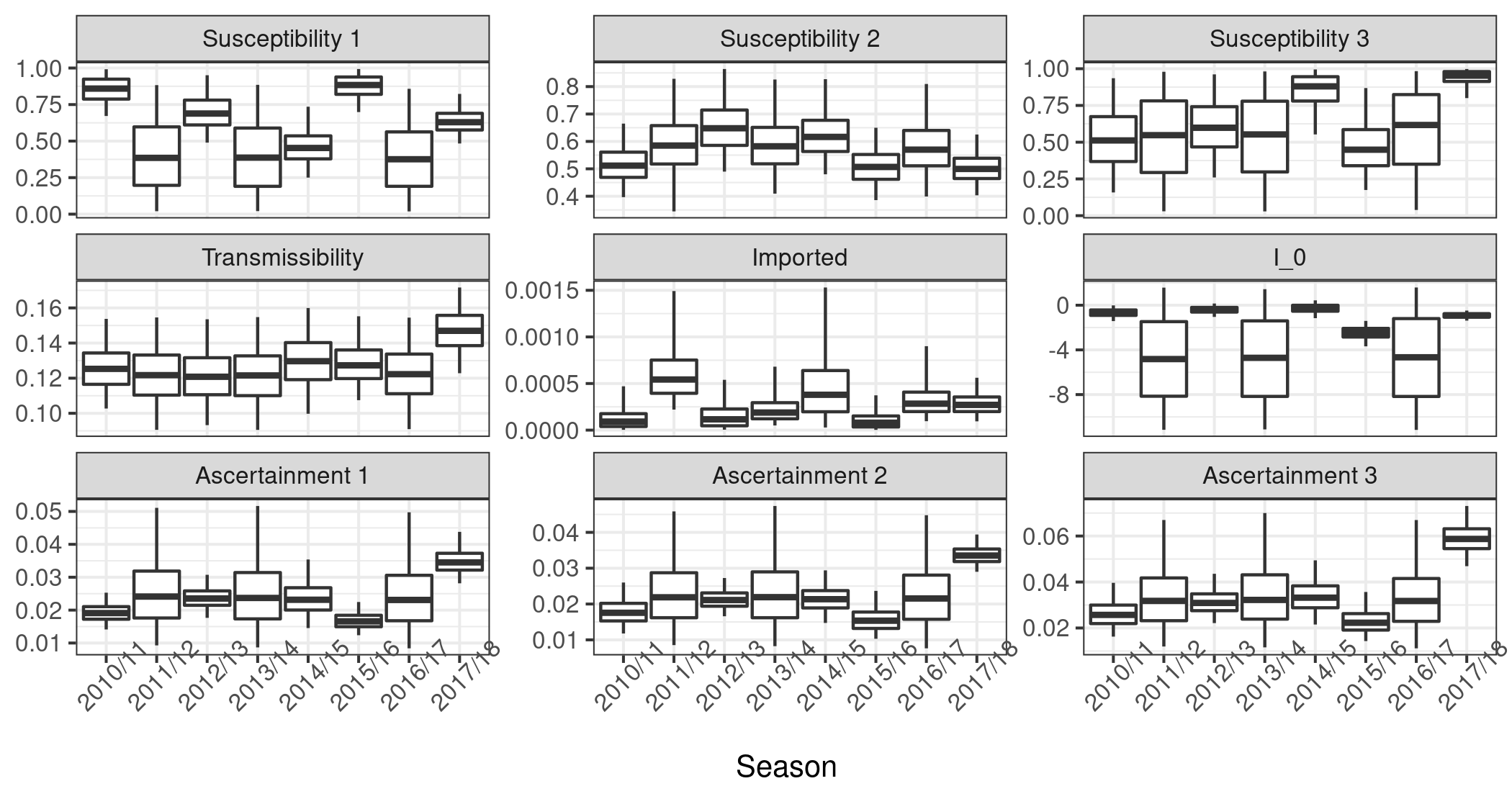


Supplementary Figure 29: Fit of the model to the data for NL and influenza virus subtype B. Red indicates the predicted values, while black shows the data and associated uncertainty (95% CI).


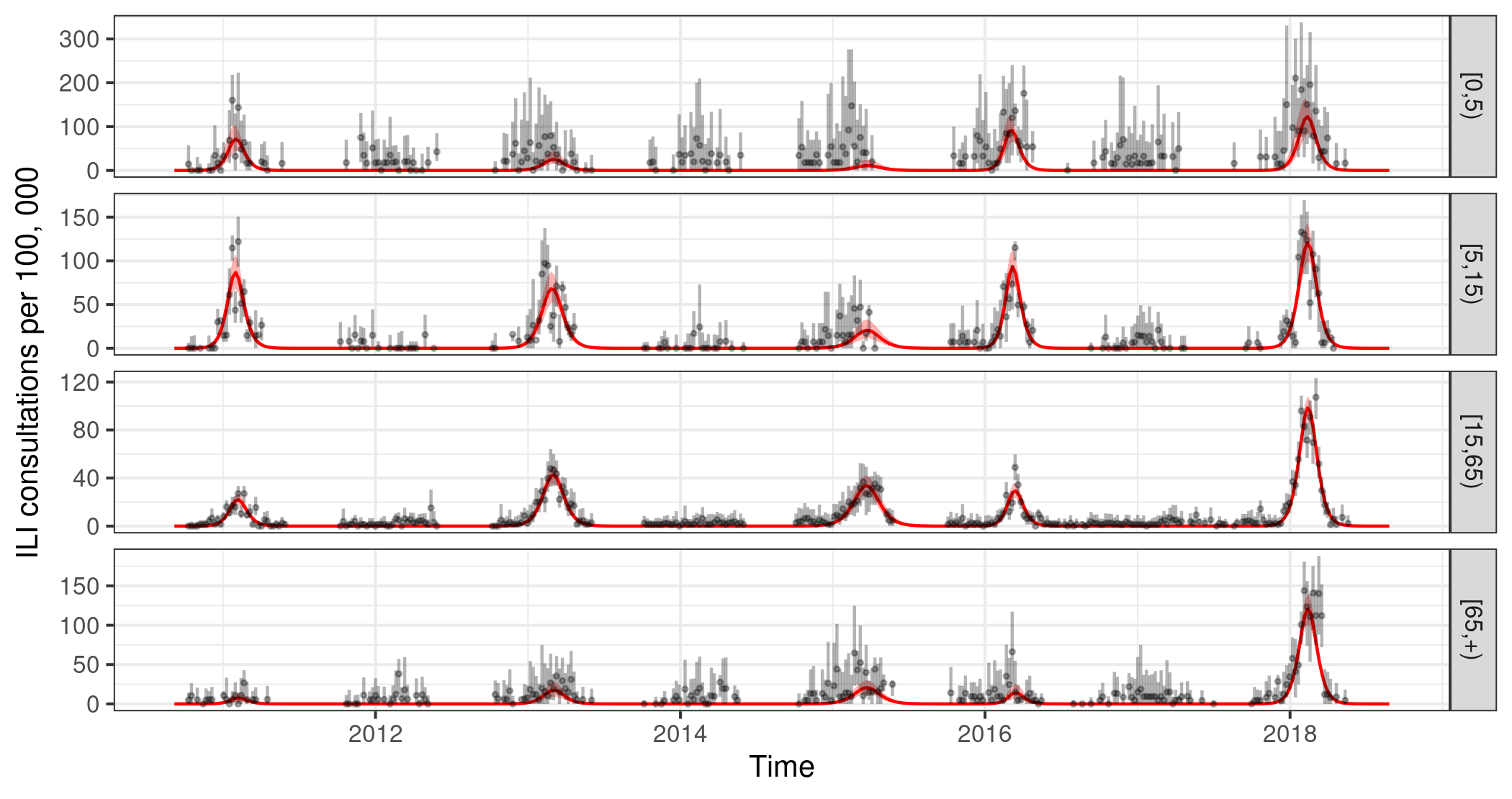


Supplementary Figure 30: Posterior parameter values for NL and influenza virus subtype H1N1. Box plots highlight the median, the 50% CI and the 95% CI. Susceptibility and ascertainment rates are assumed to be different for children, adults and elderly.


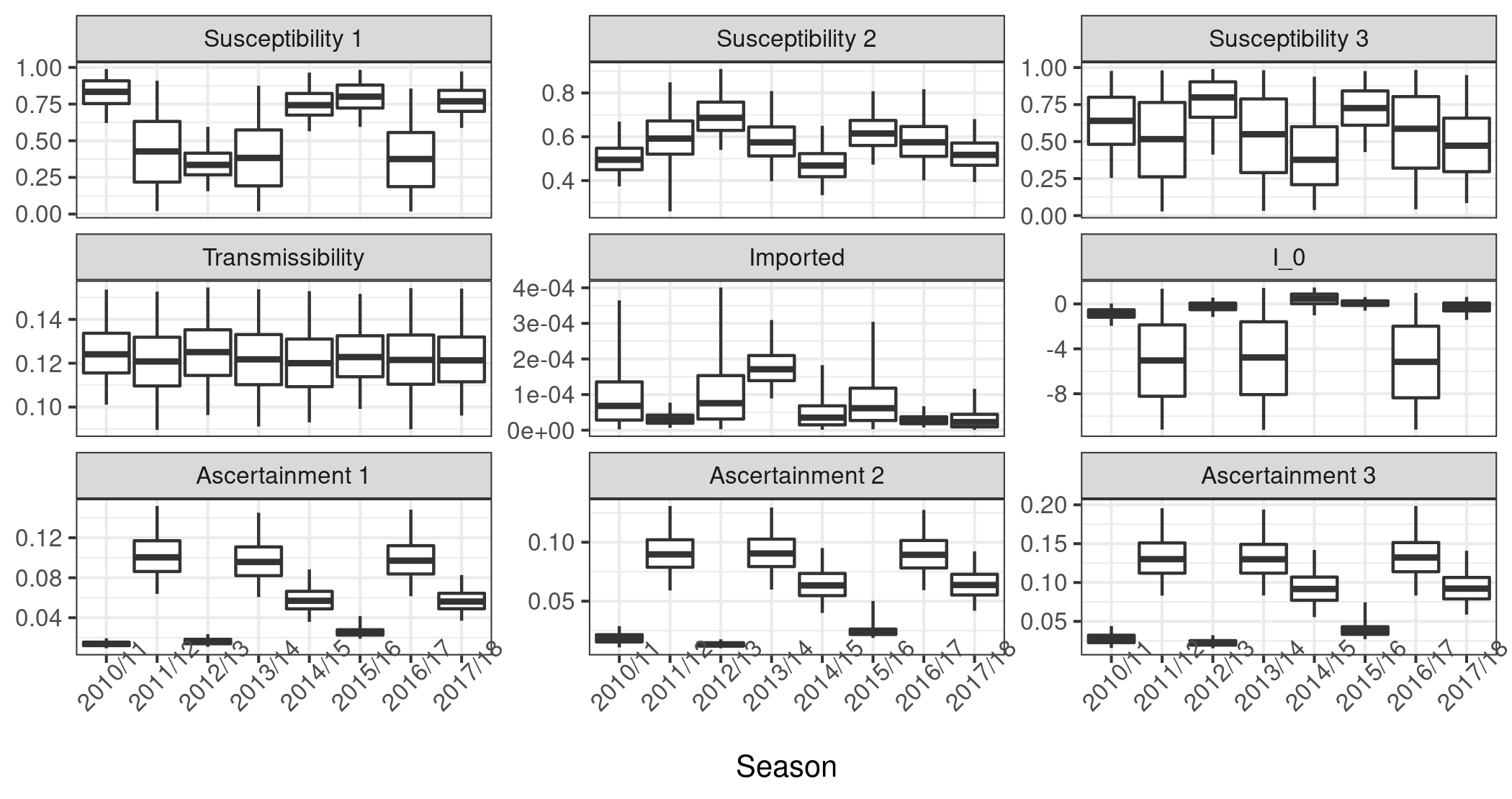


Supplementary Figure 31: Fit of the model to the data for NL and influenza virus subtype H1N1. Red indicates the predicted values, while black shows the data and associated uncertainty (95% CI).


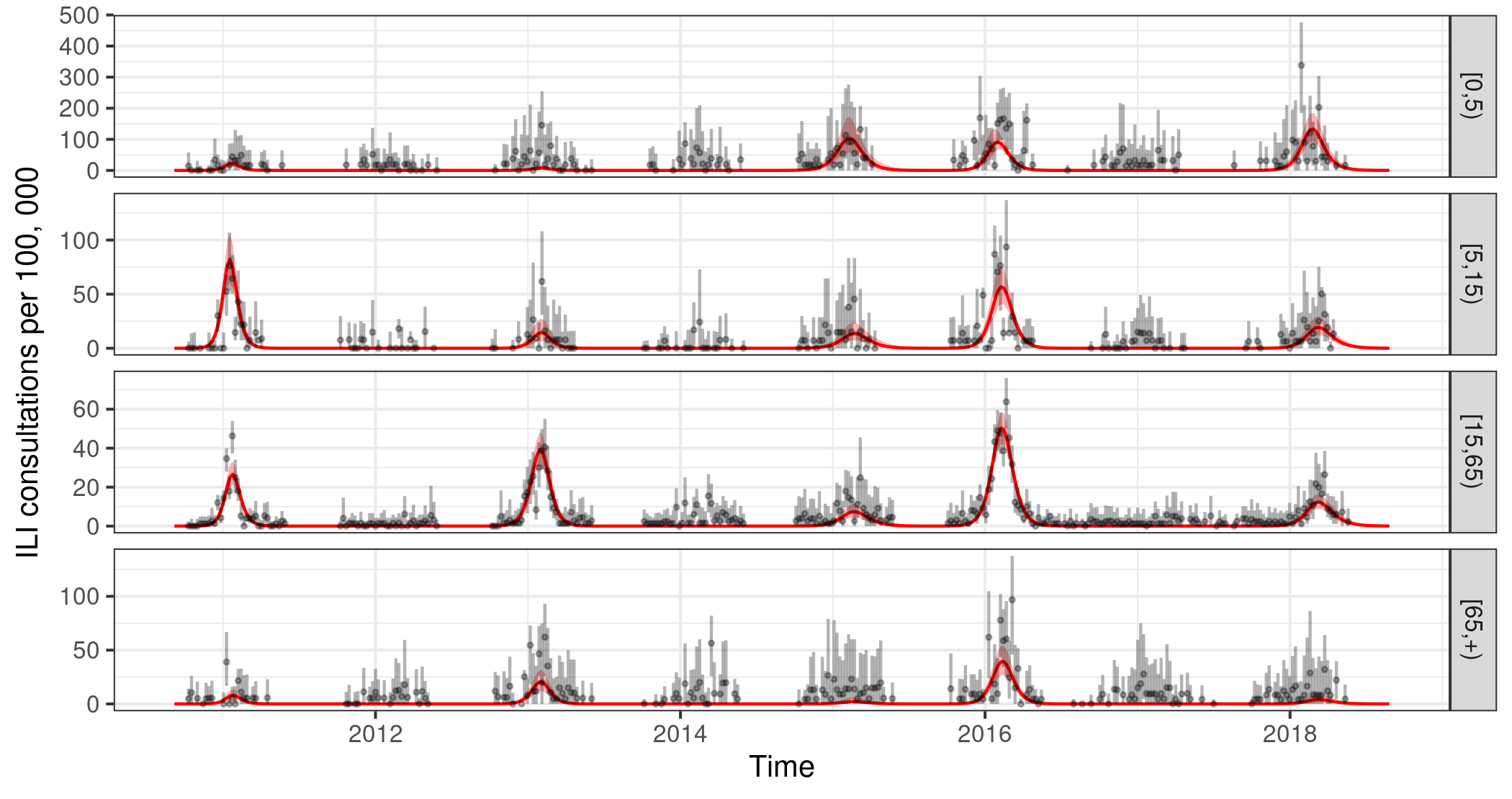


Supplementary Figure 32: Posterior parameter values for NL and influenza virus subtype H3N2. Box plots highlight the median, the 50% CI and the 95% CI. Susceptibility and ascertainment rates are assumed to be different for children, adults and elderly.


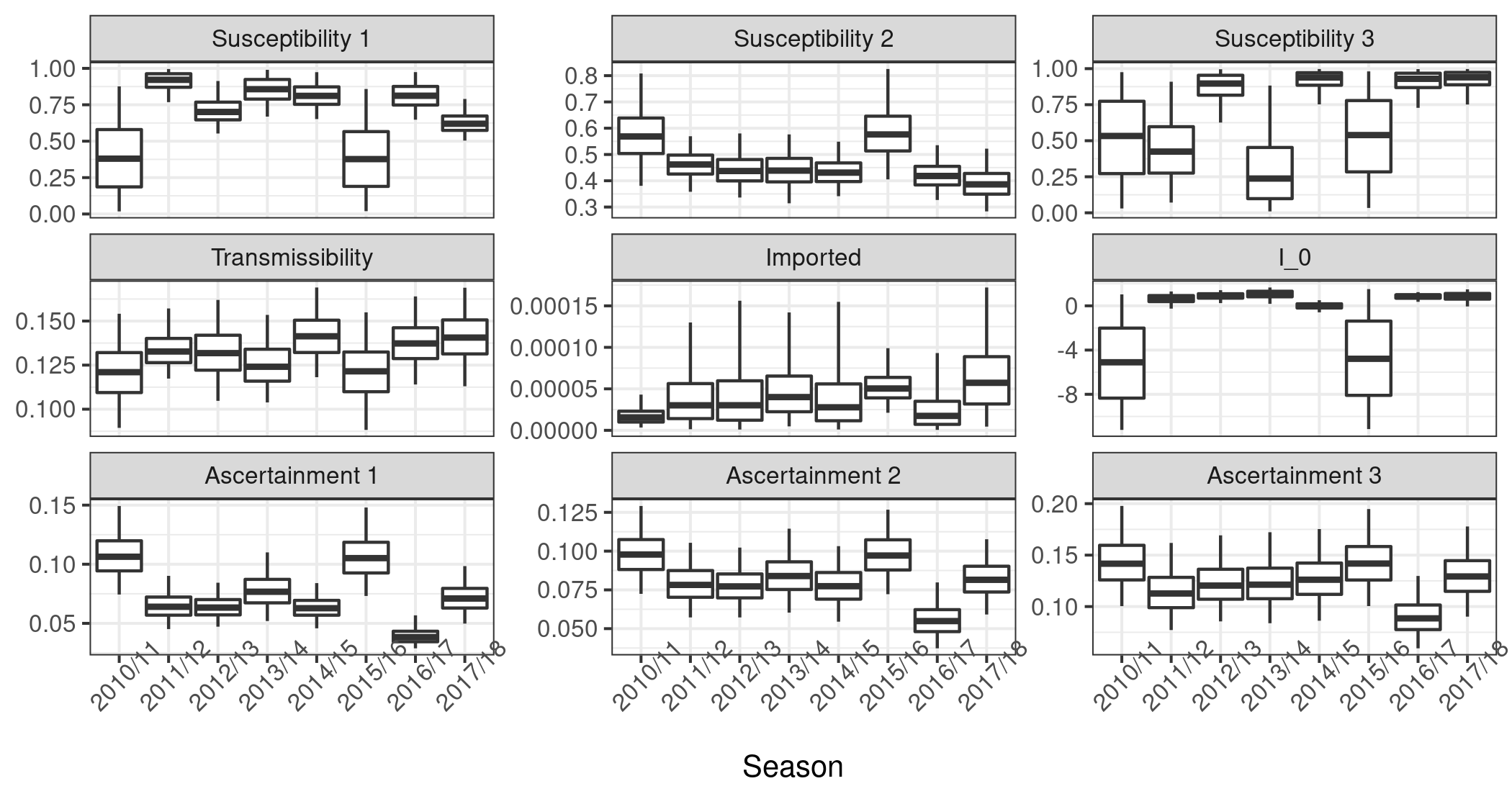


Supplementary Figure 33: Fit of the model to the data for NL and influenza virus subtype H3N2. Red indicates the predicted values, while black shows the data and associated uncertainty (95% CI).


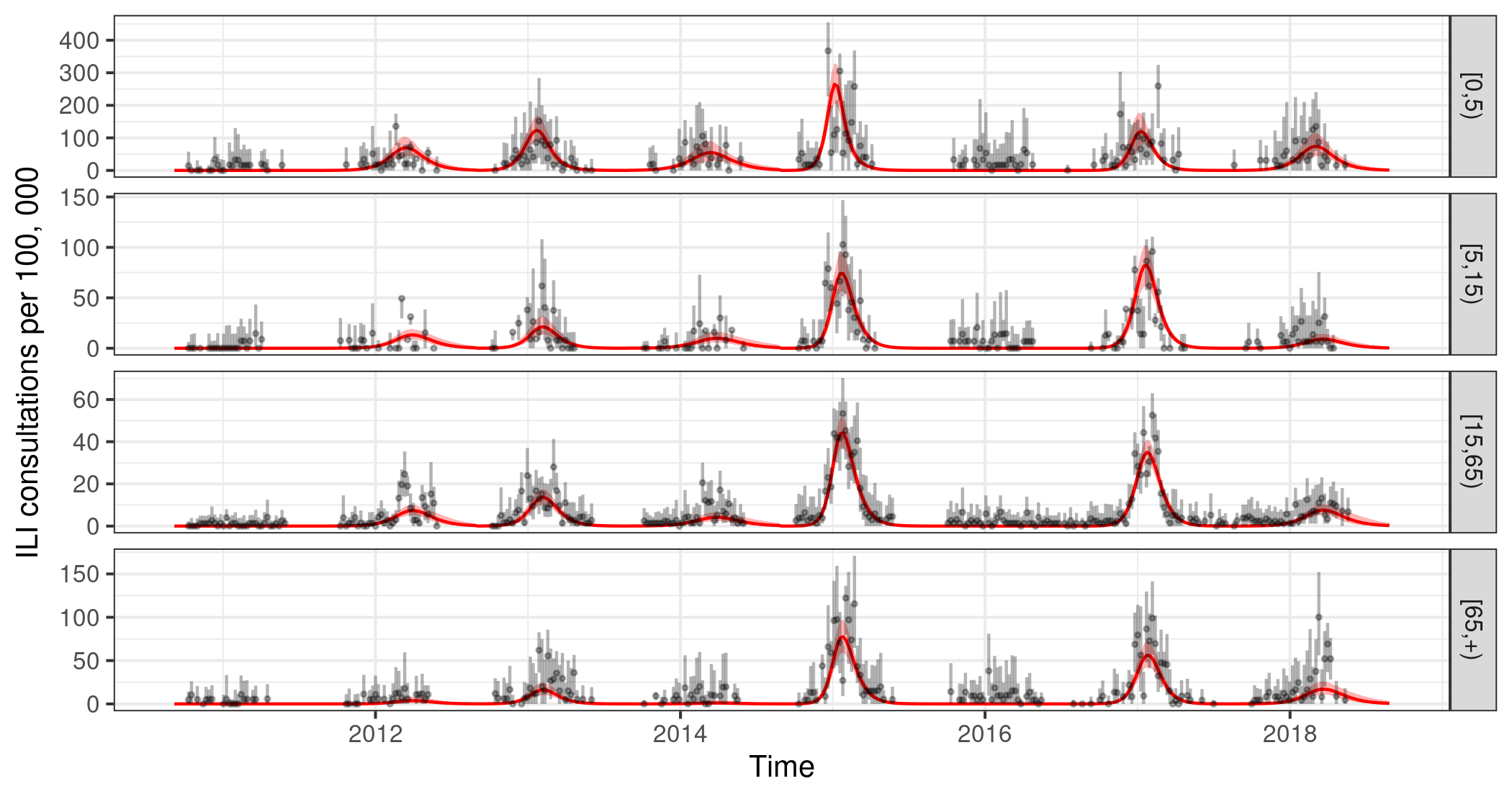


Supplementary Figure 34: Posterior parameter values for NV and influenza virus subtype B. Box plots highlight the median, the 50% CI and the 95% CI. Susceptibility and ascertainment rates are assumed to be different for children, adults and elderly.


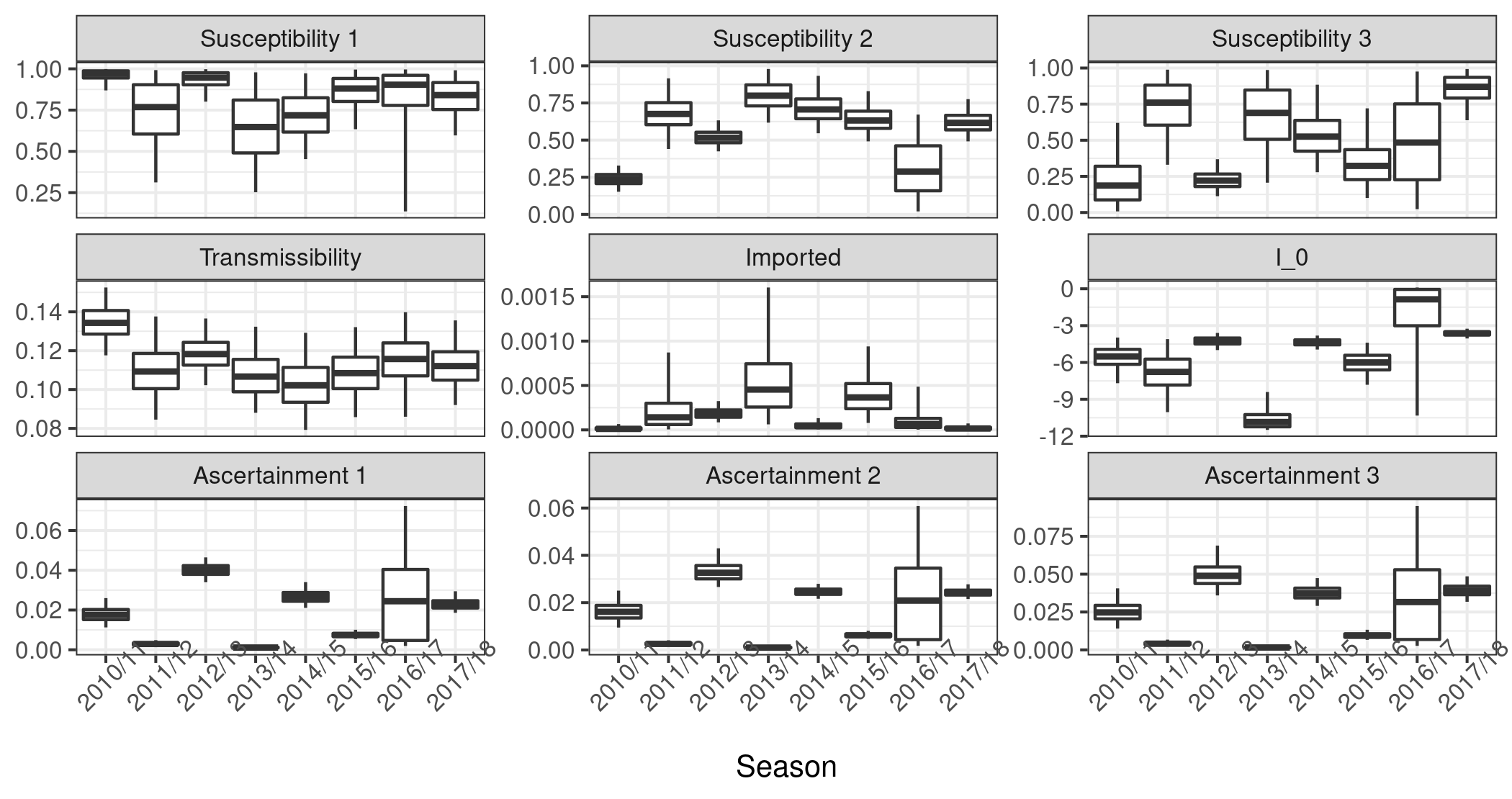


Supplementary Figure 35: Fit of the model to the data for NV and influenza virus subtype B. Red indicates the predicted values, while black shows the data and associated uncertainty (95% CI).


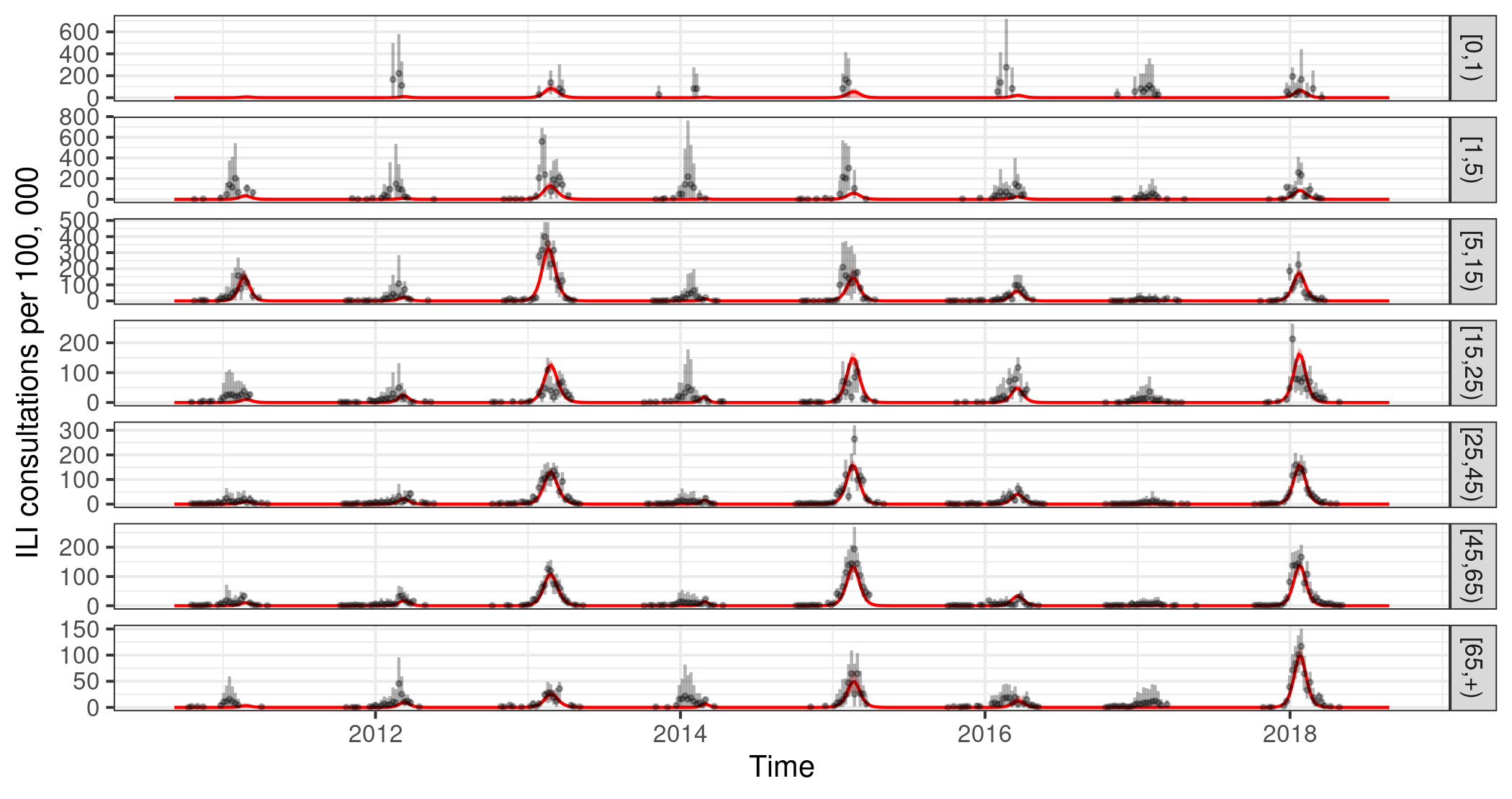


Supplementary Figure 36: Posterior parameter values for NV and influenza virus subtype H1N1. Box plots highlight the median, the 50% CI and the 95% CI. Susceptibility and ascertainment rates are assumed to be different for children, adults and elderly.


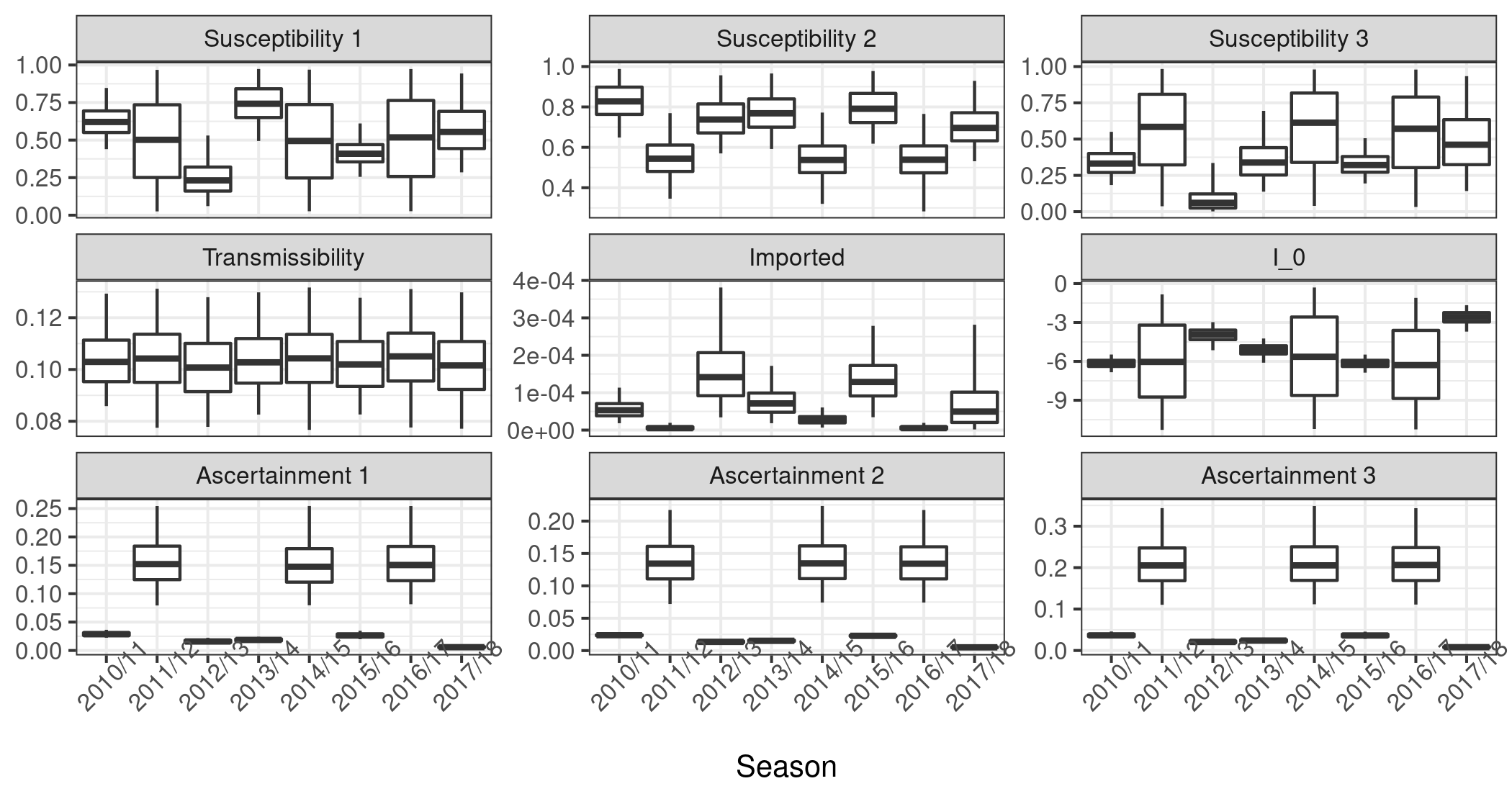


Supplementary Figure 37: Fit of the model to the data for NV and influenza virus subtype H1N1. Red indicates the predicted values, while black shows the data and associated uncertainty (95% CI).


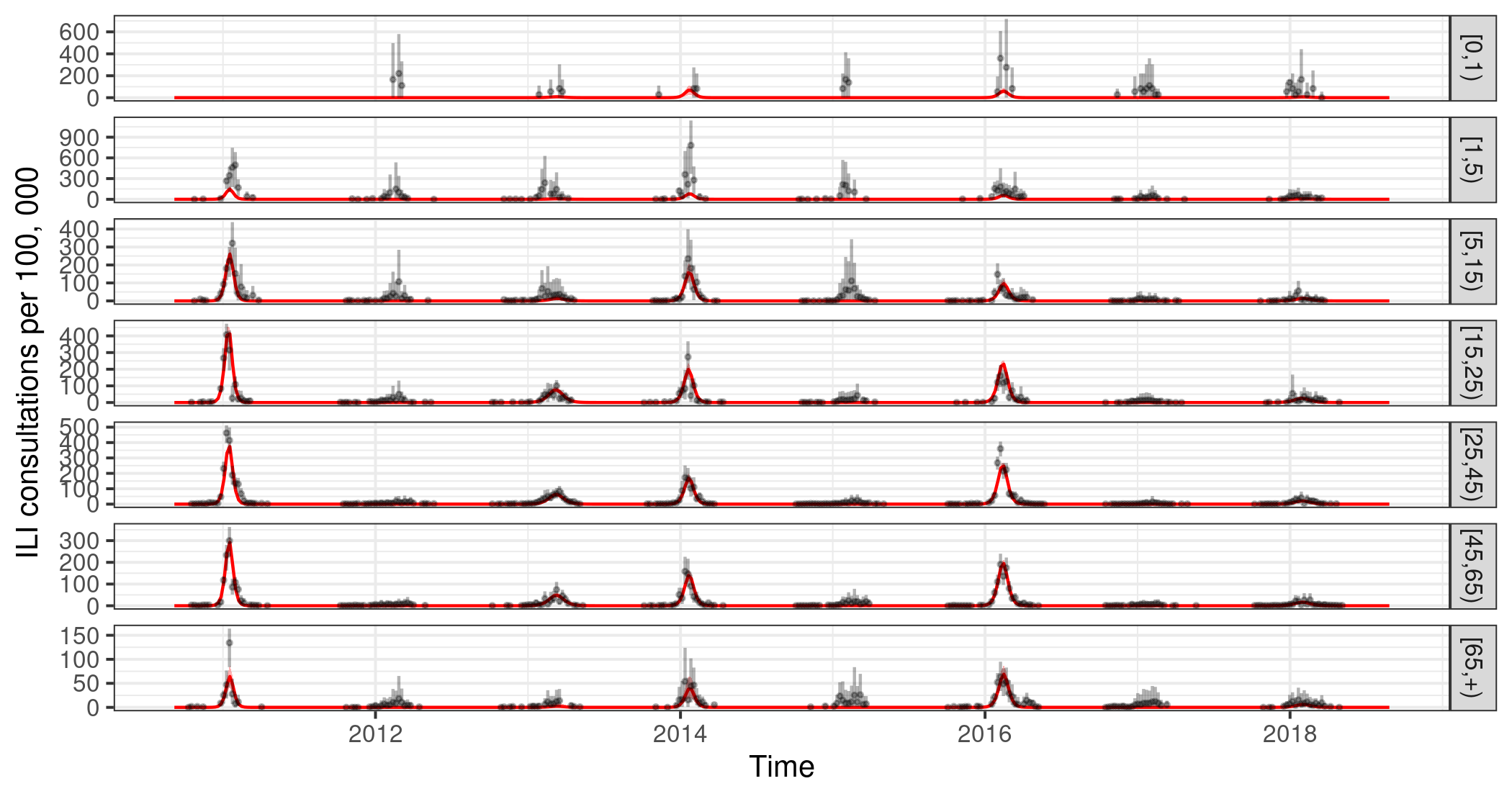


Supplementary Figure 38: Posterior parameter values for NV and influenza virus subtype H3N2. Box plots highlight the median, the 50% CI and the 95% CI. Susceptibility and ascertainment rates are assumed to be different for children, adults and elderly.


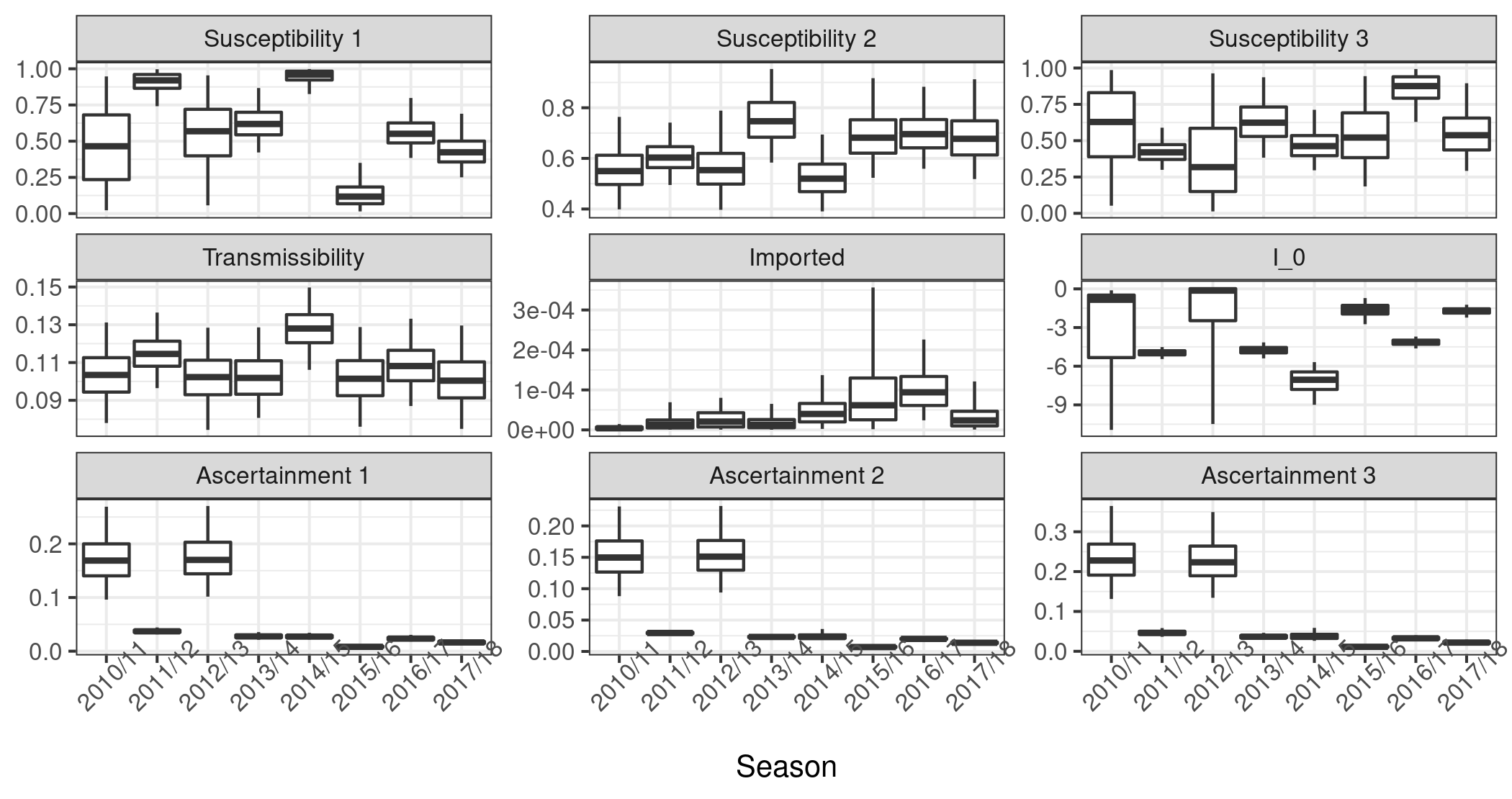


Supplementary Figure 39: Fit of the model to the data for NV and influenza virus subtype H3N2. Red indicates the predicted values, while black shows the data and associated uncertainty (95% CI).


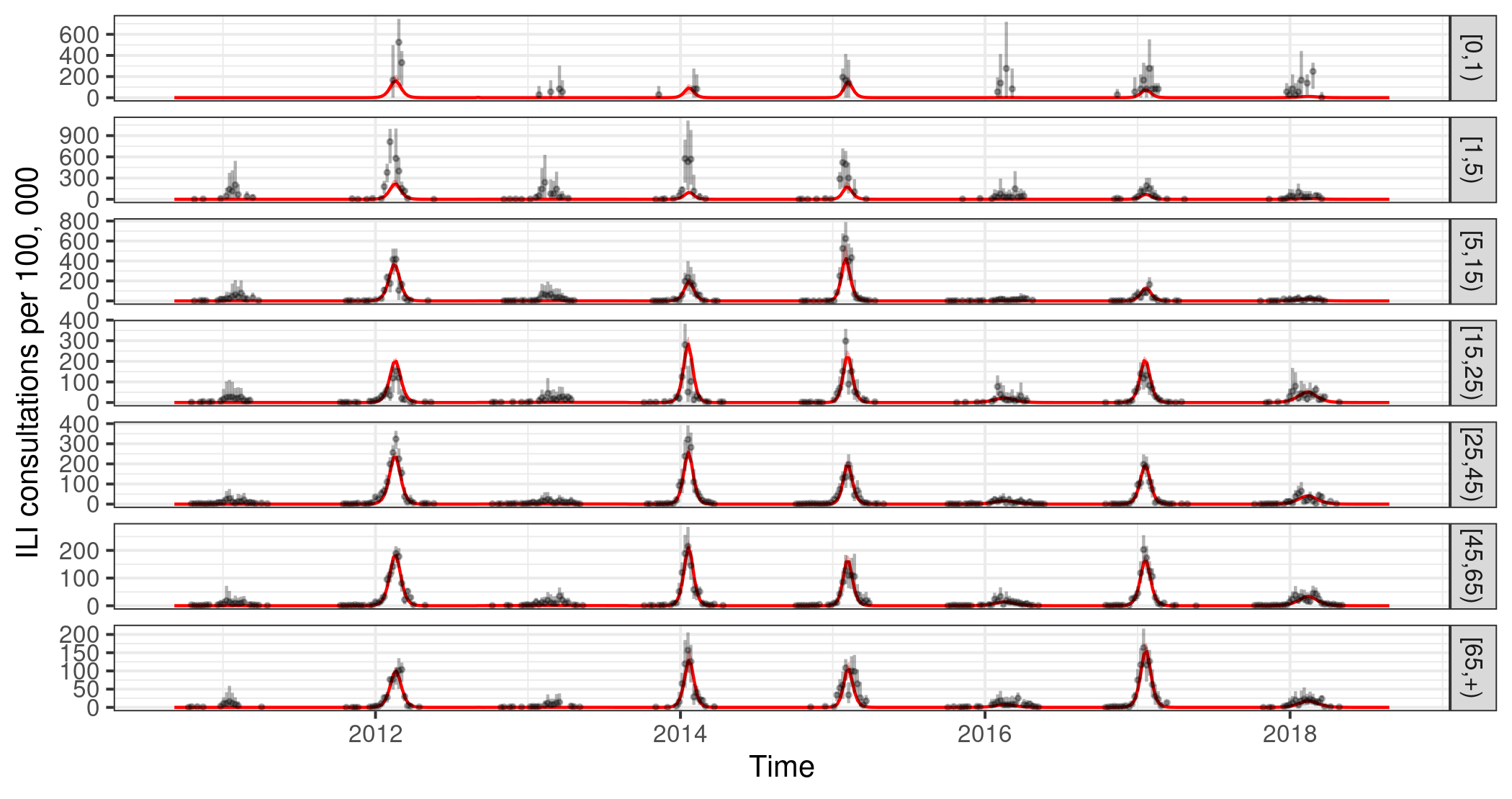


Supplementary Figure 40: Posterior parameter values for PT and influenza virus subtype B. Box plots highlight the median, the 50% CI and the 95% CI. Susceptibility and ascertainment rates are assumed to be different for children, adults and elderly.


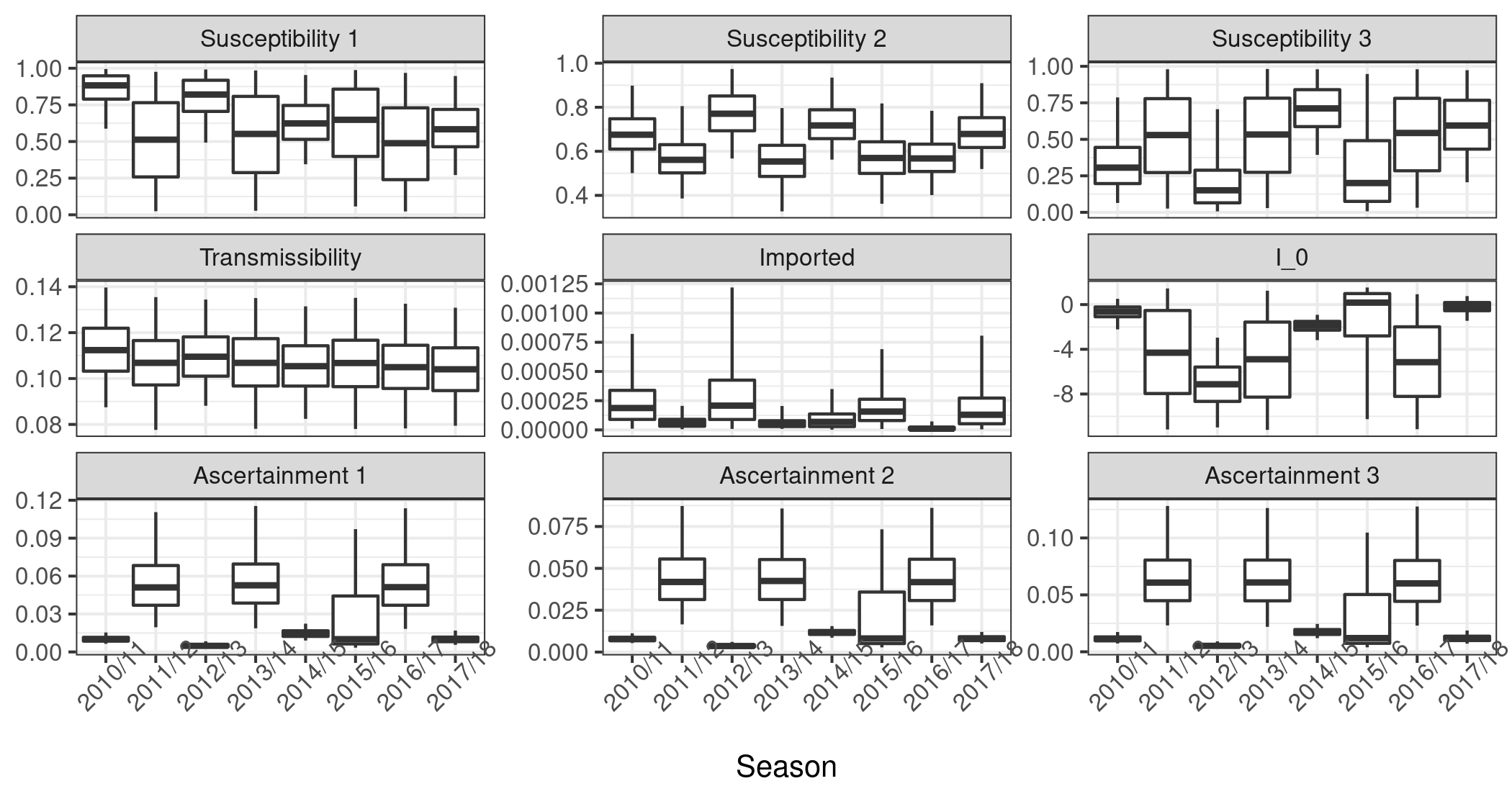


Supplementary Figure 41: Fit of the model to the data for PT and influenza virus subtype B. Red indicates the predicted values, while black shows the data and associated uncertainty (95% CI).


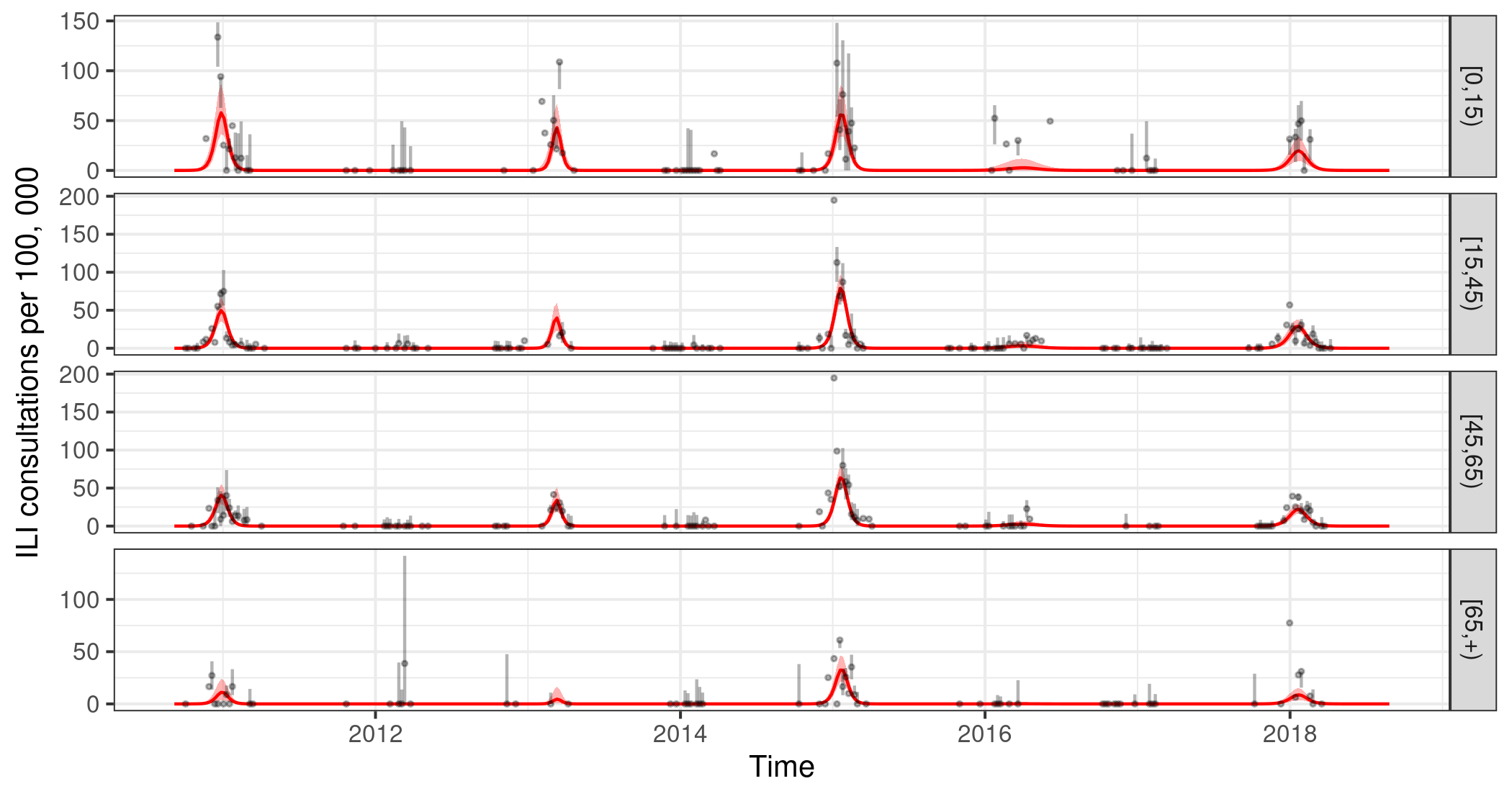


Supplementary Figure 42: Posterior parameter values for PT and influenza virus subtype H1N1. Box plots highlight the median, the 50% CI and the 95% CI. Susceptibility and ascertainment rates are assumed to be different for children, adults and elderly.


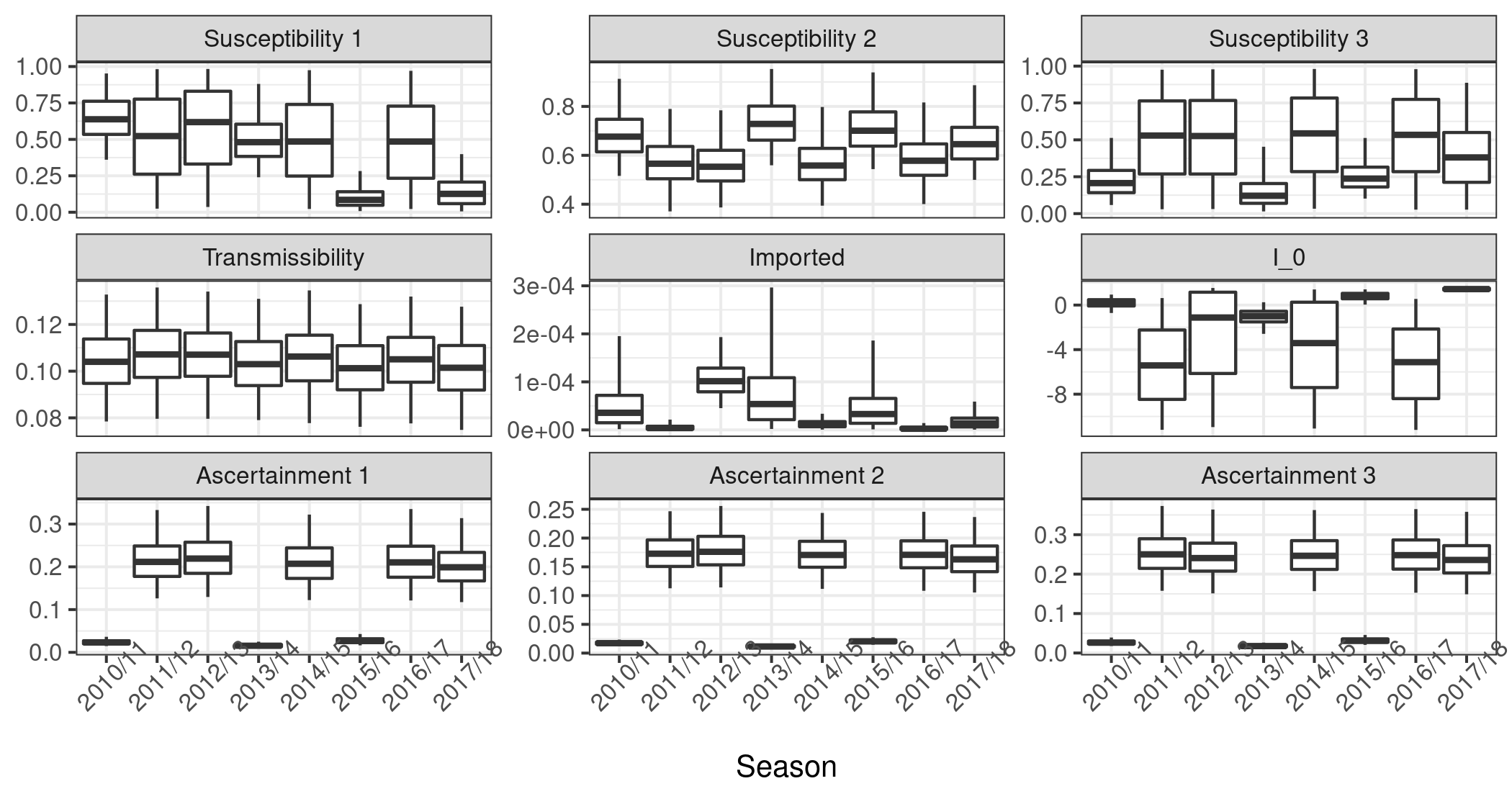


Supplementary Figure 43: Fit of the model to the data for PT and influenza virus subtype H1N1. Red indicates the predicted values, while black shows the data and associated uncertainty (95% CI).


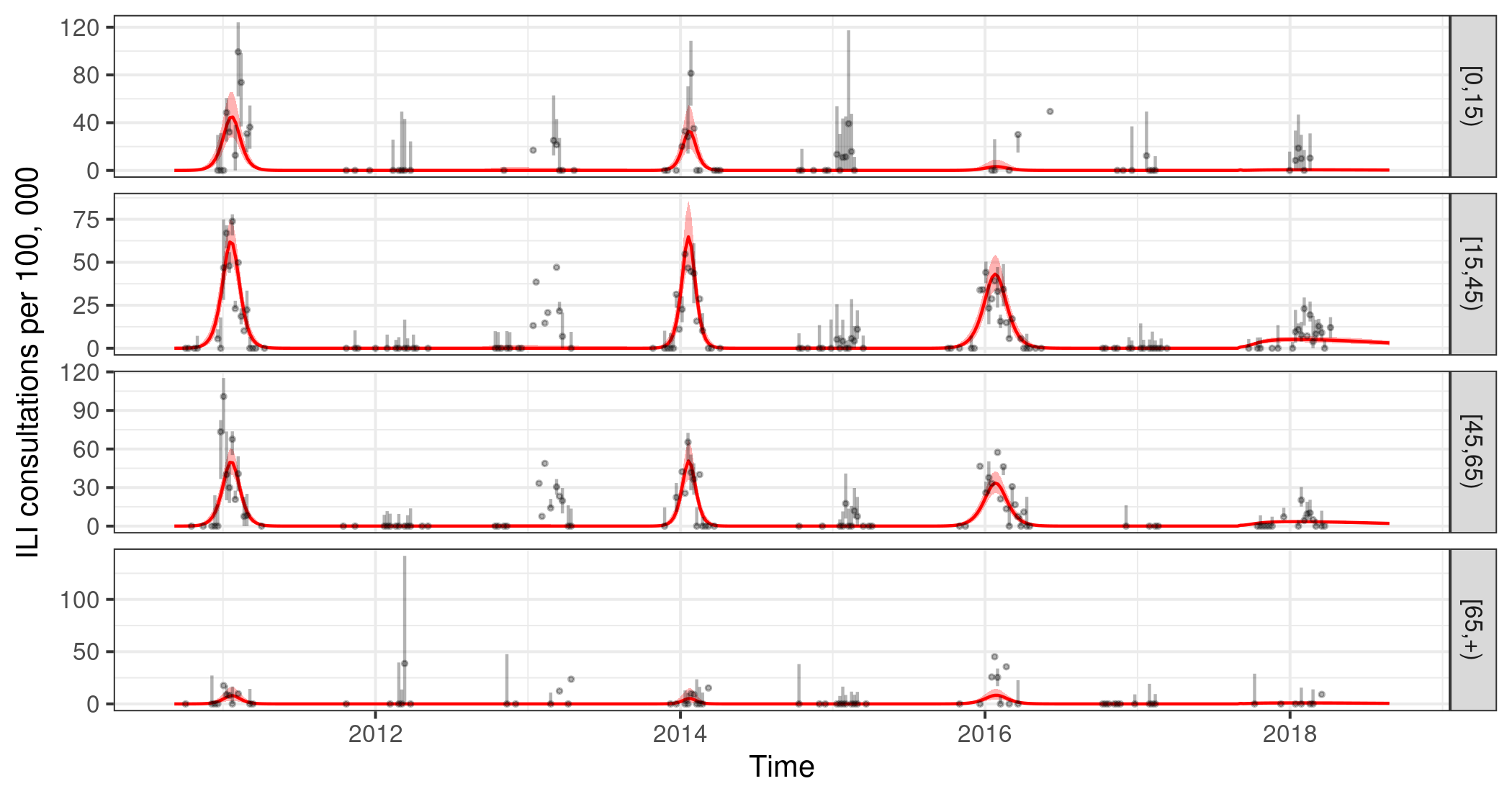


Supplementary Figure 44: Posterior parameter values for PT and influenza virus subtype H3N2. Box plots highlight the median, the 50% CI and the 95% CI. Susceptibility and ascertainment rates are assumed to be different for children, adults and elderly.


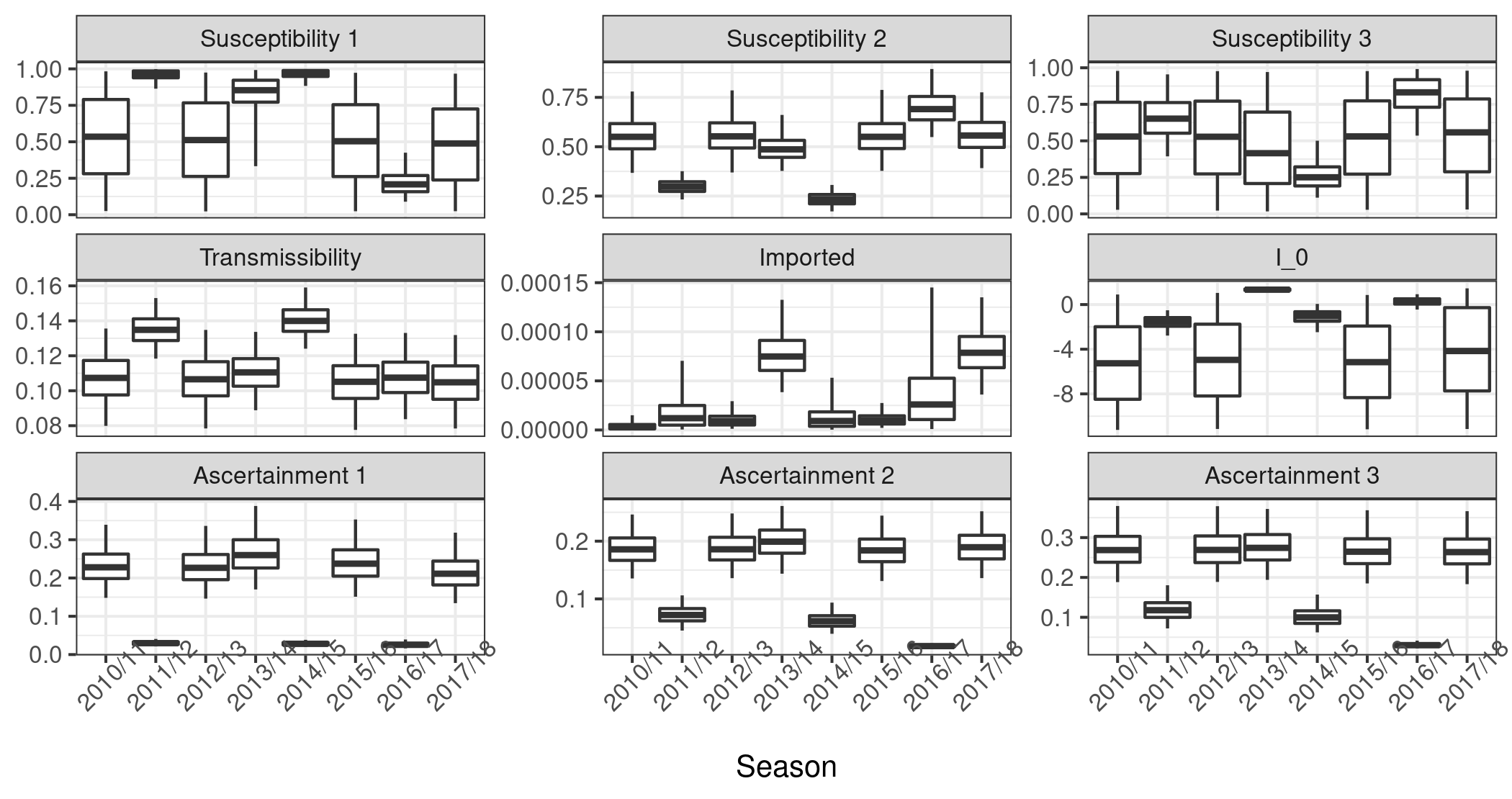


Supplementary Figure 45: Fit of the model to the data for PT and influenza virus subtype H3N2. Red indicates the predicted values, while black shows the data and associated uncertainty (95% CI).


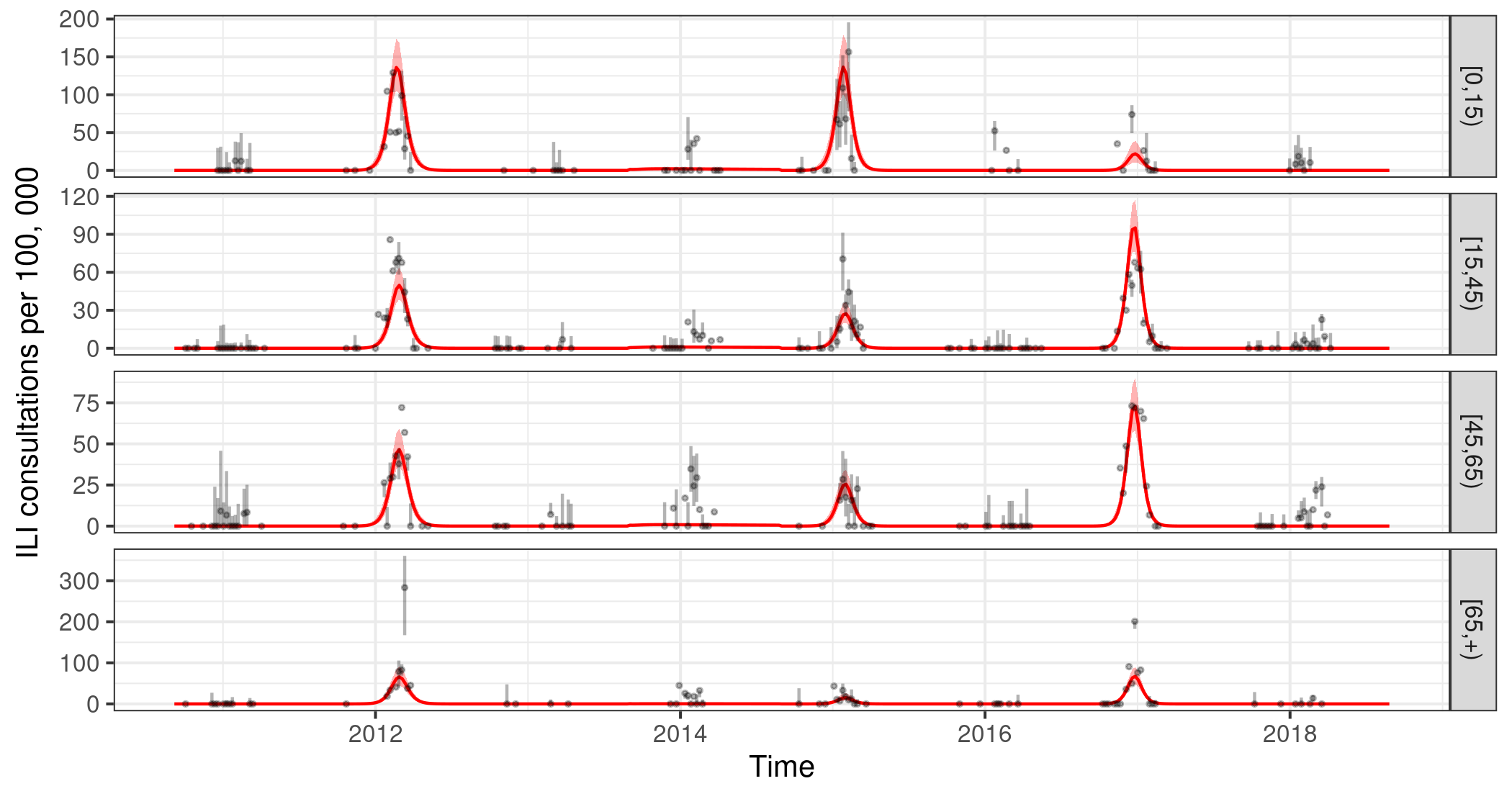


Supplementary Figure 46: Posterior parameter values for SC and influenza virus subtype B. Box plots highlight the median, the 50% CI and the 95% CI. Susceptibility and ascertainment rates are assumed to be different for children, adults and elderly.


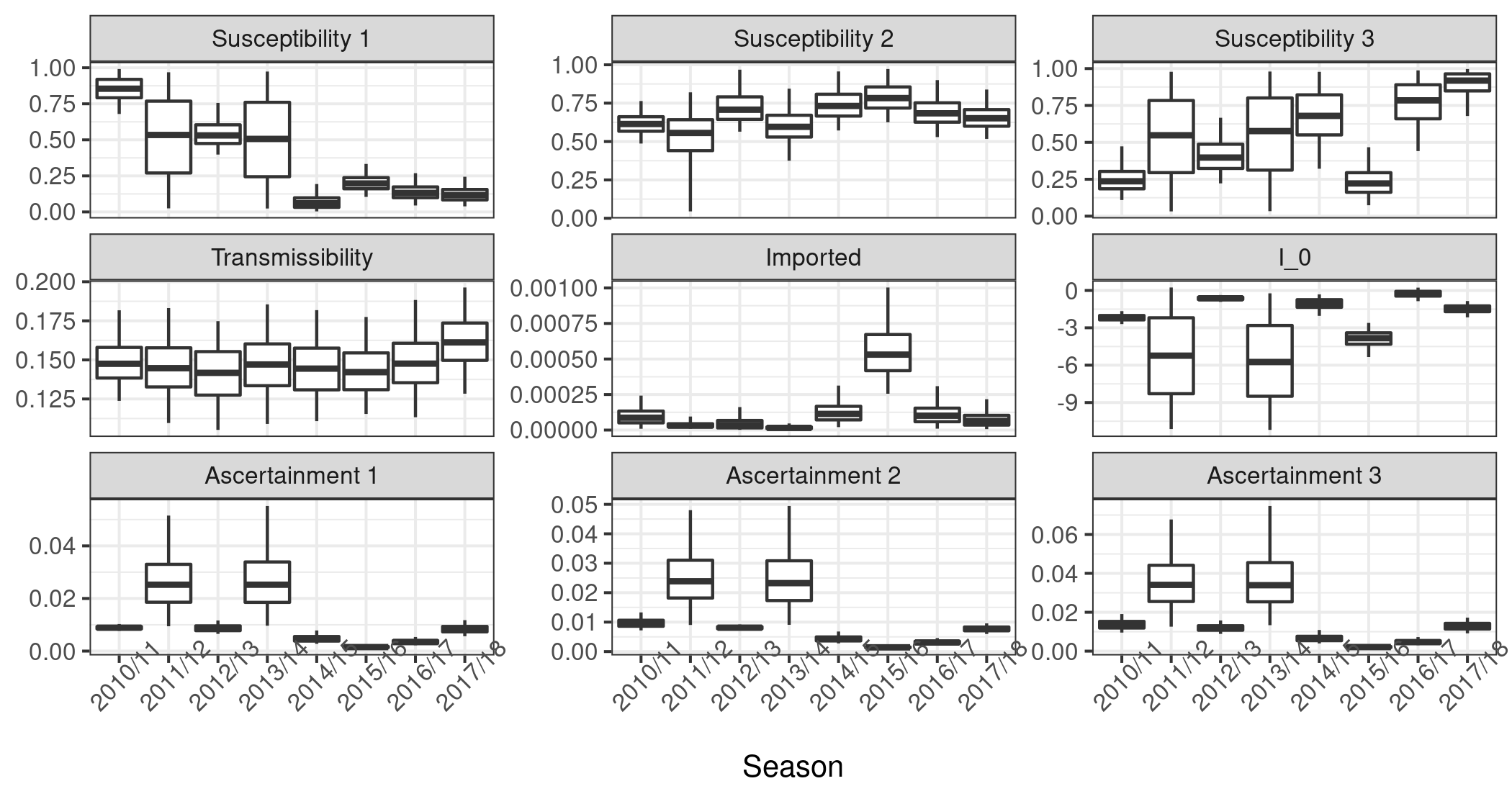


Supplementary Figure 47: Fit of the model to the data for SC and influenza virus subtype B. Red indicates the predicted values, while black shows the data and associated uncertainty (95% CI).


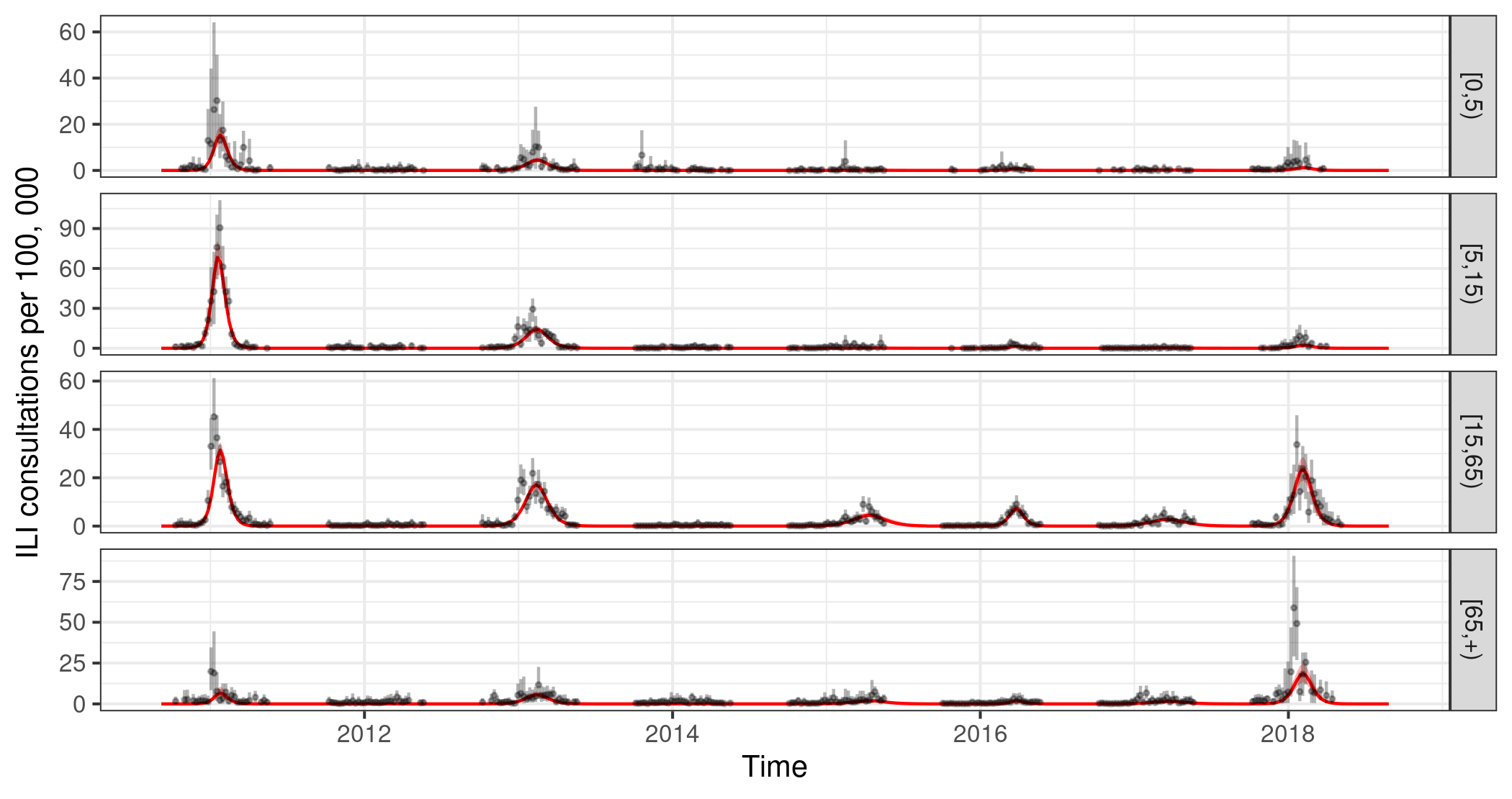


Supplementary Figure 48: Posterior parameter values for SC and influenza virus subtype H1N1. Box plots highlight the median, the 50% CI and the 95% CI. Susceptibility and ascertainment rates are assumed to be different for children, adults and elderly.


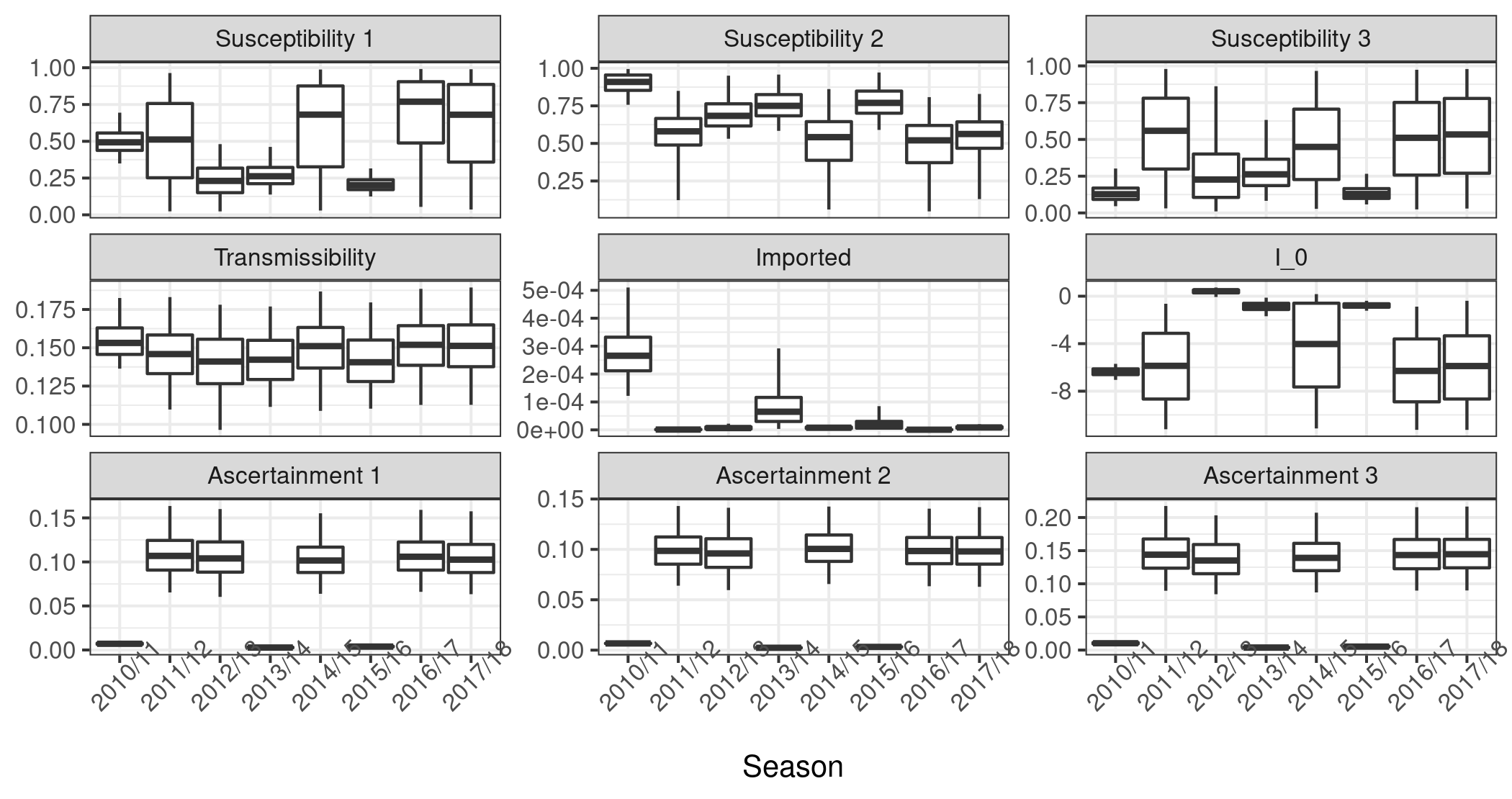


Supplementary Figure 49: Fit of the model to the data for SC and influenza virus subtype H1N1. Red indicates the predicted values, while black shows the data and associated uncertainty (95% CI).


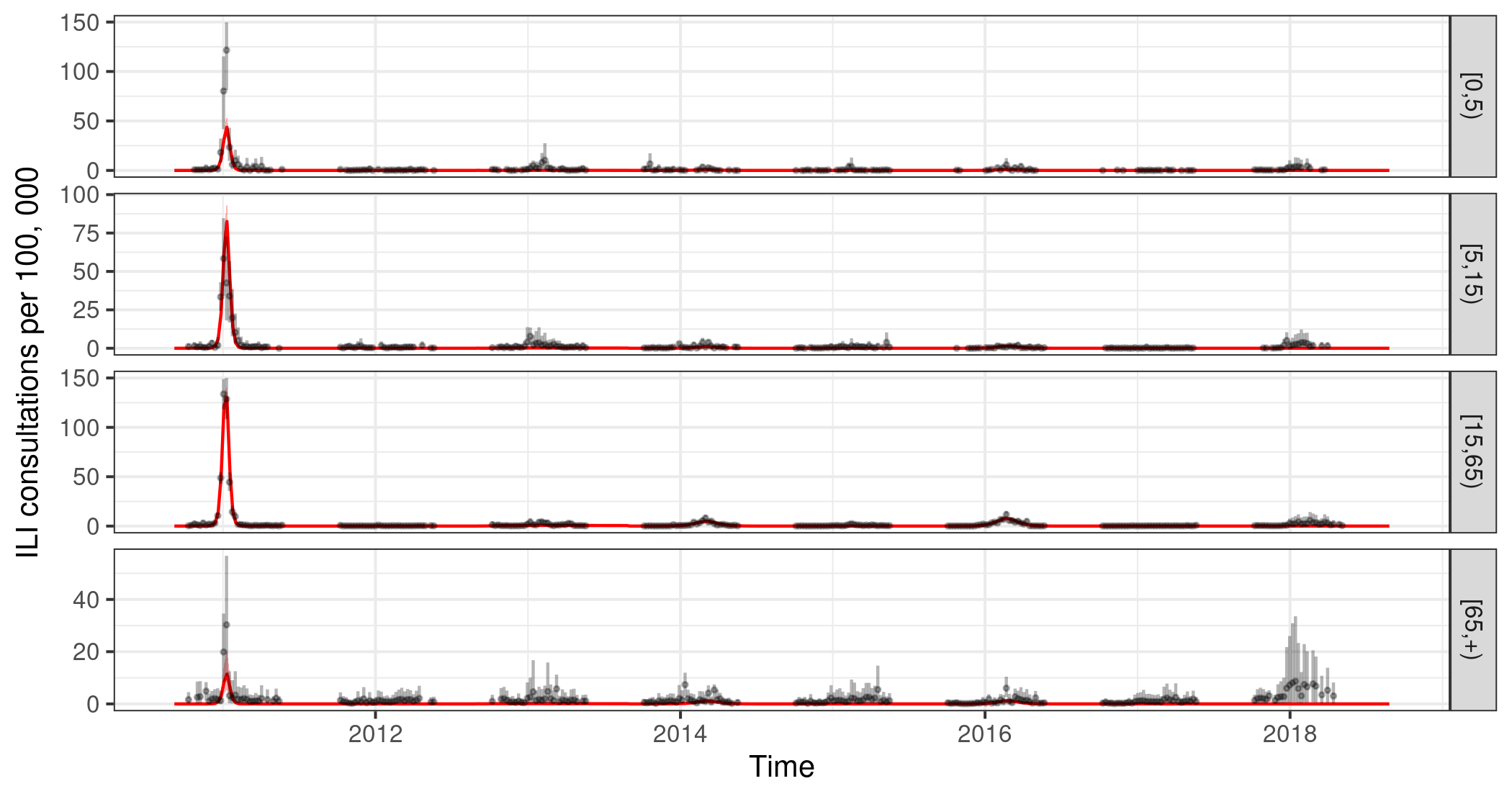


Supplementary Figure 50: Posterior parameter values for SC and influenza virus subtype H3N2. Box plots highlight the median, the 50% CI and the 95% CI. Susceptibility and ascertainment rates are assumed to be different for children, adults and elderly.


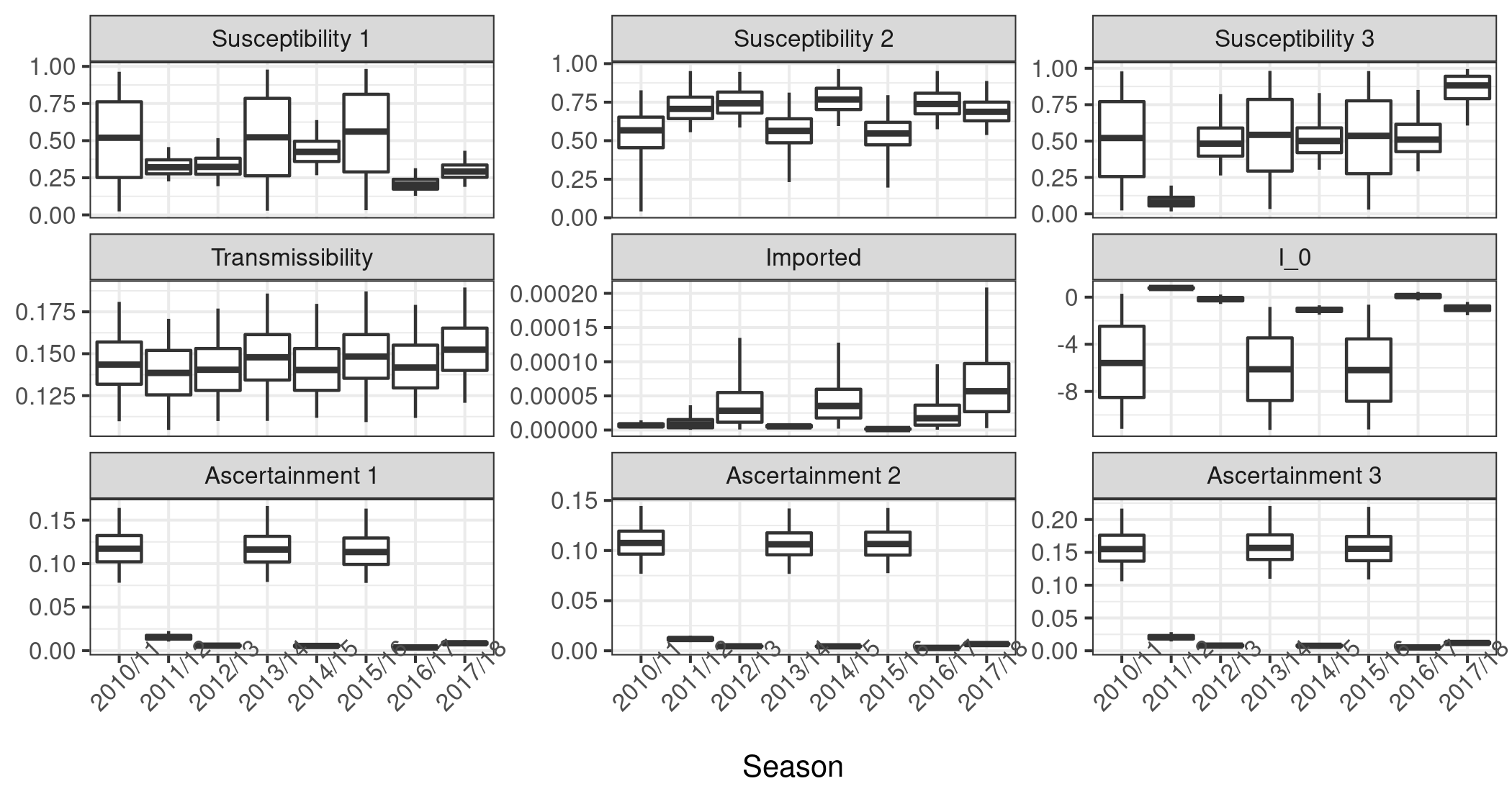


Supplementary Figure 51: Fit of the model to the data for SC and influenza virus subtype H3N2. Red indicates the predicted values, while black shows the data and associated uncertainty (95% CI).


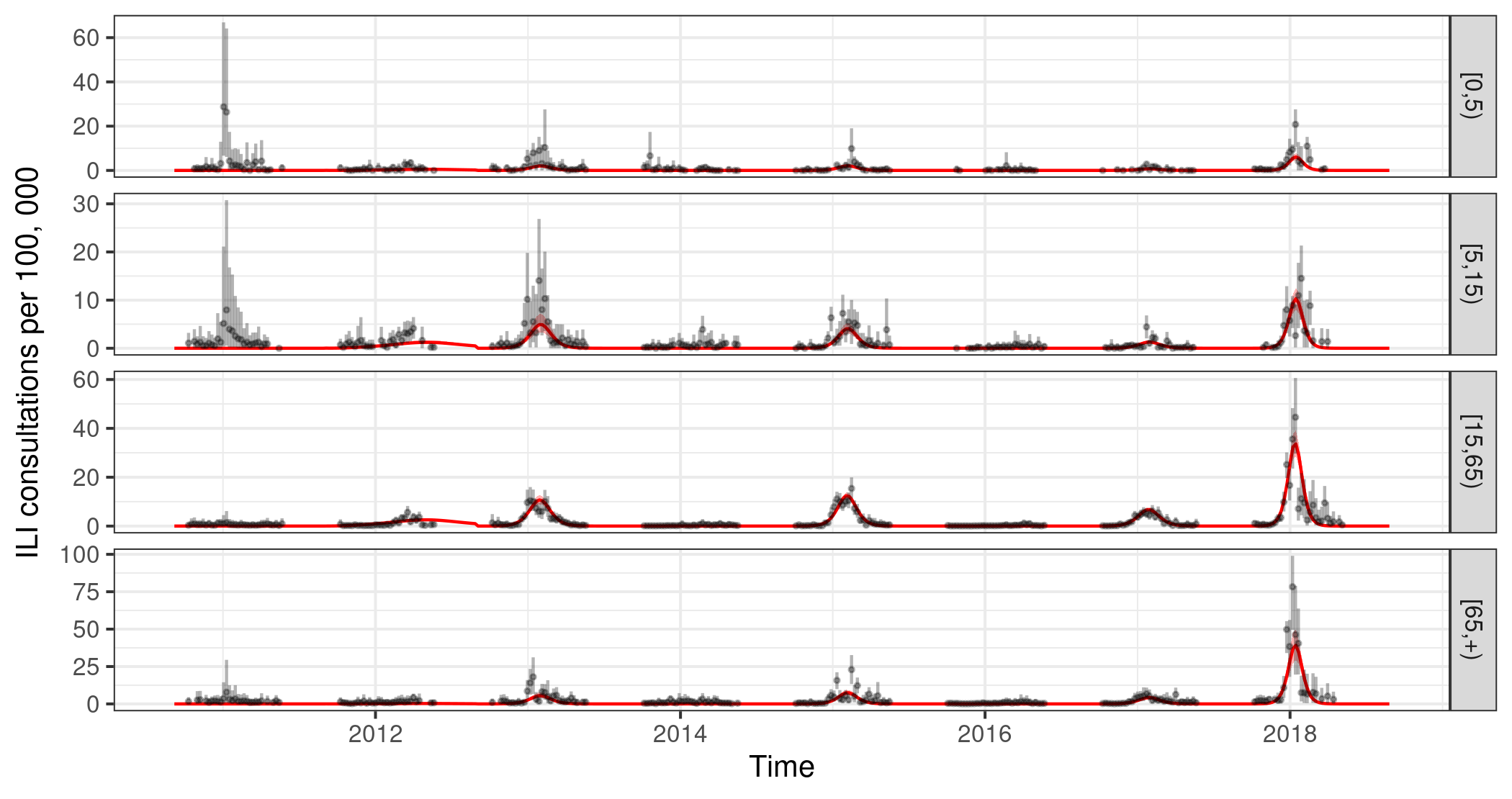


Estimated sample sizes for the inference mixing by setting, season, and influenza virus subtype

We excluded from the further analysis the inference results of ten seasons per strain based on poor visual model fit (for influenza A(H1N1) in Portugal 2017/2018 and influenza A(H3N2) in Ireland 2010/2011) and effective sample sizes below 100 (Supplementary Table 5): Influenza A(H1N1) in Ireland (2012/2013); influenza A(H3N2) in France (2015/2016), Ireland (2013/2014), Scotland (2011/2012) and England (2017/2018); and influenza type B in Portugal (2015/2016), Spain (2012/2013), and Navarra (2016/2017). Results changed only slightly for the settings involved.

| Supplementary Table 5: Estimated sample sizes for the inference. Values shown here are a combination of the ESS of the likelihood/minimal ESS of the parameters, i.e. the maximum of these values is shown. | | | | | | | | | |
| --- | --- | --- | --- | --- | --- | --- | --- | --- | --- |
| Setting | Subtype | 2010/11 | 2011/12 | 2012/13 | 2013/14 | 2014/15 | 2015/16 | 2016/17 | 2017/18 |
| EN | B | 400 | 115 | 1125 | 4428 | 947 | 772 | 2546 | 663 |
| EN | H1N1 | 1127 | 5000 | 816 | 2799 | 455 | 512 | 2902 | 1345 |
| EN | H3N2 | 4223 | 203 | 2905 | 1033 | 249 | 3188 | 2479 | 54 |
| ES | B | 233 | 654 | 88 | 3998 | 327 | 131 | 1734 | 295 |
| ES | H1N1 | 284 | 2053 | 196 | 210 | 177 | 185 | 3401 | 447 |
| ES | H3N2 | 414 | 188 | 844 | 392 | 277 | 120 | 262 | 229 |
| FR | B | n/a | n/a | n/a | n/a | 528 | 222 | 3324 | 614 |
| FR | H1N1 | n/a | n/a | n/a | n/a | 402 | 371 | 3760 | 377 |
| FR | H3N2 | n/a | n/a | n/a | n/a | 276 | 33 | 228 | 1202 |
| IE | B | 627 | 1248 | 3223 | 3482 | 4469 | 2980 | 3379 | 2778 |
| IE | H1N1 | 818 | 2129 | 19 | 2873 | 115 | 723 | 5000 | 4018 |
| IE | H3N2 | 240 | 826 | 2642 | 20 | 318 | 1953 | 2392 | 1995 |
| NL | B | 810 | 4760 | 1950 | 5000 | 1119 | 615 | 4710 | 825 |
| NL | H1N1 | 1884 | 1207 | 2600 | 4448 | 1706 | 212 | 5000 | 2668 |
| NL | H3N2 | 5000 | 1420 | 1615 | 1374 | 1022 | 4478 | 687 | 1414 |
| NV | B | 438 | 3099 | 272 | 4198 | 521 | 869 | 84 | 712 |
| NV | H1N1 | 335 | 5000 | 2475 | 982 | 4592 | 398 | 4782 | 1691 |
| NV | H3N2 | 987 | 309 | 432 | 644 | 271 | 3715 | 684 | 702 |
| PT | B | 3600 | 4066 | 5447 | 3156 | 4600 | 11 | 5000 | 3176 |
| PT | H1N1 | 4103 | 3891 | 1563 | 4394 | 2137 | 3935 | 5000 | 1342 |
| PT | H3N2 | 4456 | 2834 | 4069 | 481 | 2944 | 4476 | 4789 | 3682 |
| SC | B | 324 | 2472 | 459 | 4457 | 1050 | 1800 | 1645 | 1487 |
| SC | H1N1 | 266 | 1301 | 837 | 613 | 169 | 222 | 2371 | 4247 |
| SC | H3N2 | 1531 | 51 | 824 | 3726 | 685 | 1958 | 786 | 599 |
| EN: England, ES: Spain, ESS: effective sample size, FR: France, IE: Ireland, n/a: not available, NL: Netherlands, NV: Navarra, PT: Portugal, SC: Scotland. | | | | | | | | | |

Spearman rank correlation coefficients between settings based on the incidence by season

| Supplementary Table 6: Spearman rank correlation of the median estimated incidence by season between settings for influenza A(H1N1)pdm09. | | | | | | | | |
| --- | --- | --- | --- | --- | --- | --- | --- | --- |
| Setting | EN | ES | FR | IE | NL | NV | PT | SC |
| EN |  |  |  |  |  |  |  |  |
| ES | **0.79*** |  |  |  |  |  |  |  |
| FR | 0.40 | 0.80 |  |  |  |  |  |  |
| IE | **0.93***** | **0.88**** | 0.80 |  |  |  |  |  |
| NL | 0.62 | 0.38 | 0.40 | 0.64 |  |  |  |  |
| NV | **0.98****** | **0.86**** | 0.40 | **0.90**** | 0.48 |  |  |  |
| PT | **0.90**** | **0.86**** | 0.40 | **0.83*** | 0.33 | **0.95***** |  |  |
| SC | **0.93***** | **0.88**** | 0.80 | **1.00****** | 0.64 | **0.90**** | **0.83*** |  |
| EN: England, ES: Spain, FR: France, IE: Ireland, NL: Netherlands, NV: Navarra, PT: Portugal, SC: Scotland.  *: p < .05  **: p < .01  ***: p < .001  ****: p < .0001 | | | | | | | | |

| Supplementary Table 7: Spearman rank correlation of the median estimated incidence by season between settings for influenza A(H3N2). | | | | | | | | |
| --- | --- | --- | --- | --- | --- | --- | --- | --- |
| Setting | EN | ES | FR | IE | NL | NV | PT | SC |
| EN |  |  |  |  |  |  |  |  |
| ES | **0.98****** |  |  |  |  |  |  |  |
| FR | **1.00**** | **1.00**** |  |  |  |  |  |  |
| IE | **0.73*** | 0.66 | **1.00***** |  |  |  |  |  |
| NL | **0.99****** | **0.99****** | **1.00***** | **0.74*** |  |  |  |  |
| NV | 0.49 | 0.60 | 0.74 | 0.04 | 0.52 |  |  |  |
| PT | 0.60 | 0.65 | 0.70 | 0.52 | 0.64 | 0.69 |  |  |
| SC | **0.84**** | **0.73*** | **0.99**** | 0.55 | **0.77*** | 0.25 | 0.37 |  |
| EN: England, ES: Spain, FR: France, IE: Ireland, NL: Netherlands, NV: Navarra, PT: Portugal, SC: Scotland.  *: p < .05  **: p < .01  ***: p < .001  ****: p < .0001 | | | | | | | | |

| Supplementary Table 8: Spearman rank correlation of the median estimated incidence by season between settings for the B subtype. | | | | | | | | |
| --- | --- | --- | --- | --- | --- | --- | --- | --- |
| Setting | EN | ES | FR | IE | NL | NV | PT | SC |
| EN |  |  |  |  |  |  |  |  |
| ES | 0.65 |  |  |  |  |  |  |  |
| FR | **1.00***** | **1.00**** |  |  |  |  |  |  |
| IE | **0.93***** | 0.47 | **1.00**** |  |  |  |  |  |
| NL | **0.94***** | 0.50 | **1.00***** | **1.00****** |  |  |  |  |
| NV | 0.45 | **0.94***** | **1.00***** | 0.27 | 0.31 |  |  |  |
| PT | **0.95***** | 0.54 | **1.00**** | **0.99****** | **1.00****** | 0.33 |  |  |
| SC | 0.58 | -0.19 | 0.60 | **0.78*** | **0.75*** | -0.37 | **0.73*** |  |
| EN: England, ES: Spain, FR: France, IE: Ireland, NL: Netherlands, NV: Navarra, PT: Portugal, SC: Scotland.  *: p < .05  **: p < .01  ***: p < .001  ****: p < .0001 | | | | | | | | |

Intermediate base case results per setting

Supplementary Figure 52. Base-case values per setting for the rates of events per 100,000 population (mean and 95% uncertainty interval).


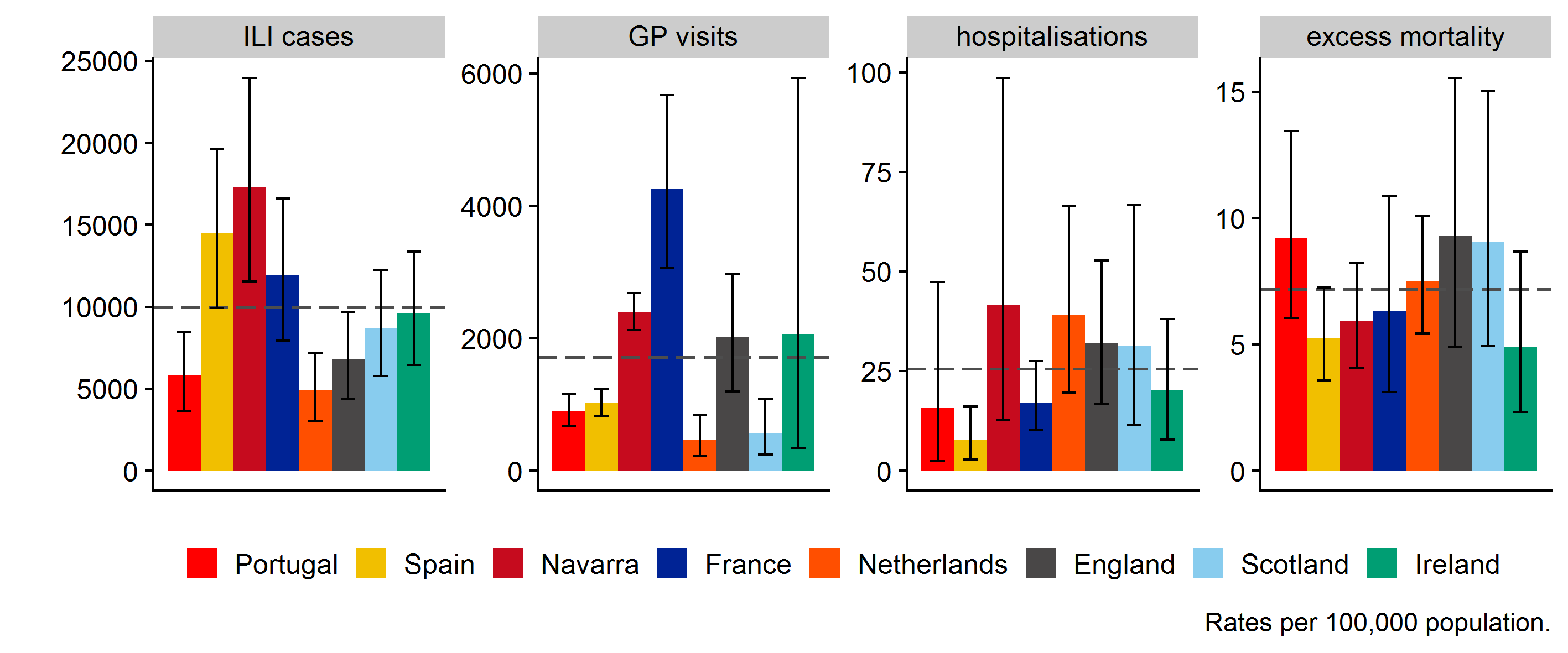


| Supplementary Table 9: Base-case values for the rates of events per 100,000 population by setting, (mean and 95% uncertainty interval). | | | | | | | | |
| --- | --- | --- | --- | --- | --- | --- | --- | --- |
| **natural outcomes** | **PT** | **ES** | **NV** | **FR** | **NL** | **EN** | **SC** | **IE** |
| ILI cases | 5822.24 (3607.40, 8473.74) | 14476.88 (9920.07, 19619.42) | 17269.23 (11550.85, 23964.80) | 11954.62 (7946.38, 16597.30) | 4898.57 (3030.91, 7173.18) | 6813.67 (4389.02, 9689.78) | 8720.07 (5768.81, 12227.44) | 9624.00 (6438.65, 13367.07) |
| GP visits | 901.30 (667.40, 1157.43) | 1019.17 (824.36, 1230.25) | 2399.32 (2124.10, 2684.62) | 4260.65 (3057.76, 5676.70) | 470.07 (222.89, 841.40) | 2010.69 (1198.60, 2967.44) | 559.43 (240.02, 1076.66) | 2064.44 (343.07, 5935.30) |
| hospitalisations | 15.64 (2.31, 47.40) | 7.71  (2.78, 16.11) | 41.57 (12.83, 98.62) | 16.96 (10.21, 27.52) | 39.07 (19.62, 66.39) | 31.99 (16.74, 52.73) | 31.39 (11.54, 66.69) | 20.14 (7.84, 37.99) |
| excess mortality | 9.21 (6.04, 13.43) | 5.23  (3.56, 7.24) | 5.91  (4.05, 8.22) | 6.31 (3.11, 10.87) | 7.51 (5.42, 10.10) | 9.31 (4.91, 15.55) | 9.05 (4.93, 15.03) | 4.91 (2.32, 8.68) |
| EN: England, ES: Spain, FR: France, GP: general practitioner, IE: Ireland, ILI: influenza-like illness, NL: Netherlands, NV: Navarra, PT: Portugal, SC: Scotland. | | | | | | | | |

Supplementary Figure 53. Base-case values per setting and by age groups for the rates of events per 100,000 population (mean and 95% uncertainty interval).


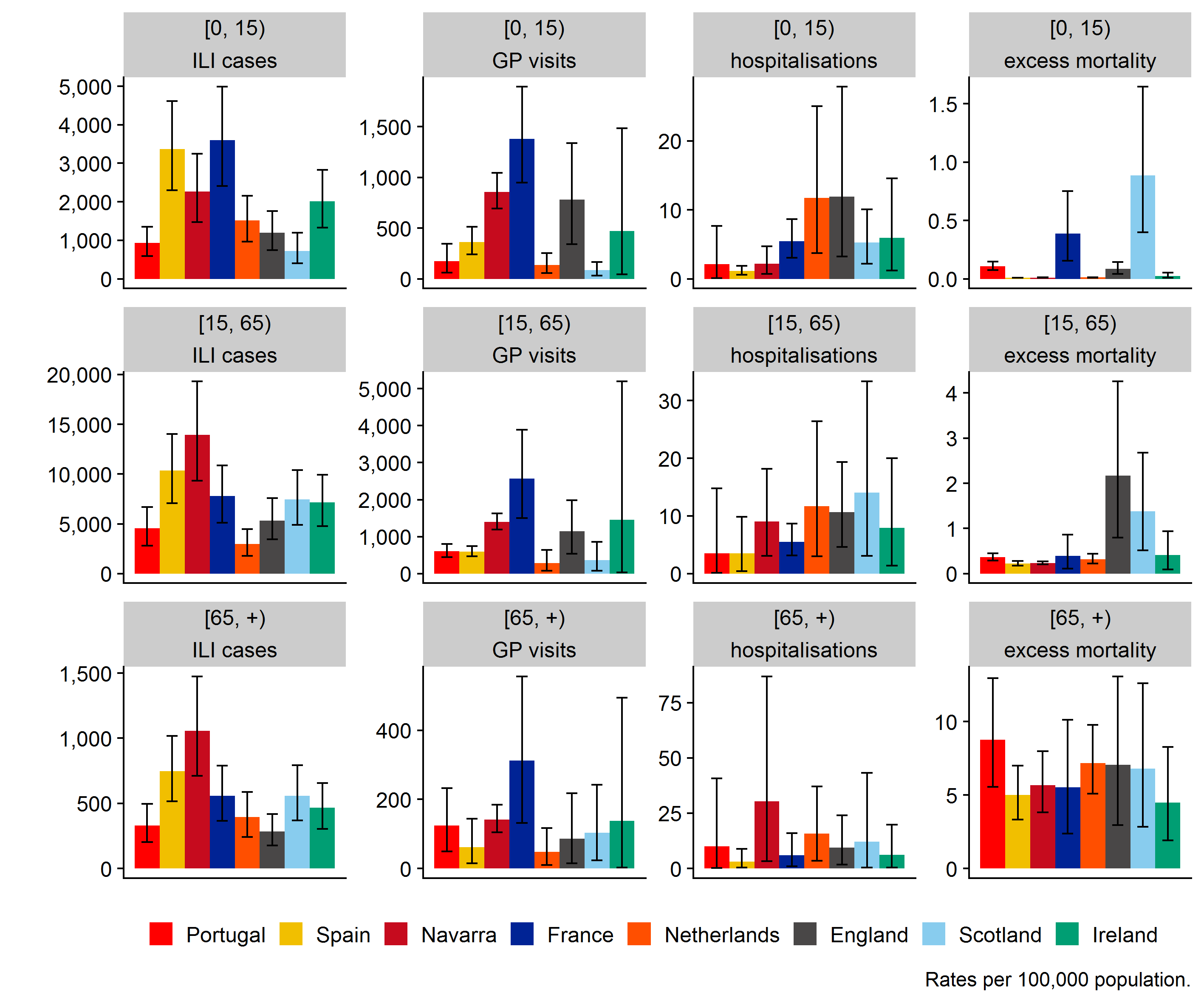


The age-stratified values show the largest burden of symptomatic ILI cases and GP visits for adults aged 15-64 years, while the largest burden of hospitalisations and excess deaths is in the elderly.

The figure shows the differences of the intermediate base-case values across settings, which reflects the variability between settings in terms of influenza epidemiology, healthcare seeking behaviour, level of detail of surveillance, severity of hospitalised influenza cases, resource organisation of healthcare systems, and a possible link with climate and antibiotic consumption.^51,52^

Higher base-case values make the programme more cost-effective if more disease can be prevented.

| Supplementary Table 10: Base-case results for the rates of events per 100,000 population by setting and age group (mean and 95% uncertainty interval). | | | | | | | | | |
| --- | --- | --- | --- | --- | --- | --- | --- | --- | --- |
| **natural outcomes** | **Age group** | **PT** | **ES** | **NV** | **FR** | **NL** | **EN** | **SC** | **IE** |
| ILI cases | [0, 15) | 935.01 (595.39, 1354.09) | 3371.56 (2299.70, 4611.21) | 2270.55 (1477.85, 3247.03) | 3596.87 (2414.43, 4986.97) | 1512.19 (970.65, 2154.81) | 1201.62 (749.31, 1764.68) | 724.01 (404.59, 1192.58) | 2018.21 (1330.52, 2832.97) |
|  | [15, 65) | 4557.57 (2803.18, 6695.17) | 10357.95 (7077.19, 14031.04) | 13940.96 (9328.32, 19292.78) | 7801.02 (5124.20, 10886.94) | 2991.59 (1802.66, 4470.54) | 5329.71 (3455.17, 7584.12) | 7436.77 (4901.39, 10407.51) | 7138.30 (4759.67, 9931.28) |
|  | [65, +) | 329.67 (200.78, 494.76) | 747.37 (516.11, 1018.50) | 1057.72 (711.44, 1475.74) | 556.73 (363.80, 790.52) | 394.79 (240.73, 587.59) | 282.34 (175.21, 416.10) | 559.29 (367.72, 794.07) | 467.49 (304.96, 655.72) |
| GP visits | [0, 15) | 172.72 (59.08, 345.78) | 364.41 (240.71, 514.31) | 857.59 (694.77, 1043.50) | 1381.76 (948.00, 1894.16) | 135.55 (54.77, 251.56) | 780.45 (343.22, 1339.12) | 85.78 (31.99, 166.48) | 472.79 (42.35, 1483.00) |
|  | [15, 65) | 604.68 (439.89, 800.61) | 592.74 (462.81, 739.07) | 1400.17 (1189.03, 1624.48) | 2566.40 (1496.91, 3890.97) | 286.07 (74.09, 641.76) | 1143.78 (531.42, 1986.81) | 370.15 (83.45, 863.88) | 1453.53 (31.57, 5190.86) |
|  | [65, +) | 123.90 (49.16, 232.57) | 62.02 (14.29, 144.10) | 141.56 (104.68, 184.35) | 312.49 (131.42, 556.43) | 48.45 (10.00, 117.01) | 86.46 (14.69, 217.50) | 103.50 (22.79, 242.62) | 138.13 (2.83, 494.12) |
| Hospitali-sations | [0, 15) | 2.12 (0.07, 7.66) | 1.15 (0.60, 1.91) | 2.20 (0.70, 4.71) | 5.45 (3.03, 8.65) | 11.73 (3.76, 25.03) | 11.93 (3.26, 27.86) | 5.25 (2.20, 10.10) | 5.92 (1.19, 14.58) |
|  | [15, 65) | 3.49 (0.11, 14.75) | 3.54 (0.44, 9.83) | 9.07 (3.11, 18.20) | 5.53 (3.12, 8.64) | 11.66 (2.98, 26.44) | 10.66 (4.64, 19.32) | 14.06 (3.07, 33.29) | 7.97 (1.42, 19.98) |
|  | [65, +) | 10.03 (0.12, 40.66) | 3.02 (0.30, 8.85) | 30.30 (3.35, 86.90) | 5.98 (0.88, 16.01) | 15.68 (3.55, 37.01) | 9.40 (1.66, 24.00) | 12.07 (0.35, 43.29) | 6.24 (0.44, 19.76) |
| excess mortality | [0, 15) | 0.11 (0.08, 0.15) | 0.01 (0.01, 0.01) | 0.01 (0.01, 0.01) | 0.39 (0.15, 0.75) | 0.01 (0.01, 0.01) | 0.09 (0.04, 0.14) | 0.89 (0.40, 1.65) | 0.03 (0.01, 0.05) |
|  | [15, 65) | 0.36 (0.29, 0.45) | 0.23 (0.18, 0.28) | 0.23 (0.20, 0.27) | 0.39 (0.11, 0.86) | 0.32 (0.22, 0.44) | 2.17 (0.80, 4.25) | 1.38 (0.51, 2.68) | 0.41 (0.10, 0.94) |
|  | [65, +) | 8.74 (5.56, 12.94) | 4.99 (3.33, 6.99) | 5.67 (3.81, 7.97) | 5.53 (2.36, 10.11) | 7.18 (5.09, 9.77) | 7.05 (2.93, 13.07) | 6.78 (2.84, 12.61) | 4.48 (1.92, 8.27) |
| EN: England, ES: Spain, FR: France, GP: general practitioner, IE: Ireland, ILI: influenza-like illness, NL: Netherlands, NV: Navarra, PT: Portugal, SC: Scotland. | | | | | | | | | |

Relative reductions in infections for the elderly per setting


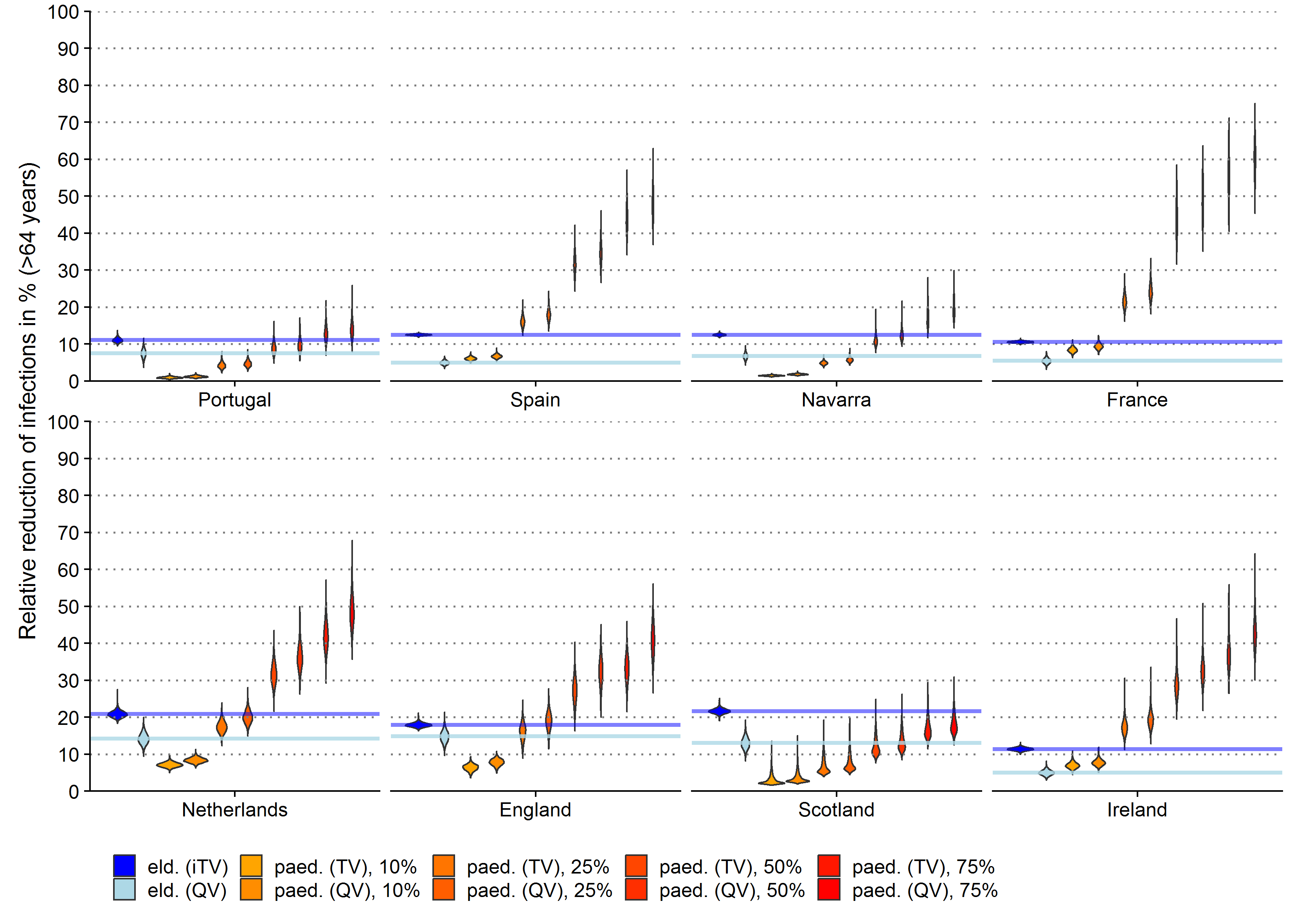
Supplementary Figure 54. Distributions of the relative reduction in infections for the elderly by setting, comparing only non-combined programmes (i.e., moving the elderly to an improved vaccine or adopting mass paediatric vaccination).

The low relative reductions seen in some settings with a paediatric vaccination programme at 10% (Supplementary Figure 54) uptake may partly be explained with the data indicating that most settings vaccinate already a certain proportion of children (e.g. high-risk groups; see Supplementary Table 2).

Relative reductions in infections across ages per setting

Detailed changes in the relative reduction of infections as compared to the base case per setting (complementary to Figure 2 in the main text).

| Supplementary Table 11: Mean (95%-CrI) reduction of influenza virus infections per 100,000 population across vaccination strategies and settings by age groups of children and adolescents (0-14 years), adults (15-64 years), and the elderly (65+ years). | | | | | | |
| --- | --- | --- | --- | --- | --- | --- |
| **setting** | **Portugal (PT)** | | | **Spain (ES)** | | |
| **vaccination strategy / age group** | **[0, 15)** | **[15, 65)** | **[65, +)** | **[0, 15)** | **[15, 65)** | **[65, +)** |
| elderly (iTV) | 25 (17.7, 33.9) | 201.3 (152.7, 257.7) | 154.4 (114.4, 201.1) | 91.9 (81, 104.1) | 414.1 (360.8, 473.4) | 304 (268.3, 343.6) |
| elderly (QV) | 19.1 (10.9, 30.5) | 128.5 (78.1, 188.9) | 105.5 (61.6, 163.2) | 47.5 (37.2, 60.7) | 197 (145.9, 265.1) | 120.3 (95.1, 150.6) |
| paed. (TV), 10% | 178.7 (155.7, 208.1) | 122.2 (86.8, 162.9) | 12.6 (7.5, 19.4) | 1279.1 (1196.3, 1374) | 1695.5 (1569.7, 1832.8) | 147.6 (126.6, 170.3) |
| paed. (TV), 25% | 812.2 (711.2, 926.6) | 648 (507.9, 803.3) | 58.9 (40.8, 83.1) | 3256.9 (3006.4, 3535.4) | 4477.7 (4102.1, 4909.1) | 392.2 (329.8, 460.8) |
| paed. (TV), 50% | 1558.2 (1306.2, 1836.5) | 1430.1 (1113, 1797.7) | 123.7 (87.5, 170.8) | 5608.8 (5196.5, 6053.9) | 8676 (7808.4, 9696.6) | 771.4 (648.3, 917.1) |
| paed. (TV), 75% | 2066.2 (1717, 2453.1) | 2100.9 (1627.2, 2665.3) | 179.2 (128, 244.1) | 7110.7 (6624.1, 7658.4) | 12168.6 (10801.6, 13812.2) | 1071.1 (894.5, 1280.7) |
| paed. (QV), 10% | 221.3 (189.5, 259.3) | 181.6 (140, 229.9) | 16.3 (10.4, 24.3) | 1446.5 (1354.6, 1560) | 1881.9 (1736.2, 2048.5) | 163.3 (140.1, 189.2) |
| paed. (QV), 25% | 895.8 (777, 1029.7) | 755 (598.1, 932.7) | 65.7 (45.6, 92.3) | 3672.2 (3394.9, 3984.2) | 5001.7 (4541.9, 5529.5) | 437 (367.1, 514.2) |
| paed. (QV), 50% | 1704.9 (1428.4, 2019) | 1619.4 (1260.5, 2035.6) | 135.5 (95.1, 187.9) | 6345.4 (5913.2, 6814.9) | 9561.5 (8610.8, 10685.2) | 843.8 (712.4, 997) |
| paed. (QV), 75% | 2259.3 (1878.8, 2688.7) | 2363.3 (1831.9, 3007.4) | 195.4 (137.5, 269) | 7890.9 (7391, 8430.5) | 13282.2 (11790.5, 14962.3) | 1165.9 (973.2, 1383.3) |
| eld. (iTV) + paed. (TV), 10% | 203.3 (176.5, 236.5) | 323.4 (262.5, 392.7) | 165.7 (123.8, 214) | 1373.5 (1290.3, 1468.5) | 2121.4 (1981.3, 2264.3) | 437.7 (388.6, 491.1) |
| eld. (iTV) + paed. (TV), 25% | 836 (732.3, 953) | 848.3 (697.3, 1009.5) | 207.5 (157.5, 264.6) | 3360 (3107.1, 3636.9) | 4933 (4550.8, 5359.4) | 660.2 (578.9, 746.2) |
| eld. (iTV) + paed. (TV), 50% | 1578.5 (1324.2, 1859.6) | 1630 (1312, 2006.7) | 266.2 (201.7, 341.3) | 5699.1 (5287.6, 6143.1) | 9130.7 (8266.2, 10161.1) | 999.2 (863.1, 1149) |
| eld. (iTV) + paed. (TV), 75% | 2086.2 (1736.4, 2477) | 2302.7 (1825.7, 2870.5) | 317 (239.1, 408.3) | 7193.4 (6703, 7750.2) | 12655.4 (11267.2, 14317.4) | 1269.7 (1085.9, 1483.1) |
| eld. (QV) + paed. (TV), 10% | 197.5 (169.3, 231.8) | 251.3 (189.9, 323.3) | 117.4 (72.3, 176.6) | 1329.4 (1244.5, 1427.1) | 1903.5 (1767.1, 2046.9) | 260.3 (221.7, 302.5) |
| eld. (QV) + paed. (TV), 25% | 830 (725.1, 948) | 779 (630.5, 943.1) | 161.6 (108.4, 228.9) | 3316.9 (3061.9, 3597.9) | 4718.6 (4337.7, 5141.3) | 492.6 (420.6, 570.3) |
| eld. (QV) + paed. (TV), 50% | 1574.3 (1319.2, 1856) | 1564.5 (1243.4, 1943.2) | 222.7 (155.7, 305.9) | 5651.6 (5241.7, 6094.2) | 8889 (8021.3, 9910.6) | 847.7 (720.4, 995.9) |
| eld. (QV) + paed. (TV), 75% | 2080.3 (1729, 2472.9) | 2238.4 (1759.9, 2816.5) | 274.9 (195.2, 374.2) | 7152.8 (6658.6, 7706.6) | 12400.4 (11022.1, 14053.9) | 1132.3 (953.1, 1341.6) |
| eld. (iTV) + paed. (QV), 10% | 245.8 (210.9, 287.2) | 383 (317.4, 456.1) | 169.2 (126.5, 218.8) | 1541.6 (1447.9, 1656.1) | 2309.6 (2150.8, 2479) | 452.1 (401.7, 507) |
| eld. (iTV) + paed. (QV), 25% | 919.5 (797.9, 1055.7) | 955.8 (788.2, 1138) | 213.8 (161.8, 273.8) | 3773.7 (3494.6, 4085.6) | 5457.9 (4995.3, 5975.5) | 700.9 (613, 794.7) |
| eld. (iTV) + paed. (QV), 50% | 1724.8 (1444.9, 2038.7) | 1820.1 (1460.3, 2238) | 277.2 (209.1, 357) | 6435.7 (6002.4, 6905.4) | 10015.3 (9052.9, 11135.6) | 1064.2 (922.7, 1225.5) |
| eld. (iTV) + paed. (QV), 75% | 2278.6 (1895.5, 2711.6) | 2566.1 (2032.7, 3219) | 332 (248, 430.7) | 7968 (7461.9, 8503.2) | 13765 (12270.6, 15456.8) | 1354.9 (1163.3, 1574) |
| eld. (QV) + paed. (QV), 10% | 240 (203.6, 283.3) | 311.1 (246, 386.6) | 120.6 (74.4, 181.4) | 1498 (1401.9, 1615.5) | 2093.3 (1938.8, 2262) | 273.7 (233.3, 317.7) |
| eld. (QV) + paed. (QV), 25% | 913.3 (791, 1051.3) | 886.8 (722.7, 1073.5) | 167.5 (112.2, 238.4) | 3729 (3449.8, 4038.4) | 5243.1 (4784.5, 5758.6) | 530 (451.4, 613.9) |
| eld. (QV) + paed. (QV), 50% | 1720.3 (1440.5, 2035.1) | 1755.2 (1395, 2177.6) | 233 (162, 323.1) | 6388.4 (5958.3, 6859.2) | 9774.1 (8814.1, 10876.9) | 907.2 (771, 1063.5) |
| eld. (QV) + paed. (QV), 75% | 2272.2 (1889.1, 2704.7) | 2502.7 (1964.9, 3167.4) | 288.9 (203.3, 396.2) | 7922.8 (7422.4, 8456.6) | 13504.9 (12016.4, 15154.4) | 1209.9 (1021.3, 1424.9) |
| CrI: credible interval, eld.: elderly vaccination change (moving from TV to iTV or QV), iTV: improved trivalent vaccine (i.e., adjuvanted or high-dose), paed.: paediatric mass vaccination (scenario with specified vaccine and uptake rate), QV: quadrivalent vaccine (non-adjuvanted, non-high dose), TIV: trivalent vaccine (non-adjuvanted, non-high dose). | | | | | | |

| Supplementary Table 12: Mean (95%-CrI) of the relative reduction of infections per setting and by age groups. | | | | | | |
| --- | --- | --- | --- | --- | --- | --- |
| **setting** | **Navarra (NV)** | | | **France (FR)** | | |
| **vaccination strategy / age group** | **[0, 15)** | **[15, 65)** | **[65, +)** | **[0, 15)** | **[15, 65)** | **[65, +)** |
| elderly (iTV) | 67.8 (57.9, 79) | 497.8 (429.3, 572.4) | 475.7 (412.3, 541.3) | 67.1 (55.1, 81) | 283.9 (234.5, 341.6) | 208.5 (173.7, 250.9) |
| elderly (QV) | 44.4 (33, 57.9) | 252.9 (191.5, 324.8) | 256.1 (188, 334.4) | 42.2 (31.1, 55.9) | 180.4 (131.8, 241.8) | 107.8 (78.8, 144.6) |
| paed. (TV), 10% | 593.7 (529.9, 659.1) | 675.4 (603.8, 752.2) | 54.7 (44.8, 66.4) | 1816.4 (1698.2, 1919.1) | 1934.4 (1795.7, 2073) | 165.8 (138.6, 197) |
| paed. (TV), 25% | 1861.2 (1656.8, 2070.2) | 2339.1 (2108.4, 2589.2) | 186.2 (153.2, 225.2) | 4408.6 (4095.2, 4685.3) | 4957.7 (4557.5, 5364.1) | 425.6 (353.6, 509.5) |
| paed. (TV), 50% | 3687.5 (3238.8, 4220.3) | 5273.6 (4670.8, 6146.3) | 418.5 (338.4, 537.6) | 7821.8 (7156.2, 8484.2) | 10346.9 (8987.3, 11906.8) | 869.9 (699.1, 1087.3) |
| paed. (TV), 75% | 4985.9 (4351.3, 5654.8) | 8227.2 (7103.2, 9633.5) | 655.9 (514.6, 852.7) | 9172.1 (8423.4, 9882) | 13517.5 (11763.3, 15031.7) | 1091.2 (885.1, 1326.8) |
| paed. (QV), 10% | 763.7 (685, 842.6) | 854.3 (766.3, 949.2) | 67.7 (55.2, 82.4) | 2069.3 (1939.8, 2183.1) | 2253 (2099, 2406) | 185.6 (155.9, 219.9) |
| paed. (QV), 25% | 2315.5 (2065.8, 2569) | 2802.7 (2528.3, 3107.9) | 219.4 (179.5, 266.9) | 4976.3 (4624.8, 5311.6) | 5784.6 (5282.8, 6337.2) | 477.9 (396.1, 577.5) |
| paed. (QV), 50% | 4273 (3760.7, 4857.3) | 6127.8 (5435.8, 7107.7) | 478.6 (386.3, 616.8) | 8698.1 (8003.5, 9336.6) | 11941.1 (10430.9, 13446) | 965.9 (776, 1192.4) |
| paed. (QV), 75% | 5819.5 (5095.3, 6559.4) | 9755.5 (8385.6, 11387.4) | 752.5 (595.9, 967.1) | 10305.6 (9530, 11122.2) | 15551.8 (13795.2, 17313) | 1185.8 (971.8, 1429.2) |
| eld. (iTV) + paed. (TV), 10% | 661.1 (594, 730.2) | 1177.3 (1077.6, 1285.6) | 524.9 (456.6, 596) | 1886.4 (1766.3, 1991.1) | 2222.8 (2071.5, 2372.5) | 359.2 (304, 424) |
| eld. (iTV) + paed. (TV), 25% | 1927.5 (1720.4, 2139.2) | 2852 (2607.3, 3112.4) | 643.3 (561.4, 730.5) | 4481 (4163.9, 4760.9) | 5254.8 (4845.7, 5674.8) | 595 (503, 701.5) |
| eld. (iTV) + paed. (TV), 50% | 3752.4 (3298.5, 4292.1) | 5812.6 (5197.4, 6690.3) | 852.7 (737.7, 986.9) | 7886.3 (7216.4, 8543.8) | 10661.5 (9300.1, 12193.7) | 999.7 (820.1, 1225.5) |
| eld. (iTV) + paed. (TV), 75% | 5044.2 (4403.9, 5722.4) | 8786 (7655.8, 10204) | 1065.3 (903.3, 1264.4) | 9219.5 (8470.7, 9927.2) | 13794.9 (12069.2, 15303.8) | 1199.5 (988.1, 1443.6) |
| eld. (QV) + paed. (TV), 10% | 638.1 (569.8, 707.1) | 931.4 (833.1, 1034.5) | 307.3 (236.2, 388.9) | 1861.2 (1741.6, 1965.4) | 2122.7 (1976.1, 2267.4) | 267.3 (221.3, 321.4) |
| eld. (QV) + paed. (TV), 25% | 1905.5 (1699.1, 2117.6) | 2602.3 (2361, 2866.6) | 430.6 (346.1, 520.9) | 4459.1 (4142.2, 4738) | 5166.7 (4761.8, 5577.4) | 517.6 (431.8, 615.6) |
| eld. (QV) + paed. (TV), 50% | 3729.6 (3276.8, 4262.4) | 5550.1 (4932.6, 6431.8) | 649.2 (529.9, 787.9) | 7882.4 (7211.6, 8538.4) | 10619.2 (9259.5, 12120) | 941.8 (767.1, 1158.7) |
| eld. (QV) + paed. (TV), 75% | 5025.5 (4386.5, 5705.6) | 8518.3 (7389.4, 9946.5) | 874.4 (705.2, 1082.5) | 9204.2 (8464.1, 9902) | 13702.8 (11996.3, 15188.6) | 1140.7 (934.7, 1380.7) |
| eld. (iTV) + paed. (QV), 10% | 831.1 (748.5, 913.6) | 1357.4 (1244.2, 1479) | 536.7 (466.7, 609) | 2139.9 (2008.9, 2254.1) | 2543.5 (2381.2, 2707) | 377.4 (319.6, 445.1) |
| eld. (iTV) + paed. (QV), 25% | 2382.1 (2129.1, 2638.3) | 3318.7 (3032.1, 3628.3) | 673.2 (585.8, 765.9) | 5052.3 (4696.7, 5390.9) | 6094.5 (5585.1, 6666.9) | 643.9 (543.5, 764.3) |
| eld. (iTV) + paed. (QV), 50% | 4338.1 (3816.7, 4927.9) | 6675 (5964.9, 7684.9) | 906.9 (779.1, 1059.2) | 8757.9 (8066.8, 9398.1) | 12246.5 (10718.4, 13711.8) | 1086.3 (889.3, 1314.4) |
| eld. (iTV) + paed. (QV), 75% | 5878 (5150.9, 6619) | 10329.3 (8955.2, 11947.3) | 1152.2 (972, 1369.4) | 10358.6 (9583.2, 11177.1) | 15838.8 (14119, 17562.6) | 1283.5 (1062.1, 1532.4) |
| eld. (QV) + paed. (QV), 10% | 808.2 (725.6, 891.3) | 1112.5 (1002.9, 1227.3) | 318 (244.6, 401.7) | 2115.3 (1984.4, 2229.9) | 2445.1 (2284.4, 2604.6) | 284.3 (234.9, 341.4) |
| eld. (QV) + paed. (QV), 25% | 2360.3 (2106.9, 2615.9) | 3071.4 (2783, 3382.7) | 457.9 (366.7, 555.1) | 5033.4 (4674, 5379) | 6016.9 (5505.6, 6592.9) | 563.7 (468.2, 675.8) |
| eld. (QV) + paed. (QV), 50% | 4315.4 (3797.3, 4907.5) | 6419 (5713.3, 7437.7) | 698.7 (568.4, 852.3) | 8750.2 (8062.5, 9385.3) | 12195.7 (10659.7, 13634.1) | 1021 (829.3, 1244.4) |
| eld. (QV) + paed. (QV), 75% | 5859.3 (5131, 6600.4) | 10073.2 (8701.9, 11705.5) | 953.7 (768.4, 1187) | 10348.2 (9579.8, 11166.2) | 15755.1 (14049.5, 17468.8) | 1216.4 (1004.1, 1458.5) |
| CrI: credible interval, eld.: elderly vaccination change (moving from TV to iTV or QV), iTV: improved trivalent vaccine (i.e., adjuvanted or high-dose), paed.: paediatric mass vaccination (scenario with specified vaccine and uptake rate), QV: quadrivalent vaccine (non-adjuvanted, non-high dose), TIV: trivalent vaccine (non-adjuvanted, non-high dose). | | | | | | |

| Supplementary Table 13: Mean (95%-CrI) of the relative reduction of infections per setting and by age groups. | | | | | | |
| --- | --- | --- | --- | --- | --- | --- |
| **setting** | **Netherlands (NL)** | | | **England (EN)** | | |
| **vaccination strategy / age group** | **[0, 15)** | **[15, 65)** | **[65, +)** | **[0, 15)** | **[15, 65)** | **[65, +)** |
| elderly (iTV) | 133.5 (107.2, 163.7) | 556.1 (447.9, 679) | 351.4 (283.4, 428.6) | 92.6 (73.6, 113.8) | 543.1 (434.9, 666.4) | 219 (175.5, 268.4) |
| elderly (QV) | 74.9 (53.8, 101.4) | 407.9 (301, 541.3) | 239.6 (179.6, 317.7) | 72.5 (51.3, 98.7) | 405 (294, 550.2) | 181.4 (132.1, 243) |
| paed. (TV), 10% | 834.9 (757, 915.9) | 994.7 (895.7, 1101.3) | 119.5 (100.3, 140.7) | 724.7 (633.3, 804.5) | 963.8 (844.7, 1073) | 77.7 (56, 98.8) |
| paed. (TV), 25% | 2021.2 (1811.8, 2227.3) | 2517.3 (2245.8, 2792.1) | 292.8 (243.7, 346.7) | 1744 (1500, 1964.2) | 2457.8 (2118.5, 2780.7) | 195.6 (138.4, 253.8) |
| paed. (TV), 50% | 3355.7 (3020.7, 3699.8) | 4471.6 (3810.6, 5224.9) | 534.1 (439.6, 646.3) | 2896.2 (2480.5, 3249.6) | 4547.3 (3949.3, 5183.9) | 330.5 (242.8, 426.6) |
| paed. (TV), 75% | 4060.7 (3685.1, 4485.1) | 5661.7 (4871.8, 6602.9) | 707.9 (586.2, 855.5) | 3472.8 (3038.7, 3861.5) | 5839.1 (5086.9, 6693) | 404.6 (308.1, 511.5) |
| paed. (QV), 10% | 1014.9 (923.8, 1109.4) | 1236.9 (1120, 1359.2) | 140.6 (118.3, 165.2) | 909.2 (806.5, 1005.3) | 1232.9 (1105.5, 1364.4) | 95.1 (71.4, 119.2) |
| paed. (QV), 25% | 2391.2 (2144.7, 2631.9) | 3030.2 (2698.3, 3371.6) | 336.9 (280.8, 401) | 2091.3 (1825.4, 2338.3) | 3014.5 (2652.9, 3370) | 230.7 (168.8, 293.2) |
| paed. (QV), 50% | 3872.8 (3498.8, 4264.4) | 5304 (4521.7, 6191.8) | 610.4 (503.3, 739.9) | 3413.4 (2967.2, 3821.7) | 5580.1 (4875.3, 6347) | 394.8 (294.3, 503.3) |
| paed. (QV), 75% | 4725.1 (4284.3, 5205.5) | 6869.7 (5853.4, 7964.6) | 819.5 (671.6, 996.5) | 4072.4 (3568.2, 4550.1) | 7268.4 (6255.9, 8334.7) | 498.4 (377.1, 635.7) |
| eld. (iTV) + paed. (TV), 10% | 963.4 (879.3, 1053.4) | 1541 (1389.3, 1710.9) | 448.7 (370.6, 534.7) | 816.6 (718.4, 903) | 1509.9 (1359.9, 1680.7) | 285.5 (232.8, 344) |
| eld. (iTV) + paed. (TV), 25% | 2142.7 (1932, 2356.3) | 3049.4 (2754.2, 3365.6) | 589.4 (492.5, 697.1) | 1831.4 (1581, 2058.9) | 3003.9 (2663.3, 3367.1) | 385.4 (310.3, 467.3) |
| eld. (iTV) + paed. (TV), 50% | 3453.3 (3111.6, 3806.6) | 4967.1 (4288.1, 5751.4) | 782.5 (655.1, 929.3) | 2963.1 (2545.6, 3318.7) | 5064.1 (4480.7, 5714.4) | 496.6 (397.6, 605.9) |
| eld. (iTV) + paed. (TV), 75% | 4142.2 (3755.4, 4571.1) | 6150 (5341.5, 7113.8) | 922.7 (772.9, 1096.4) | 3523.6 (3090.8, 3915.5) | 6336.2 (5567.1, 7202.9) | 558.5 (449, 680.2) |
| eld. (QV) + paed. (TV), 10% | 911.2 (827.6, 997.6) | 1413.8 (1264.5, 1586.5) | 352.6 (285.4, 435) | 797.2 (699.6, 885.2) | 1374.2 (1209, 1571.5) | 253.8 (197.1, 321.2) |
| eld. (QV) + paed. (TV), 25% | 2099.8 (1886.5, 2309.6) | 2955.6 (2658, 3262.3) | 516 (429.1, 616.9) | 1816.7 (1569.2, 2041.4) | 2876.8 (2512.1, 3250.8) | 363.2 (282.3, 451.6) |
| eld. (QV) + paed. (TV), 50% | 3428.3 (3086.6, 3779.9) | 4936.9 (4256.2, 5715) | 741.9 (616.5, 886.9) | 2966.1 (2547, 3319.2) | 4976.2 (4362, 5635.4) | 482.5 (375.2, 600.8) |
| eld. (QV) + paed. (TV), 75% | 4133 (3745.3, 4573.1) | 6185.3 (5349.6, 7176.4) | 908.4 (756.1, 1079.6) | 3527 (3094.9, 3920.4) | 6249.2 (5474.1, 7126.6) | 545 (429.4, 672) |
| eld. (iTV) + paed. (QV), 10% | 1144.1 (1047.8, 1244.6) | 1786.3 (1623.1, 1967.5) | 466.9 (386.2, 556) | 1001.4 (892.3, 1104.8) | 1782.1 (1622.4, 1968.4) | 300.5 (244.8, 362.3) |
| eld. (iTV) + paed. (QV), 25% | 2514.6 (2264.4, 2764) | 3570.4 (3215.2, 3946.2) | 627.5 (525.8, 742.5) | 2179.5 (1907.5, 2433.2) | 3567.8 (3200.9, 3967.7) | 415.5 (336.5, 502.8) |
| eld. (iTV) + paed. (QV), 50% | 3973.6 (3598.2, 4370.5) | 5824.7 (5019, 6747.9) | 849.2 (709.7, 1010.9) | 3483 (3034.9, 3905.1) | 6119.1 (5417.6, 6918.1) | 552.1 (441.1, 675.3) |
| eld. (iTV) + paed. (QV), 75% | 4806.4 (4358.5, 5291.5) | 7382.6 (6356, 8497.5) | 1019.3 (848.1, 1215.9) | 4128.5 (3623.2, 4603.6) | 7808 (6767.5, 8896.5) | 639.2 (506.5, 786.3) |
| eld. (QV) + paed. (QV), 10% | 1092.5 (996.8, 1193) | 1661.6 (1496.1, 1842.7) | 368.5 (298, 455.8) | 982.3 (874.9, 1086.9) | 1648.9 (1474.7, 1862.5) | 266.7 (206.7, 338.1) |
| eld. (QV) + paed. (QV), 25% | 2473.2 (2220.3, 2722.9) | 3483.3 (3118.8, 3857.7) | 549.4 (455.2, 659.9) | 2165.5 (1899, 2419.9) | 3446.7 (3055.5, 3859.9) | 389.3 (302.7, 484.4) |
| eld. (QV) + paed. (QV), 50% | 3951.3 (3571.1, 4355.9) | 5815.7 (4998.6, 6737.4) | 801.1 (662.6, 962.9) | 3489.2 (3038.7, 3921.1) | 6052.1 (5313.3, 6873.4) | 530.9 (411.5, 664.3) |
| eld. (QV) + paed. (QV), 75% | 4796.4 (4346.7, 5282.9) | 7435.5 (6392.8, 8564.8) | 993.3 (822.1, 1194.1) | 4135.2 (3622.1, 4611.2) | 7751.4 (6693, 8831) | 614.5 (478.1, 768.9) |
| CrI: credible interval, eld.: elderly vaccination change (moving from TV to iTV or QV), iTV: improved trivalent vaccine (i.e., adjuvanted or high-dose), paed.: paediatric mass vaccination (scenario with specified vaccine and uptake rate), QV: quadrivalent vaccine (non-adjuvanted, non-high dose), TIV: trivalent vaccine (non-adjuvanted, non-high dose). | | | | | | |

| Supplementary Table 14: Mean (95%-CrI) of the relative reduction of infections per setting and by age groups. | | | | | | |
| --- | --- | --- | --- | --- | --- | --- |
| **setting** | **Scotland (SC)** | | | **Ireland (IE)** | | |
| **vaccination strategy / age group** | **[0, 15)** | **[15, 65)** | **[65, +)** | **[0, 15)** | **[15, 65)** | **[65, +)** |
| elderly (iTV) | 77.3 (54.1, 134.3) | 1058.1 (896.9, 1246.7) | 435.3 (360.3, 522.1) | 78.9 (63.2, 96.6) | 328.7 (266.9, 398.9) | 177.4 (142.7, 217.1) |
| elderly (QV) | 43.2 (27.1, 80) | 807 (620.9, 1030.1) | 262.3 (196.5, 344.4) | 41 (29.1, 55.4) | 176 (128.6, 231) | 77.6 (56, 102.9) |
| paed. (TV), 10% | 412.8 (212.6, 908.4) | 757.4 (559.4, 1337.5) | 60.8 (35.2, 137.8) | 1354.9 (1268.2, 1470.9) | 1393.5 (1253.2, 1549.3) | 108.5 (84.7, 137.9) |
| paed. (TV), 25% | 894.7 (511.9, 1710.8) | 1780.8 (1405.4, 2717.1) | 137.2 (87.2, 264.3) | 3181.7 (2867.7, 3519.6) | 3431.1 (3046.7, 3916.3) | 271.6 (211, 351.9) |
| paed. (TV), 50% | 1457.1 (945.8, 2416.7) | 3399.9 (2817.9, 4440.3) | 244 (172.9, 388.7) | 4625.5 (4175.7, 5169.2) | 5768.1 (5037.6, 6682.5) | 450.9 (351.7, 586.6) |
| paed. (TV), 75% | 1830.9 (1311.3, 2788.7) | 4856.7 (4155.8, 5956.3) | 341.4 (256.9, 491.7) | 5451.8 (4948.1, 6024.4) | 7508.4 (6614.2, 8606.8) | 580.3 (456.5, 744) |
| paed. (QV), 10% | 481.9 (268, 988.9) | 933.3 (717.7, 1536.8) | 72.2 (44.1, 153.1) | 1533.4 (1438.7, 1655) | 1619.6 (1472.7, 1779.2) | 120 (95.3, 149.8) |
| paed. (QV), 25% | 1003.9 (603.2, 1840.6) | 2059.6 (1659.2, 3001.9) | 154.2 (100.7, 285.7) | 3667.9 (3337, 4027) | 4085.1 (3679.5, 4582.5) | 302.4 (239, 384.5) |
| paed. (QV), 50% | 1599.6 (1074, 2555.6) | 3872 (3220.3, 4930.4) | 270.1 (193.4, 416.7) | 5312.5 (4814.3, 5881.2) | 6937.5 (6146.7, 7885.4) | 511.4 (404.7, 654.6) |
| paed. (QV), 75% | 1929.7 (1412.7, 2883.1) | 5238 (4491.6, 6346.7) | 366.6 (277.8, 519.7) | 6365.5 (5818.5, 6954) | 9318.8 (8302.8, 10492.2) | 671.8 (535.4, 846.3) |
| eld. (iTV) + paed. (TV), 10% | 483.5 (270.4, 992) | 1812.6 (1516.6, 2435.1) | 486 (395.9, 605.5) | 1435.7 (1344.2, 1557.4) | 1725.5 (1561.2, 1898.9) | 275.9 (226.5, 330.7) |
| eld. (iTV) + paed. (TV), 25% | 956.9 (566.9, 1782.7) | 2836.3 (2398.6, 3807.7) | 550.1 (444.5, 705.7) | 3259 (2945.2, 3609.1) | 3764.3 (3366.8, 4254.6) | 422.9 (345.2, 515.8) |
| eld. (iTV) + paed. (TV), 50% | 1512.5 (998.9, 2471.6) | 4472.9 (3870.8, 5541.4) | 641.3 (521.1, 808.8) | 4677.6 (4224.9, 5217.8) | 6084.6 (5350, 7014.1) | 581.5 (467.9, 723.7) |
| eld. (iTV) + paed. (TV), 75% | 1875.1 (1354.5, 2834.4) | 5929.6 (5201.5, 7034.6) | 724.2 (593.1, 901.3) | 5496.1 (4984.6, 6066.2) | 7836.8 (6919.8, 8956.4) | 699.6 (563.5, 869.4) |
| eld. (QV) + paed. (TV), 10% | 454.6 (251.1, 948.6) | 1569.1 (1247.1, 2179.9) | 317.8 (237.5, 425.1) | 1396.5 (1306.6, 1514.1) | 1572.8 (1420.3, 1738.9) | 182.9 (147.7, 223.1) |
| eld. (QV) + paed. (TV), 25% | 934.9 (550.2, 1758.1) | 2601 (2142.1, 3552.1) | 386.6 (289.9, 533.6) | 3224.6 (2910.1, 3565.3) | 3616.6 (3228.9, 4108.2) | 340.9 (274.4, 426.5) |
| eld. (QV) + paed. (TV), 50% | 1497 (984.4, 2462.8) | 4244.9 (3640.2, 5298.9) | 480.7 (372.6, 641.5) | 4664.6 (4211.8, 5205.6) | 5959.1 (5236, 6880.7) | 512.4 (407.8, 653.4) |
| eld. (QV) + paed. (TV), 75% | 1859.9 (1341.8, 2816.7) | 5689.3 (4937.5, 6807.4) | 566.1 (446.6, 733.4) | 5492.4 (4984.2, 6062.8) | 7721.4 (6817, 8839) | 636 (505.6, 801.6) |
| eld. (iTV) + paed. (QV), 10% | 552 (327, 1079.9) | 1990.1 (1683.2, 2649.4) | 495.8 (404.1, 618.2) | 1614.7 (1517.4, 1738.2) | 1953.1 (1783.5, 2129.1) | 286.3 (235.7, 342.3) |
| eld. (iTV) + paed. (QV), 25% | 1064.9 (657.1, 1912) | 3117.9 (2664.4, 4095.2) | 564.7 (456.8, 722.6) | 3746.8 (3413.6, 4114.1) | 4422.7 (4007.9, 4927.9) | 450.7 (370.2, 546.5) |
| eld. (iTV) + paed. (QV), 50% | 1651.4 (1124.3, 2604.6) | 4944.7 (4288.5, 6000.3) | 663.6 (539.6, 834.2) | 5370 (4867.7, 5939.9) | 7271.7 (6473.4, 8215.5) | 636.2 (516.6, 783.8) |
| eld. (iTV) + paed. (QV), 75% | 1970.4 (1450.8, 2927.3) | 6312.4 (5530.5, 7428.4) | 745.6 (611.5, 925.2) | 6405.4 (5860.3, 6999.2) | 9644.9 (8615.4, 10832.6) | 780.6 (633.8, 957.9) |
| eld. (QV) + paed. (QV), 10% | 522.7 (306.6, 1032.4) | 1748 (1416, 2375.8) | 326.3 (243.6, 437.8) | 1575.9 (1479.8, 1697) | 1801.5 (1643.1, 1970.3) | 192.3 (155.6, 234.1) |
| eld. (QV) + paed. (QV), 25% | 1042.1 (640.1, 1887) | 2885.1 (2413.8, 3853.8) | 399.2 (300.1, 547.5) | 3713.9 (3383.1, 4077.3) | 4278.4 (3865.6, 4786.5) | 366.2 (296.3, 453.6) |
| eld. (QV) + paed. (QV), 50% | 1632.9 (1107.4, 2587.1) | 4715.9 (4048, 5785) | 499.9 (387, 665.2) | 5361.4 (4860.1, 5930.7) | 7160.2 (6367.2, 8108.4) | 562.4 (449.8, 709.5) |
| eld. (QV) + paed. (QV), 75% | 1952.5 (1436.1, 2904.1) | 6072.8 (5276.5, 7203.9) | 584.4 (461.9, 753.4) | 6397.6 (5855.5, 6987) | 9525.7 (8491.5, 10713.5) | 708.8 (567.8, 886.5) |
| CrI: credible interval, eld.: elderly vaccination change (moving from TV to iTV or QV), iTV: improved trivalent vaccine (i.e., adjuvanted or high-dose), paed.: paediatric mass vaccination (scenario with specified vaccine and uptake rate), QV: quadrivalent vaccine (non-adjuvanted, non-high dose), TIV: trivalent vaccine (non-adjuvanted, non-high dose). | | | | | | |

Number of events averted per 100,000 doses per setting

Detailed changes in the number of events averted across age groups per 100,000 doses per setting (complementary to Figure 4 in the main text).


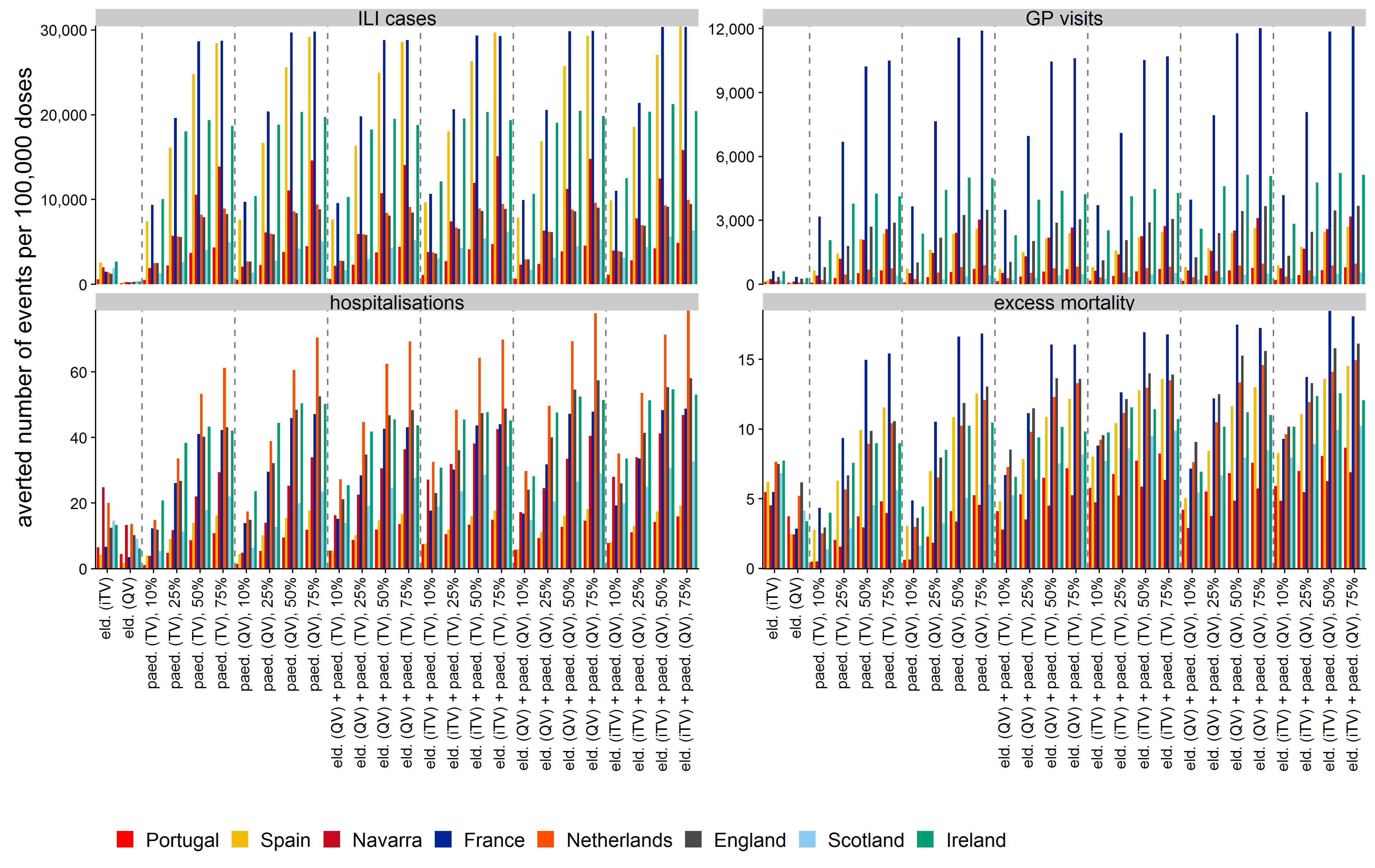
Supplementary Figure 55. Mean number of events averted across age groups, per 100,000 doses.

| Supplementary Table 15: Mean (95%-CrI) number of events averted per 100,000 doses across all ages per setting. | | | | |
| --- | --- | --- | --- | --- |
| **vaccination strategy** | **symptomatic cases** | **GP visits** | **hospitalisations** | **excess deaths** |
| **setting** | **Portugal (PT)** | | | |
| elderly (iTV) | 590 (530, 650) | 120 (100, 130) | 6.5 (5.9, 7.2) | 5.5 (4.9, 6.0) |
| elderly (QV) | 110 (89.8, 120) | 78.4 (65.2, 90.2) | 4.5 (3.7, 5.2) | 3.7 (3.1, 4.3) |
| paed. (TV), 10% | 520 (490, 540) | 67.5 (62.7, 72.0) | 1.1 (1.0, 1.2) | 0.5 (0.4, 0.5) |
| paed. (TV), 25% | 2200 (2100, 2300) | 300 ( 280, 310) | 4.9 (4.5, 5.2) | 2.0 (1.8, 2.3) |
| paed. (TV), 50% | 3700 (3400, 3900) | 530 ( 490, 560) | 8.7 (8.0, 9.3) | 3.7 (3.3, 4.1) |
| paed. (TV), 75% | 4400 (4100, 4600) | 650 ( 600, 690) | 10.8 (9.9, 11.6) | 4.8 (4.3, 5.3) |
| paed. (QV), 10% | 560 (540, 580) | 88.8 (82.7, 94.3) | 1.5 (1.3, 1.6) | 0.6 (0.5, 0.7) |
| paed. (QV), 25% | 2300 (2200, 2400) | 330 ( 310, 350) | 5.4 (5.0, 5.8) | 2.3 (2.0, 2.5) |
| paed. (QV), 50% | 3800 (3600, 4000) | 580 ( 540, 620) | 9.5 (8.7, 10.2) | 4.1 (3.6, 4.5) |
| paed. (QV), 75% | 4500 (4200, 4800) | 720 ( 670, 770) | 11.8 (10.8, 12.7) | 5.2 (4.6, 5.8) |
| eld. (iTV) + paed. (TV), 10% | 1100 (1000, 1200) | 180 (170, 190) | 7.5 (6.8, 8.1) | 5.8 (5.2, 6.3) |
| eld. (iTV) + paed. (TV), 25% | 2700 (2600, 2800) | 400 ( 380, 420) | 10.5 (9.8, 11.2) | 6.8 (6.2, 7.4) |
| eld. (iTV) + paed. (TV), 50% | 4100 (3900, 4300) | 610 ( 580, 650) | 13.4 (12.5, 14.3) | 7.7 (7.0, 8.4) |
| eld. (iTV) + paed. (TV), 75% | 4700 (4400, 5000) | 730 ( 680, 770) | 14.9 (13.8, 15.9) | 8.2 (7.5, 8.9) |
| eld. (QV) + paed. (TV), 10% | 620 (590, 650) | 140 (130, 160) | 5.5 (4.7, 6.2) | 4.1 (3.4, 4.7) |
| eld. (QV) + paed. (TV), 25% | 2300 (2200, 2400) | 370 ( 340, 390) | 8.8 (7.9, 9.5) | 5.3 (4.6, 5.9) |
| eld. (QV) + paed. (TV), 50% | 3800 (3500, 4000) | 590 ( 550, 620) | 12.0 (10.9, 12.9) | 6.5 (5.7, 7.2) |
| eld. (QV) + paed. (TV), 75% | 4400 (4100, 4700) | 700 ( 650, 750) | 13.6 (12.5, 14.7) | 7.2 (6.4, 7.9) |
| eld. (iTV) + paed. (QV), 10% | 1100 (1100, 1200) | 200 ( 190, 210) | 7.8 (7.1, 8.4) | 5.9 (5.3, 6.5) |
| eld. (iTV) + paed. (QV), 25% | 2800 (2700, 2900) | 430 ( 410, 460) | 11.1 (10.3, 11.8) | 7.0 (6.4, 7.6) |
| eld. (iTV) + paed. (QV), 50% | 4200 (4000, 4500) | 670 ( 630, 710) | 14.3 (13.2, 15.2) | 8.1 (7.3, 8.8) |
| eld. (iTV) + paed. (QV), 75% | 4900 (4600, 5100) | 790 ( 740, 840) | 15.9 (14.7, 17.0) | 8.6 (7.8, 9.4) |
| eld. (QV) + paed. (QV), 10% | 660 (630, 690) | 160 (150, 180) | 5.8 (4.9, 6.5) | 4.2 (3.5, 4.8) |
| eld. (QV) + paed. (QV), 25% | 2400 (2300, 2500) | 400 ( 380, 420) | 9.3 (8.4, 10.1) | 5.5 (4.8, 6.2) |
| eld. (QV) + paed. (QV), 50% | 3900 (3600, 4100) | 640 ( 600, 680) | 12.8 (11.7, 13.8) | 6.8 (6.0, 7.6) |
| eld. (QV) + paed. (QV), 75% | 4600 (4300, 4800) | 770 ( 720, 820) | 14.6 (13.4, 15.7) | 7.6 (6.7, 8.3) |
| **setting** | **Spain (ES)** | | | |
| elderly (iTV) | 2500 (2400, 2700) | 180 ( 170, 190) | 4.3 (4.1, 4.4) | 6.2 (5.9, 6.5) |
| elderly (QV) | 270 (250, 290) | 80.6 (73.1, 87.0) | 1.7 (1.6, 1.9) | 2.5 (2.3, 2.6) |
| paed. (TV), 10% | 7400 (7300, 7500) | 650 ( 630, 670) | 4.0 (3.9, 4.1) | 2.8 (2.6, 2.9) |
| paed. (TV), 25% | 16000 (16000, 16000) | 1400 ( 1400, 1500) | 9.1 (8.7, 9.4) | 6.3 (5.9, 6.6) |
| paed. (TV), 50% | 25000 (24000, 26000) | 2100 ( 2100, 2200) | 14.0 (13.4, 14.5) | 9.9 (9.3, 10.5) |
| paed. (TV), 75% | 28000 (27000, 30000) | 2400 ( 2300, 2400) | 16.2 (15.4, 16.8) | 11.5 (10.8, 12.2) |
| paed. (QV), 10% | 7600 (7500, 7800) | 730 ( 710, 750) | 4.5 (4.3, 4.6) | 3.1 (2.9, 3.2) |
| paed. (QV), 25% | 17000 (16000, 17000) | 1600 ( 1600, 1700) | 10.1 (9.8, 10.5) | 7.0 (6.6, 7.4) |
| paed. (QV), 50% | 26000 (25000, 26000) | 2400 ( 2300, 2400) | 15.5 (14.9, 16.0) | 10.9 (10.2, 11.4) |
| paed. (QV), 75% | 29000 (28000, 30000) | 2600 ( 2500, 2700) | 17.7 (17.0, 18.4) | 12.6 (11.8, 13.2) |
| eld. (iTV) + paed. (TV), 10% | 9600 (9500, 9800) | 810 ( 790, 830) | 7.7 (7.4, 7.9) | 8.0 (7.7, 8.3) |
| eld. (iTV) + paed. (TV), 25% | 18000 (18000, 18000) | 1600 ( 1500, 1600) | 12.0 (11.6, 12.3) | 10.4 (10.0, 10.9) |
| eld. (iTV) + paed. (TV), 50% | 26000 (25000, 27000) | 2200 ( 2200, 2300) | 16.0 (15.5, 16.5) | 12.8 (12.1, 13.3) |
| eld. (iTV) + paed. (TV), 75% | 30000 (28000, 31000) | 2500 ( 2400, 2500) | 17.7 (17.0, 18.4) | 13.6 (12.9, 14.3) |
| eld. (QV) + paed. (TV), 10% | 7600 (7500, 7800) | 720 ( 710, 740) | 5.5 (5.3, 5.7) | 4.8 (4.6, 5.0) |
| eld. (QV) + paed. (TV), 25% | 16000 (16000, 17000) | 1500 ( 1500, 1500) | 10.2 (9.9, 10.5) | 7.8 (7.4, 8.2) |
| eld. (QV) + paed. (TV), 50% | 25000 (24000, 26000) | 2200 ( 2100, 2200) | 14.7 (14.2, 15.2) | 10.9 (10.3, 11.4) |
| eld. (QV) + paed. (TV), 75% | 29000 (27000, 30000) | 2400 ( 2300, 2500) | 16.7 (16.0, 17.4) | 12.2 (11.4, 12.8) |
| eld. (iTV) + paed. (QV), 10% | 9900 (9700, 10000) | 890 ( 870, 910) | 8.1 (7.9, 8.3) | 8.3 (8.0, 8.6) |
| eld. (iTV) + paed. (QV), 25% | 19000 (18000, 19000) | 1800 ( 1700, 1800) | 13.0 (12.6, 13.4) | 11.1 (10.6, 11.5) |
| eld. (iTV) + paed. (QV), 50% | 27000 (26000, 28000) | 2500 ( 2400, 2500) | 17.5 (16.9, 18.0) | 13.6 (12.9, 14.2) |
| eld. (iTV) + paed. (QV), 75% | 30000 (29000, 32000) | 2700 ( 2600, 2800) | 19.2 (18.5, 19.9) | 14.5 (13.8, 15.2) |
| eld. (QV) + paed. (QV), 10% | 7900 (7700, 8000) | 800 ( 780, 820) | 5.9 (5.7, 6.1) | 5.1 (4.8, 5.3) |
| eld. (QV) + paed. (QV), 25% | 17000 (17000, 17000) | 1700 ( 1600, 1700) | 11.2 (10.8, 11.6) | 8.4 (8.0, 8.9) |
| eld. (QV) + paed. (QV), 50% | 26000 (25000, 26000) | 2400 ( 2300, 2500) | 16.1 (15.5, 16.6) | 11.7 (11.0, 12.3) |
| eld. (QV) + paed. (QV), 75% | 29000 (28000, 30000) | 2600 ( 2600, 2700) | 18.1 (17.4, 18.8) | 13.0 (12.3, 13.7) |
| CrI: credible interval, eld.: elderly vaccination change, GP: general practitioner, iTV: improved trivalent vaccine (i.e., adjuvanted or high-dose), paed.: paediatric mass vaccination, QV: quadrivalent vaccine (non-adjuvanted, non-high dose), TV: trivalent vaccine (non-adjuvanted, non-high dose). | | | | |
| Supplementary Table 16: Mean (95%-CrI) number of events averted per 100,000 doses across all ages per setting. | | | | |
| **vaccination strategy** | **symptomatic cases** | **GP visits** | **hospitalisations** | **excess deaths** |
| **setting** | **Navarra (NV)** | | | |
| elderly (iTV) | 2000 (1900, 2100) | 240 ( 230, 250) | 24.8 (23.6, 25.9) | 4.5 (4.3, 4.7) |
| elderly (QV) | 260 (240, 290) | 130 (120, 140) | 13.3 (12.0, 14.6) | 2.4 (2.2, 2.7) |
| paed. (TV), 10% | 1900 (1800, 2000) | 410 ( 390, 420) | 3.9 (3.6, 4.1) | 0.5 (0.5, 0.5) |
| paed. (TV), 25% | 5700 (5500, 5900) | 1200 (1100, 1200) | 11.8 (11.1, 12.4) | 1.6 (1.5, 1.7) |
| paed. (TV), 50% | 11000 (10000, 11000) | 2100 (2000, 2200) | 22.0 (20.5, 23.2) | 2.9 (2.7, 3.1) |
| paed. (TV), 75% | 14000 (13000, 15000) | 2600 ( 2400, 2700) | 29.4 (26.9, 31.6) | 4.0 (3.6, 4.3) |
| paed. (QV), 10% | 2100 (2000, 2200) | 520 ( 510, 540) | 4.8 (4.6, 5.1) | 0.6 (0.6, 0.7) |
| paed. (QV), 25% | 6100 (5900, 6300) | 1500 (1400, 1500) | 14.0 (13.2, 14.7) | 1.8 (1.7, 2.0) |
| paed. (QV), 50% | 11000 (10000, 11000) | 2400 (2300, 2500) | 25.3 (23.5, 26.6) | 3.4 (3.1, 3.6) |
| paed. (QV), 75% | 15000 (14000, 16000) | 3000 ( 2900, 3200) | 34.0 (31.2, 36.3) | 4.6 (4.1, 4.9) |
| eld. (iTV) + paed. (TV), 10% | 3800 (3700, 3900) | 640 ( 620, 660) | 27.1 (25.9, 28.3) | 4.7 (4.5, 5.0) |
| eld. (iTV) + paed. (TV), 25% | 7400 (7200, 7600) | 1400 (1300, 1400) | 31.9 (30.6, 33.2) | 5.2 (5.0, 5.5) |
| eld. (iTV) + paed. (TV), 50% | 12000 (11000, 12000) | 2300 ( 2200, 2300) | 38.2 (36.4, 39.8) | 5.9 (5.6, 6.1) |
| eld. (iTV) + paed. (TV), 75% | 15000 (14000, 16000) | 2700 ( 2600, 2900) | 42.5 (40.1, 44.7) | 6.4 (6.0, 6.7) |
| eld. (QV) + paed. (TV), 10% | 2200 (2100, 2200) | 540 ( 520, 550) | 16.3 (15.0, 17.6) | 2.8 (2.5, 3.0) |
| eld. (QV) + paed. (TV), 25% | 5900 (5700, 6200) | 1300 (1300, 1300) | 22.5 (21.2, 23.8) | 3.5 (3.3, 3.7) |
| eld. (QV) + paed. (TV), 50% | 11000 (10000, 11000) | 2200 (2100, 2300) | 30.6 (28.8, 32.2) | 4.5 (4.2, 4.8) |
| eld. (QV) + paed. (TV), 75% | 14000 (13000, 15000) | 2700 ( 2500, 2800) | 36.4 (33.8, 38.7) | 5.2 (4.8, 5.6) |
| eld. (iTV) + paed. (QV), 10% | 4000 (3800, 4100) | 750 ( 730, 780) | 28.0 (26.8, 29.2) | 4.9 (4.6, 5.1) |
| eld. (iTV) + paed. (QV), 25% | 7800 (7500, 8000) | 1700 (1600, 1700) | 34.0 (32.6, 35.4) | 5.5 (5.2, 5.7) |
| eld. (iTV) + paed. (QV), 50% | 12000 (12000, 13000) | 2600 ( 2500, 2700) | 41.2 (39.3, 43.0) | 6.3 (5.9, 6.6) |
| eld. (iTV) + paed. (QV), 75% | 16000 (15000, 17000) | 3200 ( 3000, 3300) | 46.9 (44.2, 49.2) | 6.9 (6.5, 7.3) |
| eld. (QV) + paed. (QV), 10% | 2300 (2300, 2400) | 650 ( 630, 670) | 17.2 (15.9, 18.5) | 2.9 (2.6, 3.1) |
| eld. (QV) + paed. (QV), 25% | 6300 (6100, 6500) | 1600 (1500, 1600) | 24.5 (23.0, 25.9) | 3.8 (3.5, 4.0) |
| eld. (QV) + paed. (QV), 50% | 11000 (11000, 12000) | 2500 ( 2400, 2600) | 33.5 (31.5, 35.3) | 4.9 (4.5, 5.2) |
| eld. (QV) + paed. (QV), 75% | 15000 (14000, 16000) | 3100 ( 3000, 3300) | 40.5 (37.7, 43.0) | 5.7 (5.3, 6.1) |
| **setting** | **France (FR)** | | | |
| elderly (iTV) | 1500 (1400, 1600) | 620 ( 580, 650) | 6.7 (6.2, 7.1) | 5.5 (5.1, 5.8) |
| elderly (QV) | 230 (210, 250) | 350 (320, 390) | 3.5 (3.2, 3.9) | 2.9 (2.5, 3.1) |
| paed. (TV), 10% | 9400 (9200, 9600) | 3200 (3100, 3300) | 12.3 (11.9, 12.6) | 4.4 (4.1, 4.6) |
| paed. (TV), 25% | 20000 (19000, 20000) | 6700 ( 6500, 6800) | 26.1 (25.3, 26.9) | 9.4 (8.8, 9.9) |
| paed. (TV), 50% | 29000 (28000, 30000) | 10000 ( 9800, 11000) | 41.0 (39.1, 42.9) | 15.0 (13.8, 16.0) |
| paed. (TV), 75% | 29000 (28000, 30000) | 10000 (10000, 11000) | 42.3 (40.6, 44.0) | 15.4 (14.4, 16.4) |
| paed. (QV), 10% | 9700 (9500, 9900) | 3700 (3600, 3700) | 13.9 (13.5, 14.3) | 4.9 (4.6, 5.1) |
| paed. (QV), 25% | 20000 (20000, 21000) | 7700 ( 7500, 7800) | 29.5 (28.5, 30.5) | 10.5 (9.9, 11.1) |
| paed. (QV), 50% | 30000 (29000, 31000) | 12000 (11000, 12000) | 45.9 (43.8, 48.0) | 16.6 (15.4, 17.8) |
| paed. (QV), 75% | 30000 (29000, 31000) | 12000 (12000, 12000) | 47.2 (45.4, 48.8) | 16.9 (15.8, 17.8) |
| eld. (iTV) + paed. (TV), 10% | 11000 (10000, 11000) | 3700 (3600, 3800) | 17.7 (17.1, 18.3) | 8.8 (8.3, 9.3) |
| eld. (iTV) + paed. (TV), 25% | 21000 (20000, 21000) | 7100 ( 7000, 7300) | 30.2 (29.3, 31.1) | 12.6 (11.9, 13.3) |
| eld. (iTV) + paed. (TV), 50% | 29000 (28000, 30000) | 11000 (10000, 11000) | 43.7 (41.7, 45.5) | 16.9 (15.8, 18.0) |
| eld. (iTV) + paed. (TV), 75% | 29000 (28000, 30000) | 11000 (10000, 11000) | 44.1 (42.4, 45.7) | 16.8 (15.7, 17.8) |
| eld. (QV) + paed. (TV), 10% | 9600 (9400, 9800) | 3500 (3400, 3600) | 15.2 (14.7, 15.7) | 6.7 (6.3, 7.1) |
| eld. (QV) + paed. (TV), 25% | 20000 (19000, 20000) | 7000 ( 6800, 7100) | 28.4 (27.5, 29.3) | 11.1 (10.5, 11.7) |
| eld. (QV) + paed. (TV), 50% | 29000 (28000, 30000) | 10000 (10000, 11000) | 42.6 (40.7, 44.4) | 16.1 (15.0, 17.1) |
| eld. (QV) + paed. (TV), 75% | 29000 (28000, 30000) | 11000 (10000, 11000) | 43.2 (41.5, 44.8) | 16.1 (15.1, 17.0) |
| eld. (iTV) + paed. (QV), 10% | 11000 (11000, 11000) | 4200 ( 4100, 4300) | 19.3 (18.7, 19.9) | 9.3 (8.8, 9.8) |
| eld. (iTV) + paed. (QV), 25% | 21000 (21000, 22000) | 8100 ( 7900, 8300) | 33.6 (32.5, 34.6) | 13.7 (13.0, 14.4) |
| eld. (iTV) + paed. (QV), 50% | 30000 (29000, 31000) | 12000 (11000, 12000) | 48.4 (46.3, 50.4) | 18.5 (17.3, 19.6) |
| eld. (iTV) + paed. (QV), 75% | 30000 (29000, 31000) | 12000 (12000, 12000) | 48.8 (47.1, 50.5) | 18.1 (17.0, 19.1) |
| eld. (QV) + paed. (QV), 10% | 9900 (9700, 10000) | 4000 (3900, 4000) | 16.8 (16.2, 17.3) | 7.2 (6.7, 7.6) |
| eld. (QV) + paed. (QV), 25% | 21000 (20000, 21000) | 7900 ( 7700, 8100) | 31.8 (30.7, 32.8) | 12.2 (11.5, 12.9) |
| eld. (QV) + paed. (QV), 50% | 30000 (29000, 31000) | 12000 (11000, 12000) | 47.2 (45.2, 49.2) | 17.5 (16.3, 18.6) |
| eld. (QV) + paed. (QV), 75% | 30000 (29000, 31000) | 12000 (12000, 12000) | 47.8 (46.1, 49.4) | 17.3 (16.2, 18.2) |
| CrI: credible interval, eld.: elderly vaccination change, GP: general practitioner, iTV: improved trivalent vaccine (i.e., adjuvanted or high-dose), paed.: paediatric mass vaccination, QV: quadrivalent vaccine (non-adjuvanted, non-high dose), TV: trivalent vaccine (non-adjuvanted, non-high dose). | | | | |
| Supplementary Table 17: Mean (95%-CrI) number of events averted per 100,000 doses across all ages per setting. | | | | |
| **vaccination strategy** | **symptomatic cases** | **GP visits** | **hospitalisations** | **excess deaths** |
| **setting** | **Netherlands (NL)** | | | |
| elderly (iTV) | 1400 (1300, 1500) | 130 ( 120, 130) | 20.1 (18.7, 21.4) | 7.6 (7.1, 8.1) |
| elderly (QV) | 270 (250, 300) | 86.7 (78.4, 94.0) | 13.6 (12.3, 14.7) | 5.2 (4.7, 5.7) |
| paed. (TV), 10% | 2500 (2400, 2600) | 200 ( 200, 210) | 14.8 (14.3, 15.2) | 2.5 (2.4, 2.7) |
| paed. (TV), 25% | 5700 (5500, 5800) | 460 ( 440, 470) | 33.6 (32.4, 34.8) | 5.7 (5.3, 6.0) |
| paed. (TV), 50% | 8200 (7800, 8600) | 690 ( 660, 720) | 53.3 (51.0, 55.4) | 9.0 (8.4, 9.5) |
| paed. (TV), 75% | 8900 (8500, 9300) | 760 ( 720, 790) | 61.2 (58.6, 63.6) | 10.4 (9.7, 11.0) |
| paed. (QV), 10% | 2700 (2600, 2800) | 250 ( 240, 260) | 17.5 (16.9, 18.0) | 3.0 (2.8, 3.1) |
| paed. (QV), 25% | 6000 (5700, 6100) | 550 ( 530, 560) | 38.9 (37.5, 40.3) | 6.5 (6.1, 6.9) |
| paed. (QV), 50% | 8600 (8200, 9000) | 810 ( 770, 840) | 60.6 (58.0, 63.0) | 10.2 (9.6, 10.9) |
| paed. (QV), 75% | 9400 (9000, 9800) | 900 ( 860, 940) | 70.4 (67.3, 73.3) | 12.1 (11.3, 12.8) |
| eld. (iTV) + paed. (TV), 10% | 3800 (3600, 3900) | 320 ( 300, 330) | 32.5 (31.0, 33.9) | 9.2 (8.7, 9.8) |
| eld. (iTV) + paed. (TV), 25% | 6700 (6400, 6900) | 550 ( 540, 570) | 48.4 (46.6, 50.2) | 11.2 (10.5, 11.8) |
| eld. (iTV) + paed. (TV), 50% | 8900 (8500, 9300) | 760 ( 730, 790) | 64.3 (61.8, 66.8) | 13.0 (12.2, 13.7) |
| eld. (iTV) + paed. (TV), 75% | 9500 (9000, 9900) | 820 ( 780, 850) | 69.8 (67.0, 72.4) | 13.5 (12.7, 14.2) |
| eld. (QV) + paed. (TV), 10% | 2800 (2700, 2900) | 280 ( 270, 290) | 27.3 (25.9, 28.5) | 7.3 (6.8, 7.8) |
| eld. (QV) + paed. (TV), 25% | 5900 (5700, 6100) | 530 ( 520, 550) | 44.7 (43.0, 46.3) | 9.8 (9.2, 10.3) |
| eld. (QV) + paed. (TV), 50% | 8400 (8000, 8800) | 750 ( 720, 780) | 62.5 (59.9, 64.9) | 12.3 (11.6, 13.0) |
| eld. (QV) + paed. (TV), 75% | 9100 (8700, 9500) | 820 ( 780, 850) | 69.3 (66.4, 71.9) | 13.3 (12.5, 14.0) |
| eld. (iTV) + paed. (QV), 10% | 3900 (3800, 4100) | 360 ( 350, 370) | 35.1 (33.5, 36.6) | 9.6 (9.0, 10.2) |
| eld. (iTV) + paed. (QV), 25% | 7000 (6700, 7200) | 640 ( 620, 660) | 53.5 (51.5, 55.5) | 11.9 (11.2, 12.6) |
| eld. (iTV) + paed. (QV), 50% | 9300 (8900, 9800) | 880 ( 850, 910) | 71.4 (68.5, 74.1) | 14.1 (13.3, 14.9) |
| eld. (iTV) + paed. (QV), 75% | 10000 (9500, 10000) | 960 ( 920, 1000) | 78.6 (75.3, 81.7) | 14.9 (14.0, 15.8) |
| eld. (QV) + paed. (QV), 10% | 2900 (2800, 3000) | 330 ( 320, 340) | 29.8 (28.3, 31.1) | 7.6 (7.1, 8.1) |
| eld. (QV) + paed. (QV), 25% | 6200 (6000, 6400) | 620 ( 600, 640) | 49.7 (47.7, 51.5) | 10.5 (9.9, 11.1) |
| eld. (QV) + paed. (QV), 50% | 8800 (8400, 9200) | 870 ( 840, 910) | 69.4 (66.5, 72.1) | 13.3 (12.5, 14.1) |
| eld. (QV) + paed. (QV), 75% | 9600 (9200, 10000) | 960 ( 920, 1000) | 77.9 (74.5, 81.0) | 14.6 (13.6, 15.4) |
| **setting** | **England (EN)** | | | |
| elderly (iTV) | 1200 (1100, 1300) | 340 ( 320, 360) | 12.5 (11.6, 13.4) | 7.5 (6.9, 8.0) |
| elderly (QV) | 280 (250, 310) | 270 (240, 290) | 10.2 (9.1, 11.1) | 6.2 (5.5, 6.8) |
| paed. (TV), 10% | 2500 (2400, 2600) | 810 ( 780, 840) | 11.9 (11.3, 12.5) | 2.9 (2.7, 3.2) |
| paed. (TV), 25% | 5600 (5300, 5900) | 1800 (1700, 1900) | 26.7 (25.3, 28.3) | 6.7 (6.1, 7.3) |
| paed. (TV), 50% | 7900 (7500, 8400) | 2700 (2600, 2800) | 40.2 (38.2, 42.4) | 9.9 (9.1, 10.6) |
| paed. (TV), 75% | 8300 (7800, 8800) | 2900 (2800, 3000) | 43.1 (41.0, 45.3) | 10.6 (9.8, 11.3) |
| paed. (QV), 10% | 2700 (2600, 2800) | 1000 ( 990, 1100) | 14.9 (14.3, 15.6) | 3.6 (3.4, 3.9) |
| paed. (QV), 25% | 5900 (5600, 6200) | 2200 (2100, 2300) | 32.2 (30.6, 33.9) | 7.9 (7.3, 8.6) |
| paed. (QV), 50% | 8400 (7900, 8900) | 3200 (3100, 3400) | 48.4 (46.0, 50.8) | 11.9 (11.0, 12.7) |
| paed. (QV), 75% | 8900 (8400, 9300) | 3500 (3300, 3700) | 52.6 (49.7, 55.5) | 13.1 (12.0, 14.0) |
| eld. (iTV) + paed. (TV), 10% | 3600 (3500, 3800) | 1100 (1100, 1200) | 23.1 (22.0, 24.1) | 9.6 (9.0, 10.1) |
| eld. (iTV) + paed. (TV), 25% | 6600 (6300, 6900) | 2100 (2000, 2200) | 36.1 (34.4, 37.8) | 12.2 (11.4, 12.9) |
| eld. (iTV) + paed. (TV), 50% | 8600 (8200, 9100) | 2900 (2800, 3000) | 47.4 (45.2, 49.6) | 14.0 (13.1, 14.9) |
| eld. (iTV) + paed. (TV), 75% | 8900 (8400, 9400) | 3100 (2900, 3200) | 48.8 (46.6, 51.0) | 13.9 (13.0, 14.7) |
| eld. (QV) + paed. (TV), 10% | 2800 (2700, 2900) | 1100 (1000, 1100) | 21.2 (20.0, 22.3) | 8.5 (7.8, 9.1) |
| eld. (QV) + paed. (TV), 25% | 5800 (5500, 6200) | 2000 (1900, 2100) | 34.8 (33.1, 36.6) | 11.5 (10.6, 12.3) |
| eld. (QV) + paed. (TV), 50% | 8100 (7700, 8600) | 2900 (2800, 3000) | 46.7 (44.5, 49.1) | 13.6 (12.7, 14.6) |
| eld. (QV) + paed. (TV), 75% | 8500 (8000, 8900) | 3000 (2900, 3200) | 48.3 (46.1, 50.5) | 13.6 (12.7, 14.5) |
| eld. (iTV) + paed. (QV), 10% | 3800 (3700, 4000) | 1300 (1300, 1400) | 26.0 (24.8, 27.1) | 10.2 (9.5, 10.8) |
| eld. (iTV) + paed. (QV), 25% | 6900 (6600, 7200) | 2500 (2400, 2500) | 41.4 (39.6, 43.2) | 13.3 (12.5, 14.1) |
| eld. (iTV) + paed. (QV), 50% | 9100 (8700, 9600) | 3500 (3300, 3600) | 55.3 (52.8, 57.8) | 15.8 (14.8, 16.7) |
| eld. (iTV) + paed. (QV), 75% | 9500 (9000, 9900) | 3700 (3500, 3800) | 58.1 (55.2, 60.9) | 16.1 (15.1, 17.1) |
| eld. (QV) + paed. (QV), 10% | 2900 (2800, 3100) | 1300 (1200, 1300) | 24.0 (22.7, 25.2) | 9.1 (8.3, 9.7) |
| eld. (QV) + paed. (QV), 25% | 6100 (5900, 6500) | 2400 (2300, 2500) | 40.0 (38.1, 42.0) | 12.5 (11.6, 13.4) |
| eld. (QV) + paed. (QV), 50% | 8600 (8100, 9100) | 3400 (3300, 3600) | 54.6 (51.9, 57.2) | 15.3 (14.2, 16.3) |
| eld. (QV) + paed. (QV), 75% | 9000 (8600, 9500) | 3700 (3500, 3800) | 57.4 (54.5, 60.2) | 15.6 (14.5, 16.6) |
| CrI: credible interval, eld.: elderly vaccination change, GP: general practitioner, iTV: improved trivalent vaccine (i.e., adjuvanted or high-dose), paed.: paediatric mass vaccination, QV: quadrivalent vaccine (non-adjuvanted, non-high dose), TV: trivalent vaccine (non-adjuvanted, non-high dose). | | | | |
| Supplementary Table 18: Mean (95%-CrI) number of events averted per 100,000 doses across all ages per setting. | | | | |
| **vaccination strategy** | **symptomatic cases** | **GP visits** | **hospitalisations** | **excess deaths** |
| **setting** | **Scotland (SC)** | | | |
| elderly (iTV) | 1900 (1700, 2000) | 170 ( 160, 180) | 14.6 (13.6, 15.5) | 6.8 (6.4, 7.2) |
| elderly (QV) | 370 (330, 400) | 110 (100, 120) | 9.1 (8.3, 9.9) | 4.1 (3.7, 4.5) |
| paed. (TV), 10% | 1300 (830, 1600) | 98.5 (69.0, 120) | 5.4 (3.7, 6.5) | 1.4 (0.9, 1.6) |
| paed. (TV), 25% | 2700 (1900, 3200) | 210 ( 160, 240) | 11.3 (8.5, 13.3) | 2.9 (2.1, 3.4) |
| paed. (TV), 50% | 4000 (3300, 4600) | 330 ( 280, 370) | 17.9 (15.2, 20.1) | 4.6 (3.8, 5.1) |
| paed. (TV), 75% | 4900 (4300, 5500) | 400 ( 360, 440) | 22.0 (19.5, 23.9) | 5.6 (5.0, 6.1) |
| paed. (QV), 10% | 1400 (910, 1700) | 120 (87.2, 140) | 6.4 (4.7, 7.6) | 1.6 (1.2, 1.9) |
| paed. (QV), 25% | 2800 (2000, 3400) | 240 ( 190, 270) | 12.8 (9.9, 14.9) | 3.2 (2.5, 3.7) |
| paed. (QV), 50% | 4200 (3400, 4800) | 370 ( 320, 410) | 20.0 (17.3, 22.1) | 5.1 (4.3, 5.6) |
| paed. (QV), 75% | 5100 (4400, 5600) | 430 ( 390, 470) | 23.4 (21.0, 25.4) | 6.0 (5.3, 6.5) |
| eld. (iTV) + paed. (TV), 10% | 3100 (2600, 3400) | 260 ( 230, 280) | 19.0 (17.1, 20.4) | 7.7 (7.1, 8.2) |
| eld. (iTV) + paed. (TV), 25% | 4300 (3500, 4800) | 350 ( 300, 390) | 23.6 (20.8, 25.8) | 8.6 (7.8, 9.2) |
| eld. (iTV) + paed. (TV), 50% | 5400 (4700, 6000) | 460 ( 410, 500) | 28.6 (25.9, 30.8) | 9.5 (8.7, 10.1) |
| eld. (iTV) + paed. (TV), 75% | 6200 (5500, 6700) | 520 ( 470, 550) | 31.2 (28.7, 33.3) | 9.9 (9.1, 10.5) |
| eld. (QV) + paed. (TV), 10% | 1700 (1200, 2000) | 210 ( 180, 230) | 14.0 (12.2, 15.3) | 5.2 (4.7, 5.7) |
| eld. (QV) + paed. (TV), 25% | 3000 (2200, 3600) | 310 ( 260, 340) | 19.1 (16.3, 21.2) | 6.4 (5.6, 7.0) |
| eld. (QV) + paed. (TV), 50% | 4300 (3600, 4900) | 420 ( 370, 450) | 24.7 (21.9, 26.8) | 7.5 (6.8, 8.1) |
| eld. (QV) + paed. (TV), 75% | 5200 (4500, 5700) | 480 ( 440, 510) | 27.7 (25.2, 29.7) | 8.2 (7.4, 8.7) |
| eld. (iTV) + paed. (QV), 10% | 3100 (2600, 3500) | 280 ( 250, 300) | 19.9 (18.0, 21.4) | 7.9 (7.3, 8.5) |
| eld. (iTV) + paed. (QV), 25% | 4400 (3600, 5000) | 380 ( 330, 420) | 25.0 (22.1, 27.2) | 8.9 (8.1, 9.6) |
| eld. (iTV) + paed. (QV), 50% | 5600 (4800, 6200) | 500 ( 450, 540) | 30.6 (27.8, 32.8) | 9.9 (9.1, 10.6) |
| eld. (iTV) + paed. (QV), 75% | 6300 (5600, 6800) | 540 ( 500, 580) | 32.6 (30.1, 34.7) | 10.2 (9.5, 10.9) |
| eld. (QV) + paed. (QV), 10% | 1700 (1300, 2000) | 220 ( 190, 250) | 14.9 (13.0, 16.3) | 5.5 (4.9, 5.9) |
| eld. (QV) + paed. (QV), 25% | 3100 (2300, 3700) | 330 ( 280, 370) | 20.5 (17.6, 22.6) | 6.7 (5.9, 7.3) |
| eld. (QV) + paed. (QV), 50% | 4500 (3700, 5100) | 450 ( 410, 490) | 26.6 (23.8, 28.8) | 8.0 (7.2, 8.6) |
| eld. (QV) + paed. (QV), 75% | 5300 (4600, 5800) | 510 ( 460, 540) | 29.1 (26.5, 31.0) | 8.5 (7.7, 9.1) |
| **setting** | **Ireland (IE)** | | | |
| elderly (iTV) | 2700 (2500, 2900) | 600 ( 560, 640) | 13.3 (12.4, 14.2) | 7.7 (7.2, 8.3) |
| elderly (QV) | 330 (300, 360) | 300 (270, 330) | 6.1 (5.5, 6.7) | 3.4 (3.0, 3.7) |
| paed. (TV), 10% | 10000 (9700, 10000) | 2100 (2000, 2100) | 20.8 (20.0, 21.4) | 4.0 (3.7, 4.3) |
| paed. (TV), 25% | 18000 (17000, 19000) | 3800 ( 3700, 3900) | 38.4 (36.7, 39.7) | 7.6 (7.0, 8.1) |
| paed. (TV), 50% | 19000 (18000, 20000) | 4300 ( 4100, 4400) | 43.3 (41.2, 45.0) | 9.0 (8.2, 9.6) |
| paed. (TV), 75% | 19000 (18000, 19000) | 4100 ( 4000, 4300) | 42.1 (40.0, 43.8) | 9.0 (8.2, 9.6) |
| paed. (QV), 10% | 10000 (10000, 11000) | 2400 (2300, 2400) | 23.6 (22.8, 24.3) | 4.4 (4.1, 4.7) |
| paed. (QV), 25% | 19000 (18000, 19000) | 4400 ( 4300, 4500) | 44.4 (42.6, 45.8) | 8.5 (7.8, 9.1) |
| paed. (QV), 50% | 20000 (19000, 21000) | 5000 ( 4800, 5200) | 50.4 (48.1, 52.3) | 10.2 (9.4, 10.9) |
| paed. (QV), 75% | 20000 (19000, 20000) | 5000 ( 4800, 5100) | 50.2 (48.0, 52.1) | 10.5 (9.7, 11.2) |
| eld. (iTV) + paed. (TV), 10% | 12000 (12000, 12000) | 2500 ( 2500, 2600) | 30.8 (29.6, 32.0) | 9.8 (9.1, 10.4) |
| eld. (iTV) + paed. (TV), 25% | 20000 (19000, 20000) | 4100 ( 4000, 4200) | 45.4 (43.5, 47.0) | 11.6 (10.7, 12.3) |
| eld. (iTV) + paed. (TV), 50% | 20000 (19000, 21000) | 4500 ( 4300, 4600) | 47.7 (45.5, 49.5) | 11.4 (10.6, 12.2) |
| eld. (iTV) + paed. (TV), 75% | 19000 (18000, 20000) | 4300 ( 4100, 4400) | 45.2 (43.1, 47.0) | 10.7 (9.9, 11.4) |
| eld. (QV) + paed. (TV), 10% | 10000 (10000, 11000) | 2300 (2200, 2400) | 25.4 (24.5, 26.3) | 6.6 (6.1, 7.0) |
| eld. (QV) + paed. (TV), 25% | 18000 (17000, 19000) | 4000 ( 3800, 4100) | 41.8 (40.0, 43.2) | 9.4 (8.7, 10.0) |
| eld. (QV) + paed. (TV), 50% | 20000 (19000, 20000) | 4400 ( 4200, 4500) | 45.5 (43.4, 47.3) | 10.1 (9.4, 10.8) |
| eld. (QV) + paed. (TV), 75% | 19000 (18000, 20000) | 4200 ( 4100, 4400) | 43.7 (41.6, 45.5) | 9.8 (9.0, 10.5) |
| eld. (iTV) + paed. (QV), 10% | 13000 (12000, 13000) | 2800 ( 2800, 2900) | 33.6 (32.4, 34.7) | 10.2 (9.5, 10.8) |
| eld. (iTV) + paed. (QV), 25% | 20000 (20000, 21000) | 4800 ( 4600, 4900) | 51.3 (49.4, 53.0) | 12.4 (11.5, 13.1) |
| eld. (iTV) + paed. (QV), 50% | 21000 (20000, 22000) | 5200 ( 5000, 5400) | 54.7 (52.3, 56.7) | 12.6 (11.7, 13.4) |
| eld. (iTV) + paed. (QV), 75% | 20000 (20000, 21000) | 5100 ( 5000, 5300) | 53.1 (50.9, 55.1) | 12.1 (11.2, 12.8) |
| eld. (QV) + paed. (QV), 10% | 11000 (10000, 11000) | 2600 (2500, 2700) | 28.2 (27.2, 29.1) | 6.9 (6.5, 7.4) |
| eld. (QV) + paed. (QV), 25% | 19000 (18000, 20000) | 4600 ( 4500, 4700) | 47.6 (45.8, 49.1) | 10.2 (9.4, 10.8) |
| eld. (QV) + paed. (QV), 50% | 20000 (20000, 21000) | 5100 ( 4900, 5300) | 52.5 (50.1, 54.4) | 11.2 (10.4, 11.9) |
| eld. (QV) + paed. (QV), 75% | 20000 (19000, 21000) | 5100 ( 4900, 5200) | 51.4 (49.2, 53.4) | 11.0 (10.2, 11.7) |
| CrI: credible interval, eld.: elderly vaccination change, GP: general practitioner, iTV: improved trivalent vaccine (i.e., adjuvanted or high-dose), paed.: paediatric mass vaccination, QV: quadrivalent vaccine (non-adjuvanted, non-high dose), TV: trivalent vaccine (non-adjuvanted, non-high dose). | | | | |

Direct medical costs (in million €) by cost category per setting

Detailed changes in the direct medical costs (in million €) as compared to the base case per setting.


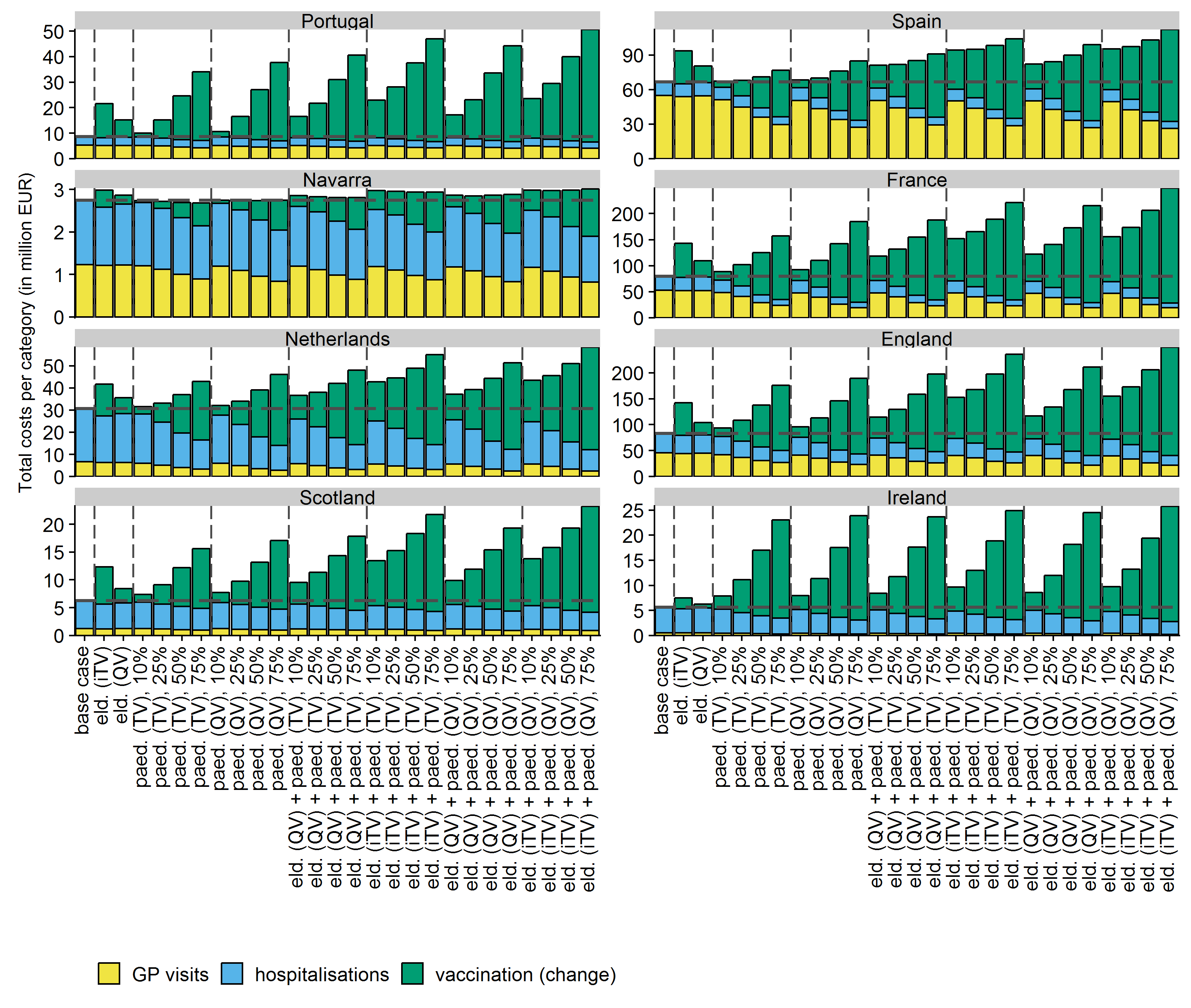
Supplementary Figure 56. Direct medical costs per strategy and setting, by cost category.

| Supplementary Table 19: Mean (95%-CrI) change of the direct medical costs (in million €) as compared to the base case per setting and by cost category. | | | | | | | | |
| --- | --- | --- | --- | --- | --- | --- | --- | --- |
| **setting** | **Portugal (PT)** | | | | **Spain (ES)** | | | |
| **vaccination strategy** | **vaccination** | **GP visits** | **hospitalisations** | **total** | **vaccination** | **GP visits** | **hospitalisations** | **total** |
| elderly (iTV) | 13.3 | -0.1 (-0.1, -0.1) | -0.2 (-0.3, -0.2) | 12.9 (12.9, 13.0) | 28.7 | -1.0 (-1.0, -0.9) | -0.7 (-0.7, -0.6) | 27.0 (26.9, 27.1) |
| elderly (QV) | 6.8 | -0.1 (-0.1, -0.1) | -0.2 (-0.2, -0.1) | 6.5 (6.5, 6.5) | 14.6 | -0.4 (-0.5, -0.4) | -0.3 (-0.3, -0.2) | 13.9 (13.8, 13.9) |
| paed. (TV), 10% | 1.5 | -0.1 (-0.1, -0.1) | 0.0 (0.0, 0.0) | 1.4 (1.4, 1.4) | 5.4 | -4.0 (-4.1, -3.9) | -0.7 (-0.7, -0.7) | 0.7 (0.6, 0.8) |
| paed. (TV), 25% | 7.2 | -0.3 (-0.4, -0.3) | -0.2 (-0.2, -0.2) | 6.6 (6.6, 6.6) | 13.4 | -10.4 (-10.6, -10.1) | -1.8 (-1.9, -1.8) | 1.2 (0.9, 1.6) |
| paed. (TV), 50% | 17.0 | -0.7 (-0.7, -0.6) | -0.4 (-0.4, -0.4) | 15.9 (15.9, 16.0) | 26.9 | -19.0 (-19.5, -18.4) | -3.5 (-3.6, -3.4) | 4.4 (3.7, 5.1) |
| paed. (TV), 75% | 26.9 | -1.0 (-1.0, -0.9) | -0.6 (-0.6, -0.5) | 25.4 (25.3, 25.5) | 40.3 | -25.4 (-26.2, -24.5) | -4.9 (-5.1, -4.7) | 10.1 (9.1, 11.1) |
| paed. (QV), 10% | 2.2 | -0.1 (-0.1, -0.1) | -0.1 (-0.1, -0.1) | 2.1 (2.0, 2.1) | 6.9 | -4.5 (-4.6, -4.4) | -0.8 (-0.8, -0.7) | 1.6 (1.5, 1.7) |
| paed. (QV), 25% | 8.5 | -0.4 (-0.4, -0.4) | -0.2 (-0.2, -0.2) | 7.9 (7.9, 8.0) | 17.2 | -11.6 (-12.0, -11.3) | -2.1 (-2.1, -2.0) | 3.5 (3.1, 3.9) |
| paed. (QV), 50% | 19.7 | -0.8 (-0.8, -0.7) | -0.5 (-0.5, -0.4) | 18.4 (18.4, 18.5) | 34.3 | -21.2 (-21.8, -20.6) | -3.9 (-4.0, -3.7) | 9.2 (8.5, 10.0) |
| paed. (QV), 75% | 30.8 | -1.1 (-1.1, -1.0) | -0.6 (-0.7, -0.6) | 29.1 (29.0, 29.2) | 51.5 | -27.9 (-28.8, -27.0) | -5.3 (-5.5, -5.1) | 18.2 (17.2, 19.4) |
| eld. (iTV) + paed. (TV), 10% | 14.8 | -0.2 (-0.2, -0.2) | -0.3 (-0.3, -0.3) | 14.4 (14.3, 14.4) | 34.0 | -5.0 (-5.1, -4.9) | -1.3 (-1.4, -1.3) | 27.7 (27.6, 27.9) |
| eld. (iTV) + paed. (TV), 25% | 20.4 | -0.5 (-0.5, -0.4) | -0.4 (-0.5, -0.4) | 19.5 (19.5, 19.6) | 42.1 | -11.4 (-11.6, -11.1) | -2.4 (-2.5, -2.4) | 28.3 (28.0, 28.6) |
| eld. (iTV) + paed. (TV), 50% | 30.3 | -0.8 (-0.9, -0.8) | -0.6 (-0.7, -0.6) | 28.9 (28.8, 29.0) | 55.5 | -19.9 (-20.4, -19.3) | -4.0 (-4.2, -3.9) | 31.6 (30.9, 32.3) |
| eld. (iTV) + paed. (TV), 75% | 40.2 | -1.1 (-1.1, -1.0) | -0.8 (-0.9, -0.7) | 38.3 (38.2, 38.5) | 68.9 | -26.3 (-27.1, -25.4) | -5.3 (-5.5, -5.1) | 37.3 (36.3, 38.4) |
| eld. (QV) + paed. (TV), 10% | 8.3 | -0.2 (-0.2, -0.1) | -0.2 (-0.2, -0.2) | 7.9 (7.9, 8.0) | 19.9 | -4.4 (-4.5, -4.3) | -0.9 (-1.0, -0.9) | 14.5 (14.4, 14.7) |
| eld. (QV) + paed. (TV), 25% | 13.9 | -0.4 (-0.4, -0.4) | -0.4 (-0.4, -0.3) | 13.1 (13.1, 13.2) | 28.0 | -10.9 (-11.1, -10.6) | -2.1 (-2.1, -2.0) | 15.1 (14.7, 15.4) |
| eld. (QV) + paed. (TV), 50% | 23.8 | -0.8 (-0.8, -0.7) | -0.6 (-0.6, -0.5) | 22.4 (22.4, 22.5) | 41.4 | -19.4 (-19.9, -18.8) | -3.7 (-3.8, -3.6) | 18.4 (17.7, 19.1) |
| eld. (QV) + paed. (TV), 75% | 33.7 | -1.0 (-1.1, -1.0) | -0.7 (-0.8, -0.7) | 31.9 (31.8, 32.0) | 54.9 | -25.8 (-26.6, -24.9) | -5.0 (-5.2, -4.8) | 24.1 (23.1, 25.2) |
| eld. (iTV) + paed. (QV), 10% | 15.5 | -0.2 (-0.2, -0.2) | -0.3 (-0.3, -0.3) | 15.0 (14.9, 15.0) | 35.5 | -5.5 (-5.6, -5.3) | -1.4 (-1.4, -1.4) | 28.7 (28.5, 28.8) |
| eld. (iTV) + paed. (QV), 25% | 21.8 | -0.5 (-0.5, -0.5) | -0.5 (-0.5, -0.4) | 20.9 (20.8, 20.9) | 45.8 | -12.6 (-12.9, -12.3) | -2.6 (-2.7, -2.6) | 30.5 (30.2, 30.9) |
| eld. (iTV) + paed. (QV), 50% | 33.0 | -0.9 (-0.9, -0.8) | -0.7 (-0.7, -0.6) | 31.4 (31.3, 31.5) | 63.0 | -22.1 (-22.7, -21.5) | -4.4 (-4.5, -4.2) | 36.5 (35.8, 37.2) |
| eld. (iTV) + paed. (QV), 75% | 44.1 | -1.2 (-1.2, -1.1) | -0.9 (-0.9, -0.8) | 42.1 (41.9, 42.2) | 80.1 | -28.8 (-29.7, -27.9) | -5.8 (-6.0, -5.6) | 45.6 (44.5, 46.7) |
| eld. (QV) + paed. (QV), 10% | 9.0 | -0.2 (-0.2, -0.2) | -0.2 (-0.3, -0.2) | 8.6 (8.5, 8.6) | 21.4 | -4.9 (-5.0, -4.8) | -1.0 (-1.1, -1.0) | 15.5 (15.3, 15.6) |
| eld. (QV) + paed. (QV), 25% | 15.3 | -0.5 (-0.5, -0.4) | -0.4 (-0.4, -0.4) | 14.4 (14.4, 14.5) | 31.7 | -12.1 (-12.4, -11.8) | -2.3 (-2.3, -2.2) | 17.3 (17.0, 17.7) |
| eld. (QV) + paed. (QV), 50% | 26.4 | -0.8 (-0.9, -0.8) | -0.6 (-0.7, -0.6) | 25.0 (24.9, 25.1) | 48.9 | -21.6 (-22.1, -20.9) | -4.1 (-4.2, -3.9) | 23.3 (22.6, 24.0) |
| eld. (QV) + paed. (QV), 75% | 37.6 | -1.1 (-1.2, -1.1) | -0.8 (-0.9, -0.7) | 35.6 (35.5, 35.8) | 66.0 | -28.2 (-29.1, -27.4) | -5.5 (-5.7, -5.2) | 32.3 (31.3, 33.4) |
| CrI: credible interval, eld.: elderly vaccination change (moving from TV to iTV or QV), GP: general practitioner, iTV: improved trivalent vaccine (i.e., adjuvanted or high-dose), paed.: paediatric mass vaccination (scenario with specified vaccine and uptake rate), QV: quadrivalent vaccine (non-adjuvanted, non-high dose), TIV: trivalent vaccine (non-adjuvanted, non-high dose).  Note: Negative values indicate cost savings as compared to the base case, positive values indicate cost increases. Potential total cost savings are indicated in bold. €1 = £0.88 (2017). | | | | | | | | |

| Supplementary Table 20: Mean (95%-CrI) change of the direct medical costs (in million €) as compared to the base case per setting and by cost category. | | | | | | | | |
| --- | --- | --- | --- | --- | --- | --- | --- | --- |
| **setting** | **Navarra (NV)** | | | | **France (FR)** | | | |
| **vaccination strategy** | **vaccination** | **GP visits** | **hospitalisations** | **total** | **vaccination** | **GP visits** | **hospitalisations** | **total** |
| elderly (iTV) | 0.4 | 0.0 (0.0, 0.0) | -0.1 (-0.2, -0.1) | 0.2 (0.2, 0.2) | 65.3 | -0.8 (-0.9, -0.8) | -1.1 (-1.2, -1.0) | 63.4 (63.2, 63.5) |
| elderly (QV) | 0.2 | 0.0 (0.0, 0.0) | -0.1 (-0.1, -0.1) | 0.1 (0.1, 0.1) | 31.1 | -0.5 (-0.5, -0.4) | -0.6 (-0.7, -0.5) | 30.1 (30.0, 30.2) |
| paed. (TV), 10% | 0.0 | 0.0 (0.0, 0.0) | 0.0 (0.0, 0.0) | 0.0 (0.0, 0.0) | 16.1 | -4.8 (-4.9, -4.7) | -2.4 (-2.4, -2.3) | 8.9 (8.8, 9.1) |
| paed. (TV), 25% | 0.2 | -0.1 (-0.1, -0.1) | -0.1 (-0.1, -0.1) | 0.0 (0.0, 0.0) | 40.3 | -12.1 (-12.4, -11.8) | -6.0 (-6.2, -5.8) | 22.2 (21.7, 22.6) |
| paed. (TV), 50% | 0.3 | -0.2 (-0.2, -0.2) | -0.2 (-0.2, -0.2) | -0.1 (-0.1, 0.0) | 81.2 | -23.6 (-24.6, -22.6) | -12.0 (-12.5, -11.4) | 45.6 (44.1, 47.2) |
| paed. (TV), 75% | 0.5 | -0.3 (-0.4, -0.3) | -0.3 (-0.3, -0.2) | -0.1 (-0.1, 0.0) | 122.4 | -29.4 (-30.4, -28.5) | -15.0 (-15.6, -14.4) | 77.9 (76.3, 79.4) |
| paed. (QV), 10% | 0.1 | 0.0 (0.0, 0.0) | 0.0 (0.0, 0.0) | 0.0 (0.0, 0.0) | 21.0 | -5.6 (-5.7, -5.4) | -2.7 (-2.7, -2.6) | 12.7 (12.5, 12.9) |
| paed. (QV), 25% | 0.2 | -0.1 (-0.1, -0.1) | -0.1 (-0.1, -0.1) | 0.0 (0.0, 0.0) | 51.5 | -13.9 (-14.2, -13.5) | -6.8 (-7.0, -6.5) | 30.9 (30.3, 31.5) |
| paed. (QV), 50% | 0.5 | -0.3 (-0.3, -0.3) | -0.2 (-0.2, -0.2) | 0.0 (0.0, 0.0) | 103.3 | -26.7 (-27.8, -25.7) | -13.4 (-14.0, -12.8) | 63.1 (61.5, 64.8) |
| paed. (QV), 75% | 0.7 | -0.4 (-0.4, -0.4) | -0.3 (-0.3, -0.3) | 0.0 (0.0, 0.0) | 155.3 | -33.4 (-34.4, -32.3) | -16.7 (-17.3, -16.1) | 105.2 (103.6, 106.8) |
| eld. (iTV) + paed. (TV), 10% | 0.5 | -0.1 (-0.1, -0.1) | -0.2 (-0.2, -0.2) | 0.2 (0.2, 0.2) | 81.4 | -5.6 (-5.8, -5.5) | -3.4 (-3.5, -3.3) | 72.4 (72.2, 72.6) |
| eld. (iTV) + paed. (TV), 25% | 0.6 | -0.1 (-0.1, -0.1) | -0.2 (-0.2, -0.2) | 0.2 (0.2, 0.2) | 105.6 | -12.9 (-13.2, -12.6) | -6.9 (-7.1, -6.7) | 85.8 (85.3, 86.3) |
| eld. (iTV) + paed. (TV), 50% | 0.8 | -0.3 (-0.3, -0.2) | -0.3 (-0.3, -0.3) | 0.2 (0.2, 0.2) | 146.5 | -24.3 (-25.3, -23.3) | -12.8 (-13.3, -12.2) | 109.5 (108.0, 111.0) |
| eld. (iTV) + paed. (TV), 75% | 0.9 | -0.4 (-0.4, -0.3) | -0.4 (-0.4, -0.4) | 0.2 (0.2, 0.2) | 187.7 | -30.0 (-31.0, -29.1) | -15.6 (-16.2, -15.0) | 142.0 (140.4, 143.5) |
| eld. (QV) + paed. (TV), 10% | 0.3 | 0.0 (0.0, 0.0) | -0.1 (-0.1, -0.1) | 0.1 (0.1, 0.1) | 47.2 | -5.3 (-5.4, -5.2) | -2.9 (-3.0, -2.8) | 39.0 (38.8, 39.2) |
| eld. (QV) + paed. (TV), 25% | 0.4 | -0.1 (-0.1, -0.1) | -0.2 (-0.2, -0.1) | 0.1 (0.1, 0.1) | 71.4 | -12.6 (-12.9, -12.3) | -6.5 (-6.7, -6.3) | 52.3 (51.8, 52.8) |
| eld. (QV) + paed. (TV), 50% | 0.6 | -0.2 (-0.3, -0.2) | -0.2 (-0.3, -0.2) | 0.1 (0.0, 0.1) | 112.3 | -24.1 (-25.1, -23.1) | -12.5 (-13.0, -11.9) | 75.8 (74.3, 77.3) |
| eld. (QV) + paed. (TV), 75% | 0.7 | -0.4 (-0.4, -0.3) | -0.3 (-0.4, -0.3) | 0.1 (0.0, 0.1) | 153.5 | -29.8 (-30.7, -28.9) | -15.3 (-15.9, -14.7) | 108.4 (106.9, 109.8) |
| eld. (iTV) + paed. (QV), 10% | 0.5 | -0.1 (-0.1, -0.1) | -0.2 (-0.2, -0.2) | 0.2 (0.2, 0.2) | 86.3 | -6.4 (-6.5, -6.2) | -3.7 (-3.8, -3.6) | 76.2 (76.0, 76.4) |
| eld. (iTV) + paed. (QV), 25% | 0.6 | -0.2 (-0.2, -0.2) | -0.2 (-0.2, -0.2) | 0.2 (0.2, 0.2) | 116.8 | -14.7 (-15.0, -14.3) | -7.7 (-7.9, -7.5) | 94.5 (93.9, 95.1) |
| eld. (iTV) + paed. (QV), 50% | 0.9 | -0.3 (-0.3, -0.3) | -0.3 (-0.3, -0.3) | 0.2 (0.2, 0.3) | 168.6 | -27.4 (-28.4, -26.4) | -14.1 (-14.7, -13.5) | 127.1 (125.4, 128.7) |
| eld. (iTV) + paed. (QV), 75% | 1.1 | -0.4 (-0.4, -0.4) | -0.4 (-0.5, -0.4) | 0.3 (0.2, 0.3) | 220.6 | -34.0 (-35.0, -32.9) | -17.3 (-17.9, -16.7) | 169.3 (167.8, 170.9) |
| eld. (QV) + paed. (QV), 10% | 0.3 | -0.1 (-0.1, -0.1) | -0.1 (-0.1, -0.1) | 0.1 (0.1, 0.1) | 52.1 | -6.0 (-6.2, -5.9) | -3.2 (-3.3, -3.1) | 42.8 (42.6, 43.0) |
| eld. (QV) + paed. (QV), 25% | 0.4 | -0.2 (-0.2, -0.1) | -0.2 (-0.2, -0.2) | 0.1 (0.1, 0.1) | 82.6 | -14.4 (-14.7, -14.0) | -7.3 (-7.5, -7.0) | 61.0 (60.4, 61.6) |
| eld. (QV) + paed. (QV), 50% | 0.7 | -0.3 (-0.3, -0.3) | -0.3 (-0.3, -0.2) | 0.1 (0.1, 0.1) | 134.4 | -27.2 (-28.2, -26.2) | -13.8 (-14.4, -13.2) | 93.4 (91.8, 95.0) |
| eld. (QV) + paed. (QV), 75% | 0.9 | -0.4 (-0.4, -0.4) | -0.4 (-0.4, -0.3) | 0.1 (0.1, 0.2) | 186.5 | -33.7 (-34.7, -32.7) | -17.0 (-17.6, -16.4) | 135.7 (134.2, 137.3) |
| CrI: credible interval, eld.: elderly vaccination change (moving from TV to iTV or QV), GP: general practitioner, iTV: improved trivalent vaccine (i.e., adjuvanted or high-dose), paed.: paediatric mass vaccination (scenario with specified vaccine and uptake rate), QV: quadrivalent vaccine (non-adjuvanted, non-high dose), TIV: trivalent vaccine (non-adjuvanted, non-high dose).  Note: Negative values indicate cost savings as compared to the base case, positive values indicate cost increases. Potential total cost savings are indicated in bold. €1 = £0.88 (2017). | | | | | | | | |

| Supplementary Table 21: Mean (95%-CrI) change of the direct medical costs (in million €) as compared to the base case per setting and by cost category. | | | | | | | | |
| --- | --- | --- | --- | --- | --- | --- | --- | --- |
| **setting** | **Netherlands (NL)** | | | | **England (EN)** | | | |
| **vaccination strategy** | **vaccination** | **GP visits** | **hospitalisations** | **total** | **vaccination** | **GP visits** | **hospitalisations** | **total** |
| elderly (iTV) | 14.2 | -0.4 (-0.4, -0.3) | -2.8 (-3.0, -2.6) | 11.0 (10.8, 11.2) | 62.9 | -1.4 (-1.5, -1.3) | -2.6 (-2.8, -2.4) | 58.9 (58.7, 59.2) |
| elderly (QV) | 7.1 | -0.2 (-0.3, -0.2) | -1.9 (-2.1, -1.7) | 4.9 (4.7, 5.1) | 24.1 | -1.1 (-1.2, -0.9) | -2.1 (-2.3, -1.9) | 20.9 (20.6, 21.2) |
| paed. (TV), 10% | 3.4 | -0.6 (-0.6, -0.6) | -1.9 (-1.9, -1.8) | 1.0 (0.9, 1.0) | 16.2 | -3.5 (-3.6, -3.4) | -2.7 (-2.8, -2.5) | 10.0 (9.8, 10.3) |
| paed. (TV), 25% | 8.6 | -1.5 (-1.5, -1.5) | -4.6 (-4.8, -4.4) | 2.4 (2.2, 2.7) | 40.5 | -8.6 (-9.0, -8.3) | -6.6 (-7.0, -6.3) | 25.2 (24.5, 25.9) |
| paed. (TV), 50% | 17.4 | -2.6 (-2.7, -2.5) | -8.4 (-8.8, -8.0) | 6.4 (5.9, 6.9) | 81.0 | -15.1 (-15.7, -14.5) | -11.6 (-12.2, -11.0) | 54.3 (53.1, 55.5) |
| paed. (TV), 75% | 26.5 | -3.2 (-3.4, -3.1) | -10.9 (-11.4, -10.4) | 12.3 (11.7, 12.9) | 125.4 | -18.7 (-19.4, -17.9) | -14.4 (-15.1, -13.7) | 92.3 (90.8, 93.7) |
| paed. (QV), 10% | 4.5 | -0.7 (-0.8, -0.7) | -2.2 (-2.3, -2.1) | 1.5 (1.4, 1.6) | 20.3 | -4.4 (-4.6, -4.3) | -3.3 (-3.5, -3.2) | 12.5 (12.3, 12.8) |
| paed. (QV), 25% | 10.6 | -1.8 (-1.8, -1.7) | -5.4 (-5.6, -5.2) | 3.5 (3.2, 3.7) | 48.3 | -10.5 (-10.9, -10.1) | -8.0 (-8.4, -7.6) | 29.8 (29.0, 30.6) |
| paed. (QV), 50% | 21.2 | -3.0 (-3.2, -2.9) | -9.6 (-10.0, -9.2) | 8.5 (8.0, 9.1) | 94.9 | -18.1 (-18.9, -17.4) | -14.0 (-14.7, -13.3) | 62.8 (61.3, 64.2) |
| paed. (QV), 75% | 32.1 | -3.9 (-4.0, -3.7) | -12.7 (-13.2, -12.1) | 15.5 (14.8, 16.3) | 145.9 | -22.5 (-23.5, -21.5) | -17.5 (-18.5, -16.6) | 105.8 (103.9, 107.8) |
| eld. (iTV) + paed. (TV), 10% | 17.6 | -0.9 (-1.0, -0.9) | -4.5 (-4.8, -4.3) | 12.2 (11.9, 12.4) | 79.1 | -4.9 (-5.0, -4.7) | -5.2 (-5.4, -4.9) | 69.1 (68.7, 69.5) |
| eld. (iTV) + paed. (TV), 25% | 22.8 | -1.8 (-1.9, -1.8) | -7.1 (-7.3, -6.8) | 13.9 (13.6, 14.2) | 103.4 | -10.0 (-10.4, -9.6) | -9.0 (-9.4, -8.5) | 84.5 (83.7, 85.3) |
| eld. (iTV) + paed. (TV), 50% | 31.6 | -2.9 (-3.0, -2.8) | -10.5 (-10.9, -10.0) | 18.2 (17.7, 18.8) | 143.9 | -16.3 (-16.9, -15.6) | -13.7 (-14.3, -13.1) | 114.0 (112.7, 115.2) |
| eld. (iTV) + paed. (TV), 75% | 40.7 | -3.5 (-3.6, -3.4) | -12.8 (-13.3, -12.2) | 24.4 (23.8, 25.1) | 188.3 | -19.7 (-20.5, -19.0) | -16.3 (-17.0, -15.6) | 152.2 (150.8, 153.7) |
| eld. (QV) + paed. (TV), 10% | 10.5 | -0.9 (-0.9, -0.8) | -3.8 (-3.9, -3.6) | 5.9 (5.7, 6.2) | 40.3 | -4.6 (-4.7, -4.4) | -4.7 (-5.0, -4.5) | 31.0 (30.6, 31.4) |
| eld. (QV) + paed. (TV), 25% | 15.7 | -1.7 (-1.8, -1.7) | -6.5 (-6.7, -6.2) | 7.5 (7.2, 7.8) | 64.6 | -9.7 (-10.1, -9.3) | -8.7 (-9.1, -8.2) | 46.2 (45.4, 47.0) |
| eld. (QV) + paed. (TV), 50% | 24.5 | -2.8 (-3.0, -2.7) | -10.2 (-10.6, -9.7) | 11.5 (11.0, 12.0) | 105.1 | -16.1 (-16.8, -15.5) | -13.5 (-14.2, -12.8) | 75.5 (74.1, 76.7) |
| eld. (QV) + paed. (TV), 75% | 33.6 | -3.5 (-3.6, -3.4) | -12.7 (-13.2, -12.1) | 17.4 (16.8, 18.1) | 149.4 | -19.6 (-20.4, -18.9) | -16.1 (-16.9, -15.4) | 113.7 (112.2, 115.2) |
| eld. (iTV) + paed. (QV), 10% | 18.7 | -1.1 (-1.1, -1.0) | -4.9 (-5.1, -4.6) | 12.7 (12.5, 13.0) | 83.2 | -5.8 (-6.0, -5.6) | -5.8 (-6.1, -5.6) | 71.6 (71.2, 72.0) |
| eld. (iTV) + paed. (QV), 25% | 24.8 | -2.1 (-2.2, -2.0) | -7.8 (-8.1, -7.5) | 14.9 (14.6, 15.3) | 111.2 | -11.8 (-12.2, -11.3) | -10.3 (-10.7, -9.8) | 89.1 (88.2, 90.0) |
| eld. (iTV) + paed. (QV), 50% | 35.4 | -3.3 (-3.5, -3.2) | -11.6 (-12.1, -11.1) | 20.4 (19.8, 21.0) | 157.8 | -19.3 (-20.1, -18.6) | -16.0 (-16.7, -15.3) | 122.5 (121.0, 123.9) |
| eld. (iTV) + paed. (QV), 75% | 46.3 | -4.1 (-4.3, -3.9) | -14.4 (-15.0, -13.8) | 27.7 (27.0, 28.5) | 208.8 | -23.7 (-24.7, -22.7) | -19.4 (-20.3, -18.4) | 165.7 (163.8, 167.6) |
| eld. (QV) + paed. (QV), 10% | 11.6 | -1.0 (-1.0, -1.0) | -4.1 (-4.3, -3.9) | 6.5 (6.3, 6.8) | 44.4 | -5.5 (-5.7, -5.3) | -5.4 (-5.6, -5.1) | 33.5 (33.1, 34.0) |
| eld. (QV) + paed. (QV), 25% | 17.7 | -2.0 (-2.1, -2.0) | -7.2 (-7.5, -6.9) | 8.5 (8.2, 8.9) | 72.3 | -11.5 (-12.0, -11.1) | -9.9 (-10.4, -9.5) | 50.9 (50.0, 51.7) |
| eld. (QV) + paed. (QV), 50% | 28.3 | -3.3 (-3.4, -3.2) | -11.3 (-11.8, -10.8) | 13.7 (13.1, 14.3) | 118.9 | -19.2 (-20.0, -18.4) | -15.8 (-16.5, -15.0) | 83.9 (82.4, 85.5) |
| eld. (QV) + paed. (QV), 75% | 39.2 | -4.1 (-4.3, -3.9) | -14.3 (-14.9, -13.6) | 20.8 (20.0, 21.6) | 170.0 | -23.6 (-24.6, -22.6) | -19.1 (-20.1, -18.2) | 127.2 (125.3, 129.1) |
| CrI: credible interval, eld.: elderly vaccination change (moving from TV to iTV or QV), GP: general practitioner, iTV: improved trivalent vaccine (i.e., adjuvanted or high-dose), paed.: paediatric mass vaccination (scenario with specified vaccine and uptake rate), QV: quadrivalent vaccine (non-adjuvanted, non-high dose), TIV: trivalent vaccine (non-adjuvanted, non-high dose).  Note: Negative values indicate cost savings as compared to the base case, positive values indicate cost increases. Potential total cost savings are indicated in bold. €1 = £0.88 (2017). | | | | | | | | |

| Supplementary Table 22: Mean (95%-CrI) change of the direct medical costs (in million €) as compared to the base case per setting and by cost category. | | | | | | | | |
| --- | --- | --- | --- | --- | --- | --- | --- | --- |
| **setting** | **Scotland (SC)** | | | | **Ireland (IE)** | | | |
| **vaccination strategy** | **vaccination** | **GP visits** | **hospitalisations** | **total** | **vaccination** | **GP visits** | **hospitalisations** | **total** |
| elderly (iTV) | 6.7 | -0.1 (-0.1, -0.1) | -0.5 (-0.6, -0.5) | 6.1 (6.0, 6.1) | 2.2 | 0.0 (0.0, 0.0) | -0.3 (-0.4, -0.3) | 1.8 (1.8, 1.8) |
| elderly (QV) | 2.6 | -0.1 (-0.1, -0.1) | -0.3 (-0.4, -0.3) | 2.2 (2.1, 2.2) | 0.8 | 0.0 (0.0, 0.0) | -0.2 (-0.2, -0.1) | 0.6 (0.6, 0.6) |
| paed. (TV), 10% | 1.4 | -0.1 (-0.1, 0.0) | -0.2 (-0.2, -0.1) | 1.1 (1.1, 1.2) | 2.6 | 0.0 (0.0, 0.0) | -0.4 (-0.4, -0.4) | 2.2 (2.2, 2.2) |
| paed. (TV), 25% | 3.5 | -0.1 (-0.1, -0.1) | -0.5 (-0.5, -0.3) | 2.9 (2.8, 3.1) | 6.5 | -0.1 (-0.1, -0.1) | -1.0 (-1.0, -0.9) | 5.4 (5.4, 5.5) |
| paed. (TV), 50% | 7.0 | -0.2 (-0.2, -0.2) | -0.8 (-0.9, -0.7) | 6.0 (5.8, 6.1) | 13.1 | -0.2 (-0.2, -0.2) | -1.6 (-1.6, -1.5) | 11.3 (11.2, 11.4) |
| paed. (TV), 75% | 10.8 | -0.3 (-0.3, -0.3) | -1.1 (-1.2, -1.0) | 9.4 (9.3, 9.5) | 19.6 | -0.2 (-0.2, -0.2) | -2.0 (-2.1, -1.9) | 17.4 (17.3, 17.5) |
| paed. (QV), 10% | 1.8 | -0.1 (-0.1, 0.0) | -0.2 (-0.3, -0.2) | 1.5 (1.4, 1.6) | 2.8 | -0.1 (-0.1, -0.1) | -0.4 (-0.5, -0.4) | 2.3 (2.3, 2.3) |
| paed. (QV), 25% | 4.1 | -0.1 (-0.2, -0.1) | -0.5 (-0.6, -0.4) | 3.5 (3.4, 3.6) | 6.9 | -0.1 (-0.1, -0.1) | -1.1 (-1.2, -1.1) | 5.7 (5.7, 5.8) |
| paed. (QV), 50% | 8.1 | -0.2 (-0.3, -0.2) | -0.9 (-1.0, -0.8) | 6.9 (6.8, 7.1) | 13.9 | -0.2 (-0.2, -0.2) | -1.8 (-1.9, -1.7) | 11.9 (11.8, 12.0) |
| paed. (QV), 75% | 12.4 | -0.3 (-0.3, -0.3) | -1.2 (-1.3, -1.1) | 10.9 (10.8, 11.0) | 20.8 | -0.3 (-0.3, -0.3) | -2.4 (-2.5, -2.2) | 18.2 (18.1, 18.4) |
| eld. (iTV) + paed. (TV), 10% | 8.1 | -0.1 (-0.2, -0.1) | -0.7 (-0.8, -0.6) | 7.2 (7.2, 7.3) | 4.8 | -0.1 (-0.1, -0.1) | -0.7 (-0.7, -0.7) | 4.0 (3.9, 4.0) |
| eld. (iTV) + paed. (TV), 25% | 10.2 | -0.2 (-0.2, -0.2) | -1.0 (-1.1, -0.8) | 9.0 (8.9, 9.2) | 8.7 | -0.1 (-0.2, -0.1) | -1.3 (-1.3, -1.2) | 7.3 (7.2, 7.4) |
| eld. (iTV) + paed. (TV), 50% | 13.7 | -0.3 (-0.3, -0.3) | -1.3 (-1.4, -1.2) | 12.1 (12.0, 12.2) | 15.2 | -0.2 (-0.2, -0.2) | -1.8 (-1.9, -1.7) | 13.2 (13.1, 13.3) |
| eld. (iTV) + paed. (TV), 75% | 17.5 | -0.4 (-0.4, -0.3) | -1.6 (-1.7, -1.5) | 15.5 (15.4, 15.7) | 21.8 | -0.3 (-0.3, -0.2) | -2.2 (-2.3, -2.1) | 19.3 (19.2, 19.4) |
| eld. (QV) + paed. (TV), 10% | 4.0 | -0.1 (-0.1, -0.1) | -0.5 (-0.6, -0.5) | 3.3 (3.3, 3.4) | 3.4 | -0.1 (-0.1, -0.1) | -0.5 (-0.6, -0.5) | 2.8 (2.8, 2.8) |
| eld. (QV) + paed. (TV), 25% | 6.1 | -0.2 (-0.2, -0.1) | -0.8 (-0.9, -0.7) | 5.1 (5.0, 5.2) | 7.3 | -0.1 (-0.1, -0.1) | -1.1 (-1.2, -1.0) | 6.1 (6.0, 6.1) |
| eld. (QV) + paed. (TV), 50% | 9.5 | -0.3 (-0.3, -0.2) | -1.1 (-1.2, -1.0) | 8.2 (8.0, 8.3) | 13.8 | -0.2 (-0.2, -0.2) | -1.7 (-1.8, -1.6) | 11.9 (11.9, 12.1) |
| eld. (QV) + paed. (TV), 75% | 13.3 | -0.3 (-0.4, -0.3) | -1.4 (-1.5, -1.3) | 11.6 (11.5, 11.7) | 20.4 | -0.2 (-0.3, -0.2) | -2.1 (-2.2, -2.0) | 18.0 (17.9, 18.2) |
| eld. (iTV) + paed. (QV), 10% | 8.5 | -0.1 (-0.2, -0.1) | -0.8 (-0.8, -0.7) | 7.6 (7.5, 7.7) | 4.9 | -0.1 (-0.1, -0.1) | -0.8 (-0.8, -0.7) | 4.1 (4.0, 4.1) |
| eld. (iTV) + paed. (QV), 25% | 10.8 | -0.2 (-0.2, -0.2) | -1.0 (-1.1, -0.9) | 9.6 (9.5, 9.7) | 9.1 | -0.2 (-0.2, -0.2) | -1.4 (-1.5, -1.3) | 7.5 (7.5, 7.6) |
| eld. (iTV) + paed. (QV), 50% | 14.8 | -0.3 (-0.3, -0.3) | -1.4 (-1.5, -1.3) | 13.1 (12.9, 13.2) | 16.1 | -0.2 (-0.3, -0.2) | -2.1 (-2.2, -2.0) | 13.8 (13.7, 13.9) |
| eld. (iTV) + paed. (QV), 75% | 19.1 | -0.4 (-0.4, -0.4) | -1.7 (-1.8, -1.5) | 17.0 (16.9, 17.2) | 23.0 | -0.3 (-0.3, -0.3) | -2.6 (-2.7, -2.4) | 20.1 (20.0, 20.3) |
| eld. (QV) + paed. (QV), 10% | 4.3 | -0.1 (-0.1, -0.1) | -0.6 (-0.6, -0.5) | 3.7 (3.6, 3.7) | 3.6 | -0.1 (-0.1, -0.1) | -0.6 (-0.6, -0.6) | 2.9 (2.9, 2.9) |
| eld. (QV) + paed. (QV), 25% | 6.7 | -0.2 (-0.2, -0.2) | -0.8 (-0.9, -0.7) | 5.7 (5.6, 5.8) | 7.7 | -0.1 (-0.2, -0.1) | -1.2 (-1.3, -1.2) | 6.3 (6.3, 6.4) |
| eld. (QV) + paed. (QV), 50% | 10.6 | -0.3 (-0.3, -0.3) | -1.2 (-1.3, -1.1) | 9.1 (9.0, 9.3) | 14.7 | -0.2 (-0.2, -0.2) | -1.9 (-2.0, -1.8) | 12.5 (12.4, 12.6) |
| eld. (QV) + paed. (QV), 75% | 14.9 | -0.4 (-0.4, -0.3) | -1.5 (-1.6, -1.3) | 13.1 (13.0, 13.3) | 21.6 | -0.3 (-0.3, -0.3) | -2.4 (-2.6, -2.3) | 18.9 (18.8, 19.0) |
| CrI: credible interval, eld.: elderly vaccination change (moving from TV to iTV or QV), GP: general practitioner, iTV: improved trivalent vaccine (i.e., adjuvanted or high-dose), paed.: paediatric mass vaccination (scenario with specified vaccine and uptake rate), QV: quadrivalent vaccine (non-adjuvanted, non-high dose), TIV: trivalent vaccine (non-adjuvanted, non-high dose).  Note: Negative values indicate cost savings as compared to the base case, positive values indicate cost increases. Potential total cost savings are indicated in bold. €1 = £0.88 (2017). | | | | | | | | |

QALY gains by category per setting

Detailed changes in the QALY loss averted as compared to the base case per setting (see Supplementary Figure 57B).


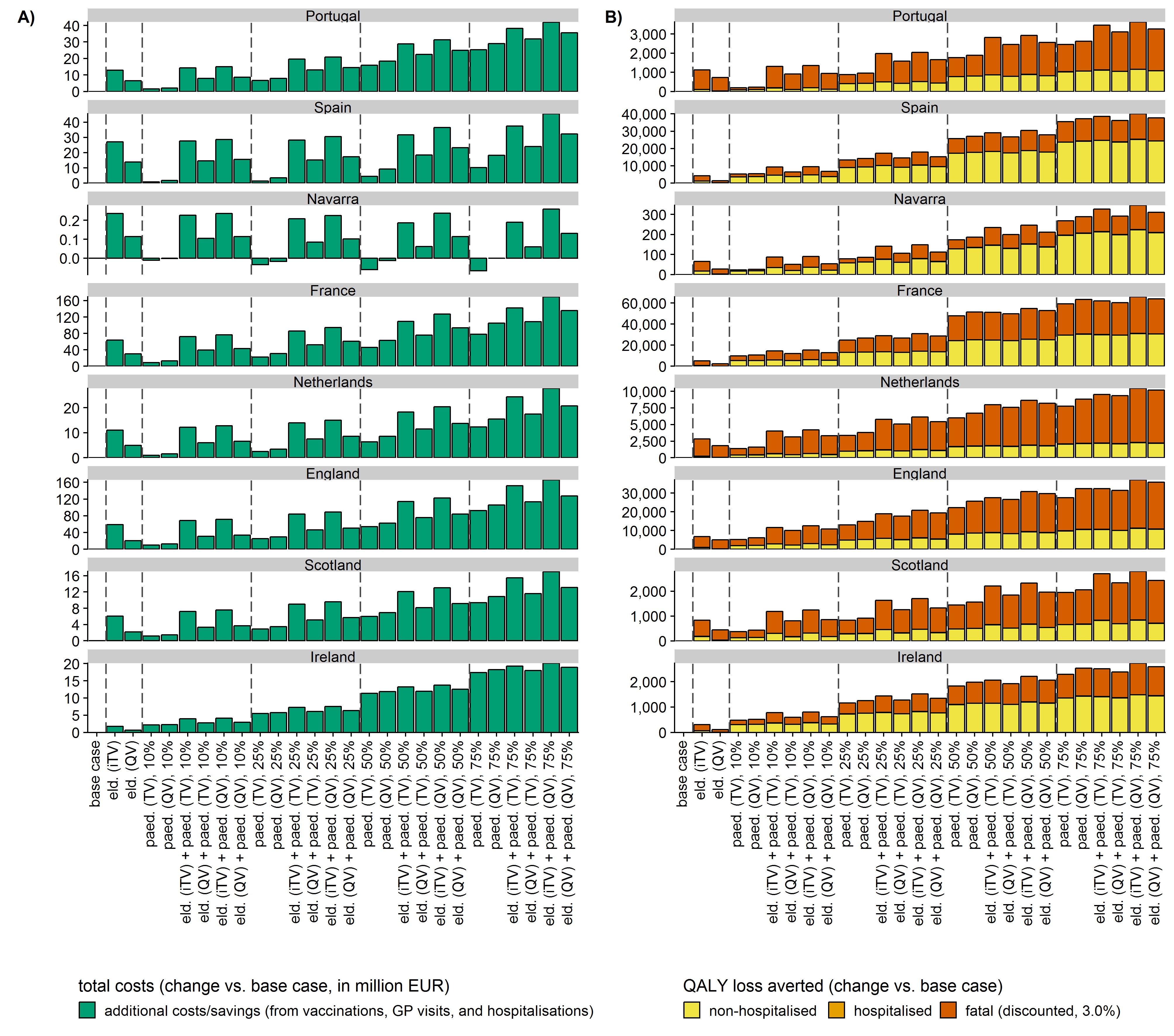
Supplementary Figure 57. Change in total costs (panel A) and total QALYs (panel B) per strategy as compared to the base case.

| Supplementary Table 23: Mean (95%-CrI) change of the QALY loss averted as compared to the base case per setting. | | | | | | | |  |
| --- | --- | --- | --- | --- | --- | --- | --- | --- |
| **vaccination strategy** | **symptomatic cases** | | **hospitalisations** | **excess deaths** | | | **total** |  |
| **setting** | **Portugal (PT)** | | | | | | |  |
| elderly (iTV) | 97.2 (87.0, 110) | | 1.7 (1.5, 1.9) | 1000 (930, 1100) | | | 1100 (1000, 1200) |  |
| elderly (QV) | 17.0 (14.3, 19.4) | | 1.2 (1.0, 1.3) | 710 (580, 810) | | | 720 (600, 840) |  |
| paed. (TV), 10% | 87.3 (83.4, 90.8) | | 0.3 (0.3, 0.3) | 100 (86.7, 110) | | | 190 (170, 200) |  |
| paed. (TV), 25% | 410 (390, 430) | | 1.4 (1.3, 1.5) | 480 (420, 520) | | | 890 (820, 940) |  |
| paed. (TV), 50% | 770 ( 720, 820) | | 2.9 (2.6, 3.1) | 990 ( 890, 1100) | | | 1800 (1600, 1900) |  |
| paed. (TV), 75% | 1000 ( 960, 1100) | | 4.0 (3.7, 4.3) | 1400 (1300, 1600) | | | 2500 (2300, 2600) |  |
| paed. (QV), 10% | 94.6 (90.6, 98.3) | | 0.4 (0.4, 0.4) | 130 (110, 150) | | | 230 (210, 240) |  |
| paed. (QV), 25% | 420 (400, 440) | | 1.6 (1.4, 1.7) | 530 (470, 580) | | | 950 (880, 1000) |  |
| paed. (QV), 50% | 800 ( 750, 840) | | 3.1 (2.9, 3.4) | 1100 ( 970, 1200) | | | 1900 (1700, 2000) |  |
| paed. (QV), 75% | 1100 ( 990, 1100) | | 4.4 (4.0, 4.7) | 1600 (1400, 1700) | | | 2600 (2400, 2800) |  |
| eld. (iTV) + paed. (TV), 10% | 180 ( 170, 190) | | 2.0 (1.8, 2.2) | 1100 (1000, 1200) | | | 1300 (1200, 1400) |  |
| eld. (iTV) + paed. (TV), 25% | 500 ( 480, 520) | | 3.0 (2.8, 3.2) | 1500 (1300, 1600) | | | 2000 (1800, 2100) |  |
| eld. (iTV) + paed. (TV), 50% | 860 ( 810, 910) | | 4.4 (4.1, 4.7) | 1900 (1800, 2100) | | | 2800 (2600, 3000) |  |
| eld. (iTV) + paed. (TV), 75% | 1100 (1000, 1200) | | 5.5 (5.1, 5.9) | 2300 (2100, 2500) | | | 3500 (3200, 3700) |  |
| eld. (QV) + paed. (TV), 10% | 100 (99.5, 110) | | 1.5 (1.2, 1.6) | 800 (670, 920) | | | 910 (780, 1000) |  |
| eld. (QV) + paed. (TV), 25% | 420 ( 400, 440) | | 2.5 (2.3, 2.8) | 1200 (1000, 1300) | | | 1600 (1400, 1700) |  |
| eld. (QV) + paed. (TV), 50% | 790 ( 740, 840) | | 3.9 (3.6, 4.2) | 1700 (1500, 1800) | | | 2500 (2200, 2600) |  |
| eld. (QV) + paed. (TV), 75% | 1000 ( 970, 1100) | | 5.0 (4.6, 5.4) | 2100 (1800, 2300) | | | 3100 (2900, 3300) |  |
| eld. (iTV) + paed. (QV), 10% | 190 ( 180, 200) | | 2.1 (1.9, 2.2) | 1200 (1000, 1300) | | | 1300 (1200, 1500) |  |
| eld. (iTV) + paed. (QV), 25% | 520 ( 490, 540) | | 3.2 (3.0, 3.4) | 1500 (1400, 1700) | | | 2000 (1900, 2200) |  |
| eld. (iTV) + paed. (QV), 50% | 890 ( 840, 940) | | 4.7 (4.4, 5.0) | 2000 (1900, 2200) | | | 2900 (2700, 3100) |  |
| eld. (iTV) + paed. (QV), 75% | 1100 (1100, 1200) | | 5.9 (5.4, 6.3) | 2500 (2300, 2700) | | | 3600 (3400, 3900) |  |
| eld. (QV) + paed. (QV), 10% | 110 (110, 120) | | 1.5 (1.3, 1.7) | 830 (700, 950) | | | 940 (810, 1100) |  |
| eld. (QV) + paed. (QV), 25% | 440 ( 420, 460) | | 2.7 (2.4, 2.9) | 1200 (1100, 1400) | | | 1700 (1500, 1800) |  |
| eld. (QV) + paed. (QV), 50% | 810 ( 760, 860) | | 4.2 (3.8, 4.5) | 1700 (1500, 1900) | | | 2600 (2300, 2800) |  |
| eld. (QV) + paed. (QV), 75% | 1100 (1000, 1100) | | 5.4 (4.9, 5.8) | 2200 (1900, 2400) | | | 3300 (3000, 3500) |  |
| **setting** | **Spain (ES)** | | | | | | |  |
| elderly (iTV) | 1100 (1000, 1100) | | 6.2 (5.9, 6.5) | 3100 (3000, 3200) | | | 4200 (4000, 4400) |  |
| elderly (QV) | 110 ( 100, 120) | | 2.5 (2.3, 2.7) | 1200 (1100, 1300) | | | 1400 (1200, 1500) |  |
| paed. (TV), 10% | 3500 (3500, 3600) | | 6.6 (6.4, 6.8) | 1600 (1600, 1700) | | | 5200 (5000, 5300) |  |
| paed. (TV), 25% | 9000 ( 8800, 9200) | | 17.4 (16.8, 18.0) | 4300 ( 4100, 4600) | | | 13000 (13000, 14000) |  |
| paed. (TV), 50% | 17000 (17000, 18000) | | 33.4 (32.1, 34.6) | 8500 ( 8000, 8900) | | | 26000 (25000, 27000) |  |
| paed. (TV), 75% | 24000 (23000, 24000) | | 46.3 (44.2, 48.2) | 12000 (11000, 12000) | | | 35000 (34000, 37000) |  |
| paed. (QV), 10% | 3600 (3600, 3700) | | 7.3 (7.1, 7.6) | 1800 (1700, 1900) | | | 5500 (5300, 5600) |  |
| paed. (QV), 25% | 9300 ( 9100, 9500) | | 19.5 (18.8, 20.2) | 4800 ( 4600, 5100) | | | 14000 (14000, 15000) |  |
| paed. (QV), 50% | 18000 (17000, 18000) | | 37.0 (35.5, 38.3) | 9300 ( 8800, 9800) | | | 27000 (26000, 28000) |  |
| paed. (QV), 75% | 24000 (23000, 25000) | | 50.7 (48.5, 52.7) | 13000 (12000, 14000) | | | 37000 (36000, 39000) |  |
| eld. (iTV) + paed. (TV), 10% | 4600 (4500, 4700) | | 12.6 (12.2, 12.9) | 4600 (4400, 4800) | | | 9200 (9000, 9500) |  |
| eld. (iTV) + paed. (TV), 25% | 10000 ( 9900, 10000) | | 23.1 (22.4, 23.7) | 7100 ( 6800, 7400) | | | 17000 (17000, 18000) |  |
| eld. (iTV) + paed. (TV), 50% | 18000 (18000, 19000) | | 38.4 (37.1, 39.6) | 11000 (10000, 11000) | | | 29000 (28000, 30000) |  |
| eld. (iTV) + paed. (TV), 75% | 25000 (24000, 26000) | | 50.7 (48.7, 52.6) | 14000 (13000, 15000) | | | 39000 (37000, 40000) |  |
| eld. (QV) + paed. (TV), 10% | 3600 (3600, 3700) | | 9.0 (8.7, 9.3) | 2800 (2600, 2900) | | | 6400 (6300, 6600) |  |
| eld. (QV) + paed. (TV), 25% | 9100 ( 8900, 9300) | | 19.7 (19.0, 20.3) | 5400 ( 5100, 5600) | | | 15000 (14000, 15000) |  |
| eld. (QV) + paed. (TV), 50% | 17000 (17000, 18000) | | 35.2 (33.9, 36.4) | 9300 ( 8800, 9800) | | | 27000 (26000, 28000) |  |
| eld. (QV) + paed. (TV), 75% | 24000 (23000, 25000) | | 47.8 (45.7, 49.7) | 12000 (12000, 13000) | | | 36000 (35000, 38000) |  |
| eld. (iTV) + paed. (QV), 10% | 4700 (4600, 4800) | | 13.3 (12.9, 13.7) | 4800 (4600, 5000) | | | 9500 (9200, 9700) |  |
| eld. (iTV) + paed. (QV), 25% | 10000 (10000, 11000) | | 25.1 (24.3, 25.8) | 7500 ( 7200, 7900) | | | 18000 (17000, 18000) |  |
| eld. (iTV) + paed. (QV), 50% | 19000 (18000, 19000) | | 41.8 (40.3, 43.1) | 12000 (11000, 12000) | | | 30000 (29000, 31000) |  |
| eld. (iTV) + paed. (QV), 75% | 25000 (24000, 26000) | | 54.9 (52.8, 56.9) | 15000 (14000, 16000) | | | 40000 (39000, 42000) |  |
| eld. (QV) + paed. (QV), 10% | 3800 (3700, 3800) | | 9.7 (9.4, 10.0) | 2900 (2800, 3100) | | | 6700 (6500, 6900) |  |
| eld. (QV) + paed. (QV), 25% | 9400 ( 9200, 9600) | | 21.6 (20.9, 22.3) | 5800 ( 5500, 6100) | | | 15000 (15000, 16000) |  |
| eld. (QV) + paed. (QV), 50% | 18000 (17000, 18000) | | 38.5 (37.1, 39.8) | 10000 ( 9400, 10000) | | | 28000 (27000, 29000) |  |
| eld. (QV) + paed. (QV), 75% | 24000 (23000, 25000) | | 51.9 (49.8, 53.8) | 13000 (13000, 14000) | | | 38000 (36000, 39000) |  |
| CrI: credible interval, eld.: elderly vaccination change, GP: general practitioner, iTV: improved trivalent vaccine (i.e., adjuvanted or high-dose), paed.: paediatric mass vaccination, QV: quadrivalent vaccine (non-adjuvanted, non-high dose), TV: trivalent vaccine (non-adjuvanted, non-high dose). | | | | | | | |  |
| Supplementary Table 24: Mean (95%-CrI) change of the QALY loss averted as compared to the base case per setting. | | | | | | | |  |
| **vaccination strategy** | **symptomatic cases** | **hospitalisations** | | | **excess deaths** | **total** | |  |
| **setting** | **Navarra (NV)** | | | | | | |  |
| elderly (iTV) | 17.1 (16.2, 17.9) | 0.7 (0.7, 0.8) | | | 47.1 (44.9, 49.3) | 64.9 (61.9, 67.9) | |  |
| elderly (QV) | 2.2 (2.0, 2.4) | 0.4 (0.4, 0.4) | | | 25.4 (22.9, 27.7) | 27.9 (25.2, 30.5) | |  |
| paed. (TV), 10% | 17.5 (16.8, 18.1) | 0.1 (0.1, 0.1) | | | 5.9 (5.5, 6.3) | 23.5 (22.5, 24.4) | |  |
| paed. (TV), 25% | 58.5 (56.2, 60.5) | 0.4 (0.4, 0.4) | | | 20.1 (18.8, 21.3) | 79.0 (75.9, 81.9) | |  |
| paed. (TV), 50% | 130 (120, 130) | 0.9 (0.9, 1.0) | | | 45.1 (41.6, 47.9) | 170 (160, 180) | |  |
| paed. (TV), 75% | 200 (180, 210) | 1.4 (1.3, 1.5) | | | 70.6 (64.0, 76.3) | 270 (250, 290) | |  |
| paed. (QV), 10% | 19.0 (18.3, 19.6) | 0.2 (0.1, 0.2) | | | 7.3 (6.8, 7.8) | 26.4 (25.4, 27.4) | |  |
| paed. (QV), 25% | 62.4 (60.1, 64.5) | 0.5 (0.5, 0.5) | | | 23.7 (22.1, 25.2) | 86.6 (83.2, 89.8) | |  |
| paed. (QV), 50% | 130 (130, 140) | 1.1 (1.0, 1.1) | | | 51.6 (47.6, 54.8) | 190 (180, 190) | |  |
| paed. (QV), 75% | 210 (190, 220) | 1.7 (1.5, 1.8) | | | 81.2 (73.9, 87.4) | 290 (270, 310) | |  |
| eld. (iTV) + paed. (TV), 10% | 34.5 (33.4, 35.6) | 0.9 (0.8, 0.9) | | | 52.5 (50.1, 54.8) | 87.9 (84.6, 91.1) | |  |
| eld. (iTV) + paed. (TV), 25% | 75.5 (73.1, 77.7) | 1.1 (1.1, 1.2) | | | 65.4 (62.5, 68.2) | 140 (140, 150) | |  |
| eld. (iTV) + paed. (TV), 50% | 150 (140, 150) | 1.6 (1.5, 1.7) | | | 88.2 (83.8, 92.2) | 230 (220, 240) | |  |
| eld. (iTV) + paed. (TV), 75% | 210 (200, 230) | 2.1 (2.0, 2.2) | | | 110 (100, 120) | 330 (310, 350) | |  |
| eld. (QV) + paed. (TV), 10% | 19.7 (18.9, 20.3) | 0.5 (0.5, 0.6) | | | 30.9 (28.3, 33.4) | 51.1 (48.1, 53.9) | |  |
| eld. (QV) + paed. (TV), 25% | 60.6 (58.4, 62.7) | 0.8 (0.7, 0.8) | | | 44.3 (41.3, 47.1) | 110 (100, 110) | |  |
| eld. (QV) + paed. (TV), 50% | 130 (120, 130) | 1.3 (1.2, 1.3) | | | 68.0 (63.5, 71.9) | 200 (190, 210) | |  |
| eld. (QV) + paed. (TV), 75% | 200 (180, 210) | 1.8 (1.6, 1.9) | | | 92.3 (85.3, 98.7) | 290 (270, 310) | |  |
| eld. (iTV) + paed. (QV), 10% | 36.0 (34.9, 37.1) | 0.9 (0.8, 0.9) | | | 53.8 (51.3, 56.2) | 90.7 (87.3, 94.0) | |  |
| eld. (iTV) + paed. (QV), 25% | 79.4 (76.9, 81.6) | 1.2 (1.1, 1.2) | | | 68.7 (65.6, 71.7) | 150 (140, 150) | |  |
| eld. (iTV) + paed. (QV), 50% | 150 (140, 160) | 1.7 (1.6, 1.8) | | | 94.1 (89.3, 98.5) | 250 (240, 260) | |  |
| eld. (iTV) + paed. (QV), 75% | 220 (210, 240) | 2.3 (2.2, 2.4) | | | 120 (110, 130) | 350 (330, 370) | |  |
| eld. (QV) + paed. (QV), 10% | 21.1 (20.4, 21.8) | 0.5 (0.5, 0.6) | | | 32.1 (29.4, 34.6) | 53.8 (50.7, 56.7) | |  |
| eld. (QV) + paed. (QV), 25% | 64.6 (62.3, 66.7) | 0.9 (0.8, 0.9) | | | 47.3 (44.1, 50.4) | 110 (110, 120) | |  |
| eld. (QV) + paed. (QV), 50% | 140 (130, 140) | 1.4 (1.3, 1.5) | | | 73.5 (68.5, 77.9) | 210 (200, 220) | |  |
| eld. (QV) + paed. (QV), 75% | 210 (190, 220) | 2.0 (1.8, 2.1) | | | 100 (93.6, 110) | 310 (290, 330) | |  |
| **setting** | **France (FR)** | | | | | | |  |
| elderly (iTV) | 720 ( 670, 770) | 5.3 (5.0, 5.6) | | | 4400 (4100, 4600) | 5100 (4800, 5400) | |  |
| elderly (QV) | 110 (98.5, 120) | 2.8 (2.5, 3.1) | | | 2300 (2000, 2500) | 2400 (2100, 2600) | |  |
| paed. (TV), 10% | 5200 (5100, 5300) | 11.2 (10.9, 11.5) | | | 4600 (4400, 4800) | 9800 (9500, 10000) | |  |
| paed. (TV), 25% | 13000 (13000, 13000) | 28.5 (27.6, 29.3) | | | 12000 (11000, 12000) | 25000 (24000, 26000) | |  |
| paed. (TV), 50% | 24000 (23000, 25000) | 56.9 (54.2, 59.5) | | | 24000 (22000, 25000) | 48000 (46000, 50000) | |  |
| paed. (TV), 75% | 29000 (28000, 30000) | 71.3 (68.4, 74.2) | | | 30000 (28000, 32000) | 59000 (57000, 62000) | |  |
| paed. (QV), 10% | 5400 (5300, 5500) | 12.7 (12.3, 13.0) | | | 5200 (4900, 5400) | 11000 (10000, 11000) | |  |
| paed. (QV), 25% | 13000 (13000, 14000) | 32.2 (31.1, 33.2) | | | 13000 (13000, 14000) | 27000 (26000, 28000) | |  |
| paed. (QV), 50% | 25000 (24000, 26000) | 63.7 (60.8, 66.6) | | | 27000 (25000, 28000) | 52000 (49000, 54000) | |  |
| paed. (QV), 75% | 30000 (29000, 31000) | 79.5 (76.6, 82.3) | | | 33000 (31000, 34000) | 63000 (61000, 66000) | |  |
| eld. (iTV) + paed. (TV), 10% | 5900 ( 5800, 6000) | 16.2 (15.7, 16.7) | | | 8700 ( 8200, 9100) | 15000 (14000, 15000) | |  |
| eld. (iTV) + paed. (TV), 25% | 14000 (13000, 14000) | 32.9 (31.9, 33.9) | | | 15000 (15000, 16000) | 29000 (28000, 30000) | |  |
| eld. (iTV) + paed. (TV), 50% | 25000 (24000, 25000) | 60.6 (57.8, 63.1) | | | 27000 (25000, 28000) | 51000 (49000, 53000) | |  |
| eld. (iTV) + paed. (TV), 75% | 30000 (29000, 31000) | 74.3 (71.4, 77.1) | | | 32000 (30000, 34000) | 62000 (60000, 64000) | |  |
| eld. (QV) + paed. (TV), 10% | 5300 ( 5200, 5400) | 13.9 (13.4, 14.3) | | | 6700 ( 6400, 7100) | 12000 (12000, 12000) | |  |
| eld. (QV) + paed. (TV), 25% | 13000 (13000, 13000) | 31.0 (30.0, 31.9) | | | 14000 (13000, 14000) | 27000 (26000, 28000) | |  |
| eld. (QV) + paed. (TV), 50% | 24000 (23000, 25000) | 59.2 (56.5, 61.7) | | | 25000 (24000, 27000) | 50000 (48000, 52000) | |  |
| eld. (QV) + paed. (TV), 75% | 29000 (28000, 30000) | 72.8 (70.0, 75.5) | | | 31000 (29000, 33000) | 60000 (58000, 63000) | |  |
| eld. (iTV) + paed. (QV), 10% | 6100 ( 6000, 6200) | 17.7 (17.1, 18.2) | | | 9200 ( 8700, 9600) | 15000 (15000, 16000) | |  |
| eld. (iTV) + paed. (QV), 25% | 14000 (14000, 14000) | 36.6 (35.4, 37.7) | | | 17000 (16000, 18000) | 31000 (30000, 32000) | |  |
| eld. (iTV) + paed. (QV), 50% | 26000 (25000, 26000) | 67.1 (64.2, 70.0) | | | 29000 (27000, 31000) | 55000 (52000, 57000) | |  |
| eld. (iTV) + paed. (QV), 75% | 31000 (30000, 32000) | 82.3 (79.4, 85.1) | | | 35000 (33000, 37000) | 66000 (63000, 68000) | |  |
| eld. (QV) + paed. (QV), 10% | 5500 ( 5400, 5600) | 15.3 (14.8, 15.8) | | | 7300 ( 6900, 7600) | 13000 (12000, 13000) | |  |
| eld. (QV) + paed. (QV), 25% | 14000 (13000, 14000) | 34.6 (33.4, 35.7) | | | 15000 (14000, 16000) | 29000 (28000, 30000) | |  |
| eld. (QV) + paed. (QV), 50% | 25000 (24000, 26000) | 65.5 (62.7, 68.3) | | | 28000 (26000, 29000) | 53000 (51000, 55000) | |  |
| eld. (QV) + paed. (QV), 75% | 31000 (29000, 32000) | 80.7 (77.8, 83.4) | | | 33000 (32000, 35000) | 64000 (62000, 66000) | |  |
| CrI: credible interval, eld.: elderly vaccination change, GP: general practitioner, iTV: improved trivalent vaccine (i.e., adjuvanted or high-dose), paed.: paediatric mass vaccination, QV: quadrivalent vaccine (non-adjuvanted, non-high dose), TV: trivalent vaccine (non-adjuvanted, non-high dose). | | | | | | | |  |
| Supplementary Table 25: Mean (95%-CrI) change of the QALY loss averted as compared to the base case per setting. | | | | | | | | |
| **vaccination strategy** | **symptomatic cases** | **hospitalisations** | | | **excess deaths** | | **total** | |
| **setting** | **Netherlands (NL)** | | | | | | | |
| elderly (iTV) | 210 ( 190, 220) | 7.9 (7.3, 8.4) | | | 2600 (2400, 2800) | | 2800 (2600, 3000) | |
| elderly (QV) | 39.0 (35.2, 42.3) | 5.3 (4.8, 5.8) | | | 1800 (1600, 1900) | | 1800 (1600, 2000) | |
| paed. (TV), 10% | 400 ( 390, 410) | 6.2 (6.0, 6.4) | | | 970 ( 910, 1000) | | 1400 (1300, 1400) | |
| paed. (TV), 25% | 980 ( 950, 1000) | 15.4 (14.9, 16.0) | | | 2400 (2200, 2500) | | 3400 (3200, 3500) | |
| paed. (TV), 50% | 1600 (1600, 1700) | 28.2 (27.0, 29.3) | | | 4300 (4000, 4600) | | 6000 (5700, 6300) | |
| paed. (TV), 75% | 2000 (1900, 2100) | 36.8 (35.2, 38.2) | | | 5700 (5300, 6000) | | 7800 (7300, 8200) | |
| paed. (QV), 10% | 420 ( 410, 440) | 7.3 (7.1, 7.6) | | | 1100 (1100, 1200) | | 1600 (1500, 1600) | |
| paed. (QV), 25% | 1000 (1000, 1100) | 17.9 (17.2, 18.5) | | | 2800 (2600, 2900) | | 3800 (3600, 4000) | |
| paed. (QV), 50% | 1700 (1600, 1800) | 32.0 (30.7, 33.3) | | | 5000 (4600, 5200) | | 6700 (6300, 7100) | |
| paed. (QV), 75% | 2100 (2000, 2200) | 42.3 (40.4, 44.0) | | | 6600 (6200, 7000) | | 8800 (8300, 9300) | |
| eld. (iTV) + paed. (TV), 10% | 590 ( 570, 620) | 13.6 (13.0, 14.2) | | | 3400 (3200, 3600) | | 4000 (3800, 4200) | |
| eld. (iTV) + paed. (TV), 25% | 1200 (1100, 1200) | 22.3 (21.4, 23.1) | | | 4600 (4300, 4800) | | 5800 (5500, 6000) | |
| eld. (iTV) + paed. (TV), 50% | 1800 (1700, 1900) | 34.0 (32.7, 35.3) | | | 6200 (5800, 6500) | | 8000 (7600, 8400) | |
| eld. (iTV) + paed. (TV), 75% | 2200 (2100, 2200) | 41.9 (40.3, 43.5) | | | 7300 (6900, 7700) | | 9500 (9000, 10000) | |
| eld. (QV) + paed. (TV), 10% | 440 ( 420, 450) | 11.4 (10.9, 12.0) | | | 2700 (2500, 2900) | | 3200 (3000, 3300) | |
| eld. (QV) + paed. (TV), 25% | 1000 ( 990, 1100) | 20.5 (19.8, 21.3) | | | 4000 (3800, 4300) | | 5100 (4800, 5300) | |
| eld. (QV) + paed. (TV), 50% | 1700 (1600, 1800) | 33.0 (31.7, 34.3) | | | 5900 (5500, 6200) | | 7600 (7200, 8000) | |
| eld. (QV) + paed. (TV), 75% | 2100 (2000, 2200) | 41.6 (39.9, 43.2) | | | 7200 (6800, 7600) | | 9300 (8800, 9800) | |
| eld. (iTV) + paed. (QV), 10% | 620 ( 600, 640) | 14.7 (14.1, 15.3) | | | 3600 (3400, 3800) | | 4200 (4000, 4400) | |
| eld. (iTV) + paed. (QV), 25% | 1200 (1200, 1300) | 24.6 (23.7, 25.5) | | | 4900 (4600, 5200) | | 6200 (5800, 6400) | |
| eld. (iTV) + paed. (QV), 50% | 1900 (1800, 2000) | 37.7 (36.2, 39.2) | | | 6700 (6400, 7100) | | 8700 (8200, 9100) | |
| eld. (iTV) + paed. (QV), 75% | 2300 (2200, 2400) | 47.3 (45.2, 49.1) | | | 8100 (7600, 8600) | | 10000 (9900, 11000) | |
| eld. (QV) + paed. (QV), 10% | 460 ( 450, 480) | 12.5 (11.9, 13.0) | | | 2800 (2600, 3000) | | 3300 (3100, 3500) | |
| eld. (QV) + paed. (QV), 25% | 1100 (1000, 1100) | 22.8 (21.9, 23.7) | | | 4300 (4100, 4600) | | 5400 (5200, 5700) | |
| eld. (QV) + paed. (QV), 50% | 1800 (1700, 1800) | 36.7 (35.2, 38.2) | | | 6400 (6000, 6800) | | 8200 (7800, 8600) | |
| eld. (QV) + paed. (QV), 75% | 2200 (2100, 2300) | 46.8 (44.7, 48.7) | | | 8000 (7500, 8400) | | 10000 (9600, 11000) | |
| **setting** | **England (EN)** | | | | | | | |
| elderly (iTV) | 910 ( 830, 980) | 20.2 (18.8, 21.6) | | | 5800 (5400, 6200) | | 6800 (6300, 7200) | |
| elderly (QV) | 200 ( 180, 220) | 16.5 (14.8, 18.0) | | | 4800 (4300, 5200) | | 5000 (4500, 5500) | |
| paed. (TV), 10% | 2000 (1900, 2100) | 20.7 (19.8, 21.7) | | | 3200 (3000, 3400) | | 5200 (4900, 5500) | |
| paed. (TV), 25% | 4900 ( 4700, 5200) | 51.7 (48.9, 54.6) | | | 8000 ( 7500, 8600) | | 13000 (12000, 14000) | |
| paed. (TV), 50% | 8100 ( 7700, 8600) | 90.5 (85.9, 95.2) | | | 14000 (13000, 15000) | | 22000 (21000, 24000) | |
| paed. (TV), 75% | 9800 ( 9300, 10000) | 110 ( 110, 120) | | | 18000 (16000, 19000) | | 28000 (26000, 29000) | |
| paed. (QV), 10% | 2100 (2100, 2200) | 26.0 (24.8, 27.1) | | | 4000 (3700, 4200) | | 6100 (5800, 6400) | |
| paed. (QV), 25% | 5200 ( 5000, 5500) | 62.2 (59.1, 65.4) | | | 9600 ( 9000, 10000) | | 15000 (14000, 16000) | |
| paed. (QV), 50% | 8600 ( 8100, 9100) | 110 ( 100, 110) | | | 17000 (16000, 18000) | | 26000 (24000, 27000) | |
| paed. (QV), 75% | 10000 ( 9900, 11000) | 140 ( 130, 140) | | | 22000 (20000, 23000) | | 32000 (31000, 34000) | |
| eld. (iTV) + paed. (TV), 10% | 2900 ( 2800, 3000) | 40.2 (38.3, 42.0) | | | 8800 ( 8300, 9200) | | 12000 (11000, 12000) | |
| eld. (iTV) + paed. (TV), 25% | 5800 ( 5500, 6100) | 69.8 (66.5, 73.0) | | | 13000 (12000, 14000) | | 19000 (18000, 20000) | |
| eld. (iTV) + paed. (TV), 50% | 8900 ( 8400, 9400) | 110 ( 100, 110) | | | 19000 (18000, 20000) | | 28000 (26000, 29000) | |
| eld. (iTV) + paed. (TV), 75% | 11000 (10000, 11000) | 130 ( 120, 130) | | | 22000 (21000, 23000) | | 32000 (31000, 34000) | |
| eld. (QV) + paed. (TV), 10% | 2200 (2100, 2300) | 36.9 (34.8, 38.8) | | | 7800 (7300, 8400) | | 10000 (9400, 11000) | |
| eld. (QV) + paed. (TV), 25% | 5100 ( 4900, 5400) | 67.3 (63.9, 70.8) | | | 13000 (12000, 13000) | | 18000 (17000, 19000) | |
| eld. (QV) + paed. (TV), 50% | 8300 ( 7800, 8800) | 110 (100.0, 110) | | | 18000 (17000, 19000) | | 27000 (25000, 28000) | |
| eld. (QV) + paed. (TV), 75% | 10000 ( 9500, 11000) | 130 ( 120, 130) | | | 21000 (20000, 23000) | | 32000 (30000, 33000) | |
| eld. (iTV) + paed. (QV), 10% | 3000 ( 2900, 3200) | 45.3 (43.3, 47.2) | | | 9500 ( 9000, 10000) | | 13000 (12000, 13000) | |
| eld. (iTV) + paed. (QV), 25% | 6100 ( 5800, 6400) | 80.1 (76.5, 83.6) | | | 15000 (14000, 15000) | | 21000 (20000, 22000) | |
| eld. (iTV) + paed. (QV), 50% | 9400 ( 8900, 9900) | 120 ( 120, 130) | | | 21000 (20000, 22000) | | 31000 (29000, 32000) | |
| eld. (iTV) + paed. (QV), 75% | 11000 (11000, 12000) | 150 ( 140, 160) | | | 26000 (24000, 27000) | | 37000 (35000, 39000) | |
| eld. (QV) + paed. (QV), 10% | 2300 (2300, 2400) | 41.9 (39.6, 43.9) | | | 8500 (7900, 9100) | | 11000 (10000, 12000) | |
| eld. (QV) + paed. (QV), 25% | 5400 ( 5200, 5700) | 77.3 (73.7, 81.1) | | | 14000 (13000, 15000) | | 19000 (18000, 20000) | |
| eld. (QV) + paed. (QV), 50% | 8800 ( 8400, 9300) | 120 ( 120, 130) | | | 21000 (20000, 22000) | | 30000 (28000, 31000) | |
| eld. (QV) + paed. (QV), 75% | 11000 (10000, 11000) | 150 ( 140, 160) | | | 25000 (24000, 26000) | | 36000 (34000, 38000) | |
| CrI: credible interval, eld.: elderly vaccination change, GP: general practitioner, iTV: improved trivalent vaccine (i.e., adjuvanted or high-dose), paed.: paediatric mass vaccination, QV: quadrivalent vaccine (non-adjuvanted, non-high dose), TV: trivalent vaccine (non-adjuvanted, non-high dose). | | | | | | | | |
| Supplementary Table 26: Mean (95%-CrI) change of the QALY loss averted as compared to the base case per setting. | | | | | | | | |
| **vaccination strategy** | **symptomatic cases** | **hospitalisations** | | | **excess deaths** | | **total** | |
| **setting** | **Scotland (SC)** | | | | | | | |
| elderly (iTV) | 180 (160, 190) | 3.0 (2.8, 3.2) | | | 650 (610, 690) | | 830 (780, 880) | |
| elderly (QV) | 33.8 (30.8, 36.5) | 1.9 (1.7, 2.0) | | | 400 (360, 440) | | 440 (400, 470) | |
| paed. (TV), 10% | 130 (82.4, 160) | 1.2 (0.8, 1.4) | | | 250 (160, 300) | | 380 (250, 460) | |
| paed. (TV), 25% | 280 (200, 340) | 2.6 (2.0, 3.1) | | | 550 (400, 650) | | 830 (600, 990) | |
| paed. (TV), 50% | 480 ( 390, 550) | 4.7 (4.0, 5.2) | | | 960 ( 800, 1100) | | 1400 (1200, 1600) | |
| paed. (TV), 75% | 650 ( 560, 730) | 6.4 (5.7, 6.9) | | | 1300 (1100, 1400) | | 2000 (1700, 2100) | |
| paed. (QV), 10% | 140 (89.9, 170) | 1.4 (1.0, 1.7) | | | 290 (200, 350) | | 430 (300, 520) | |
| paed. (QV), 25% | 300 (210, 360) | 3.0 (2.3, 3.5) | | | 620 (470, 730) | | 920 (680, 1100) | |
| paed. (QV), 50% | 500 ( 410, 570) | 5.2 (4.5, 5.8) | | | 1100 ( 910, 1200) | | 1600 (1300, 1800) | |
| paed. (QV), 75% | 670 ( 580, 740) | 6.8 (6.1, 7.4) | | | 1400 (1200, 1500) | | 2100 (1800, 2200) | |
| eld. (iTV) + paed. (TV), 10% | 300 ( 250, 340) | 4.1 (3.7, 4.4) | | | 880 ( 790, 950) | | 1200 (1100, 1300) | |
| eld. (iTV) + paed. (TV), 25% | 450 ( 370, 510) | 5.5 (4.8, 6.0) | | | 1200 (1000, 1300) | | 1600 (1400, 1800) | |
| eld. (iTV) + paed. (TV), 50% | 650 ( 560, 720) | 7.4 (6.7, 8.0) | | | 1600 (1400, 1700) | | 2200 (2000, 2400) | |
| eld. (iTV) + paed. (TV), 75% | 820 ( 730, 890) | 9.1 (8.3, 9.6) | | | 1900 (1700, 2000) | | 2700 (2500, 2900) | |
| eld. (QV) + paed. (TV), 10% | 160 (120, 190) | 3.0 (2.6, 3.3) | | | 640 (550, 710) | | 810 (680, 900) | |
| eld. (QV) + paed. (TV), 25% | 320 (230, 380) | 4.5 (3.8, 4.9) | | | 930 (790, 1000) | | 1300 (1000, 1400) | |
| eld. (QV) + paed. (TV), 50% | 510 ( 420, 580) | 6.4 (5.7, 7.0) | | | 1300 (1200, 1500) | | 1800 (1600, 2000) | |
| eld. (QV) + paed. (TV), 75% | 690 ( 600, 760) | 8.0 (7.3, 8.6) | | | 1600 (1500, 1800) | | 2300 (2100, 2500) | |
| eld. (iTV) + paed. (QV), 10% | 310 ( 260, 350) | 4.3 (3.9, 4.6) | | | 930 ( 830, 1000) | | 1200 (1100, 1300) | |
| eld. (iTV) + paed. (QV), 25% | 460 ( 380, 530) | 5.8 (5.2, 6.3) | | | 1200 (1100, 1400) | | 1700 (1500, 1900) | |
| eld. (iTV) + paed. (QV), 50% | 670 ( 580, 730) | 8.0 (7.2, 8.5) | | | 1700 (1500, 1800) | | 2300 (2100, 2500) | |
| eld. (iTV) + paed. (QV), 75% | 840 ( 740, 910) | 9.5 (8.7, 10.1) | | | 2000 (1800, 2100) | | 2800 (2500, 3000) | |
| eld. (QV) + paed. (QV), 10% | 170 (120, 200) | 3.2 (2.8, 3.5) | | | 680 (590, 750) | | 860 (720, 950) | |
| eld. (QV) + paed. (QV), 25% | 330 ( 250, 390) | 4.8 (4.1, 5.3) | | | 1000 ( 850, 1100) | | 1300 (1100, 1500) | |
| eld. (QV) + paed. (QV), 50% | 530 ( 440, 600) | 6.9 (6.2, 7.5) | | | 1400 (1300, 1600) | | 2000 (1700, 2200) | |
| eld. (QV) + paed. (QV), 75% | 700 ( 610, 770) | 8.4 (7.7, 9.0) | | | 1700 (1600, 1800) | | 2400 (2200, 2600) | |
| **setting** | **Ireland (IE)** | | | | | | | |
| elderly (iTV) | 64.3 (59.0, 68.9) | 0.7 (0.7, 0.7) | | | 240 (230, 260) | | 310 (290, 330) | |
| elderly (QV) | 7.9 (7.0, 8.6) | 0.3 (0.3, 0.4) | | | 110 (95.7, 120) | | 120 (100, 130) | |
| paed. (TV), 10% | 310 (300, 310) | 1.4 (1.3, 1.4) | | | 180 (160, 190) | | 480 (460, 500) | |
| paed. (TV), 25% | 730 ( 690, 750) | 3.4 (3.2, 3.5) | | | 440 ( 400, 470) | | 1200 (1100, 1200) | |
| paed. (TV), 50% | 1100 (1000, 1100) | 5.3 (5.1, 5.5) | | | 730 ( 670, 770) | | 1800 (1700, 1900) | |
| paed. (TV), 75% | 1400 (1300, 1400) | 6.7 (6.3, 6.9) | | | 930 ( 860, 990) | | 2300 (2200, 2400) | |
| paed. (QV), 10% | 320 (310, 330) | 1.6 (1.5, 1.6) | | | 200 (180, 210) | | 510 (490, 530) | |
| paed. (QV), 25% | 760 ( 730, 780) | 3.9 (3.7, 4.0) | | | 490 ( 460, 520) | | 1300 (1200, 1300) | |
| paed. (QV), 50% | 1100 (1100, 1200) | 6.2 (5.9, 6.4) | | | 830 ( 770, 880) | | 2000 (1900, 2100) | |
| paed. (QV), 75% | 1400 (1400, 1500) | 7.9 (7.6, 8.2) | | | 1100 (1000, 1200) | | 2500 (2400, 2600) | |
| eld. (iTV) + paed. (TV), 10% | 370 (360, 380) | 2.1 (2.0, 2.1) | | | 410 (380, 430) | | 780 (740, 810) | |
| eld. (iTV) + paed. (TV), 25% | 790 ( 750, 810) | 4.0 (3.8, 4.1) | | | 650 ( 600, 680) | | 1400 (1400, 1500) | |
| eld. (iTV) + paed. (TV), 50% | 1100 (1100, 1200) | 5.9 (5.6, 6.1) | | | 910 ( 840, 960) | | 2100 (1900, 2100) | |
| eld. (iTV) + paed. (TV), 75% | 1400 (1300, 1500) | 7.2 (6.8, 7.4) | | | 1100 (1000, 1200) | | 2500 (2400, 2600) | |
| eld. (QV) + paed. (TV), 10% | 310 (300, 320) | 1.7 (1.6, 1.8) | | | 280 (260, 300) | | 590 (570, 620) | |
| eld. (QV) + paed. (TV), 25% | 730 ( 700, 760) | 3.7 (3.5, 3.8) | | | 530 ( 500, 570) | | 1300 (1200, 1300) | |
| eld. (QV) + paed. (TV), 50% | 1100 (1000, 1100) | 5.6 (5.3, 5.8) | | | 810 ( 750, 860) | | 1900 (1800, 2000) | |
| eld. (QV) + paed. (TV), 75% | 1400 (1300, 1400) | 6.9 (6.6, 7.2) | | | 1000 ( 940, 1100) | | 2400 (2300, 2500) | |
| eld. (iTV) + paed. (QV), 10% | 380 (370, 390) | 2.2 (2.2, 2.3) | | | 420 (400, 450) | | 810 (770, 840) | |
| eld. (iTV) + paed. (QV), 25% | 820 ( 780, 850) | 4.5 (4.3, 4.7) | | | 700 ( 650, 740) | | 1500 (1500, 1600) | |
| eld. (iTV) + paed. (QV), 50% | 1200 (1100, 1200) | 6.7 (6.4, 7.0) | | | 1000 ( 940, 1100) | | 2200 (2100, 2300) | |
| eld. (iTV) + paed. (QV), 75% | 1500 (1400, 1500) | 8.4 (8.1, 8.7) | | | 1200 (1200, 1300) | | 2700 (2600, 2900) | |
| eld. (QV) + paed. (QV), 10% | 330 (320, 330) | 1.9 (1.8, 1.9) | | | 300 (280, 310) | | 620 (600, 650) | |
| eld. (QV) + paed. (QV), 25% | 760 ( 730, 790) | 4.2 (4.0, 4.3) | | | 580 ( 540, 620) | | 1400 (1300, 1400) | |
| eld. (QV) + paed. (QV), 50% | 1200 (1100, 1200) | 6.5 (6.2, 6.7) | | | 900 ( 840, 960) | | 2100 (2000, 2100) | |
| eld. (QV) + paed. (QV), 75% | 1400 (1400, 1500) | 8.1 (7.8, 8.4) | | | 1100 (1100, 1200) | | 2600 (2500, 2700) | |
| CrI: credible interval, eld.: elderly vaccination change, GP: general practitioner, iTV: improved trivalent vaccine (i.e., adjuvanted or high-dose), paed.: paediatric mass vaccination, QV: quadrivalent vaccine (non-adjuvanted, non-high dose), TV: trivalent vaccine (non-adjuvanted, non-high dose). | | | | | | | | |

Cost-effectiveness acceptability curve (CEAC) and frontier (CEAF) per setting


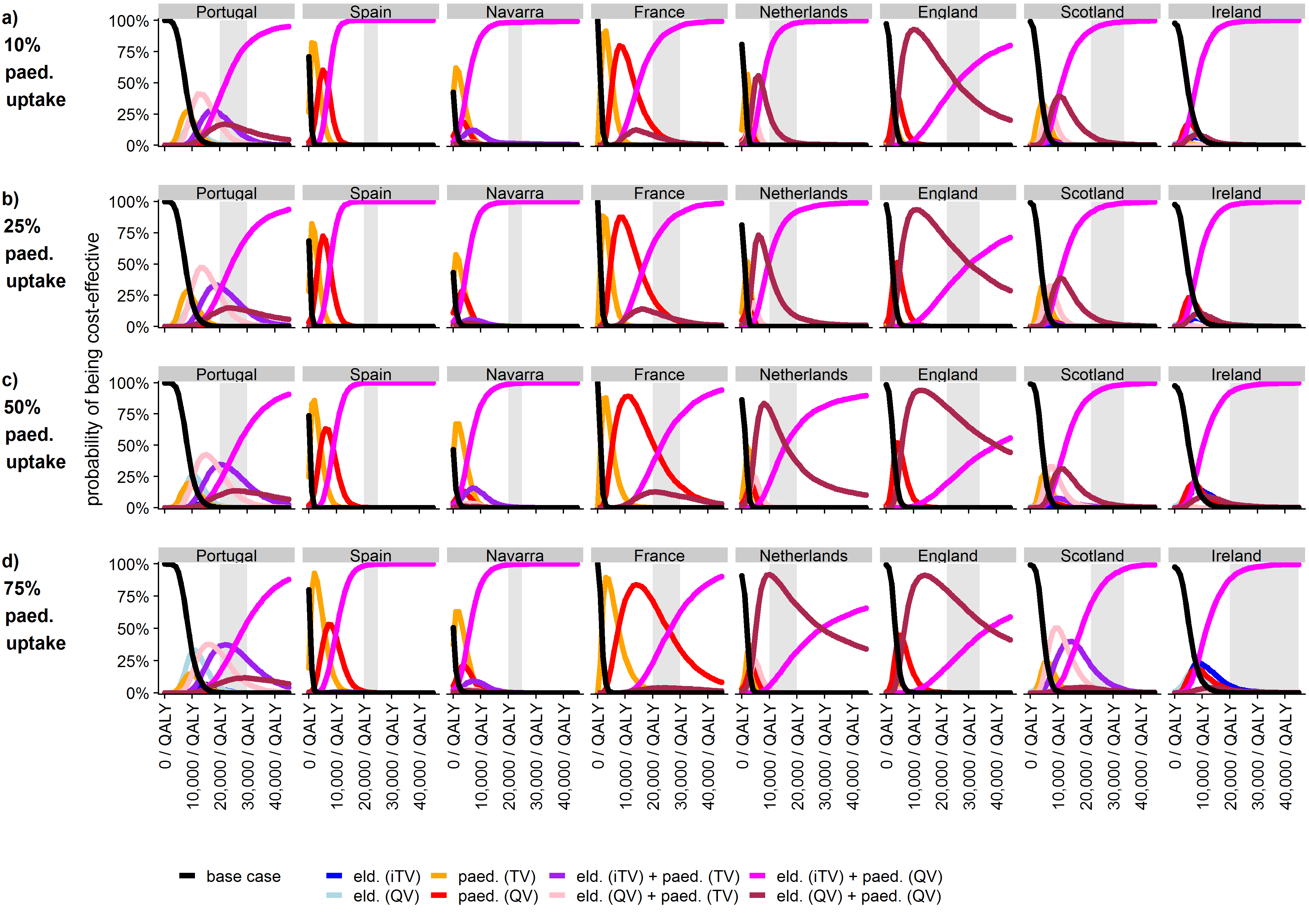
Supplementary Figure 58. Probability of vaccination strategies being cost-effective over willingness-to-pay ranges of €0-€45,000/QALY.


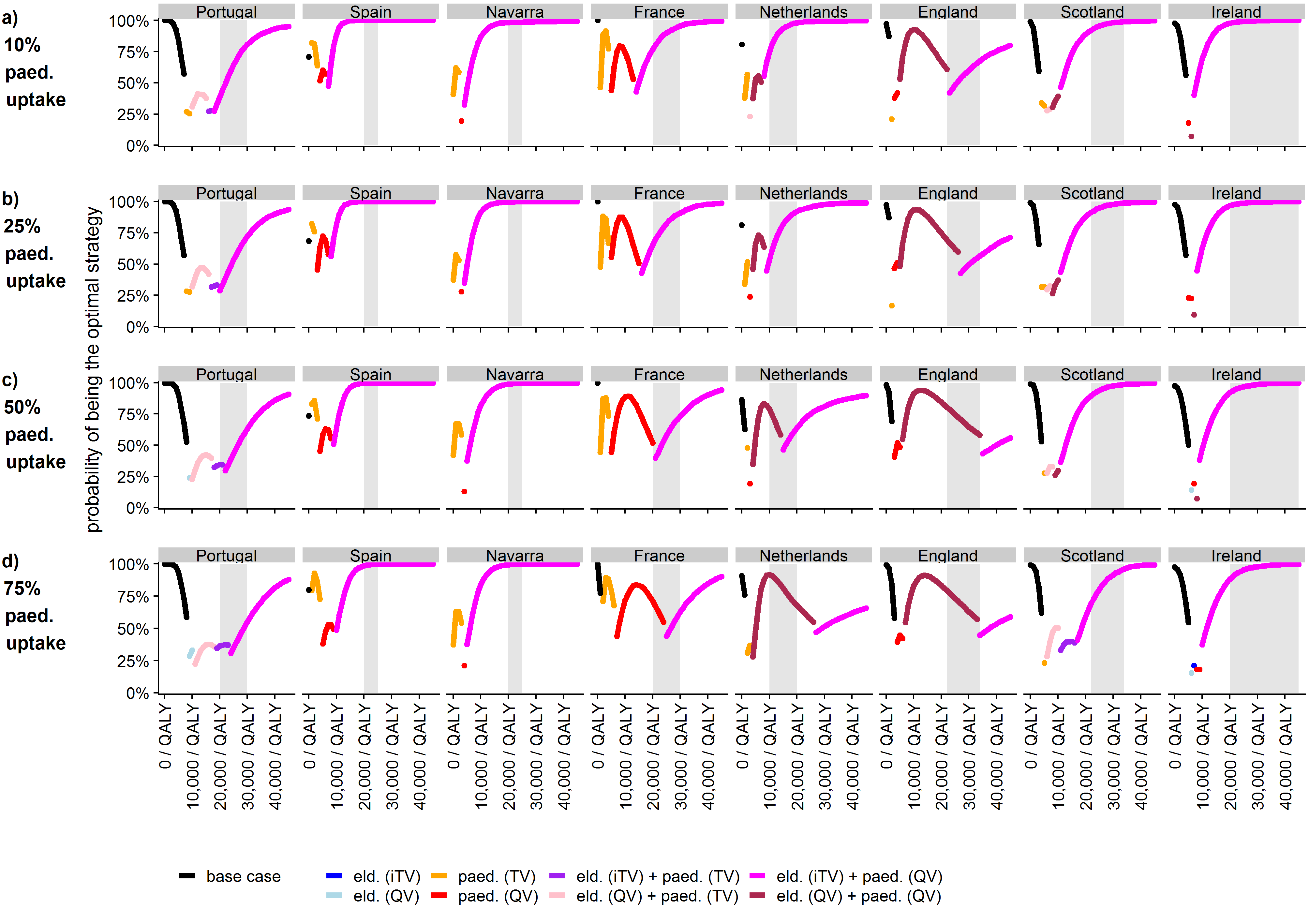
Supplementary Figure 59. Optimal vaccination strategy for willingness-to-pay ranges of €0-€45,000/QALY per setting (cost-effectiveness acceptability frontier, CEAF).

Threshold analysis of the iTV price per dose (based on cost-effectiveness acceptability frontier, CEAF)


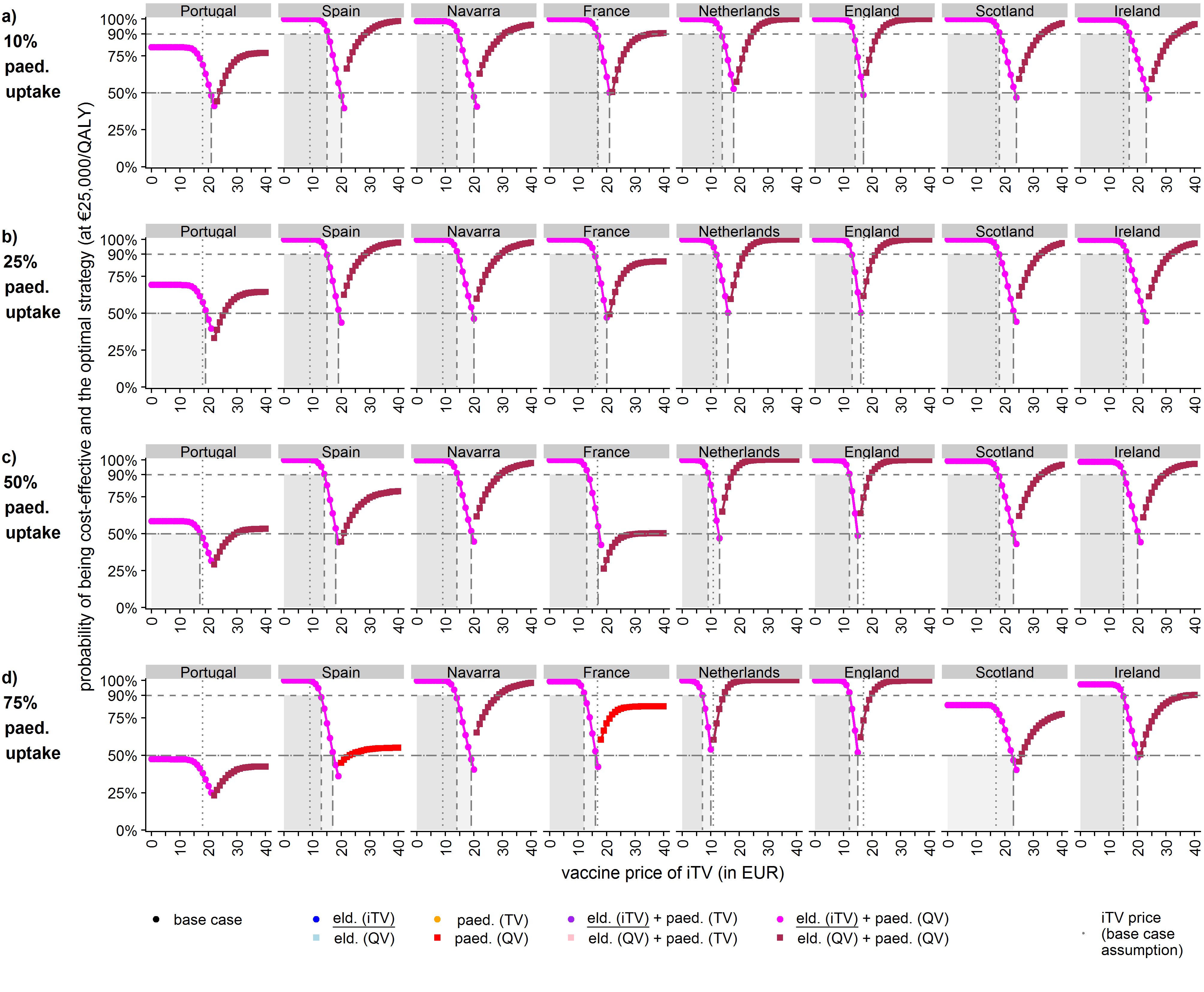
Supplementary Figure 60. Threshold analysis of the iTV price per dose at which the probability of being cost-effective and being the optimal strategy changes across settings (based on cost-effectiveness acceptability frontier, CEAF).

CEAF: cost-effectiveness acceptability frontier, eld.: elderly vaccination change (moving from TV to iTV or QV), EUR: euros, iTV: “improved” trivalent vaccine (i.e., adjuvanted or high-dose), QALY: quality-adjusted life year, QV: quadrivalent vaccine (non-adjuvanted, non-high dose), TV: trivalent vaccine (non-adjuvanted, non-high dose).

CHEERS checklist - Items to include when reporting economic evaluations of health interventions

| Section/item | Item No | Recommendation | Reported on page No/ line No |
| --- | --- | --- | --- |
| **Title and abstract** | | | |
| Title | 1 | Identify the study as an economic evaluation or use more specific terms such as “cost-effectiveness analysis”, and describe the interventions compared. | Title, p. 1 |
| Abstract | 2 | Provide a structured summary of objectives, perspective, setting, methods (including study design and inputs), results (including base case and uncertainty analyses), and conclusions. | Summary, p. 3 |
| **Introduction** | | | |
| Background and objectives | 3 | Provide an explicit statement of the broader context for the study. | Research in context, p 4, and Introduction, p. 5 |
|  |  | Present the study question and its relevance for health policy or practice decisions. | Introduction, p. 5 |
| **Methods** | | | |
| Target population and subgroups | 4 | Describe characteristics of the base case population and subgroups analysed, including why they were chosen. | Methods, p. 6 |
| Setting and location | 5 | State relevant aspects of the system(s) in which the decision(s) need(s) to be made. | Methods, p. 5 |
| Study perspective | 6 | Describe the perspective of the study and relate this to the costs being evaluated. | Methods, p. 6 |
| Comparators | 7 | Describe the interventions or strategies being compared and state why they were chosen. | Methods, p. 6, and Table 1 |
| Time horizon | 8 | State the time horizon(s) over which costs and consequences are being evaluated and say why appropriate. | Methods, p. 6 |
| Discount rate | 9 | Report the choice of discount rate(s) used for costs and outcomes and say why appropriate. | Methods, p. 6 |
| Choice of health outcomes | 10 | Describe what outcomes were used as the measure(s) of benefit in the evaluation and their relevance for the type of analysis performed. | Methods, p. 6 |
| Measurement of effectiveness | 11a | *Single study-based estimates:*Describe fully the design features of the single effectiveness study and why the single study was a sufficient source of clinical effectiveness data. | n/a |
|  | 11b | *Synthesis-based estimates:*Describe fully the methods used for identification of included studies and synthesis of clinical effectiveness data. | Methods, p. 5-6; Appendix, pp. 5-7 |
| Measurement and valuation of preference based outcomes | 12 | If applicable, describe the population and methods used to elicit preferences for outcomes. | n/a |
| Estimating resources and costs | 13a | *Single study-based economic evaluation:*Describe approaches used to estimate resource use associated with the alternative interventions. Describe primary or secondary research methods for valuing each resource item in terms of its unit cost. Describe any adjustments made to approximate to opportunity costs. | n/a |
|  | 13b | *Model-based economic evaluation:*Describe approaches and data sources used to estimate resource use associated with model health states. Describe primary or secondary research methods for valuing each resource item in terms of its unit cost. Describe any adjustments made to approximate to opportunity costs. | Methods, pp. 5-7, and Appendix, pp. 2-7 |
| Currency, price date, and conversion | 14 | Report the dates of the estimated resource quantities and unit costs. Describe methods for adjusting estimated unit costs to the year of reported costs if necessary. Describe methods for converting costs into a common currency base and the exchange rate. | Methods, p. 6 |
| Choice of model | 15 | Describe and give reasons for the specific type of decision-analytical model used. Providing a figure to show model structure is strongly recommended. | Methods, p. 5, and Appendix, pp. 2-3 |
| Assumptions | 16 | Describe all structural or other assumptions underpinning the decision-analytical model. | Methods, p. 5, and Appendix, pp. 2-3 |
| Analytical methods | 17 | Describe all analytical methods supporting the evaluation. This could include methods for dealing with skewed, missing, or censored data; extrapolation methods; methods for pooling data; approaches to validate or make adjustments (such as half cycle corrections) to a model; and methods for handling population heterogeneity and uncertainty. | Methods, pp. 5-7, and Appendix, pp. 2-7 |
| **Results** | | | |
| Study parameters | 18 | Report the values, ranges, references, and, if used, probability distributions for all parameters. Report reasons or sources for distributions used to represent uncertainty where appropriate. Providing a table to show the input values is strongly recommended. | Methods, p. 7, and Appendix, pp. 2-7 |
| Incremental costs and outcomes | 19 | For each intervention, report mean values for the main categories of estimated costs and outcomes of interest, as well as mean differences between the comparator groups. If applicable, report incremental cost-effectiveness ratios. | Appendix, pp. 38-52 |
| Characterising uncertainty | 20a | *Single study-based economic evaluation:*Describe the effects of sampling uncertainty for the estimated incremental cost and incremental effectiveness parameters, together with the impact of methodological assumptions (such as discount rate, study perspective). | n/a |
|  | 20b | *Model-based economic evaluation:*Describe the effects on the results of uncertainty for all input parameters, and uncertainty related to the structure of the model and assumptions. | Methods, p. 7, and Appendix, pp. 2-8 |
| Characterising heterogeneity | 21 | If applicable, report differences in costs, outcomes, or cost-effectiveness that can be explained by variations between subgroups of patients with different baseline characteristics or other observed variability in effects that are not reducible by more information. | Methods, p. 7, and Appendix, pp. 2-8 |
| **Discussion** | | | |
| Study findings, limitations, generalisability, and current knowledge | 22 | Summarise key study findings and describe how they support the conclusions reached. Discuss limitations and the generalisability of the findings and how the findings fit with current knowledge. | Discussion, pp. 10-11 |
| **Other** | | | |
| Source of funding | 23 | Describe how the study was funded and the role of the funder in the identification, design, conduct, and reporting of the analysis. Describe other non-monetary sources of support. | Title page, p. 2, and Methods, p. 7 |
| Conflicts of interest | 24 | Describe any potential for conflict of interest of study contributors in accordance with journal policy. In the absence of a journal policy, we recommend authors comply with International Committee of Medical Journal Editors recommendations. | Title page, p. 2 |

For consistency, the CHEERS statement checklist format is based on the format of the CONSORT statement checklist

## References

1. Baguelin M, Flasche S, Camacho A, Demiris N, Miller E, Edmunds WJ. Assessing optimal target populations for influenza vaccination programmes: an evidence synthesis and modelling study. *PLoS Med* 2013; **10**(10): e1001527.

2. Baguelin M, Camacho A, Flasche S, Edmunds WJ. Extending the elderly- and risk-group programme of vaccination against seasonal influenza in England and Wales: a cost-effectiveness study. *BMC Med* 2015; **13**: 236.

3. Ferguson NM, Cummings DA, Cauchemez S, et al. Strategies for containing an emerging influenza pandemic in Southeast Asia. *Nature* 2005; **437**(7056): 209-14.

4. Kissling E, Valenciano M, Cohen JM, et al. I-MOVE Multi-Centre Case Control Study 2010-11: Overall and Stratified Estimates of Influenza Vaccine Effectiveness in Europe. *PLoS ONE* 2011; **6**(11).

5. Kissling E, Valenciano M, Larrauri A, et al. Low and decreasing vaccine effectiveness against influenza A(H3) in 2011/12 among vaccination target groups in Europe: results from the I-MOVE multicentre case–control study. *Eurosurveillance* 2013; **18**(5): 20390.

6. Kissling E, Valenciano M, Buchholz U, et al. Influenza vaccine effectiveness estimates in Europe in a season with three influenza type/subtypes circulating: the I-MOVE multicentre case–control study, influenza season 2012/13. *Eurosurveillance* 2014; **19**(6): 20701.

7. Valenciano M, Kissling E, Reuss A, et al. The European I-MOVE Multicentre 2013–2014 Case-Control Study. Homogeneous moderate influenza vaccine effectiveness against A(H1N1)pdm09 and heterogenous results by country against A(H3N2). *Vaccine* 2015; **33**(24): 2813-22.

8. Valenciano M, Kissling E, Reuss A, et al. Vaccine effectiveness in preventing laboratory-confirmed influenza in primary care patients in a season of co-circulation of influenza A(H1N1)pdm09, B and drifted A(H3N2), I-MOVE Multicentre Case–Control Study, Europe 2014/15. *Eurosurveillance* 2016; **21**(7): 30139.

9. Kissling E, Valenciano M, Pozo F, et al. 2015/16 I-MOVE/I-MOVE+ multicentre case-control study in Europe: Moderate vaccine effectiveness estimates against influenza A(H1N1)pdm09 and low estimates against lineage-mismatched influenza B among children. *Influenza and Other Respiratory Viruses* 2018; **12**(4): 423-37.

10. Kissling E, Rondy M, Team I-MI-Ms. Early 2016/17 vaccine effectiveness estimates against influenza A(H3N2): I-MOVE multicentre case control studies at primary care and hospital levels in Europe. *Eurosurveillance* 2017; **22**(7): 30464.

11. Belongia EA, Simpson MD, King JP, et al. Variable influenza vaccine effectiveness by subtype: a systematic review and meta-analysis of test-negative design studies. *Lancet Infect Dis* 2016; **16**(8): 942-51.

12. Carrat F, Vergu E, Ferguson NM, et al. Time lines of infection and disease in human influenza: a review of volunteer challenge studies. *American journal of epidemiology* 2008; **167**(7): 775-85.

13. Friesema IH, Koppeschaar CE, Donker GA, et al. Internet-based monitoring of influenza-like illness in the general population: experience of five influenza seasons in The Netherlands. *Vaccine* 2009; **27**(45): 6353-7.

14. van Noort SP, Codeco CT, Koppeschaar CE, van Ranst M, Paolotti D, Gomes MG. Ten-year performance of Influenzanet: ILI time series, risks, vaccine effects, and care-seeking behaviour. *Epidemics* 2015; **13**: 28-36.

15. Lieberman D, Lieberman D, Shimoni A, Keren-Naus A, Steinberg R, Shemer-Avni Y. Identification of respiratory viruses in adults: nasopharyngeal versus oropharyngeal sampling. *J Clin Microbiol* 2009; **47**(11): 3439-43.

16. World Health Organization. FluID - a global influenza epidemiological data sharing platform. 2018. <http://apps.who.int/flumart/Default?ReportNo=16> (accessed 07/01/2019.

17. Mossong J, Hens N, Jit M, et al. Social contacts and mixing patterns relevant to the spread of infectious diseases. 2008; **5**(3): e74.

18. Béraud G, Kazmercziak S, Beutels P, et al. The French connection: the first large population-based contact survey in France relevant for the spread of infectious diseases. 2015; **10**(7): e0133203.

19. World Health Organization. Guidance on the economic evaluation of influenza vaccination. Geneva: World Health Organization; 2016.

20. World Health Organization. WHO Manual for estimating the economic burden of seasonal influenza. Geneva: World Health Organization; 2016.

21. van Leeuwen E, Klepac P, Thorrington D, Pebody R, Baguelin M. fluEvidenceSynthesis: An R package for evidence synthesis based analysis of epidemiological outbreaks. *PLoS computational biology* 2017; **13**(11): e1005838.

22. Cromer D, van Hoek AJ, Jit M, Edmunds WJ, Fleming D, Miller E. The burden of influenza in England by age and clinical risk group: a statistical analysis to inform vaccine policy. *J Infect* 2014; **68**(4): 363-71.

23. Backer JA, Wallinga J, Meijer A, Donker GA, van der Hoek W, van Boven M. The impact of influenza vaccination on infection, hospitalisation and mortality in the Netherlands between 2003 and 2015. *Epidemics* 2019; **26**: 77-85.

24. Oliva J, Delgado-Sanz C, Larrauri A, Spanish Influenza Surveillance S. Estimating the burden of seasonal influenza in Spain from surveillance of mild and severe influenza disease, 2010-2016. *Influenza Other Respir Viruses* 2018; **12**(1): 161-70.

25. Paolotti D, Carnahan A, Colizza V, et al. Web-based participatory surveillance of infectious diseases: the Influenzanet participatory surveillance experience. *Clinical Microbiology and Infection* 2014; **20**(1): 17-21.

26. Iuliano AD, Roguski KM, Chang HH, et al. Estimates of global seasonal influenza-associated respiratory mortality: a modelling study. *The Lancet* 2018; **391**(10127): 1285-300.

27. Hozo SP, Djulbegovic B, Hozo I. Estimating the mean and variance from the median, range, and the size of a sample. *BMC Med Res Methodol* 2005; **5**: 13.

28. Nunes B, Viboud C, Machado A, et al. Excess mortality associated with influenza epidemics in Portugal, 1980 to 2004. *PloS one* 2011; **6**(6): e20661.

29. Pebody RG, Green HK, Warburton F, et al. Significant spike in excess mortality in England in winter 2014/15 – influenza the likely culprit. *Epidemiology and Infection* 2018; **146**(9): 1106-13.

30. Hollmann M, Garin O, Galante M, Ferrer M, Dominguez A, Alonso J. Impact of influenza on health-related quality of life among confirmed (H1N1) 2009 patients. *PloS one* 2013; **8**(3): e60477.

31. Mangen MJ, Rozenbaum MH, Huijts SM, et al. Cost-effectiveness of adult pneumococcal conjugate vaccination in the Netherlands. *Eur Respir J* 2015; **46**(5): 1407-16.

32. Mangen MJ, Huijts SM, Bonten MJ, de Wit GA. The impact of community-acquired pneumonia on the health-related quality-of-life in elderly. *BMC Infect Dis* 2017; **17**(1): 208.

33. Ferreira LN, Ferreira PL, Pereira LN, Oppe M. The valuation of the EQ-5D in Portugal. *Quality of Life Research* 2014; **23**(2): 413-23.

34. Chevalier J, de Pouvourville G. Valuing EQ-5D using time trade-off in France. *The European journal of health economics : HEPAC : health economics in prevention and care* 2013; **14**(1): 57-66.

35. Bailey H, Kind P. Preliminary findings of an investigation into the relationship between national culture and EQ-5D value sets. *Quality of Life Research* 2010; **19**(8): 1145-54.

36. Ferreira LN, Ferreira PL, Pereira LN, Oppe M. EQ-5D Portuguese population norms. *Quality of Life Research* 2014; **23**(2): 425-30.

37. Badia X, Roset M, Herdman M, Kind P. A comparison of United Kingdom and Spanish general population time trade-off values for EQ-5D health states. *Medical Decision Making* 2001; **21**(1): 7-16.

38. Szende A, Janssen B, Cabases J. Self-reported population health: an international perspective based on EQ-5D: Springer; 2014.

39. Organisation for Economic Co-operation and Development (OECD). Consumer prices - Annual inflation, all items non-food non-energy. 2017a. <http://stats.oecd.org/> (accessed 15.03.2018.

40. Eurostat. Exchange rates - Overview. 2018. <https://ec.europa.eu/eurostat/web/exchange-rates/overview> (accessed 19.03.2018.

41. Ethgen O, Cornier M, Chriv E, Baron-Papillon F. The cost of vaccination throughout life: A western European overview. *Hum Vaccin Immunother* 2016; **12**(8): 2029-37.

42. National Health Care Institute (Zorginstituut Nederland). Guideline for economic evaluations in healthcare. Diemen: National Health Care Institute (Zorginstituut Nederland); 2016.

43. Curtis LA, Burns A. Unit costs of health and social care 2017. Kent: Personal Social Services Research Unit, University of Kent; 2017.

44. Health Service Executive. GP visit cards. 2017. <https://www.hse.ie/eng/cards-schemes/gp-visit-cards/> (accessed 25.04.2018.

45. Torner N, Navas E, Soldevila N, et al. Costs associated with influenza-related hospitalization in the elderly. *Human vaccines & immunotherapeutics* 2017; **13**(2): 412-6.

46. Rozenbaum MH, Mangen M-JJ, Huijts SM, van der Werf TS, Postma MJ. Incidence, direct costs and duration of hospitalization of patients hospitalized with community acquired pneumonia: a nationwide retrospective claims database analysis. *Vaccine* 2015; **33**(28): 3193-9.

47. Kanters TA, Bouwmans CAM, van der Linden N, Tan SS, Hakkaart-van Roijen L. Update of the Dutch manual for costing studies in health care. *PloS one* 2017; **12**(11): e0187477-e.

48. Pitman RJ, Nagy LD, Sculpher MJ. Cost-effectiveness of childhood influenza vaccination in England and Wales: Results from a dynamic transmission model. *Vaccine* 2013; **31**(6): 927-42.

49. NHS Improvement. Reference costs. 2017. <https://improvement.nhs.uk/resources/reference-costs/> (accessed 21.03.2018.

50. ISD Scotland. Acute Medical - Specialty Costs. 2018. <https://www.isdscotland.org/Health-Topics/Finance/Costs/Detailed-Tables/Speciality-Costs/Acute-Medical.asp> (accessed 20.04.2018.

51. Dushoff J, Plotkin JB, Levin SA, Earn DJD. Dynamical resonance can account for seasonality of influenza epidemics. 2004; **101**(48): 16915-6.

52. Kwong JC, Maaten S, Upshur REG, Patrick DM, Marra F. The Effect of Universal Influenza Immunization on Antibiotic Prescriptions: An Ecological Study. *Clinical Infectious Diseases* 2009; **49**(5): 750-6.
